# Supplementary material for: Rhodanese-Fold Containing Proteins in Humans: Not Just Key Players in Sulfur Trafficking
Source: Antioxidants (Basel). 2023 Mar 31;12(4):843. doi: 10.3390/antiox12040843 (PMC10135228; doi:10.3390/antiox12040843)
Supplement: Supplementary file 1 [file antioxidants-12-00843-s001.zip › antioxidants-2288036-supplementary.docx]

Supplemental file 1:

Sequence from mammals, plants, yeast or bacteria used for Rhd domain conserved motives DxR and CxxGxR, retrieve from BlastP using TSTD1 as template.

>sp|Q5T7W7|TSTD2_HUMAN Thiosulfate sulfurtransferase/rhodanese-like domain-containing protein 2 OS=Homo sapiens OX=9606 GN=TSTD2 PE=1 SV=1

MPSSTSPDQGDDLENCILRFSDLDLKDMSLINPSSSLKAELDGSTKKKYSFAKKKAFALFVKTKEVPTKRSFECKEKLWKCCRQLFTDQTSIHRHVATQHADEIYHQTASILKQLAVTLSTSKSLSSADEKNPLKECLPHSHDVSAWLPDISCFNPDELISGQGSEEGEVLLYYCYHDLEDPQWICAWQTALCQHLHLTGKIRIAAEGINGTVGGSKLATRLYVEVMLSFPLFKDDLCKDDFKTSKGGAHCFPELRVGVFEEIVPMGISPKKISYKKPGIHLSPGEFHKEVEKFLSQANQEQSDTILLDCRNFYESKIGRFQGCLAPDIRKFSYFPSYVDKNLELFREKRVLMYCTGGIRCERGSAYLKAKGVCKEVFQLKGGIHKYLEEFPDGFYKGKLFVFDERYALSYNSDVVSECSYCGARWDQYKLCSTPQCRQLVLTCPACQGQGFTACCVTCQDKGSRKVSGPMQDSFKEECECTARRPRIPRELLQHVRQPVSPEPGPDADEDGPVLM

>sp|Q9M158|STR4_ARATH Rhodanese-like domain-containing protein 4, chloroplastic OS=Arabidopsis thaliana OX=3702 GN=STR4 PE=1 SV=2

MEALKTATFSPMSVLSEKRSEPRKPFSLPNLFPPKSQRPISQESFLKRFNGGLALLTSVLSSATAPAKSLTYEEALQQSMTTSSSFDSDGLIEGISNFVTDNPLVIAGGVAALAVPFVLSQVLNKKPKSWGVESAKNAYTKLGTDDNAQLLDIRATADFRQVGSPNIKGLGKKAVSTVYNGEDKPGFLKKLSLKFKDPENTTLYILDKFDGNSELVAELVALNGFKSAYAIKDGAEGPRGWLNSSLPWIEPKKTLSLDLSSLTDSISGVFGESSDGVSVALGVAAAAGLSVFAFTEIETILQLLGSAALVQLAGKKLLFAEDRKQTLKQVDEFLNTKVAPKELVDELKEIGKALLPQSTSNKALPAPATVTAEAESATATTTTVDKPVPEPETVAATTTTVDKPVPEPEPVPEPVPVPAIEAAVAAQVITEPTETEAKPKPHSRPLSPYASYPDLKPPSSPMPSQP

>sp|Q24JL3|STR2_ARATH Thiosulfate/3-mercaptopyruvate sulfurtransferase 2 OS=Arabidopsis thaliana OX=3702 GN=STR2 PE=1 SV=1

MKRAFSSQLRSAYPASKSTHFGRVMASSGSEAKANYAPISTNEPVVSVDWLHSNLGDADIKVLDASWYMAHEQRNPIQEYQVAHIPGALFFDLNGIADRKTNLRHMLPSEEAFAAGCSALGIENNDGVVVYDGMGLFSAARVWWMFRVFGHDKVWVLDGGLPKWRASGYDVESSVSNDAILKASAATEAIEKIYQGQTISPITFQTKFRPHLVLALDQVKENIEDKTYQHIDARSKARFDGIAPEPWKGLPSGHIPGSKCVPFPLMFDSSQTLLPAEELKKQFEQEDISLDSPIAASCGTGVTACILALGLYRLGKTNVAIYDGSWTEWATAPNLPIVGSSS

>sp|Q16762|THTR_HUMAN Thiosulfate sulfurtransferase OS=Homo sapiens OX=9606 GN=TST PE=1 SV=4

MVHQVLYRALVSTKWLAESIRTGKLGPGLRVLDASWYSPGTREARKEYLERHVPGASFFDIEECRDTASPYEMMLPSEAGFAEYVGRLGISNHTHVVVYDGEHLGSFYAPRVWWMFRVFGHRTVSVLNGGFRNWLKEGHPVTSEPSRPEPAVFKATLDRSLLKTYEQVLENLESKRFQLVDSRSQGRFLGTEPEPDAVGLDSGHIRGAVNMPFMDFLTEDGFEKGPEELRALFQTKKVDLSQPLIATCRKGVTACHVALAAYLCGKPDVAVYDGSWSEWFRRAPPESRVSQGKSEKA

>sp|P31142|THTM_ECOLI 3-mercaptopyruvate sulfurtransferase OS=Escherichia coli (strain K12) OX=83333 GN=sseA PE=1 SV=3

MSTTWFVGADWLAEHIDDPEIQIIDARMASPGQEDRNVAQEYLNGHIPGAVFFDIEALSDHTSPLPHMLPRPETFAVAMRELGVNQDKHLIVYDEGNLFSAPRAWWMLRTFGVEKVSILGGGLAGWQRDDLLLEEGAVELPEGEFNAAFNPEAVVKVTDVLLASHENTAQIIDARPAARFNAEVDEPRPGLRRGHIPGALNVPWTELVREGELKTTDELDAIFFGRGVSYDKPIIVSCGSGVTAAVVLLALATLDVPNVKLYDGAWSEWGARADLPVEPVK

>sp|Q12305|RDL1_YEAST Thiosulfate:glutathione sulfurtransferase OS=Saccharomyces cerevisiae (strain ATCC 204508 / S288c) OX=559292 GN=RDL1 PE=1 SV=1

MWKAVMNAWNGTESQSKNVSNIQSYSFEDMKRIVGKHDPNVVLVDVREPSEYSIVHIPASINVPYRSHPDAFALDPLEFEKQIGIPKPDSAKELIFYCASGKRGGEAQKVASSHGYSNTSLYPGSMNDWVSHGGDKLDL

>sp|O64530|STR1_ARATH Thiosulfate/3-mercaptopyruvate sulfurtransferase 1, mitochondrial OS=Arabidopsis thaliana OX=3702 GN=STR1 PE=1 SV=1

MASTLFSRTFLAASHRLITPSLPQKIFNPATFLSRSLHSQLGSASTAYKSTTWARRAMASTGVETKAGYSTSSVSTSEPVVSVDWLHANLREPDLKILDASWYMPDEQRNPIQEYQVAHIPRALFFDLDGISDRKTSLPHMLPTEEAFAAGCSALGIDNKDEVVVYDGKGIFSAARVWWMFRVFGHEKVWVLDGGLPRWRASGYDVESSASGDAILKASAASEAIEKIYQGQTVSPITFQTKFQPHLVWTLDQVKNNMEDPTYQHIDARSKARFDGTAPEPRKGIRSGHIPGSKCIPFPQMFDSCNTLLPAEELKKRFDQEDISLDKPIMASCGTGVTACILAMGLHRLGKTDVPIYDGSWTEWATQPDLPIESVESSS

>sp|Q8RUD6|HARC1_ARATH Protein HIGH ARSENIC CONTENT 1, mitochondrial OS=Arabidopsis thaliana OX=3702 GN=HAC1 PE=1 SV=1

MYTYSLLNLSHCRRQTRKKRKTDHTEGFLMEETKPKTVEDVETVDVYTAKGFLSTGHRYLDVRTNEEFAKSHVEEALNIPYMFKTDEGRVINPDFLSQVASVCKKDEHLIVACNAGGRGSRACVDLLNEGYDHVANMGGGYSAWVDAGFAGDKPPEDLKIACKFRPKEN

>sp|P00586|THTR_BOVIN Thiosulfate sulfurtransferase OS=Bos taurus OX=9913 GN=TST PE=1 SV=3

MVHQVLYRALVSTKWLAESVRAGKVGPGLRVLDASWYSPGTREARKEYLERHVPGASFFDIEECRDKASPYEVMLPSEAGFADYVGSLGISNDTHVVVYDGDDLGSFYAPRVWWMFRVFGHRTVSVLNGGFRNWLKEGHPVTSEPSRPEPAIFKATLNRSLLKTYEQVLENLESKRFQLVDSRAQGRYLGTQPEPDAVGLDSGHIRGSVNMPFMNFLTEDGFEKSPEELRAMFEAKKVDLTKPLIATCRKGVTACHIALAAYLCGKPDVAIYDGSWFEWFHRAPPETWVSQGKGGKA

>sp|P24329|THTR_RAT Thiosulfate sulfurtransferase OS=Rattus norvegicus OX=10116 GN=Tst PE=1 SV=3

MVHQVLYRALVSTKWLAESIRSGKVGPSLRVLDASWYSPGTRQARKEYQERHVPGASFFDIEECRDTTSPYEMMLPSEAHFGDYVGNLGISNDTHVVVYDGDDLGSFYAPRVWWMFRVFGHRTVSVLNGGFRNWLKEGHPVTSEPSRPEPAVFKATLNRSLLKTYEQVLENLQSKRFQLVDSRAQGRYLGTQPEPDAVGLDSGHIRGSVNVPFMNFLTEDGFEKSPEELRAIFQDKKVDLSQPLIATCRKGVTACHIALAAYLCGKPDVAVYDGSWSEWFRRAPPETRVSQGKSGKA

>sp|P52196|THTR_MOUSE Thiosulfate sulfurtransferase OS=Mus musculus OX=10090 GN=Tst PE=1 SV=3

MVHQVLYRALVSTKWLAESIRSGRLGPSLRVLDASWYSPGTRQARKEYQERHVPGASFFDIEECRDTTSPYEMMLPSEAHFGDYVGNLGISNDTHVVVYDGDDLGSFYAPRVWWMFRVFGHRTVSVLNGGFRNWLKEGHPVTSEPSRPEPAVFKATLNLSLLKTYEQVLENLQSKRFQLVDSRAQGRYLGTQPEPDIVGLDSGHIRGSVNMPFMDFLTKDGFEKSPEELRAIFQDKKVDLSQPLIATCRKGVTACHVALAAYLCGKPDVAVYDGSWSEWFRRAPPETRVSQGKSGKA

>sp|Q9HUK9|THTR_PSEAE Thiosulfate sulfurtransferase OS=Pseudomonas aeruginosa (strain ATCC 15692 / DSM 22644 / CIP 104116 / JCM 14847 / LMG 12228 / 1C / PRS 101 / PAO1) OX=208964 GN=rhdA PE=1 SV=1

MSVFSDLPLVIEPSDLAPRLGAPELILVDLTSAARYAEGHIPGARFVDPKRTQWGQPPAPGLLPAKADLEALFGELGHRPEATYVVYDDEGGGWAGRFIWLLDVIGHHHYHYLNGGLPAWIADAQALDREVPAPVGGPLPLTLHDEPSATREYLQSRLGAADLAVWDARNPSEYAGTKVLAAKAGHVPGAINFEWTAGMDPARALRIRADIAEVLEDLGITPDKEVITHCQTHHRSGFTYLVAKALGYPRVKGYAGSWSEWGNHPDTPVEV

>sp|Q39129|STR16_ARATH Thiosulfate sulfurtransferase 16, chloroplastic OS=Arabidopsis thaliana OX=3702 GN=STR16 PE=1 SV=2

MAEESRVPSSVSVTVAHDLLLAGHRYLDVRTPEEFSQGHACGAINVPYMNRGASGMSKNPDFLEQVSSHFGQSDNIIVGCQSGGRSIKATTDLLHAGFTGVKDIVGGYSAWAKNGLPTKA

>sp|Q556Y8|DUSPR_DICDI Probable rhodanese domain-containing dual specificity protein phosphatase OS=Dictyostelium discoideum OX=44689 GN=DDB_G0273199 PE=3 SV=1

MVLINRVNIQPEELPLLYHVNSMDVYNLLQDIGSSKIIIDLRTKEQYEKNHIRTSVNIPPPPSTTPLYENGEIKEFNLSKYIGSNVTAKHWNLIFQKLIVYSDKPFLYNIDELEKTISTTTITTTATTTTTTTTTSNSIGSDQDIIKSLKVSDWDKVVLRHFLLKKKKTKVIIYQGGFDQFQKDYSFMCNPSSSPSSSSGGGGGSQLYPSEIIKDFLYLGGAENAGNRQQLINLKITHLVNMAGELDDVYPHLYKYYRANLDDRPKANIYEHFEPVIQFINDCKKQGGRVLIHCAMGISRSTTVVLAYLMKEDHMTYSDAFTFCKQKRSCINPNFGFVKQLKDYQQHLTLEWEKQEKLKKQQQQTLNINNNNTGIPLSKKLQLDVSDPLSNSSPSSPLISSTLPIPETPPAIILKNEVASPCPIKTTTSSTTINNKGQQQDKAQEEKDSIFSYADKQEKMTHPTLHSPIELPQSSL

>sp|D3RPB9|RHODA_ALLVD Sulfurtransferase Alvin_2599 OS=Allochromatium vinosum (strain ATCC 17899 / DSM 180 / NBRC 103801 / NCIMB 10441 / D) OX=572477 GN=rhd_2599 PE=1 SV=1

MVNEIDSESLSQRLADTEDVLLVDIRTPAEIAQGMIPDALQLPMHLIPIRMSEIPKDRDVVIYCRSGARSYQACAYLMQQGYGRVLNLRGGIIAWARHGLPIVAPEG

>sp|P25324|THTR_CHICK Thiosulfate sulfurtransferase OS=Gallus gallus OX=9031 GN=TST PE=1 SV=1

AAQALGRALVSAKWLSEAVRAGRVGAGLRVLDASWYPPEERDARQEFKERHIPGASFFNIEECRDKSSPYDFMLPSEAHFADYVGRLGVSNDTHVVVYDGDELGTFYAPRAWWMFRAFGHREVSVLNGGFKNWVKEGHPVTAEPSQPAEAVFKAKLDKTLLKTFEQAMENVGSKKFQVVDSRPAGRFQGTELDQGLESGHIPGAVNMPFSTFLTESGHEKSIEEIQQMFREKKVDLSKPLTATCRKGVTACHIALAAYLCGKPDVAVYDGSWSEWFHRAPPQYKVTELK

>sp|Q08742|RDL2_YEAST Thiosulfate sulfurtransferase RDL2, mitochondrial OS=Saccharomyces cerevisiae (strain ATCC 204508 / S288c) OX=559292 GN=RDL2 PE=1 SV=1

MFKHSTGILSRTVSARSPTLVLRTFTTKAPKIYTFDQVRNLVEHPNDKKLLVDVREPKEVKDYKMPTTINIPVNSAPGALGLPEKEFHKVFQFAKPPHDKELIFLCAKGVRAKTAEELARSYGYENTGIYPGSITEWLAKGGADVKPKK

>sp|P46635|THTR_CRIGR Thiosulfate sulfurtransferase OS=Cricetulus griseus OX=10029 GN=TST PE=2 SV=2

MVHQVLYRALVSTKWLAESIRSGSLGPGLRVLDASWYSPGTRQARKEYQERHVPGASFFDIEECRDTTSPYEMMLPSEAHFADYVGSLGISNDTHVVVYDGDNLGSFYAPRVWWMFRVFGHRTVSVLNGGFRNWLKEGHPVTSEPSRPEPAVFKATLDRSLLKTYEQVLENLQSKRFQLVDSRAQGRYLGTEPEPDIVGLDSGHIRGSANMPFMNFLTEDGFEKSPEELRAIFQDKKVDLSQPLIATCRKGVTACHIALAAYLCGKPDVAVYDGSWSEWFHQAPPETRVSQGKSGKA

>sp|Q38853|STR15_ARATH Rhodanese-like domain-containing protein 15, chloroplastic OS=Arabidopsis thaliana OX=3702 GN=STR15 PE=2 SV=1

METTAFNTTSRIGNWSSAISPPLQTCGSFKCQLPTRRGVIVADLRNSNFRWRKATTTSRGNVAAEAVKIPTSVPVRVARELAQAGYRYLDVRTPDEFSIGHPTRAINVPYMYRVGSGMVKNPSFLRQVSSHFRKHDEIIIGCESGQMSFMASTDLLTAGFTAITDIAGGYVAWTENELPVEE

>sp|Q72JV2|TTUD_THET2 Sulfur carrier protein TtuD OS=Thermus thermophilus (strain ATCC BAA-163 / DSM 7039 / HB27) OX=262724 GN=ttuD PE=1 SV=1

MGYAHPEVLVSTDWVQEHLEDPKVRVLEVDEDILLYDTGHIPGAQKIDWQRDFWDPVVRDFISEEEFAKLMERLGISNDTTVVLYGDKNNWWAAYAFWFFKYNGHKDVRLMNGGRQKWVEEGRPLTTEVPSYPPGRYEVPYRDESIRAYRDDVLEHIIKVKEGKGALVDVRSPQEYRGELTHMPDYPQEGALRAGHIPGAKNIPWAKAVNPDGTFKSAEELRALYEPLGITKDKDIVVYCRIAERSSHSWFVLKYLLGYPHVKNYDGSWTEWGNLVGVPIAKGEE

>sp|P27477|THTR_SYNE7 Putative thiosulfate sulfurtransferase OS=Synechococcus elongatus (strain PCC 7942 / FACHB-805) OX=1140 GN=rhdA PE=1 SV=1

MSVRSLRWPRQKAFLAVISLVVAVLLAVPGWLTPATAASQATVQFVAPTWAAERLNNKQLKILDVRTNPLAYIEGHLPGAVNIADAAYRGPNGFLPVQIWDPEKLASLFGRAGVSNNDTVLVYSDGNDVLGATLVAYLLERSGVQNIAVLDGGYKGYKDAGLPVTKEYPRYQAARFAPKDNRAFRVDIKQVEQLTGKSTFVDPRPPALFSGEQQVFIRNGHIPGARNIPWPTFTEANNANESLKNPHKLKPLSELKAILEAKGVTPDKDVIVTCSTGREASLQYLVLKHLLKYPKVRIYEGSWTEYSASNLPVETGPDRV

>sp|P52197|THTR_AZOVI Thiosulfate sulfurtransferase OS=Azotobacter vinelandii OX=354 GN=rhdA PE=1 SV=1

MDDFASLPLVIEPADLQARLSAPELILVDLTSAARYAEGHIPGARFVDPKRTQLGQPPAPGLQPPREQLESLFGELGHRPEAVYVVYDDEGGGWAGRFIWLLDVIGQQRYHYLNGGLTAWLAEDRPLSRELPAPAGGPVALSLHDEPTASRDYLLGRLGAADLAIWDARSPQEYRGEKVLAAKGGHIPGAVNFEWTAAMDPSRALRIRTDIAGRLEELGITPDKEIVTHCQTHHRSGLTYLIAKALGYPRVKGYAGSWGEWGNHPDTPVEL

>sp|Q94A65|STR14_ARATH Rhodanese-like domain-containing protein 14, chloroplastic OS=Arabidopsis thaliana OX=3702 GN=At4g27700 PE=2 SV=1

MASLTSIATPYPSSSQALRLKSSGNTLFSAGVRSAAMVSGHKTLKIQCTSTKPAKPAAEVDWRQKRELLLEKRVRSVDVKEAQRLQKENNFVILDVRPEAEYKAGHPPGAINVEMYRLIREWTAWDIARRLGFAFFGIFSGTEENPEFIQSVEAKLDKEAKIIVACSSAGTMKPTQNLPEGQQSRSLIAAYLLVLNGYKNVFHLEGGIYTWGKEGLPVETIEED

>sp|O48529|STR9_ARATH Rhodanese-like domain-containing protein 9, chloroplastic OS=Arabidopsis thaliana OX=3702 GN=STR9 PE=2 SV=1

MAGIISPSPTALYFTSNVGGRRLKAVSWAGKSVSGNVIRRRSLRIAAELKFVNAEEAKQLIAEEGYSVVDVRDKTQFERAHIKSCSHIPLFIYNEDNDIGTIIKRTVHNNFSGLFFGLPFTKVNPEFLKSVRNEFSQDSKLLLVCQEGLRSAAAASRLEEAGYENIACVTSGLQSVKPGTFESVGSTELQNAGKAGLITIQGKISAVLGTVLVCAYLFIQFFPDQAEKLFPPTS

>sp|P9WHF9|THTR_MYCTU Putative thiosulfate sulfurtransferase OS=Mycobacterium tuberculosis (strain ATCC 25618 / H37Rv) OX=83332 GN=cysA1 PE=1 SV=1

MARCDVLVSADWAESNLHAPKVVFVEVDEDTSAYDRDHIAGAIKLDWRTDLQDPVKRDFVDAQQFSKLLSERGIANEDTVILYGGNNNWFAAYAYWYFKLYGHEKVKLLDGGRKKWELDGRPLSSDPVSRPVTSYTASPPDNTIRAFRDEVLAAINVKNLIDVRSPDEFSGKILAPAHLPQEQSQRPGHIPGAINVPWSRAANEDGTFKSDEELAKLYADAGLDNSKETIAYCRIGERSSHTWFVLRELLGHQNVKNYDGSWTEYGSLVGAPIELGS

>sp|P58388|THTM_ECO57 3-mercaptopyruvate sulfurtransferase OS=Escherichia coli O157:H7 OX=83334 GN=sseA PE=3 SV=2

MSTTWFVGADWLAEHIDDPEIQIIDARMASPGQEDRNVAQEYLNGHIPGAVFFDIEALSDHTSPLPHMLPRPETFAVAMRELGVNQDKHLIVYDEGNLFSAPRAWWMLRTFGVEKVSILGGGLAGWQRDDLLLEEGAVELPEGEFNAAFNPEAVVKVTDVLLASHENTAQIIDARPAARFNAEVDEPRPGLRRGHIPGALNVPWTELVREGELKTTDELDAIFFGRGVSYDKPIIVSCGSGVTAAVVLLALATLDVPNVKLYDGAWSEWGARADLPVEPLK

>sp|Q93WI0|STR12_ARATH Rhodanese-like/PpiC domain-containing protein 12, chloroplastic OS=Arabidopsis thaliana OX=3702 GN=At5g19370 PE=1 SV=1

MFRVTGTLSAASSPAVAAASFSAALRLSITPTLAIASPPHLRWFSKFSRQFLGGRISSLRPRIPSPCPIRLSGFPALKMRASFSSGSSGSSASREILVQHLLVKNNDVELFAELQKKFLDGEEMSDLAAEYSICPSKKDGGILGWVKLGQMVPEFEEAAFKAELNQVVRCRTQFGLHLLQVLSEREPVKDIQVEELHSKMQDPVFMDEAQLIDVREPNEIEIASLPGFKVFPLRQFGTWAPDITSKLNPEKDTFVLCKVGGRSMQVANWLQSQGFKSVYNITGGIQAYSLKVDPSIPTY

>sp|Q10215|RDL_SCHPO Putative thiosulfate sulfurtransferase, mitochondrial OS=Schizosaccharomyces pombe (strain 972 / ATCC 24843) OX=284812 GN=SPAC4H3.07c PE=3 SV=2

MFSKTLSVSRLLMTRSFYSSTVVKNVSIFDFEKVYNLSKRPTGDKSTVLIDVREPDEFKQGAIETSYNLPVGKIEEAMKLSDEEFSKTYGFSKPVFEDNVVVYCRSGRRSTTASDILTKLGYKNIGNYTGSWLEWSDKIKSK

>sp|D4GYM0|THTR_HALVD Putative thiosulfate sulfurtransferase OS=Haloferax volcanii (strain ATCC 29605 / DSM 3757 / JCM 8879 / NBRC 14742 / NCIMB 2012 / VKM B-1768 / DS2) OX=309800 GN=tssA PE=1 SV=1

MSNSDYAKDVLVSADWVESHLDEFQSDDPAYRLVEVDVDTEAYDESHAPGAIGFNWESQLQDQTTRDVLTKEDFEDLLGSHGISEDSTVVLYGDNSNWFAAYTYWQFKYYGHENVHLMNGGRDYWVDNDYPTTDEIPSFPEQDYSAKGPFEDIRAYRDDVEKAVDKGLPLVDVRSPEEFSGEILAPPGLQETAQRGGHIPGASNISWAATVNDDGTFKSADELRDLYADQGIEGDESTIAYCRIGERSSIAWFALHELLGYENVTNYDGSWTEWGNLVGAPVEKGN

>sp|P59989|THTR1_MYCBO Putative thiosulfate sulfurtransferase 1 OS=Mycobacterium bovis (strain ATCC BAA-935 / AF2122/97) OX=233413 GN=cysA1 PE=3 SV=1

MARCDVLVSADWAESNLHAPKVVFVEVDEDTSAYDRDHIAGAIKLDWRTDLQDPVKRDFVDAQQFSKLLSERGIANEDTVILYGGNNNWFAAYAYWYFKLYGHEKVKLLDGGRKKWELDGRPLSSDPVSRPVTSYTASPPDNTIRAFRDEVLAAINVKNLIDVRSPDEFSGKILAPAHLPQEQSQRPGHIPGAINVPWSRAANEDGTFKSDEELAKLYADAGLDNSKETIAYCRIGERSSHTWFVLRELLGHQNVKNYDGSWTEYGSLVGAPIELGS

>sp|Q9SR92|STR10_ARATH Rhodanese-like domain-containing protein 10 OS=Arabidopsis thaliana OX=3702 GN=STR10 PE=2 SV=1

MTVLLPQLNHIHKLPVVLNRRLRQSYRLPVISAVSGKELILSGKVRAVEPKEANAVVASEGYILLDVRPAWEREKARVKGSLHVPLFVEDPDNGPITLLKKWIHLGYIGLWTGQRFTMINDEFALRVVEAVPDKESKVLVVCGEGLRSLAAVSKLHGEGYKSLGWLTGGFNRVSEGDFPEIEGTEELRFATIGGVSFYLLKLLVLLPSFGQKSR

>sp|Q3U269|TSTD2_MOUSE Thiosulfate sulfurtransferase/rhodanese-like domain-containing protein 2 OS=Mus musculus OX=10090 GN=Tstd2 PE=2 SV=3

MPSSTSPDEEDGLETCVLKVFDLDLKESNLVNPSNSLKAELDGSTKKKYSFAKKKAFALLVKTKQVPAPSYEFKGKRWRCCQQLFADQISIHRHVATQHAEDVYQQTASLLKQLTAALSASQSLTPTDKRSSPKDCLTPSQEVSAWLPDVSHVSPQELRSGQGDEEGEVLLYYCYCDLEDPHWVCAWQTALCHHLHLTGKIRIATEGINGTVGGSKVATRLYVEVMLSCPLFKDYLSEDDFKSSKGGSHCFPELRVGVFEEIVPMGISPSQVSYKKPGIHLSPGEFHKEIEKLLSQSSEEQGNTIILDCRNFYESKIGRFQGCLAPDIRKFSYFPSYVDKNLDIFRQKRVLMYCTGGIRCERGSAYLRAKGVCKEVFQLKGGIHKYLEEFPDGFYKGKLFVFDERFALAYNSSVVSECSYCGAPWDQYKLCSTPQCRQLVLTCSACQGQGFTACCVTCQDKGGKQASGPSQDSFKEECECTARRPRIPQEQQAQS

>sp|Q9RXT9|THTR_DEIRA Thiosulfate sulfurtransferase OS=Deinococcus radiodurans (strain ATCC 13939 / DSM 20539 / JCM 16871 / LMG 4051 / NBRC 15346 / NCIMB 9279 / R1 / VKM B-1422) OX=243230 GN=DR_0217 PE=1 SV=1

MDYAKDVLVSTEWAAQNLQTPGVRFIEVDEDILLYETGHLPGAVKLDWQTDLWHPVERDFIEPQQVSELLGKLGIKADDTIVLYGDKSNWWASYAYWFLTYSGVSNLKIMNGGRQKWVAEGREMTTEAPTVTATTYPALQRDESLRAYRDEVRAHLESVNNGQGAMVDVRSPDEFSGKVTHMPNYPQEGVLRGGHIPGARNIPWAKATNEDGTFKSADELKALYEGEGVTADKDVIAYCRIAERSSHSWFVLRELLGYPKVRNYDGSWTEWGNGVGLPIEKTYSEE

>sp|F4I933|STR8_ARATH Rhodanese-like domain-containing protein 8, chloroplastic OS=Arabidopsis thaliana OX=3702 GN=STR8 PE=4 SV=1

MRVSPAATLSVSLTTPLPITLTKARFSGEPLRLKQYVLSNQICSRPKHLLSEFKPTRRWTFSCKCRNRGRNGYAKFDDEGEDFIVVNFYRFVSIGDPEAEIEKHLSFLKDLNIRGRIYLNEQGINAQYSGPSKDALAYVEWLKGDDRFSDLLVQMSPAMNRHAFPKLKLQNKPSLVQYEGGISHLPLLDPPMRAKPLEPSEWKRKLKDLTDDDEASPSNSGKSYILLDVRNGYEWDVGHFRGAHRPEVDCFRNTSFGLSDEKEAPSDPLINVDKEKTDILMYCTGGIRCDVYSTVLRQRGFKNLYTLKGGVSHYLKEEGTAEWVGNLFVFDSRLSLPPAAYNDNVVDKAVGDNVVDEAGRTPQTPVDTSFARCYLCNSQVQELRHRNCANLDCNRLFLCCAECVVDLKGCCCSDCISAPRLRPVLHGVKRYEKWHVYRDSEEQNAPLV

>sp|Q94AC1|STR6_ARATH Rhodanese-like domain-containing protein 6 OS=Arabidopsis thaliana OX=3702 GN=STR6 PE=2 SV=1

MGTSSCGDHEKQRIEDEEQYGVLLYYKYTSVPDLDELVSFYESSCNSLGLLGRVRLSPKGVNVTVGGKLTALEEHIAAAKSNCLFEGTDFKLASCHHPLNDKVAEECGFTSLSIRVVEELVTFSPCPPLKPPEISNAGKHLSAAEFHSVLQSANGKSENKELVLLDARNLYETRIGKFESENVETLDPEIRQYSDLPTWIDQNAEKMKGKNVLMYCTGGIRCEMASAYIRSKGAGFENTFQLYGGIQRYLEQFPSGGFFKGKNFVFDHRISVGSSKEDIIGSCLLCNNTFDDYSPRCRCRLCRMLVLVCNHCRVKGDIYICELCRKHGKGEVPLSLDPLNQPSESNGDNTRRKLRILCLHGFRQNASSFKGRTGSLAKKLKNIAELVFIDAPHELQFIYQTATPPSGVCNKKFAWLVSSDFDKPSETGWTVAQCQFDPLQYQTQTEGFDKSLTYLKTAFEEKGPFDGILGFSQGAAMAAAVCGKQEQLVGEIDFRFCVLCSGFTPWPLLEMKEKRSIKCPSLHIFGSQPGKDRQIVTQASSDLAGLFEDGCATIVEHDFGHIIPTKSPYIDEIKAFLYQFI

>sp|F4IPI4|STR17_ARATH Rhodanese-like domain-containing protein 17 OS=Arabidopsis thaliana OX=3702 GN=STR17 PE=2 SV=1

MDSLHVLRSFLLLFIVFNHLPRTTTSMSEPKVITIDVNQAQKLLDSGYTFLDVRTVEEFKKGHVDSENVFNVPYWLYTPQGQEINPNFLKHVSSLCNQTDHLILGCKSGVRSLHATKFLVSSGFKTVRNMDGGYIAWVNKRFPVKVEHKELKYDEL

>sp|Q9I452|THTM_PSEAE Probable 3-mercaptopyruvate sulfurtransferase OS=Pseudomonas aeruginosa (strain ATCC 15692 / DSM 22644 / CIP 104116 / JCM 14847 / LMG 12228 / 1C / PRS 101 / PAO1) OX=208964 GN=sseA PE=3 SV=1

MSSAQLLTAQQLAARLSEPDLLVLDCRFALEDPSYGARVYQENHIPGAHFADLERDLSAPVRKGVTGRHPLPDPAELALKLQAWGLRQDSQVVLYDDGPGAFAARAWWLLHWLGKRDGVYLLDGGLAAWKAAGLALTNGESSLRPGDFQGQPDASLLIDAATLQAQLGQPGLALLDARAQPRFRGEVEPIDPVAGHIPGAQCAAFTDNLGSDGRFLPPEQLHQRFSALLRGRPVDELVAYCGSGVTACHNLFALSLAGFPLPRLYAGSWSEWITDPRRPVATGD

>sp|Q5RCP1|TSTD2_PONAB Thiosulfate sulfurtransferase/rhodanese-like domain-containing protein 2 OS=Pongo abelii OX=9601 GN=TSTD2 PE=2 SV=1

MPSSTSPDQGDDLETCILRFSDLDLKDMSLINPSSSLKAELDGSTKKKYSFAKKKAFALFVKTKEVPTKRSFECKEKLWKCCQQLFTDQTSIHRHVATQHADEIYHQTASILKQLAVTLSTSKSLSSADEKNPLKECLPHSHDVSAWLPDISCFNPDELISGQGSEEGEVLLYYCYRDLEDPQWICAWQTALCQQLHLTGKIRIAAEGINGTVGGSKLATRLYVEVMLSFPLFKDDLCKDDFKTSKGGAHCFPELRVGVFEEIVPMGISPKKISYKKPGIHLSPGEFHKEVEKFLSQANQEQSDTILLDCRNFYESKIGRFQGCLAPDIRKFSYFPSYVDKNLELFREKRVLMYCTGGIRCERGSAYLKAKGVCKEVFQLKGGIHKYLEEFPDGFYKGKLFVFDERYALSYNSDVVSECSYCGARWDQYKLCSTPQCHQLVLTCPACQGQGFTACCVTCQDKGSRKVSGPMQDSFKEECECTARRPRIPRELLQHVRQPVSPEPGPDAEEDGPVLV

>sp|Q0WWT7|STR11_ARATH Rhodanese-like domain-containing protein 11, chloroplastic OS=Arabidopsis thaliana OX=3702 GN=STR11 PE=2 SV=1

MESLSLPVLNPLLASGSNLFRNQHSRMTSSMVSSLKSPIGGTSLSTVRRFGVGVVRMQAVDEDIDLKQMRDIAAAKKRWDGLLREGKVKLLTPREAGYAISLSNKPLLDVRPSSERNKAWIKGSTWVPIFDNDDNLDAGTLSKKVTSFAMGGWWSGAPTLSFNRLFLSKVEEKFPKDSELIVACQKGLRSLAACELLYNAGYENLFWVQGGLESAQDEDLVTEGVQPLKLAGIGGFSEFLGWTDQQRAQAAKEGWGYRLVYTARLFGVVLAADALFVGAQQLGHYIQELRGH

>sp|Q1JPN0|STR7_ARATH Rhodanese-like domain-containing protein 7 OS=Arabidopsis thaliana OX=3702 GN=STR7 PE=2 SV=1

MLRFYHWRFPPSLAVVRMLSSPPPPHSHSPFSGGGVNSNSVGGNSKPELQFPQSQPHKLSSSPSSSLKSTVACSNAGAIRRSMATVSQAFSERTESIDSDLGSLVVVSFYKFADFPEHADFRKPLKDLCEKLRVSGGIILAPEGINGSICGIRESVEEVLAFIQRDVRLNGLRQVETPVSPEQEAIHHGHSSSSPLAAGEDAPFRWDHVRVKLKKEIVTLGIPSVSPIERVGTYVSPEEWNELISDPETVVIDVRNTYETRIGKFKGAVDPCTTAFRNFPSWVENQFALKQEGNETQAKVEKEDFSEITHKEDKAEKPKTLPRIAMYCTGGIRCEKASSLLLSQGFEEVYHLKGGILKYLEEVPKTESLWEGECFVFDKRVSVEHGLAQGTHKLCYGCKQPISDEDMEAPEYEYGVSCPYCYSKKSEEEKERARARQTQFEEWGVIGGPDKGRRPATKPDSPRKKINAKLGSSI

>sp|Q56XR7|STR4A_ARATH Rhodanese-like domain-containing protein 4A, chloroplastic OS=Arabidopsis thaliana OX=3702 GN=STR4A PE=2 SV=1

MTSLPIILASSPLRNLTKPCSTSQIPKPIQNSTKQPPIHLLTKTNLSVTISQLIITSPVL

ASESFDSISSDPSSGKIDLESILVTIDNFFNKYPFFVAGCTFTYLVVYPAVMFYLRKYKPISAMNAFRKLKNESDSQLLDIRDVKTLALLASPNLKFLGKSSVQVPFSENDEEGFLTKVKGSFSDAENTVVCVLDNFDGNSSKVAELLIKNGFKEAYYIRGGARGKNGWLAIQEELLPPPVHMYTAKNVKSSNNNEASVVGTEN

>sp|P22978|CG445_DROME Rhodanese domain-containing protein CG4456 OS=Drosophila melanogaster OX=7227 GN=CG4456 PE=2 SV=1

MATYEQVKDVPNHPDVYLIDVRRKEELQQTGFIPASINIPLDELDKALNLDGSAFKNKYGRSKPEKQSPIIFTCRSGNRVLEAEKIAKSQGYSNVVIYKGSWNEWAQKEGL

>sp|Q50036|THTR_MYCLE Putative thiosulfate sulfurtransferase OS=Mycobacterium leprae (strain TN) OX=272631 GN=cysA PE=3 SV=1

MARSDVLVSADWAESNLDSANIVFVEVDEDTSTYDGDHIAGAIKLDWRADLQDPIKRDFIDTQQFSKLLGDRGISNDNTVILYGGNNNWFAAYAYWYFKLYRHDKVKLLDGGRKKWELDGRPLSTDTVTRPATSYAAAAPDNTIRAFRDEVIASIKIKNLVDVRSPDEFSGKLLAPAHLPQEQSQRPGHIPSAINIPWSKAANEDGTFKSDEQLAKLYADAGLDRLKETIVYCRIGERSSHTWFVLRELLGYQNVKNYDGSWTEYGSLVGVPIELGS

>sp|P16385|THTR_SACER Putative thiosulfate sulfurtransferase OS=Saccharopolyspora erythraea OX=1836 GN=cysA PE=1 SV=1

MSREEVLVSTDWAEQNLNTDGVVFAEVDEDTTAYDGGHIPGAIKLDWKNELQDHVRRDFVNREGFEKLLSAKGIGNDDTVILYGGNNNWFAAYAYWYFKLYGHSDVKLLDGGRKKWELDGRELTKEEPNRAATAYKAQEPDASIRAFRDEVVDAIGNKNLVDVRSPDEFAGKLLAPAHLPQESAQRAGHIPSAINVPWSKAANEDGTFKSDEELKQVYGEAGLDTDKDTIAYCRIGERSSHTWFVLRELLGHTNVKNYDGSWTEYGSLVGVPIENPQEQGA

>sp|Q7TX80|THTR2_MYCBO Putative thiosulfate sulfurtransferase 2 OS=Mycobacterium bovis (strain ATCC BAA-935 / AF2122/97) OX=233413 GN=cysA2 PE=3 SV=1

MARCDVLVSADWAESNLHAPKVVFVEVDEDTSAYDRDHIAGAIKLDWRTDLQDPVKRDFVDAQQFSKLLSERGIANEDTVILYGGNNNWFAAYAYWYFKLYGHEKVKLLDGGRKKWELDGRPLSSDPVSRPVTSYTASPPDNTIRAFRDEVLAAINVKNLIDVRSPDEFSGKILAPAHLPQEQSQRPGHIPGAINVPWSRAANEDGTFKSDEELAKLYADAGLDNSKETIAYCRIGERSSHTWFVLRELLGHQNVNIAFGYGPHACPASAYSRMCLTTFFTSLTQRFPQLQLARPFEDLERRGKGLHSVGIKELLVTWPT

>sp|Q9D0B5|TSTD3_MOUSE Thiosulfate sulfurtransferase/rhodanese-like domain-containing protein 3 OS=Mus musculus OX=10090 GN=Tstd3 PE=1 SV=1

MLARLVLGTSGRAALGSVEPALGGLKSIWRCSQAFCSTPKGVTYRELKSLLNSKDIMLIDVRNTLEILEQGKIPGSINIPLDEVGEALQMNPVDFKEKYCQVKPSKSDRLVFSCLAGVRSKKAMDTAISLGFNSAQHYAGGWKEWVTYEISEEKQES

>sp|A2X345|TROL_ORYSI Protein THYLAKOID RHODANESE-LIKE, chloroplastic OS=Oryza sativa subsp. indica OX=39946 GN=TROL PE=3 SV=1

MAATTTILSSAAPTPLTAPPRARARAPAARRRRLRARDILGAALGLANGGASAALAAPLSYEETLRLSTDSGGGGGGGGEFALPDLGLGGVLDFVAQNPLVAAAGVAAVALPLVLAQVLGGASKPYGVVSAAAAYRALVEEPGAQLVDIRPPGDARQSGAPDLREAKKKAAAVPYDGEDKNGFLKKLSLRFKDPENTTLVILDKFDGNSELVAELVTANGYKAAFAVKDGAEGRRGWLSSSLPWTAPKKGFSLSDLIGDGTDGLPVTLGLAAATGLGILAYTEIETVLQFLGSAAIVQLVASKLIYAEDRKRTLKQIDDFFNKKVAPKELVDEIKEISQALLPSTGTKSQPAITEAAPATAEAAPAAATATAAPPAAPVEETSTEAAPAEPTPLSPYTNYPDLKPPSSPSPLAPAEATKNESESESAATESAPAVNSAPVAEAAPEAAPPAAPRPLSPYPNYPDLKPPSSPSPSAP

>sp|Q6ETQ7|TROL_ORYSJ Protein THYLAKOID RHODANESE-LIKE, chloroplastic OS=Oryza sativa subsp. japonica OX=39947 GN=TROL PE=1 SV=1

MAATTTILSSAAPTPLTAPPRARARAPAARRRRLRARDILGAALGLANGGASAALAAPLSYEETLRLSTDSGGGGGGGGGGEFALPDLGLGGVLDFVAQNPLAAAAGVAAVALPLVLAQVLGGASKPYGVVSAAAAYRALVEEPGAQLVDIRPPGDARQSGAPDLREAKKKAAAVPYDGEDKNGFLKKLSLRFKDPENTTLVILDKFDGNSELVAELVTANGYKAAFAVKDGAEGRRGWLSSSLPWTAPKKGFSLSDLIGDGTDGLPVTLGLAAATGLGILAYTEIETVLQFLGSAAIVQLVASKLIYAEDRKRTLKQIDDFFNKKVAPKELVDEIKEIGQALLPSTGTKSQPAITEAAPATAEAAPAAATATAAPPAAPVEETSTEAAPAEPTPLSPYTNYPDLKPPSSPSPLAPAEATKNESESESAATESAPAVNSAPVAEAAPEAAPPAAPRPLSPYPNYPDLKPPSSPSPSAP

>sp|H0UI37|TSTD3_HUMAN Thiosulfate sulfurtransferase/rhodanese-like domain-containing protein 3 OS=Homo sapiens OX=9606 GN=TSTD3 PE=3 SV=1MKIEKCGWSEGLTSIKGNCHNFYTAISKDVTYKELKNLLNSKNIMLIDVREIWEILEYQKIPESINVPLDEVGEALQMNPRDFKEKYNEVKPSKSDS

>sp|A0R4C9|THTR_MYCS2 Putative thiosulfate sulfurtransferase OS=Mycolicibacterium smegmatis (strain ATCC 700084 / mc(2)155) OX=246196 GN=MSMEG_5789 PE=1 SV=1

MARSDVLVSTDWAESNLKAPKTVFVEVDEDTSAYDTGHIEGAVKLDWKTDLQDPIRRDFVDAQQFSKLLSERGIANDDTVILYGGNNNWFAAYAYWYFKLYGHQDVKLLDGGRKKWELDARPLSAEKVERPQTSYTAKEPDNSIRAFRDEVIAAIGTKNLVDVRSPDEFSGKILAPAHLPQEQSQRPGHIPGAINVPWSKAANEDGTFKSDEELAKLYAEAGLDGEKETIAYCRIGERSSHTWFVLQELLGHKNVKNYDGSWTEYGSLVGAPIELGS

>sp|P9WHF8|THTR_MYCTO Putative thiosulfate sulfurtransferase OS=Mycobacterium tuberculosis (strain CDC 1551 / Oshkosh) OX=83331 GN=cysA1 PE=3 SV=1

MARCDVLVSADWAESNLHAPKVVFVEVDEDTSAYDRDHIAGAIKLDWRTDLQDPVKRDFVDAQQFSKLLSERGIANEDTVILYGGNNNWFAAYAYWYFKLYGHEKVKLLDGGRKKWELDGRPLSSDPVSRPVTSYTASPPDNTIRAFRDEVLAAINVKNLIDVRSPDEFSGKILAPAHLPQEQSQRPGHIPGAINVPWSRAANEDGTFKSDEELAKLYADAGLDNSKETIAYCRIGERSSHTWFVLRELLGHQNVKNYDGSWTEYGSLVGAPIELGS

Supplemental file 2:

Human Rhd containing protein identified into the UniProt database.

| Entry | Entry name | Protein names | Gene names | Length | Domain [FT] 1 | Domaine name | ProSite accession | Domain [FT] 2 | Domaine name | ProSite accession |
| --- | --- | --- | --- | --- | --- | --- | --- | --- | --- | --- |
| P30304 | MPIP1_HUMAN | M-phase inducer phosphatase 1 (EC 3.1.3.48) (Dual specificity phosphatase Cdc25A) | CDC25A | 524 | DOMAIN 376..482 | /note="Rhodanese" | /evidence="ECO:0000255\|PROSITE-ProRule:PRU00173" |  |  |  |
| P30305 | MPIP2_HUMAN | M-phase inducer phosphatase 2 (EC 3.1.3.48) (Dual specificity phosphatase Cdc25B) | CDC25B CDC25HU2 | 580 | DOMAIN 431..538 | /note="Rhodanese" | /evidence="ECO:0000255\|PROSITE-ProRule:PRU00173" |  |  |  |
| P30307 | MPIP3_HUMAN | M-phase inducer phosphatase 3 (EC 3.1.3.48) (Dual specificity phosphatase Cdc25C) | CDC25C | 473 | DOMAIN 321..428 | /note="Rhodanese" | /evidence="ECO:0000255\|PROSITE-ProRule:PRU00173" |  |  |  |
| Q9BYV8 | CEP41_HUMAN | Centrosomal protein of 41 kDa (Cep41) (Testis-specific gene A14 protein) | CEP41 TSGA14 | 373 | DOMAIN 169..266 | /note="Rhodanese" | /evidence="ECO:0000255\|PROSITE-ProRule:PRU00173" |  |  |  |
| P28562 | DUS1_HUMAN | Dual specificity protein phosphatase 1 (EC 3.1.3.16) (EC 3.1.3.48) (Dual specificity protein phosphatase hVH1) (Mitogen-activated protein kinase phosphatase 1) (MAP kinase phosphatase 1) (MKP-1) (Protein-tyrosine phosphatase CL100) | DUSP1 CL100 MKP1 PTPN10 VH1 | 367 | DOMAIN 20..137 | /note="Rhodanese" | /evidence="ECO:0000255\|PROSITE-ProRule:PRU00173" | DOMAIN 173..314 | /note="Tyrosine-protein phosphatase" | /evidence="ECO:0000255\|PROSITE-ProRule:PRU00160" |
| Q9Y6W6 | DUS10_HUMAN | Dual specificity protein phosphatase 10 (EC 3.1.3.16) (EC 3.1.3.48) (Mitogen-activated protein kinase phosphatase 5) (MAP kinase phosphatase 5) (MKP-5) | DUSP10 MKP5 | 482 | DOMAIN 168..285 | /note="Rhodanese" | /evidence="ECO:0000255\|PROSITE-ProRule:PRU00173" | DOMAIN 321..464 | /note="Tyrosine-protein phosphatase" | /evidence="ECO:0000255\|PROSITE-ProRule:PRU00160" |
| Q9BY84 | DUS16_HUMAN | Dual specificity protein phosphatase 16 (EC 3.1.3.16) (EC 3.1.3.48) (Mitogen-activated protein kinase phosphatase 7) (MAP kinase phosphatase 7) (MKP-7) | DUSP16 KIAA1700 MKP7 | 665 | DOMAIN 22..137 | /note="Rhodanese" | /evidence="ECO:0000255\|PROSITE-ProRule:PRU00173" | DOMAIN 158..300 | /note="Tyrosine-protein phosphatase" | /evidence="ECO:0000255\|PROSITE-ProRule:PRU00160" |
| Q05923 | DUS2_HUMAN | Dual specificity protein phosphatase 2 (EC 3.1.3.16) (EC 3.1.3.48) (Dual specificity protein phosphatase PAC-1) | DUSP2 PAC1 | 314 | DOMAIN 23..144 | /note="Rhodanese" | /evidence="ECO:0000255\|PROSITE-ProRule:PRU00173" | DOMAIN 172..313 | /note="Tyrosine-protein phosphatase" | /evidence="ECO:0000255\|PROSITE-ProRule:PRU00160" |
| Q13115 | DUS4_HUMAN | Dual specificity protein phosphatase 4 (EC 3.1.3.16) (EC 3.1.3.48) (Dual specificity protein phosphatase hVH2) (Mitogen-activated protein kinase phosphatase 2) (MAP kinase phosphatase 2) (MKP-2) | DUSP4 MKP2 VH2 | 394 | DOMAIN 41..159 | /note="Rhodanese" | /evidence="ECO:0000255\|PROSITE-ProRule:PRU00173" | DOMAIN 195..336 | /note="Tyrosine-protein phosphatase" | /evidence="ECO:0000255\|PROSITE-ProRule:PRU00160" |
| Q16690 | DUS5_HUMAN | Dual specificity protein phosphatase 5 (EC 3.1.3.16) (EC 3.1.3.48) (Dual specificity protein phosphatase hVH3) | DUSP5 VH3 | 384 | DOMAIN 19..141 | /note="Rhodanese" | /evidence="ECO:0000255\|PROSITE-ProRule:PRU00173" | DOMAIN 178..319 | /note="Tyrosine-protein phosphatase" | /evidence="ECO:0000255\|PROSITE-ProRule:PRU00160" |
| Q16828 | DUS6_HUMAN | Dual specificity protein phosphatase 6 (EC 3.1.3.16) (EC 3.1.3.48) (Dual specificity protein phosphatase PYST1) (Mitogen-activated protein kinase phosphatase 3) (MAP kinase phosphatase 3) (MKP-3) | DUSP6 MKP3 PYST1 | 381 | DOMAIN 30..148 | /note="Rhodanese" | /evidence="ECO:0000255\|PROSITE-ProRule:PRU00173" | DOMAIN 206..349 | /note="Tyrosine-protein phosphatase" | /evidence="ECO:0000255\|PROSITE-ProRule:PRU00160" |
| Q16829 | DUS7_HUMAN | Dual specificity protein phosphatase 7 (EC 3.1.3.16) (EC 3.1.3.48) (Dual specificity protein phosphatase PYST2) | DUSP7 PYST2 | 419 | DOMAIN 68..187 | /note="Rhodanese" | /evidence="ECO:0000255\|PROSITE-ProRule:PRU00173" | DOMAIN 244..387 | /note="Tyrosine-protein phosphatase" | /evidence="ECO:0000255\|PROSITE-ProRule:PRU00160" |
| Q13202 | DUS8_HUMAN | Dual specificity protein phosphatase 8 (EC 3.1.3.16) (EC 3.1.3.48) (Dual specificity protein phosphatase hVH-5) | DUSP8 C11orf81 VH5 | 625 | DOMAIN 23..138 | /note="Rhodanese" | /evidence="ECO:0000255\|PROSITE-ProRule:PRU00173" | DOMAIN 160..302 | /note="Tyrosine-protein phosphatase" | /evidence="ECO:0000255\|PROSITE-ProRule:PRU00160" |
| Q99956 | DUS9_HUMAN | Dual specificity protein phosphatase 9 (EC 3.1.3.16) (EC 3.1.3.48) (Mitogen-activated protein kinase phosphatase 4) (MAP kinase phosphatase 4) (MKP-4) | DUSP9 MKP4 | 384 | DOMAIN 18..139 | /note="Rhodanese" | /evidence="ECO:0000255\|PROSITE-ProRule:PRU00173" | DOMAIN 203..346 | /note="Tyrosine-protein phosphatase" | /evidence="ECO:0000255\|PROSITE-ProRule:PRU00160" |
| O96007 | MOC2B_HUMAN | Molybdopterin synthase catalytic subunit (EC 2.8.1.12) (MOCO1-B) (Molybdenum cofactor synthesis protein 2 large subunit) (Molybdenum cofactor synthesis protein 2B) (MOCS2B) (Molybdopterin-synthase large subunit) (MPT synthase large subunit) | MOCS2 MCBPE MOCO1 | 188 |  |  |  |  |  |  |
| O96033 | MOC2A_HUMAN | Molybdopterin synthase sulfur carrier subunit (MOCO1-A) (Molybdenum cofactor synthesis protein 2 small subunit) (Molybdenum cofactor synthesis protein 2A) (MOCS2A) (Molybdopterin-synthase small subunit) (Sulfur carrier protein MOCS2A) | MOCS2 MOCO1 | 88 |  |  |  |  |  |  |
| O95396 | MOCS3_HUMAN | Adenylyltransferase and sulfurtransferase MOCS3 (Molybdenum cofactor synthesis protein 3) (Molybdopterin synthase sulfurylase) (MPT synthase sulfurylase) [Includes: Molybdopterin-synthase adenylyltransferase (EC 2.7.7.80) (Adenylyltransferase MOCS3) (Sulfur carrier protein MOCS2A adenylyltransferase); Molybdopterin-synthase sulfurtransferase (EC 2.8.1.11) (Sulfur carrier protein MOCS2A sulfurtransferase) (Sulfurtransferase MOCS3)] | MOCS3 UBA4 | 460 | DOMAIN 347..458 | /note="Rhodanese" | /evidence="ECO:0000255\|HAMAP-Rule:MF_03049" |  |  |  |
| P25325 | THTM_HUMAN | 3-mercaptopyruvate sulfurtransferase (MST) (EC 2.8.1.2) | MPST TST2 | 297 | DOMAIN 25..144 | /note="Rhodanese 1" | /evidence="ECO:0000255\|PROSITE-ProRule:PRU00173" | DOMAIN 174..288 | /note="Rhodanese 2" | /evidence="ECO:0000255\|PROSITE-ProRule:PRU00173" |
| Q9H0U6 | RM18_HUMAN | 39S ribosomal protein L18, mitochondrial (L18mt) (MRP-L18) (Mitochondrial large ribosomal subunit protein uL18m) | MRPL18 HSPC071 | 180 |  |  |  |  |  |  |

Human Rhd containing protein identified into the UniProt database (suite).

| Entry | Entry name | Protein names | Gene names | Length | Domain [FT] 1 | Domaine name | ProSite accession | Domain [FT] 2 | Domaine name | ProSite accession |
| --- | --- | --- | --- | --- | --- | --- | --- | --- | --- | --- |
| Q9H4P4 | RNF41_HUMAN | E3 ubiquitin-protein ligase NRDP1 (EC 2.3.2.27) (RING finger protein 41) (RING-type E3 ubiquitin transferase NRDP1) | RNF41 FLRF NRDP1 SBBI03 | 317 |  |  |  |  |  |  |
| Q9Y6J8 | STYL1_HUMAN | Serine/threonine/tyrosine-interacting-like protein 1 (Dual specificity phosphatase inhibitor MK-STYX) (Dual specificity protein phosphatase 24) (Inactive dual specificity protein phosphatase MK-STYX) (Map kinase phosphatase-like protein MK-STYX) | STYXL1 DUSP24 MKSTYX | 313 | DOMAIN 27..138 | /note="Rhodanese" | /evidence="ECO:0000255\|PROSITE-ProRule:PRU00173" | DOMAIN 159..302 | /note="Tyrosine-protein phosphatase" | /evidence="ECO:0000255\|PROSITE-ProRule:PRU00160" |
| Q9NUY8 | TBC23_HUMAN | TBC1 domain family member 23 (HCV non-structural protein 4A-transactivated protein 1) | TBC1D23 NS4ATP1 | 699 | DOMAIN 44..225 | /note="Rab-GAP TBC" | /evidence="ECO:0000255\|PROSITE-ProRule:PRU00163" | DOMAIN 334..446 | /note="Rhodanese" | /evidence="ECO:0000255\|PROSITE-ProRule:PRU00173" |
| Q8TEA7 | TBCK_HUMAN | TBC domain-containing protein kinase-like protein | TBCK TBCKL HSPC302 | 893 | DOMAIN 1..273 | /note="Protein kinase" | /evidence="ECO:0000255\|PROSITE-ProRule:PRU00159" | DOMAIN 466..651 | /note="Rab-GAP TBC" | /evidence="ECO:0000255\|PROSITE-ProRule:PRU00163" |
| Q16762 | THTR_HUMAN | Thiosulfate sulfurtransferase (EC 2.8.1.1) (Rhodanese) | TST | 297 | DOMAIN 25..143 | /note="Rhodanese 1" | /evidence="ECO:0000255\|PROSITE-ProRule:PRU00173" | DOMAIN 173..288 | /note="Rhodanese 2" | /evidence="ECO:0000255\|PROSITE-ProRule:PRU00173" |
| Q8NFU3 | TSTD1_HUMAN | Thiosulfate:glutathione sulfurtransferase (TST) (EC 2.8.1.-) | TSTD1 KAT | 115 | DOMAIN 17..115 | /note="Rhodanese" | /evidence="ECO:0000255\|PROSITE-ProRule:PRU00173" |  |  |  |
| Q5T7W7 | TSTD2_HUMAN | Thiosulfate sulfurtransferase/rhodanese-like domain-containing protein 2 (Rhodanese domain-containing protein 2) | TSTD2 C9orf97 PP4189 | 516 | DOMAIN 301..396 | /note="Rhodanese" | /evidence="ECO:0000255\|PROSITE-ProRule:PRU00173" |  |  |  |
| H0UI37 | TSTD3_HUMAN | Thiosulfate sulfurtransferase/rhodanese-like domain-containing protein 3 (Rhodanese domain-containing protein 3) | TSTD3 | 97 | DOMAIN 32..84 | /note="Rhodanese" |  |  |  |  |
| Q9BTM9 | URM1_HUMAN | Ubiquitin-related modifier 1 | URM1 C9orf74 | 101 |  |  |  |  |  |  |
| P40818 | UBP8_HUMAN | Ubiquitin carboxyl-terminal hydrolase 8 (EC 3.4.19.12) (Deubiquitinating enzyme 8) (Ubiquitin isopeptidase Y) (hUBPy) (Ubiquitin thioesterase 8) (Ubiquitin-specific-processing protease 8) | USP8 KIAA0055 UBPY | 1118 | DOMAIN 33..116 | /note="MIT" | DOMAIN 195..313 | /note="Rhodanese" | /evidence="ECO:0000255\|PROSITE-ProRule:PRU00173" | DOMAIN 777..1109 |

Supplemental file 3:

**Sequences retrieve by BlastP with *Arabidopsis thaliana* MOCS3 protein sequence (STR13 / Q9ZNWO)**

>tr|G7K8M6|G7K8M6_MEDTR/1465 Adenylyltransferase and sulfurtransferase MOCS3 OS=Medicago truncatula OX=3880 GN=11429995 PE=3 SV=1MSSLASEILQELESLKSEKTKIEQKISQLESQLKDINLPKQHASSASAASSNGSSSPYPTNGLEPHMIHRYSRHLVLPSFGVQGQANLLKSSILVVGAGGLGAPALLYFAASGVGKLGIVDHDKVELSNMHRQIIHTEAYIGQPKVKSAAAACRAVNSSVEVVEHEEALRTSNALEIFSKYDIIVDATDNAPTRYLISDCCVVLGKPLVSGAAVGLEGQLTIYNHNGGPCYRCLFPTPPPRSACQSCADNGVLGVVPGIIGCLQALEAIKIAAAVGEPLSGRMLLFDALSSRIRVVKIRGRSMHCEACGDNARFNKQYFREFDYEKFTQTPLRVPPLKLNLLPSESRISSMEYKEITLNKEPHVLVDVRPAHHFKIVSLPNSLNIPFSTLESRLPEISSILKKDEEEEKGAVSESSAQLYVVCRRGNDSQRAVQYLHKMGFTSAKDIVGGLESWAQNVDPNFPTY

>tr|I1H0M3|I1H0M3_BRADI/1482 Adenylyltransferase and sulfurtransferase MOCS3 OS=Brachypodium distachyon OX=15368 GN=100842319 PE=3 SV=1MDGGGGGRREVVAGELERLRAERQELDIRIRLLESELEAGSAAPASPAGEDAAAGVEDGLCGGGSGGACQTRREFVESGALPADMIYRYSRHLLLPDFGVEGQRKLSRSSVLVVGAGGLGSPVALYLAACGVGVLGIVDGDDVELNNLHRQIIHKEAYVGRSKVKSAADACRAINSSIKLVEHHHTLKPSNALEIVRKYDIVVDATDNLPTRYMISDCCVLLNKPLISGAALGLEGQLTVYHHNGSPCYRCLFPNPPPVAACQRCSDSGVLGVVPGVIGCLQALEAIKVATDVGEPLCGRMLLFDALSARIRIVKIRGSSPVCTICGEKSVFTQEEFQKFDYEKFTESPMSDKSTPSLSLLPDSARVTCTEYKRMIDKGEPHLLLDVRPAHHFQITSLPQSLNIPLSVLEEKLPLLEISLKETMDTSAASDEQPSLYVVCRRGNDSQSAVQLLREKGFPSAKDIVGGLQSWAQDVDPDFPAY

>tr|I1M6M9|I1M6M9_SOYBN/1457 Adenylyltransferase and sulfurtransferase MOCS3 OS=Glycine max OX=3847 GN=100814736 PE=3 SV=1MSASEILRELDSLKDEKTKIEHKISALEAQLREINLQNDAAPPNASSSSSYPTNGLTQDMIHRYSRHLVLPSFGVQGQANLLKSSILVVGAGGLGAPALLYFAASGVGRLGVIDHDVVELNNMHRQVIHTEAYVGKPKVKSAAAACCSINSTIQVVEHEEALQTSNALEILSKYDIIVDATDNAPTRYLISDCCVVLGKPLVSGAALGLEGQLTVYNYNGGPCYRCLFPTPPPRTACQSCAEGGVLGVVPGIIGCLQALEAIKIAASVGEPLSGRMLLLDALSGRIRIVKIRGRSMQCEACGENATFTQQQFREFDYEKFTQTPLRVPPLKLNLLPRESRISSKEYSEVIIKKGPHVLVDVRPAHHFKIASLPKSLNIPLSTLEARLPEVSSALKKEEEESGAVSGSSAQLYVVCRRGNDSQRAVQYLHKMGFTSAKDIVGGLESWAHNVDHQFPTY

>tr|A0A022RJM4|A0A022RJM4_ERYGU/1458 Adenylyltransferase and sulfurtransferase MOCS3 OS=Erythranthe guttata OX=4155 GN=MOCS3 PE=3 SV=1MDSGGERLTASEIHTELASLQTAKAAIDERISTLESLLKNITTEDESETASSTPAAAETPLTGQLTADMIYRYSRHLLLPSFGVEAQSNLLNSSVLVIGAGGLGSPALLYLAACGFGRVGIVDHDVVELNNLHRQIIHTEAYIGRSKVESAAATCRAINSTVRIVEHKEAFRTSNALDIMKKYDIVVDATDNAPSRYMISDCCVVLGKPLISGAALGLEGQLTVYNYNGGPCYRCLFPTPPPTTACQRCADSGVLGVVPGIIGCLQALEAIKVAGAVGEPLSGRMLLLDALSARIRIVKIRGRSLQCEACGENAPMTEEQFRNFDYEKFTQSPLSTSPLKLDLLPEDARITSKEYYEKNIKGEAHVLIDVRPAHHYKIISLPNSLNIPLSSLEQRLSEISEALGRKEESASLYVVCRRGNDSQRAVQYLRGAGFNLAKDIIGGLESWAHDVDPKFPTY

>tr|A0A059BLS1|A0A059BLS1_EUCGR/1464 Adenylyltransferase and sulfurtransferase MOCS3 OS=Eucalyptus grandis OX=71139 GN=MOCS3 PE=3 SV=1MDSAGADAARILGEIEALKATRSDIEAKISALEAQLKGLSCRDGNGSCPPATAASAGLGLGLGLGHGLAPEMIYRYSRQLLLPSFGVQAQSNLLKSSILVVGAGGLGSPALLYLAACGVGRLGIVDNDVVELNNMHRQIIHTEAYIGRSKVESAAAACRAINSTVQIVEHREALRTSNALEILSKYDIVVDATDNAPSRYMISDCCVVLGKPLVSGAALGLEGQLTVYNCSGGPCYRCLFPTPPPTTACQRCSDSGVLGVVPGIIGCLQALEAIKIASAVGEPLVERMLLFDALSGRIRIVKIRGRSLLCEVCGENATFSRQQFQEFDYEKFTQAPLSASAPPKLNLLSEDSRISSKDYSERLKNGEAHILVDVRPEHHFRIVSLPNSLNIPLSTLEKRVAEISSALKEAEGQRDANLYVICRRGNDSQRAVNHLQKIGFTSAKDIMGGLESWARDVDPSFPTY

>tr|A0A061F4K6|A0A061F4K6_THECC/1462 Adenylyltransferase and sulfurtransferase MOCS3 OS=Theobroma cacao OX=3641 GN=MOCS3 PE=3 SV=1MQPNGDDVARIRREIEALKDTKASIEHKIAVLEAQLQQHQSDDVCNGSCPPISAVDANLANGLSADSVYRYSRHLLLPSFGVEAQSNLLKSSILVVGAGGLGSPALLYLAACGVGRLGIVDHDMVELNNMHRQVIHTEAYIGQPKVKSAAAACCAINSTIQIVEHKEALRTSNALEILSQYDIIIDATDNAPSRYMISDCCVVLGKPLVSGAALGLEGQLTVYNYKGGPCYRCLFPTPPPTTACQRCSDSGVLGVVPGIIGCLQALEAIKIASAVGEPLSGRMLLFDALSARIRIVKIRGRSLQCEVCGENTTFNQQYFKDFDYEKFTQSPLSTSPPKLNLLAADSRITSKEYKERIANGEAHVLVDVRPEHHYRIVSIPNSLNIPLASLEARLPEVSSALKEEQEHNGTVSGANLYVICRRGNDSQRAVQCLHKMGFDLAKDIVGGLESWAHDVDPNFPMY

>tr|A0A067F7J0|A0A067F7J0_CITSI/1444 Adenylyltransferase and sulfurtransferase MOCS3 OS=Citrus sinensis OX=2711 GN=MOCS3 PE=3 SV=1METNGGSTDVARVLGEIETLKAAKSDIDYRISALEAQLRDTTVSQPQTDTVSNGSYRPSSAVDYGLSPDMIYRYSRHLLLPSFGVEGQSNLLKSSILVIGAGGLGSPALLYLAACGVGRLGIVDHDVVELNNMHRINSTVHIIEHREALRTSNALEILSQYEIVVDATDNAPSRYMISDCCVVLGKPLVSGAALGLEGQLTVYNYNGGPCYRCLFPTPPPTTACQRCADSGVLGVVPGIIGCLQALEAIKVASAVGEPLSGRMLLFDALSARIRIVKIRGRSSQCEACGENSTFTQDHFRNFDYEKFTQSPLSTLPLKLNLLSADSRISSKEYKEKVVNGEAHILVDVRPAHHFRIVSLPNSINIPLSDLESRLPEISSAMKEKEEHRGSNASSGSNLYVVCRRGNDSQRAVQALHKLGFTSARDIIGGLESWANDVDPSFPVY

>tr|A0A067FFX5|A0A067FFX5_CITSI/1467 Adenylyltransferase and sulfurtransferase MOCS3 OS=Citrus sinensis OX=2711 GN=MOCS3 PE=3 SV=1METNGGSTDVARVLGEIETLKAAKSDIDYRISALEAQLRDTTVSQPQTDTVSNGSYRPSSAVDYGLSPDMIYRYSRHLLLPSFGVEGQSNLLKSSILVIGAGGLGSPALLYLAACGVGRLGIVDHDVVELNNMHRQVIHTEPYIGQSKVKSAAATCRSINSTVHIIEHREALRTSNALEILSQYEIVVDATDNAPSRYMISDCCVVLGKPLVSGAALGLEGQLTVYNYNGGPCYRCLFPTPPPTTACQRCADSGVLGVVPGIIGCLQALEAIKVASAVGEPLSGRMLLFDALSARIRIVKIRGRSSQCEACGENSTFTQDHFRNFDYEKFTQSPLSTLPLKLNLLSADSRISSKEYKEKVVNGEAHILVDVRPAHHFRIVSLPNSINIPLSDLESRLPEISSAMKEKEEHRGSNASSGSNLYVVCRRGNDSQRAVQALHKLGFTSARDIIGGLESWANDVDPSFPVY

>tr|A0A067JK60|A0A067JK60_JATCU/1467 Adenylyltransferase and sulfurtransferase MOCS3 OS=Jatropha curcas OX=180498 GN=MOCS3 PE=3 SV=1MESNGTDVAQILDEIKTLKATKTDIENRIAALEAKLRHLNLPDDTVFSNDSSLPVSIVDSGFGHGLSSDTIYRYSRHLLLPSFGVQAQSNLLKSSILVVGAGGLGSPALLYLAASGVGRLGIVDHDVVELNNMHRQVIHTEPFIGQPKVKSAAAACRSINSTIQIVEHQEALRTHNALEIFSQYDVIVDATDNAPSRYMISDCCVVLGKPLVSGAALGLEGQLTVYNYKGGPCYRCLFPTPPPTTACQRCADSGVLGVVPGIIGCLQALEAIKIASDVGEPLSGRMLLFDALSARIRIVKIRGRSLNCEVCGENAAFNEKQFRDFDYEKFTQSPLSTAPPKLNLLSAGSRITCKDFNEKVVKGEAHVLVDVRPAHHFKIVALPNALNIPLTSLEARLPEISSALKEAGGCRGTESESGVSLYIVCRRGNDSQRAVQMLHSKGFTSAKDIIGGLEAWSHDVDPKFPTY

>tr|A0A067KW31|A0A067KW31_JATCU/1467 Adenylyltransferase and sulfurtransferase MOCS3 OS=Jatropha curcas OX=180498 GN=MOCS3 PE=3 SV=1MKSNGTDAAQILGEIETLKATKSDIENRIAALEAKLRHLNLPNDNVSSNGSSLPVSIVDYGFGHGLSSDMIYRYSRHLLLPSFGVQGQSNLLKSSILVVGAGGLGSPALLYLAACGVGRLGIVDHDVVELNNMHRQVIHTEPFIGQPKVKSAAAACRSINSTIQIVEHQEALRTYNALEIFSQYDIIVDATDNAPSRYMISDCCVVLGKPLVSGAALGLEGQLTVYNYKGGPCYRCLFPPPPPTTACQRCADSGVLGVVPGIIGCLQALEAIKIASDIGEPLSGRMLLLDALSARIRIVKIRSRSLNCEVCGETAAFNQKQFRDFDYEKFTQSPLSTAPPKLNLLSAGSRITSKDFNEKIVKGEGHVLLDVRPAHHFKIVALPNALNIPLASLEARLPEISSALKEEGERRGPESESGVSLYVVCRRGNDSQRAVQLLQSKGFSSAKDIIGGLEAWSYDVDPKFPTY

>tr|A0A068UGA6|A0A068UGA6_COFCA/1479 Adenylyltransferase and sulfurtransferase MOCS3 OS=Coffea canephora OX=49390 GN=MOCS3 PE=3 SV=1MESNGRPQASEILCQIESLRSSKDEIERQISDLEAQLQQLNCDENNCRKAEEDEVNSNGSSSCLILPSENGSFDPGHGLASDMIYRYSRQLLLPAFGVQGQANLLKSSVLVIGAGGLGSPALLYLAACGFGRIGIVDHDVVELNNLHRQIIHTEAYIGRPKVESAAAACRAINSTVEIVEHKEAFRATNALGIVRKYDIVVDATDNVPSRYLINDCCVVLGKPLVSGAALGLEGQLTVYNYNGGPCYRCLFPTPPPTTACQRCADSGVLGVVPGIIGCLQALEAIKIGSVVGDPFSGRMLLFDAISGRIRIVKIRGRSLQCEACGENAPLTEKQFQEFDYERFTQTPFSTAPLKLSLLPVDARISSKEYHERVVKGEPHVLVDVRPAHHYKIVSLPNSINIPFPSLEARLHEISSALMKKDESKNSADSNASLYVVCRRGNESQRAVEYLHKMGFSSAKDIIGGIQSWAHDVDPKFPTY

>tr|A0A078FVW3|A0A078FVW3_BRANA/1466 Adenylyltransferase and sulfurtransferase MOCS3 OS=Brassica napus OX=3708 GN=BnaA10g09380D PE=3 SV=1MAANGGDSSEIVRELKELKLQKAKIEHRISALEAKLQETAAVEHCNAVSNGCSVPTEVKYGLEHGMSPDQIYRYSRQLLLPSFGVEGQSNLLKSSVLVIGAGGLGSPALLYLAACGVGRLGIIDHDVVELNNMHRQVIHTEAFIGHPKVKSAATACRSINSTIKIDEYVEALRTSNALEILSQYDIIVDATDNPPSRYMISDCCVLLGKPLVSGAALGMEGQLTVYNHKGGPCYRCLFPTPPPTTACQRCSDSGVLGVVPGAIGCLQALETIKLASMVGEPLSERMLLFDALSARIRIVKIRGRSAQCTVCGDNSSFNKQQFKDFDYEEFTQFPLSAGPLNLLPAESRISSKEFKEILQKKERHVLLDVRPSHHYKIVSLPDSLNIPFANLEARLNELTSALKDKEDDHVNSGSCANPSLYVVCRRGNDSQRAVQYLRDSGFSSAKDIIGGLEAWAADVNPNFPSY

>tr|A0A078G4L7|A0A078G4L7_BRANA/1466 Adenylyltransferase and sulfurtransferase MOCS3 OS=Brassica napus OX=3708 GN=BnaC09g31780D PE=3 SV=1MAANGGDSSEIVRELKELKLQKAKIEHRISALEAKLHETAAVERCDAVSNGCSVPTEIEHGLEHGMSPDQIYRYSRQLLLPSFGVEGQSNLLKSSVLVIGAGGLGSPALLYLAACGVGRLGIIDHDVVELNNMHRQVIHTEAFIGHPKVKSAATACRSINSTIKIDEYVEALRTSNALEILSQYDIIVDATDNPPSRYMISDCCVLLGKPLVSGAALGMEGQLTVYNHKGGPCYRCLFSTPPPTTACQRCSDSGVLGVVPGAIGCLQALETIKLASMVGEPLSERMLLFDALSARIRIVKIRGRSAQCTVCGDNSSFNKQQFKDFDYEEFTQFPLSAGPLNLLPEESRISSKEFKEILQKKERHVLLDVRPSHHYKIVSLPDSLNIPFANLEARLNELTSALKEKEDGHVNSGSCANPSLYVVCRRGNDSQRAVQYLRDSGFSSAKDIIGGLEAWAAEVNPNFPSY

>tr|A0A087GB63|A0A087GB63_ARAAL/1467 Adenylyltransferase and sulfurtransferase MOCS3 OS=Arabis alpina OX=50452 GN=MOCS3 PE=3 SV=1MESNGGDSSEIVRELEALKLSKAELEHRISTLEAKLQETTAAVERCDAVSNGYSSSSAPVIEYGLEHGLSPDQIYRYSRQLLLPSFGIEAQSSLLKSSVLVIGAGGLGSPALLYLAACGVGRLGIIDHDVVELNNMHRQIIHTEAFIGHPKVKSAAAACRSVNSTIRIDEHVEALRTSNALEIISQYDIIVDATDNPPSRYMISDCCVLLGKPLVSGAALGMEGQLTVYNHNGGPCYRCLFPTPPPTSACQRCSDSGVLGVVPGIIGCLQALETIKLASMVGEPLSERMLLFDALSARVRIVKIRGRSAQCTVCGDNSSFNKQQFKNFDYEDFTQFPLTAGPLNLLPTESRISSKEFKEILQKKEQHVLLDVRPSHHYKIVSLPDSLNIPLANLEARLNELTSAVKEKEDGHDNTRSCSSIYIVCRRGNDSQRAVQYLRESGFDSAKDIIGGLEAWATDVNPNFPTY

>tr|A0A0A0L690|A0A0A0L690_CUCSA/1460 Adenylyltransferase and sulfurtransferase MOCS3 OS=Cucumis sativus OX=3659 GN=MOCS3 PE=3 SV=1MASTTDESSRILHEIETLKSAKFDLERRISALESQLHNLNQPHNNGVSNASSTSPSTFPHALSPDMIYRYSRHLLLPSFGVQGQLRLSKSSILVVGAGGLGSPALLYLAASGVGRLGIVDHDVVELNNMHRQIIHTEAYIGQSKVESAAATCRSINSTVQIVEHKEALRTSNALEIFSKYDIIVDATDNAPSRYMISDCCVVMGKPLVSGAALGLEGQLTVYNYNGGPCYRCLFPTPPPTTACQRCADSGVLGVVPGIIGCLQALEAIKIASAVGDPLSGRMLLFDALAARIRIVKIRGRSVQCEVCGENSEFKAAQFQEFDYEKFTQTPLSTSPLKLKLLEPNSRISAKEYRDRLRSGEPHVLVDVRPEHHFKIVSLPNSLNVPLASLEGRVEEVVWALKEKEENKQNDDVKVYVVCRRGNDSQRAVKYLQEKGYPSAKDIIGGLEGWAQEVDPTFPSY

>tr|A0A0B0P4P3|A0A0B0P4P3_GOSAR/1470 Adenylyltransferase and sulfurtransferase MOCS3 OS=Gossypium arboreum OX=29729 GN=LOC108450313 PE=3 SV=1MESNGGGASPIRREIEALKEEKARIEQRISVLEAQLQEEASLALQQQQNDGVCNGFCPSEISSVDANLAHGLSADSIYRYSRHLLLPSFGVQAQSNLLKSSILVVGAGGLGSPALLYLAACGVGRLGIVDHDVVELNNMHRQVIHTEAYIGQPKVKSAAAACRSINSTIQIVEHKQALRTSNALEILSQYDIVVDATDNAPSRYMISDCCVVLGKPLVSGAALGLEGQLTVYNYKGGPCYRCLFPTPPPTTACQRCSDSGVLGVVPGIIGCLQALEAIKIASDIGEPLSGRMLLFDALSARIRIVKIRGRSLQCEVCGENTTFNQQQFKDLDYEKFTQSPLSTSLQKLKLLSPDSRITSKEYKERIISGEPHVLVDVRPELHYKIVSIPNSLNIPLASLEARLPEISSALKEQEKESTGSGANLYVICRRGNDSQRAVDYLRNKGFDLAKDIVGGLDSWANDVDPNFPMY

>tr|A0A0B2QW85|A0A0B2QW85_GLYSO/1457 Adenylyltransferase and sulfurtransferase MOCS3 OS=Glycine soja OX=3848 GN=MOCS3 PE=3 SV=1MSASEILRELDSLKDEKTKIEHKISALEAQLREINLQNDAAPPNASSSSSYPTNGLTQDMIHRYSRHLVLPSFGVQGQANLLKSSILVVGAGGLGAPALLYFAASGVGRLGVIDHDVVELNNMHRQVIHTEAYVGKPKVKSAAAACCSINSTIQVVEHEEALQTSNALEILSKYDIIVDATDNAPTRYLISDCCVVLGKPLVSGAALGLEGQLTVYNYNGGPCYRCLFPTPPPRTACQSCAEGGVLGVVPGIIGCLQALEAIKIAASVGEPLSGRMLILDALSGRIRIVKIRGRSMQCEACGENATFTQQQFREFDYEKFTQTPLRVPPLKLNLLPRESRISSKEYSEVIIKKGPHVLVDVRPAHHFKIASLPKSLNIPLSTLEARLPEVSSALKKEEEESGAVSGSSAQLYVVCRRGNDSQRAVQYLHKMGFTSAKDIVGGLESWAHNVDHQFPTY

>tr|A0A0D2QY66|A0A0D2QY66_GOSRA/1470 Adenylyltransferase and sulfurtransferase MOCS3 OS=Gossypium raimondii OX=29730 GN=MOCS3 PE=3 SV=1MESNGGGASPIRREIETLKEERARIEQRISFLEAQLQEEASLAPQQQQNDGVCNGFCPSETSTVDANLAHGLSADSIYRYSRHLLLPSFGVKAQSNLLKSSILVVGAGGLGSPALLYLAACGVGRLGIVDHDVVELNNMHRQVIHTEAYIGQPKVKSAAAACRSINSTIQIVEHKQALRTSNALEILSQYDIVVDATDNAPSRYMISDCCVVLGKPLVSGAALGLEGQLTVYNYKGGPCYRCLFPTPPPTTACQRCSDSGVLGVVPGIIGCLQALEAIKIASDIGEPLSGRMLLFDALSARIRIVKIRGRSLQCEVCGENTTFNQQQFKELDYEKFTQSPLSTSPPKLKLLAPDSRITSKEYKERIISGEPHVLVDVRPELHYKIVSMPNSLNIPLASLVTRLPEISSALKEQEKEGTRSGANLYVICRRGNDSQRAVDYLRNKGFDLAKDIVGGLESWANDVDPNFPMY

>tr|A0A0D3EB85|A0A0D3EB85_BRAOL/1466 Adenylyltransferase and sulfurtransferase MOCS3 OS=Brassica oleracea var. oleracea OX=109376 GN=MOCS3 PE=3 SV=1MEANGGGSSEIVRELKELKLQKAEIEHRISALEAKLQETAAVERCDAVSNGYSVPAEIEHGLEHGMSPDQIYRYSRQLLLPSFGVEGQSNLLKSSVLVIGAGGLGSPALLYLAACGVGRLGIIDHDVVELNNMHRQVIHTEAFIGHPKVKSAATACRSINSTIKIDEYVEALRTSNALEILSQYDIIVDATDNPPSRYMISDCCVLLGKPLVSGAALGMEGQLTVYNHKGGPCYRCLFPTPPPTTACQRCSDSGVLGVVPGAIGCLQALETIKLASMVGEPLSERMLLFDALSARIRIVKIRGRSAQCTVCGDNSSFNKQQFKDFDYEEFTQFPLSAGPLNLLPAESRISSKEFKEIRQKKERHVLLDVRPSHHYKIVSLPDSLNIPFANLEARLNELTSALKEKEDGHVNSGSCANPSLYVVCRRGNDSQRAVQYLRDSGFSSAKDIIGGLEAWAADVNPNFPSY

>tr|A0A0D3F5U0|A0A0D3F5U0_9ORYZ/1473 Adenylyltransferase and sulfurtransferase MOCS3 OS=Oryza barthii OX=65489 GN=MOCS3 PE=3 SV=1MEAMAGELERLRAEREELDSRIRLLESQLGASPTPAGEGDAAGTGAGGGGGGGGATACPIRRRGNGFAAADGLPADMIYRYSRHLLLPDFGVEGQRKLSQSSILVVGAGGLGSPVALYLAACGVGCLGIVDGDDVELNNLHRQIIHKEAYVGKSKVKSAADACREINSSINVMEYHHTLKPSNALEIVRKYDIVVDATDNLPTRYMISDCCVLLNKPLISGAALGLEGQLTVYHHNGSPCYRCLFPNPPPVAACQRCSDSGVLGVVPGVIGCLQALEAIKVATDVGEPLSGRMLLFDALSARIRIVKIRGSSTVCTVCGENSAFTQDDFQKFDYENFTQSPMSDKSAPSLDILPGSARVTCKEYKRLVDNGERHLLLDVRPAHHFQIASVSQSLNIPLSELEEKLQMLETSLKDTTDASSSDKPPSLYVVCRRGNDSQIAVQLLREKGFLSAKDIIGGLQSWAQDVDPDFPVY

>tr|A0A0D3F5U1|A0A0D3F5U1_9ORYZ/1397 Adenylyltransferase and sulfurtransferase MOCS3 OS=Oryza barthii OX=65489 GN=MOCS3 PE=3 SV=1MIYRYSRHLLLPDFGVEGQRKLSQSSILVVGAGGLGSPVALYLAACGVGCLGIVDGDDVELNNLHRQIIHKEAYVGKSKVKSAADACREINSSINVMEYHHTLKPSNALEIVRKYDIVVDATDNLPTRYMISDCCVLLNKPLISGAALGLEGQLTVYHHNGSPCYRCLFPNPPPVAACQRCSDSGVLGVVPGVIGCLQALEAIKVATDVGEPLSGRMLLFDALSARIRIVKIRGSSTVCTVCGENSAFTQDDFQKFDYENFTQSPMSDKSAPSLDILPGSARVTCKEYKRLVDNGERHLLLDVRPAHHFQIASVSQSLNIPLSELEEKLQMLETSLKDTTDASSSDKPPSLYVVCRRGNDSQIAVQLLREKGFLSAKDIIGGLQSWAQDVDPDFPVY

>tr|A0A0D9VGI5|A0A0D9VGI5_9ORYZ/1471 Adenylyltransferase and sulfurtransferase MOCS3 OS=Leersia perrieri OX=77586 GN=MOCS3 PE=3 SV=1MEAMAGELARLRAEREELDRRIRLLESQLEATPRSSASPAGEAIGGGGGGIGAAACPIRRRGNGFSAADGLPADMIYRYSRHLLLPDFGVEGQRKLSGSSILVVGAGGLGSPVALYLAACGVGCLGIVDGDDVELNNLHRQIIHKEAYVGLSKVKSAADACREINSSIKVVEHHHTLKPCNALEIVRNYDIVVDATDNLPTRYMISDCCVLLNKPLVSGAALGLEGQLTVYHHNGSPCYRCLFPNPPPVAACQRCSDSGVLGVVPGVIGCLQALEAIKVATDVGEPLSGRMLLFDALSARIRIVKIRGSSSVCTVCGENSAFTQDDFQEFDYENFTQSPMSDKSAPSLDLLPESDRITCKEYKRLVDNGEPHLLLDVRPAHHFQITSVSQSLNIPLSELEEKLQILETSLKETMNTSTLDKVPPLFVVCRRGNDSQLAVQLLREKGFLSAKDIIGGLQSWARDVDHDFPVY

>tr|A0A0D9YT07|A0A0D9YT07_9ORYZ/1473 Adenylyltransferase and sulfurtransferase MOCS3 OS=Oryza glumipatula OX=40148 GN=MOCS3 PE=3 SV=1MEAMAGELERLRAEREELDSRIRLLESQFGASLTPAGEGDAAGTGAGGGGGGGGATACPIRRRGNGFAAADGLPADMIYRYSRHLLLPDFGVEGQRKLSQSSILVVGAGGLGSPVALYLAACGVGCLGIVDGDDVELNNLHRQIIHKEAYVGKSKVKSAADACREINSSINVMEYHHTLIPCNALEIVRKYDIVVDATDNLPTRYMISDCCVLLNKPLISGAALGLEGQLTVYHHNGSPCYRCLFPNPPPVAACQRCSDSGVLGVVPGVIGCLQALEAIKVATDVGEPLSGRMLLFDALSARIRIVKIRGSSTVCTVCGENSAFTQDDFQKFDYENFTQSPMSDKSAPSLDILPGSARVTCKEYKRLVDNGERHLLLDVRPAHHFQIASVSQSLNIPLSELEEKLQMLETSLKDTTDASSSDKPPSLYVVCRRGNDSQIAVQLLREKGFLSAKDIIGGLQSWAQDVDPDFPVY

>tr|A0A0E0CLC7|A0A0E0CLC7_9ORYZ/1472 Adenylyltransferase and sulfurtransferase MOCS3 OS=Oryza meridionalis OX=40149 GN=MOCS3 PE=3 SV=1MEAMADELARLRAEREELDSRIRLLESQLGASPTPAGEGDAAGTGAGGGGGGAAAACPIRRRGNGFAAADGLPADMIYRYSRHLLLPDFGVEGQRKLSQSSILVVGAGGLGSPVALYLAACGVGCLGIVDGDDVELNNLHRQIIHKEAYVGKSKVKSAADACREINSSINVMEYHHTLKPSNALEIVRKYDIVVDATDNLPTRYMISDCCVLLNKPLISGAALGLEGQLTVYHHNGSPCYRCLFPNPPPVAACQRCSDSGVLGVVPGVIGCLQALEAIKVATDVGEPLSGRMLLFDALSARIRIVKIRGSSTVCTVCGENSAFTQDDFQKFDYENFTQSPMSDKSAPSLDILPGSARVTCKEYKRLVDNGERHLLLDVRPAHHFQIASVSQSLNIPLSELEEKLQILETSLKDTTDASTSDKPPSLYVVCRRGNDSQIAVQLLREKGFLSAKDIIGGLQSWAQDVDPDFPVY

>tr|A0A0E0G7J7|A0A0E0G7J7_ORYNI/1473 Adenylyltransferase and sulfurtransferase MOCS3 OS=Oryza nivara OX=4536 GN=MOCS3 PE=3 SV=1MEAMAGELERLRAEREELDSRIRLLESQLGASPTPAGEGDAAGTGAGGGGGGGGATACPIRRRGNGFAATDGLPADMIYRYSRHLLLPDFGVEGQRKLSQSSILVVGAGGLGSPVALYLAACGVGCLGIVDGDDVELNNLHRQIIHKEAFVGKSKVKSAADACREINSSINVMEYHHTLKPSNALEIVRKYDIVVDATDNLPTRYMISDCCVLLNKPLISGAALGLEGQLTVYHHNGSPCYRCLFPNPPPVAACQRCSDSGVLGVVPGVIGCLQALEAIKVATDVGEPLSGRMLLFDALSARIRIVKIRGSSTVCTVCGENSAFTQDDFQKFDYENFTQSPMSDKSAPSLDILPGSARVTCKEYKRLVDNGERHLLLDVRPAHHFQIASVSQSLNIPLSELEEKLQMLETSLKDTTDASSSDKPPSLYVVCRRGNDSQIAVQLLREKGFLSAKDIIGGLQSWAQDVDPDFPVY

>tr|A0A0E0K0I2|A0A0E0K0I2_ORYPU/1478 Adenylyltransferase and sulfurtransferase MOCS3 OS=Oryza punctata OX=4537 GN=MOCS3 PE=3 SV=1MEAITGELARLRAEREELDSRIRFLESQLEASPPPSAPPAGEGGAAGTGAGGGGGGAAAAAVCPIRRHGNGFSAADGLPTDMIYRYSRHLLLPDFGVEGQRKLSQSSILVVGAGGLGSPVALYLAACGVGCLGIVDGDDVELNNLHRQIIHKEAYVGKSKVKSAADACREINSSIKVMEYHHTLKPSNALEIVRKYDIVVDATDNLPTRYMISDCCVLLNKPLISGAALGLEGQLTVYHHNGSPCYRCLFPNPPPVAACQRCSDSGVLGVVPGVIGCLQALEAIKVATDVGEPLSGRMLLFDALSSRIRIVKIRGSSTVCTVCGENSAFTQDDFQKFDYENFTQSPMSDKSAPSLDILPESARVTCKEYKRLVDNGERHLLLDVRPAHHFQIASVSQSLNIPLSELEEKLQMLKTSLKGTIDVSSSDKPPSLYVLCRRGNDSQMAVQLLREKGFLSAKDIIGGLQSWAQDVDPDFPVY

>tr|A0A0E0K0I3|A0A0E0K0I3_ORYPU/1470 Adenylyltransferase and sulfurtransferase MOCS3 OS=Oryza punctata OX=4537 GN=MOCS3 PE=3 SV=1MEAITGELARLRAEREELDSRIRFLESQLEASPPPSAPPAGEGGAAGTGAGGGGGGAAAAAVCPIRRHGNGFSAADGLPTDMIYRYSRHLLLPDFGVEGQRKLSQSSILVVGAGGLGSPVALYLAACGVGCLGIVDGDDVELNNLHRQIIHKEAYVGKSKVKINSSIKVMEYHHTLKPSNALEIVRKYDIVVDATDNLPTRYMISDCCVLLNKPLISGAALGLEGQLTVYHHNGSPCYRCLFPNPPPVAACQRCSDSGVLGVVPGVIGCLQALEAIKVATDVGEPLSGRMLLFDALSSRIRIVKIRGSSTVCTVCGENSAFTQDDFQKFDYENFTQSPMSDKSAPSLDILPESARVTCKEYKRLVDNGERHLLLDVRPAHHFQIASVSQSLNIPLSELEEKLQMLKTSLKGTIDVSSSDKPPSLYVLCRRGNDSQMAVQLLREKGFLSAKDIIGGLQSWAQDVDPDFPVY

>tr|A0A0E0NFQ1|A0A0E0NFQ1_ORYRU/1473 Adenylyltransferase and sulfurtransferase MOCS3 OS=Oryza rufipogon OX=4529 GN=MOCS3 PE=3 SV=1MEAMAGELERLRAEREELDSRIRLLESQLGASPTPAGEGDAAGTGAGGGVGGGGATACPIRRRGNGFAAADGLPADMIYRYSRHLLLPDFGVEGQRKLSQSSILVVGAGGLGSPVALYLAACGVGCLGIVDGDDVELNNLHRQIIHKEAFVGKSKVKSAADACREINSSINVMEYHHTLKPSNALEIVRKYDIVVDATDNLPTRYMISDCCVLLNKPLISGAALGLEGQLTVYHHNGSPCYRCLFPNPPPVAACQRCSDSGVLGVVPGVIGCLQALEAIKVATDVGEPLSGRMLLFDALSARIRIVKIRGSSTVCTVCGENSAFTQDDFQKFDYENFTQSPMSDKSAPSLDILPGSARVTCKEYKRLADNGERHLLLDVRPAHHFQIASVSQSLNIPLSELEEKLQMLETSLKDTTDASSSDKPPSLYVVCRRGNDSQIAVQLLREKGFLSAKDIIGGLQSWAQDVDPDFPVY

>tr|A0A0K9PEX5|A0A0K9PEX5_ZOSMR/1458 Adenylyltransferase and sulfurtransferase MOCS3 OS=Zostera marina OX=29655 GN=MOCS3 PE=3 SV=1MERRAIEEEINKLQVEKLRIESRIISLQSQLNQNGQQKQLDDKNLSGWKTTSSPPYGDHGLSKELIYRYSRHLLLPDFGIQGQANLLNSSILVVGAGGLGSPVALYLAACGVGRLGIVDNDIVELNNLQRQIIHTEAFIGKHKVESAAAACRAINSTIDVIEHKGALLAVNALDIISKYDVVVDATDNIPTRYMINDGCVLLNKPLVSGAALGLEGQLTIYHYNGGPCYRCLFPTPPPTSACQRCSDSGVLGVVPGIIGCLQALEAIKLASSVGVSLSGRMLLFDALSARIRTVKIRERSLNCDACGENPIFTKKLFRSFDYENFTETPIFDLSCENLMLLSKEARGSSNEYEELLNNGKPHVLLDVRPAHHFQIASLPNSINIPLPNIEKELFTIDSALKEIGQCKETDSLYVICRRGNDSQIAVQILHQNGYSTAKDLIGGLISWANLVNCDFPIY

>tr|A0A0K9QUL2|A0A0K9QUL2_SPIOL/1463 Adenylyltransferase and sulfurtransferase MOCS3 OS=Spinacia oleracea OX=3562 GN=MOCS3 PE=3 SV=1MESQREEPNRKILDEIDALKAQKSDIEARISSLESQLRDSSLAKQCNGAFPSISTVASNHSRHGLPSDAIYRYSRHLLLPSFGVQGQSNLLKSSVLVVGAGGLGSPALLYLAACGVGRIGIVDHDVVELNNMHRQVIHTEAYIGKSKVESAAAACRSINSTIEIVEYREALRTSNALEIFSKFDLIIDATDNAPSRYMINDCCVVLKKPLVSGAAVGLEGQLTIYNYQGGPCYRCLFPTPPPVTACQRCSDSGVLGVVPGVIGILQALEAIKIASNVGEPLSGRMLLFDALSGRIRVVKIRGQSPQCEACGEKSTFNPEQFREFDYEKFTQTPLSTAPPKLNLIPVESRISCKEYNEKVVNGEAHILVDVRPAHHFDIISLPNSLNIPLSSLEARLSEISAALKEEETRKSVSGARLYTVCRRGNDSQRAVQLLHKLGFTSAKDIVGGLTSWQTEVDPDFPAY

>tr|A0A0L9UV02|A0A0L9UV02_PHAAN/1457 Adenylyltransferase and sulfurtransferase MOCS3 OS=Phaseolus angularis OX=3914 GN=MOCS3 PE=3 SV=1MSASEILRELDSLKDAKAKIEHKISALEAQLREINLRNDAAPSNGSSYPSSYPSNGLTQDMIHRYSRHLMLPSFGVEGQANLLKSSILVVGAGGLGAPALLYFAAAGVGRLGIVDHDVVELNNMHRQVIHTEAYVGKPKVKSAAAACLSVNSTIEVVEHEEALRTSNALEILSKYDIIVDATDNAPTRYLISDCCVLLGKPLVSGAALGMEGQLTVYNYKGGPCYRCLFPLPPPRTACQSCAEGGVLGVVPGIIGCLQALEAIKIAAAVGEPLSGRMLLLDALSGRIRIVKIRGRSMHCEACGENATFTKQKFQEFDYEKFTETPLRVPPLKLHLLPSESRISSKEYSEIILKKEPHVLVDVRPAHHFKIVSLPKSLNIPLSTLESRLPEISAALKKEEDSGLVSGSGAQLYVVCRRGNDSQRAVQCLHKMGFTSAKDIVGGLESWAYNVDPKFPTY

>tr|A0A0S3RJD9|A0A0S3RJD9_PHAAN/1457 Adenylyltransferase and sulfurtransferase MOCS3 OS=Vigna angularis var. angularis OX=157739 GN=Vigan.03G027900 PE=3 SV=1MSASEILRELDSLKDAKAKIEHKISALEAQLREINLRNDAAPSNGSSYPSSYPSNGLTQDMIHRYSRHLMLPSFGVEGQANLLKSSILVVGAGGLGAPALLYFAAAGVGRLGIVDHDVVELNNMHRQVIHTEAYVGKPKVKSAAAACLSVNSTIEVVEHEEALRTSNALEILSKYDIIVDATDNAPTRYLISDCCVLLGKPLVSGAALGMEGQLTVYNYKGGPCYRCLFPLPPPRTACQSCAEGGVLGVVPGIIGCLQALEAIKIAAAVGEPLSGRMLLLDALSGRIRIVKIRGRSMHCEACGENATFTKQKFQEFDYEKFTETPLRVPPLKLHLLPSESRISSKEYSEIILKKEPHVLVDVRPAHHFKIVSLPKSLNIPLSTLESRLPEISAALKKEEDSGLVSGSGAQLYVVCRRGNDSQRAVQCLHKMGFTSAKDIVGGLESWAYNVDPKFPTY

>tr|A0A151U286|A0A151U286_CAJCA/1398 Adenylyltransferase and sulfurtransferase MOCS3 OS=Cajanus cajan OX=3821 GN=MOCS3 PE=3 SV=1MIHRYSRHLMLPSFGVQGQANLLKSSILVVGAGGLGAPALLYIAASGVGRLGIVDHDVVELNNMHRQVIHTEAYVGKPKVESAAAACRSINSTIQVVEHQEALRTSNALEILSKYDIIIDATDNAPTRYMISDCCVVLGKPLVSGAALGLEGQLTVYNYNGGPCYRCLFPIPPPRTACQSCADGGVLGVVPGIIGCLQALEAIKIAASVGEPLSGRMLLLDALSGRIRIVKIRGRSMHCEACGENATFTQQQFREFDYEKFTQTPLCVAPVKLNLLPSESRISSKEYSEIILKKEPHVLVDVRPAHHFKIVSLPKSLNIPLSTLEARLPEVSSALKKEEEDSGVVSGSSAQLYVVCRRGNDSQRAVQYLHKLGFTSAKDIVGGIESWAHNVDPKFPTY

>tr|A0A178UPW9|A0A178UPW9_ARATH/1464 Adenylyltransferase and sulfurtransferase MOCS3 OS=Arabidopsis thaliana OX=3702 GN=MOCS3 PE=3 SV=1MMSNGGDSSEIVRELEELKLKKAEIEHRISTLEAKLQDTAAVELYDAVSNGDSYLTAPELEHGLSPDQIYRYSRQLLLPSFAVEGQSNLLKSSVLVIGAGGLGSPALLYLAACGVGRLGIIDHDVVELNNMHRQIIHTEAFIGHPKVKSAAAACRSINSTIKVDEYVEALRTSNALEILSQYDIIVDATDNPPSRYMISDCCVLLGKPLVSGAALGMEGQLTVYNHNGGPCYRCLFPTPPPTSACQRCSDSGVLGVVPGVIGCLQALETIKLASLVGEPLSERMLLFDALSARMRIVKIRGRSSQCTVCGDNSSFNKQTFKDFDYEDFTQFPLFAGPLNLLPAESRISSKEFKEILQKKEQHVLLDVRPSHHYKIVSLPDSLNIPLANLETRLNELTSALKEKENGHANTESCTNPSVFVVCRRGNDSQRAVQYLRESGFDSAKDIIGGLEAWAANVNPNFPTY

>tr|A0A1J3DGR1|A0A1J3DGR1_NOCCA/1465 Adenylyltransferase and sulfurtransferase MOCS3 OS=Noccaea caerulescens OX=107243 GN=MOCS3 PE=3 SV=1MMEANGGDSSEIVRELEELKLRKAEIERRISTLEAKLHETAAVERLEAVSNGYSSPTAVEHGLDHGLSPDQIYRYSRQLLLPSFGVEGQSNLLKSSVLVIGAGGLGSPALLYLAACGVARLGIIDHDVVELNNMHRQIIHTEAFIGHPKVKSAAAACRSVNSTIKIDEHVEALRTSNALEILTQYDIIVDATDNPPSRYMISDCCVLLGKPLVSGAALGMEGQLTVYNHNGGPCYRCLFPTPPPTSACQRCSDSGVLGVVPGVIGCLQAVETIKLASMVGEPLSERMLLFDALSARIRIVKIRGRSSQCTVCGDNSSFDKQQFKDFDYEDFTQFPLFAGPLNLLPRESRISSKEFKEILQKKEGHILLDVRPSHHYKIVSLPDSLNIPFANLDSRLNELTSVLKEKEDASDKTGSCTSLYVVCRRGNDSQRAVQYLRESGFDSAKDIIGGLEAWAAHVNPNFPTY

>tr|A0A1J3FRY1|A0A1J3FRY1_NOCCA/1465 Adenylyltransferase and sulfurtransferase MOCS3 OS=Noccaea caerulescens OX=107243 GN=MOCS3 PE=3 SV=1MMEANGGDSSEIVRELEELKLRKAEIKHRISTLEAKLDETAAVERLEAVSNGYSSPTAVEHGLDHGLSPDQIYRYSRQLLLPSFGVEGQSNLLKSSVLVIGAGGLGSPALLYLAACGVARLGIIDHDVVELNNMHRQIIHTEAFIGHPKVKSAAAACRSVNSTIKIDQHVEALRTSNALEILTQYDIIVDATDNPPSRYMISDCCVLLGKPLVSGAALGMEGQLTVYNHNGGPCYRCLFPTPPPTSACQRCSDSGVLGVVPGVIGCLQAVETIKLASMVGEPLSERMLLFDALSARIRIVKIRGRSSQCTVCGDNSSFDKQQFKDFDYEDFTQFPLFAGPLNLLPRESRISSKEFKEILQKKERHILLDVRPSHHYKIVSLPDSLNIPFANLDSRLNELTSVLKEKEDASDKTGSCTSLYVVCRRGNDSQRAVQYLRESGFDSAKDIIGGLEAWAAHVNPNFPTY

>tr|A0A1J3K5Q3|A0A1J3K5Q3_NOCCA/1465 Adenylyltransferase and sulfurtransferase MOCS3 OS=Noccaea caerulescens OX=107243 GN=MOCS3 PE=3 SV=1MMEANGGDSSEIVRELEELKLRKAEIEHRISTLEAKLHETAAVERLEAVSNGYSSPTAVEHGLDHGLSPDQIYRYSRQLLLPSFGVEGQSNLLKSSVLVIGAGGLGSPALLYLAACGVARLGIIDHDVVELNNMHRQIIHTEAFIGHPKVKSAAAACRSVNSTIKIDQHVEALRTSNALEILTQYDIIVDATDNPPSRYMISDCCVLLGKPLVSGAALGMEGQLTVYNHNGGPCYRCLFPTPPPTSACQRCSDSGVLGVVPGVIGCLQAVETIKLASMVGEPLSERMLLFDALSARIRIVKIRGRSSQCTVCGDNSSFDKQQFRDFDYEDFTQFPLFAGPLNLLPRESRISSKEFKEILQKKERHILLDVRPSHHYKIVSLPDSLNIPFANLDSRLNELTSVLKEKEDASDKTGSCTSLYVVCRRGNDSQRAVQYLRESGFDSAKDIIGGLEAWAAHVNPNFPTY

>tr|A0A1Q3D098|A0A1Q3D098_CEPFO/1465 Adenylyltransferase and sulfurtransferase MOCS3 OS=Cephalotus follicularis OX=3775 GN=MOCS3 PE=3 SV=1MDSNGDDVSRILGELETLKGTKTDIENRILLLETQLRELNSQNDTVLNGYSQSISAVDSCFAHGLSPDAIYRYSRHLLLPSFGVTGQSNLLKSSILVVGAGGLGSPALLYLAACGVGRLGIVDHDVVELNNMHRQVIHTEAYIGQPKAKSAAAACRSINSTIQISEHQEALCTSNALEILSQYDIVVDATDNAPSRYMINDCCVVLGKPLVSGAALGVEGQLTVYNYNGGPCYRCLFPTPPPTTACQRCADSGVLGVVPGIIGCLQALEAIKIASAVGEPLSGRMLLFDALSARIRIVKIRGRPSQCEACGENALSQQQFREFDYEKFTQSPLSTAPLKLNLIPEDSRISSKEYKEKVLNREAHVLVDVRPVHHFSIVSLPKSLNIPLSSLEARLPEISSALKQEEECNMNDFGVGASLYLVCRRGNDSQRAVQYLHKMGFGSARDIIGGLTSWAQDVDPNFPIY

>tr|A0A1S2XM41|A0A1S2XM41_CICAR/1450 Adenylyltransferase and sulfurtransferase MOCS3 OS=Cicer arietinum OX=3827 GN=LOC101513756 PE=3 SV=1MASEILQQLESLKNEKTKIEHKISLLEAQLKDINLQNDAASSYLTNGLAPHMIHRYSRHLVLPSFGVQGQANLLKSSILVVGAGGLGAPALLYFAASGVGRLGIVDHDKVELNNMHRQIIHTEAYIGQPKVKSAAAACRSVNSSIEVVEHEEALRTSNALEILSKYDIIVDATDNAPTRYMISDCCVVLGKPLVSGAALGLEGQLTIYNHNGGPCYRCLFPTPPPRTACQSCADGGVLGVVPGIIGCLQALEAIKIAASVGEPLSGRMLLFDALAARIRVVKIRGRSLQCEACGENATFNQQYFREFDYEKFTQTPLCVPPTKLNLLPNESRISSNEYKEIVVNKEPHVLVDVRPAHHFKIVSLPNSLNIPLSTLESRLPEISSILKKDDEEEKGLVNGSSAKLYVVCRRGNDSQRAVQYLQKMGFTSAKDIVGGLESWAQNVDPNFPTY

>tr|A0A1S3BLE2|A0A1S3BLE2_CUCME/1467 Adenylyltransferase and sulfurtransferase MOCS3 OS=Cucumis melo OX=3656 GN=LOC103491093 PE=3 SV=1MASTTGESSRILHEIETLKSAKSDLERRISALESQLHNLNQPHNNGVSNASSTSPSTFPHGLSPDMIYRYSRHLLLPSFGVQGQLRLSKSSVLVVGAGGLGSPALLYLAASGVGRLGIVDHDVVELNNMHRQIIHTEAYIGRSKVESAAATCRSINSTVQIVEHKEALRTSNALEIFSKYDIIVDATDNAPSRYMISDCCVVLGKPLVSGAALGLEGQLTVYNYNGGPCYRCLFPTPPPTTACQRCADSGVLGVVPGIIGCLQALEAIKIASAVGDPLSGRMLLFDALAARIRIVKIRGRSVQCEVCGENSEFKAAQFQEFDYEKFTQSPLSTSPLKLKLLEPNSRISAREYRDRLHSNESHVLVDVRPEHHFKIVSLPNSLNIPLASLDGRMEEVVSALKEEEHKHKQKSKSSSNGDVELYVVCRRGNDSQRAVKYLQENGYPSAKDIIGGLEGWAQEVDPTFPSY

>tr|A0A1S3U9H3|A0A1S3U9H3_VIGRR/1457 Adenylyltransferase and sulfurtransferase MOCS3 OS=Vigna radiata var. radiata OX=3916 GN=LOC106763027 PE=3 SV=1MSASEILRELDSLKDAKAKIEHKISALEAQLREINLQNDAAPPNGSSYPSSYPTNGLTQDMIHRYSRHLMLPSFGVEGQANLLKSSILVVGAGGLGAPALLYFAAAGVGRLGIVDHDVVELNNMHRQVIHTEAYVGKPKVKSAAAACLSVNSTIEVVEHEEALRTSNALEILSKYDIIVDATDNAPTRYLISDCCVVLGKPLVSGAALGMEGQLTVYNYKGGPCYRCLFPLPPPRTACQSCAEGGVLGVVPGIIGSLQALEAIKIAAAVGEPLSGRMLLLDALSGRTRIVKIRGRSMHCEACGENATFTEQKFQEFDYEKFTETPLRVPPLKLHLLPSESRISSKEYSEIILKKEPHVLVDVRPAHHFKIVSLPKSLNIPLSTLESRLPEISSALKKEEDSGLVSGSGAQLYVVCRRGNDSQRAVQYLHKMGFTSAKDIVGGLESWAYNVDPKFPTY

>tr|A0A1U7Z5Q9|A0A1U7Z5Q9_NELNU/1465 Adenylyltransferase and sulfurtransferase MOCS3 OS=Nelumbo nucifera OX=4432 GN=LOC104586577 PE=3 SV=1MESNGGDAFRILEEIETLKAAKSDIEHRIANLQVQIGGVAVEEMTDSILCPPISTNDAGFGHGLSPEMVYRYSRQLLLPSFGVKGQSNLLKSSILVVGAGGLGSPALLYLAACGVGRLGIVDHDDVELNNMHRQIIHTEAYIGQSKVKSAAAACRAINSTIDIVEHREALRTSNALEIVRKYDIVIDATDNLPSRYMISDCCVVLGKPLVSGAALGLEGQLTVYNHIGSPCYRCLFPTPPPTSACQRCSDSGVLGVVPGVIGCLQALEAIKIASAIGEPLSGRMLLFDALSARIRIVKIRGRSLHCEVCGENAAFTEQMFQDFDYEKFTQSPLTPLPLKLKLLPENVRISAKEYKERVVKGEAHVLVDVRPEHHFKIVSLPKSLNIPLSSLEGKLPEISSALKEEEKCNGPTSSPGASLYVICRRGNDSQRAVQLLHKMGFTSAQDIIGGLESWARDVDPNFPIY

>tr|A0A1U8HD76|A0A1U8HD76_CAPAN/1461 Adenylyltransferase and sulfurtransferase MOCS3 OS=Capsicum annuum OX=4072 GN=LOC107878745 PE=3 SV=1MDSNSVEATRIRQEIDSLKSKKLNIEQQISSLEAQLHQLELNSNTASSLSNGNLGNSNGLSPDMIYRYSRHLLLPSFGVQGQANLLKSSVLVIGAGGLGSPALLYLAACGVGRIGIVDHDIVELNNLQRQIIHTEAYIGKSKVESAADACRSINSSTQIVEHKEAFRTSNALEIVSKYDVVVDATDNAPSRYMINDCCVVLGKPLVSGAALGLEGQLTVYNYNGGPCYRCLFPTPPPTNACQRCADSGVLGVVPGVIGCLQALEAIKVASLVGEPLSGRMLLLDAVSGRFRNVKLRGRSLQCEACGDHAVLTRQTFLDFDYEKFTQTPLSPGPLKLNLLSVDARISSTEYSEKVTEGETHVLVDVRPAHHYKIVSLPNSINIPLSTLEDRLPEISASLEKEAKKDSLDSGSSVSLFVICRRGNDSQRAVELLHKLGFSSAKDIIGGLESWAHNVDPKFPTY

>tr|A0A1U8M0P6|A0A1U8M0P6_GOSHI/1470 Adenylyltransferase and sulfurtransferase MOCS3 OS=Gossypium hirsutum OX=3635 GN=LOC107932786 PE=3 SV=1MESNGGGASPIRREIEALKEEKARIEQRISVLEAQLQEEASLAPQQQQNDGVCNGFCPSEISSVDANLAHGLSADSIYRYSRHLLLPSFGVQAQSNLLKSSILVVGAGGLGSPALLYLAACGVGRLGIVDHDVVELNNMHRQVIHTEAYIGQPKVKSAAAACRSINSTIQIVEHKQALRTSNALEILSQYDIVVDATDNAPSRYMISDCCVVLGKPLVSGAALGLEGQLTVYNYKGGPCYRCLFPTPPPTTACQRCSDSGVLGVVPGIIGCLQALEAIKIASDIGEPLSGRMLLFDALSARIRIVKIRGRSLQCEVCGENTTFNQQQFKDLDYEKFTQSPLSTSPQKLKLLSPDSRITSKEYKERIISGEPHVLVDVRPELHYKIVSIPNSLNIPLASLEARLPEISSALKEQQKESTGSGANLYVICRRGNDSQRAVDYLRNKGFDLAKDIVGGLDSWANDVDPNFPMY

>tr|A0A200RBA8|A0A200RBA8_9MAGN/1466 Adenylyltransferase and sulfurtransferase MOCS3 OS=Macleaya cordata OX=56857 GN=MOCS3 PE=3 SV=1MESNGVHSSLILQEIEKLKAEKKDIENRISVLEAQLNDVVAKEGTVSSSSCPPLSITYLGLEHGLSPEMIYRYSRHLLLPNFGVQGQSNLLKSSILVVGAGGLGSPALLYLAACGVGRLGIVDHDVVELNNLHRQIIHAEAYIGQSKVKSAAAACRAINSSIQIVEHKEALRTSNALEIMSKYDIVVDATDNAPSRYLISDCCVVLGKPLISGAALGLEGQLTVYNHNGGPCYRCLFPTPPPTTACQRCSDSGVLGVVPGVIGCLQALEALKVASAVGEPLSGRMLLFDALSARIRIVKIRGRSLNCEACGENAAFTRQFFQNFDYEKFTQSPLSTTPLKLKLLPETARISSEEYKERVANGEAHVLIDVRPAHHFKIVSLPKALNVPLSTLEAQLSAISSALKDEEERKGLGSGSNASLYVICRRGNDSQRAVDCLHKMGFSSAKDIIGGLESWAQNVDPNFPTY

>tr|A0A218XM71|A0A218XM71_PUNGR/1463 Adenylyltransferase and sulfurtransferase MOCS3 OS=Punica granatum OX=22663 GN=LOC116214207 PE=3 SV=1MDSNGAAGTAIRREIESLNAAKADIESRISALEAQLRDMDHGTGAISSNGSCTLAAPVDLGLEHGLSPDMIYRYSRQLLLPSFGVQAQSNLWKSSILVVGAGGLGSPALLYLAACGVGRLGIVDNDVVELNNMHRQIIHTEAYIGRPKVESAAAACRAVNSTIQIVEHKEALRTSNALEIMSKYDIVVDATDNAPSRYMISDCCVVLGKPLVSGAAIGFEGQLTVYNYKGGPCYRCLFPTPPPTTACQRCSDSGVLGVVPGIVGCLQALEAIKIASEVGEPLSGRMLLLDALSARIRIVKIRGRSLECAACGENTGFTKEQFRIFDYEKFTQSPMLASPLKLNLLPQDSRISSKEYSEIVKNGEAHVLVDVRPAHHFKIVSLPRALNVPLPTLESRVAEISSALKGVEMQDGKGASLYVVCRRGNDSQRAVEYLQKVGFPFAKDIVGGIEGWARDVDPNFPTY

>tr|A0A251UU15|A0A251UU15_HELAN/1466 Adenylyltransferase and sulfurtransferase MOCS3 OS=Helianthus annuus OX=4232 GN=CNX5 PE=3 SV=1MESNGGADESSRLLHELQSLKDSKRDIEARISVIEAQLQQIQSNQHSNNESPAVSSNGVSEFGHDLTPDMIYRYSRQLLLPSFGVQGQSNLLKSSILVIGAGGLGSPALLYLAACGVGKLGMVDHDVVELNNLHRQIIHGEAYIGKSKVESAAAACRSINSTTEIVEHKEALRTSNAIEIVSKYDIVIDATDNAPSRYLISDCCVLLGKPLVSGAALGLEGQLAVYNYNGGPCYRCLFPTPPPTTACQRCSDSGVLGVVPGVIGCLQALEAIKLASLVGEPLSGRMLLFDALSAKVRIVKIRGRSLQCEACGENGLTQQQFQQFDYEKFTQSPLAVAPLKLKLLTSDSRISSKEYDELIKKGSPHVLIDVRPSHHYKITSLPKSINIPLASLESRIPEVSSELKSVQESNGTLNGSDAGLYVVCRRGNDSQRAVQLLHKLGFTYAKDIVGGLESWAREVDPKFPTY

>tr|A0A2G2Z5P6|A0A2G2Z5P6_CAPAN/1461 Adenylyltransferase and sulfurtransferase MOCS3 OS=Capsicum annuum OX=4072 GN=MOCS3 PE=3 SV=1MDSNSVEATRIRQEIDSLKSKKLNIEQQISSLEAQLHQLELNSNTASSLSNGNLGNSNGLSPDMIYRYSRHLLLPSFGVQGQANLLKSSVLVIGAGGLGSPALLYLAACGVGRIGIVDHDIVELNNLQRQIIHTEAYIGKSKVESAADACRSINSSTQIVEHKEAFRTSNALEIVSKYDVVVDATDNAPSRYMINDCCVVLGKPLVSGAALGLEGQLTVYNYNGGPCYRCLFPTPPPTNACQRCADSGVLGVVPGVIGCLQALEAIKVASLVGEPLSGRMLLLDAVSGRFRNVKLRGRSLQCEACGDHAVLTRQTFLDFDYEKFTQTPLSPGPLKLNLLSVDARISSTEYSEKVTEGETHVLVDVRPAHHYKIVSLPNSINIPLSTLEDRLPEISASLEKEAKKDSLDSGSSASLFVICRRGNDSQRAVELLHKLGFSSAKDIIGGLESWAHNVDPKFPTY

>tr|A0A2G9H9P6|A0A2G9H9P6_9LAMI/1466 Adenylyltransferase and sulfurtransferase MOCS3 OS=Handroanthus impetiginosus OX=429701 GN=MOCS3 PE=3 SV=1MAMDSNGAVETAADVRREISSLEKAKAQIEDRISALQARLNSYGLEDGSEKTNCSAPPPAEDLFSGKLSADMIYRYSRHLLLPSFGVDAQANLLKSSVLVIGAGGLGSPALLYLAACGVGSVGIVDNDVVELNNLHRQIIHSEAYIGRSKVESAAAACRGINSTVQIVEHKEAFRTSNALEIMRKYDIVVDATDNVPSRYMISDCCVVLGKPLISGAALGLEGQLTVYNYNGGPCYRCLFPTPPPTTACQRCADSGVLGVVPGIIGCLQALEAIKVAGAVGEPLSGRMLLFDALSARIRVVKIRGRSSQCEACGENATLTEQQFCNFDYEKFTQTPLSTSPLKLQLLPPEARITSKEYNEINKKGEPHILIDVRPAHHYKIISLPNSLNIPLGSLEGGLPEISAALEVNKAGDNSRDSSLYVICRRGNDSQRAVDCLHKMGFTSAKDIIGGLESWAHDVDPKFPTY

>tr|A0A2H5QMQ7|A0A2H5QMQ7_CITUN/1467 Adenylyltransferase and sulfurtransferase MOCS3 OS=Citrus unshiu OX=55188 GN=MOCS3 PE=3 SV=1METNGGSTDVARVLGEIETLKAAKSDIDYRISALEAQLRDTTVSQPQTDTVSNGSYRPSSAVDYGLSPDMIYRYSRHLLLPSFGVEGQSNLLKSSILVIGAGGLGSPALLYLAACGVGRLGIVDHDVVELNNMHRQVIHTEPYIGQSKVKSAAATCRSINSTVHIIEHREALRTSNALEILSQYEIVVDATDNAPSRYMISDCCVVLGKPLVSGAALGLEGQLTVYNYNGGPCYRCLFPTPPPTTACQRCADSGVLGVVPGIIGCLQALEAIKVASAVGEPLSGRMLLFDALSARIRIVKIRGRSSQCEACGENSTFTQDHFRKFDYEKFTQSPLSTLPLKLNLLSADSRISSKEYKEKVVNGEAHILVDVRPAHHFRIVSLPNSINIPLSDLESRLPEISSAMKEKEEHRGSNASSGSNLYVVCRRGNDSQRAVQALHKLGFTSARDIIGGLESWANDVDPSFPVY

>tr|A0A2I0XC13|A0A2I0XC13_9ASPA/1467 Adenylyltransferase and sulfurtransferase MOCS3 OS=Dendrobium catenatum OX=906689 GN=MOCS32 PE=3 SV=1MDYKIVGNGSADIIHEIEKLKLDKKEIENRISKLEAQLKADEAVQMKANQACSSYDSILDGHAVSSNGLTPGMIYRYSRHLLLPDFGVEGQINLAKSSILVVGAGGLGSPVVLYLASCGIGCIGIVDSDIVELNNLHRQIIHPEAYVGHAKVKSAAAACRAINSSINLVEHNQALQASNALDIVSKYDIVVDATDNLPSRYMISDCCVVMNKPLISGAALGLEGQLTVYHHKGGPCYRCLFPTPPPTAACQRCSDSGVLGVVPGVIGCLQALEAIKVASGIGETLSGRMLIFDALSSRIRIVKLRGRSLQCFACGDNADFTQQSFQSFDYEGFTQSPMSDKARPKLSLISDSARITSREYKDLIDKQEPHILVDVRPAHHFKITAIPESINIPLSALEDQISLVDSHLKEVAKDSGKAASLYVVCRRGNDSQRAVDFLNKNGFPLAKDIIGGLESWAQDVDPSFPIY

>tr|A0A2I4EYX8|A0A2I4EYX8_JUGRE/1468 Adenylyltransferase and sulfurtransferase MOCS3 OS=Juglans regia OX=51240 GN=LOC108993979 PE=3 SV=1MESDASRILHELQSLRASKSDIDRRISALETQLHQINLLNDSVPNGSHHSISTIDNSHLNGPHAHYLTPEMIHRYSRHLLLPSFGVQAQSNLLKSSVLVVGAGGLGSPALLYLAACGVGRLGIVDHDVVELNNMHRQIIHTEAFIGQPKVKSAAAACRSVNSTVQIVEHHEALRTSNALGIFSKYDVIVDATDNAPSRYMISDCCVVLGKPLVSGAALGLEGQLTVYNYNGGPCYRCLFPTPPPTTACQRCADSGVLGVVPGIIGCLQALEAIKIASVVGEPLSGRMLLFDALAARIRIVKIRGRSLQCKACGENATFTQQEFQEFDYEKFTQSPLSPSPLKLNLLQADSRISSKEYHDRVVNGDAHVLVDVRPAHHFEIVSLPNSLNIPLPSLEARLPEISSALKEEEERKGVSSGSSAQLYVICRRGNDSQRAVQYLHKTGFTSARDILGGLESWAHDVDPNFPTY

>tr|A0A2J6M5W6|A0A2J6M5W6_LACSA/1460 Adenylyltransferase and sulfurtransferase MOCS3 OS=Lactuca sativa OX=4236 GN=MOCS3 PE=3 SV=1MESNGGVDESTRLLKELQSLKDSKRDIEARISALEGQLRQIQSNQQLNKKASSDCSNGGSEFGHDLTPDMIYRYSRQLLLPSFGVQGQSNLLKSSILVIGAGGLGSPALLYLAACGVGRLGMVDHDVVELNNLHRQIIHGEAYIGRSKVESAAAACRSINSTIEIIEHREALRTSNALEIVSKYDIVIDATDNAPSRYLISDCCVLLGKPLVSGAALGLEGQLAVYNYNGGPCYRCLFPTPPPTTACQRCSDSGVLGVVPGVIGCLQALEAIKVASLVGEPLSGRMLLFDALSARIRIVKIRGRSLQCEACGENATMTQENFQHFDYEKFTQSPLSPAPLKLKLLKEDCRISSKEYEEVVKKGDAHVLIDVRPSQHYKIVSLPNSMNVPLASLEDKLPEIESALKSIDNGNAGVYVVCRRGNDSQRAVELLHNKGFVSAKDIVGGLESWARDVDHRFPTY

>tr|A0A2K3MVR8|A0A2K3MVR8_TRIPR/1452 Adenylyltransferase and sulfurtransferase MOCS3 OS=Trifolium pratense OX=57577 GN=MOCS3 PE=3 SV=1MSSSEILQQLESLKDQKTKIEHKISLLEAQLKDINLHKHNGSSSPYPTNGLEPHMIHRYSRHLVLPSFGVQGQANLLKSSILVVGAGGLGAPALLYFAASGVGKLGIVDHDKVELNNMHRQIIHTEAYIGQPKVKSAAAACRSVNSSIEVVEHEEALRTSNALEIFSKYDIIVDATDNAPTRYMISDCCVVLGKPLVSGAALGLEGQLTVYNHNGGPCYRCLFPTPPPRTACQSCADGGVLGVVPGIIGCLQALEAIKIAASVGEPLSGRMLLLDALSARIRVVKIRGRSMQCEACGENAKFNQQYFREFDYENFTQTPLCVPPLKLNLIPSESRISSKEYNEIIVNKEPHVLVDVRPSHHFKIVSLPNSLNIPFSTLESRLPEISSILKKGEEEKGVVSESSARLYVVCRRGNDSQRAVQYLHKMGFTSAKDIVGGLESWAQNVDPNFPTY

>tr|A0A2N9HQ57|A0A2N9HQ57_FAGSY/1463 Adenylyltransferase and sulfurtransferase MOCS3 OS=Fagus sylvatica OX=28930 GN=MOCS3 PE=3 SV=1MEPNGQDEATRILHELQSLKATKSDIDRRISALESQLHNLTLQNDTVNNLPNPIPTVHPNHHHDLTPQMIHRYSRHLLLPSFGVKAQSNLLNSSILVVGAGGLGSPALLYLAACGVGRLGIVDHDVVELNNMHRQVIHTEGFIGQPKVKSAATTCRSINSTVQIVEHHEALRISNALQIFSQYDVIVDATDNAPSRYMISDCCVLLGKPLVSGAALGLEGQLTVYNYNGGPCYRCLFPTPPPTSACQRCADSGVLGVVPGIIGCLQALEAIKIASAVGEPLSERMLLFDALAARIRIVKIRGRSPQCVVCGENATFTQQQFQEFDYEKFTQSPLTPSPLKLNLLQADSRLSSKEYHDRVVNGEAHVLVDVRPAHHFKIVSLPNSLNIPLPGLETRLPEISSALKKEEERKGSSDAKLYVICRRGNDSQRAAQYLHKMGFTSARDIVGGLESWAHDVDPNFPTY

>tr|A0A2N9IL22|A0A2N9IL22_FAGSY/1463 Adenylyltransferase and sulfurtransferase MOCS3 OS=Fagus sylvatica OX=28930 GN=MOCS3 PE=3 SV=1MEPNGQDEATRILHELQSLKATKSDIDRRISALESQLHNLTLQNDTVNNLPNPIPTVHPNHHHDLTPQMIHRYSRHLLLPSFGVKAQSNLLNSSILVVGAGGLGSPALLYLAACGVGRLGIVDHDVVELNNMHRQVIHTEGFIGQPKVKSAATTCRSINSTVQIVEHHEALRISNALQIFSQYDVIVDATDNAPSRYMISDCCVLLGKPLVSGAALGLEGQLTVYNYNGGPCYRCLFPTPPPTSACQRCADSGVLGVVPGIIGCLQALEAIKIASAVGEPLSERMLLFDALAARIRIVKIRGRSPQCVVCGENATFTQQQFQEFDYEKFTQSPLTPSPLKLNLLQADSRLSSKEYHDRVVNGEAHVLVDVRPAHHFKIVSLPNSLNIPLPGLEAGLPEISSALKKEEERKGSSDAKLYVICRRGNDSQRAAQYLHKMGFTSARDIVGGLESWAHDVDPNFPTY

>tr|A0A2P2KVE2|A0A2P2KVE2_RHIMU/1467 Adenylyltransferase and sulfurtransferase MOCS3 OS=Rhizophora mucronata OX=61149 GN=MOCS3 PE=3 SV=1MEPNVAEADQILVELETLKAVKAEVESRISALEARLRDFNLRNGTVSANGVCPSISTVDSGSGHGLSPDAIYRYSRHLLLPSFGVQGQLNLLKSSILVVGAGGLGSPALLYLAACGVGRLGIVDHDLVELNNMHRQVIHTEAFIGQPKVKSAAAACHSINSTIKIVEHQEALRTSNALEILSQYDIIVDATDNAPSRYMINDCCVVLGKPLVSGAALGLEGQLTVYNYNGGPCYRCLFPAPPSATACQRCADSGVLGVVPGVIGCLQALEAIKIASAVGEPLSGRMLLFDALSARIRVVKIRGRSSECEVCGENATFSQRRFRDFDYEKFTQSPLSTVPLKLNLLPPDSRISSKEFNEKVVNGEPHVLVDVRPAHHFKIVSLPNALNIPLSRLEARLPDILTALKAEEEHRGVASETGTSLYVVCRRGNDSQRAVQLLHNMGFTAAKDIIGGLEAWAHNVDPKFPTY

>tr|A0A2P5AXE2|A0A2P5AXE2_PARAD/1466 Adenylyltransferase and sulfurtransferase MOCS3 OS=Parasponia andersonii OX=3476 GN=MOCS3 PE=3 SV=1MESNGGDASQILRQIETLKATRSDIDRRISALEAQLREINPSHGNDAVLNGSCPPISGFDSGFGHGLSPDNIYRYSRHLLLPSFGVQGQSNLLKSSILVVGAGGLGSPALLYLAACGVGRLGIVDHDVVEVNNMHRQIIHTEEYIGQPKVKSAAAACRSINSTIKIVEHQEALRTSNALEILSQYDIVVDATDNAPSRYMISDCCVVLGKPLVSGAALGLEGQLTVYNYNGGPCYRCLFPTPPPSTACQRCADSGVLGVVPGVIGCLQALEAIKIASAVGEPLSGRMLLFDALSARIRIVKIRGRSLQCEACGESATFTRQQFQEFDYEKFTETPLSPSPLKLNLLQADSRISTKEFKEKIVKGEVHILVDVRPEHHFKIVSLPGSLNIPFRSLEARLPEISSALEEEGNKTKSGSGAQLYVLCRRGNDSQRAVQYLHKMGFTSAKDIIGGLEGWARDVDPNLPMY

>tr|A0A2P5F287|A0A2P5F287_TREOI/1466 Adenylyltransferase and sulfurtransferase MOCS3 OS=Trema orientale OX=63057 GN=MOCS3 PE=3 SV=1MESNGGDASQILRQIETLKATRSDIDRRISALEAQLREINPSHGNDAVLNGSCPPISGFDSGFGHGLSPDNIYRYSRHLLLPSFGVQGQSNLLKSSILVVGAGGLGSPALLYLAACGVGRLGIVDHDVVELNNMHRQIIHTEEYIGQPKVKSAAAACRSINSTIKIVEHQEALRTSNALEILSQYDIVVDATDNAPSRYMISDCCVVLGKPLVSGAALGLEGQLTVYNYNGGPCYRCLFPTPPPSTACQRCADSGVLGVVPGVIGCLQALEAIKIASAIGEPLSGRMLLFDALSARIRIVKIRGRSFQCEACGENATFTRQQFQEFDYEKFTETPLSPSPLKLNLLQADSRISTKEFKEKIVKGEAHLLVDVRPEHHFKIVSLPGSLNIPFRSLEARLPEISSALEEEGNKTNSGSGAQLYVLCRRGNDSQRAVQYLHKMGFTSAKDIIGGLEGWARDVDPNFPMY

>tr|A0A2P6QCQ0|A0A2P6QCQ0_ROSCH/1466 Adenylyltransferase and sulfurtransferase MOCS3 OS=Rosa chinensis OX=74649 GN=MOCS3 PE=3 SV=1MAAADGGEVSAILRDLESLKASKSEIELRISALEARLQEINLQQPNGAVSNGSCAPLPSSVDSGYGHDLSPQMIYRYSRQLLLPSFGVQAQSKLLKSSILVVGAGGLGSPALLYLAACGVGHLGIIDHDVVELNNMHRQIIHTERFIGQPKVRSAAAACRSINSTIQVVEYQEPLRTSNALEIMSKYDIIVDATDNAPSRYMISDCCVVLGKPLVSGAALGFEGQLTVYNYNGGPCYRCLFPTPPPTTACQRCSDSGVLGVVPGIIGCLQALEAIKIAGEVGEPLSGRMLLLDSLSGRIRIVKIRGRSLQCVACGENTTFSKLQFREFDYENFTQSPLTPSPLKLNLLQADSRINTKEYKEKIDDGKAHVLIDVRPEHHFKIVSLPNSLNIPLPSLEARLPEISLALKENEENRGVNSELYVVCRRGNDSQRAVQSLHKLGFTSARDIIGGLEAWARDVDPNFPTY

>tr|A0A371FP71|A0A371FP71_MUCPR/1456 Adenylyltransferase and sulfurtransferase MOCS3 OS=Mucuna pruriens OX=157652 GN=MOCS3 PE=3 SV=1MSASEILRELDSLKDARTKIEQKISALEAQLREINLRNNAATNGSSSSSYPTNGLTQDMIHRYSRHLVLPSFGVQGQANLLKASILVVGAGGLGAPALLYFAASGVGRLGIVDHDVVELNNMHRQIIHTEAYVGKPKVKSAAAACRSVNSSIQVVEHEEALRTSNALEILSKYDIIVDATDNAPTRYMISDCCVVLGKPLVSGAALGLEGQLTVYNYNGGPCYRCLFPTPPPRTACQSCADGGVLGVVPGIIGCLQALEAIKLAASVGEPLSGRMLLLDALSGRIRIVKIRGRSIHCEACGENATFTQQHFREFDYENFTQTPLRVPPLKLNLLPSESRISCKEYSEIILKKEPHVLVDVRPAHHFKIVSMPKSLNIPLSTLEARLPEISSALKKEEGDRGVVSESSAQLYVVCRRGNDSQRAVQCLHKMGFTSAKDIVGGLESWAHNVDPKFPTY

>tr|A0A397XJF7|A0A397XJF7_BRACM/1466 Adenylyltransferase and sulfurtransferase MOCS3 OS=Brassica campestris OX=3711 GN=MOCS3 PE=3 SV=1MAANGGDSSEIVRELKELKLQKAKIEHRISALEAKLQETATVERCDAVSNGCSVTTEIEHGLEHGMSPDQIYRYSRQLLLPSFGVEGQSNLLKSSVLVIGAGGLGSPALLYLAACGVGRLGIIDHDVVELNNMHRQVIHTEAFIGHPKVKSAATACRSINSTIKIDEYVEALRTSNALEILSQYDIIVDATDNPPSRYMISDCCVLLGKPLVSGAALGMEGQLTVYNHNGGPCYRCLFPTPPPTTACQRCSDSGVLGVVPGAIGCLQALETIKLASMVGEPLSERMLLFDALSARIRIVKIRGRSAQCTVCGDNSSFNKQQFKDFDYEEFTQFPLSAGPLNLLPAESRISSKEFKEILQKKERHVLLDVRPSHHYKIVSLPDSLNIPFANLEARLNELTSALKDKEDDHVNTGSTPNPSLYVVCRRGNDSQRAVQYLRDSGFSSAKDIVGGLEAWAADVNPNFPTY

>tr|A0A3P6DAN1|A0A3P6DAN1_BRACM/1466 Adenylyltransferase and sulfurtransferase MOCS3 OS=Brassica campestris OX=3711 GN=MOCS3 PE=3 SV=1MAANGGDSSEIVRELKELKLQKAKIEHRISALEAKLQETAAVEHCNAVSNGCSVPTEVKYGLEHGMSPDQIYRYSRQLLLPSFGVEGQSNLLKSSVLVIGAGGLGSPALLYLAACGVGRLGIIDHDVVELNNMHRQVIHTEAFIGHPKVKSAATACRSINSTIKIDEYVEALRTSNALEILSQYDIIVDATDNPPSRYMISDCCVLLGKPLVSGAALGMEGQLTVYIHNGGPCYRCLFPTPPPTTACQRCSDSGVLGVVPGAIGCLQALETIKLASMVGEPLSERMLLFDALSARIRIVKIRGRSAQCTVCGDNSSFNKQQFKDFDYEEFTQFPLSAGPLNLLPAESRISSKEFKEILQKKERHVLLDVRPSHHYKIVSLPDSLNIPFANLEARLNELTSALKDKEDDHVNTGSTPNPSLYVVCRRGNDSQRAVQYLRDSGFSSAKDIIGGLEAWAADVNPNFPSY

>tr|A0A3P6EDD6|A0A3P6EDD6_BRAOL/1466 Adenylyltransferase and sulfurtransferase MOCS3 OS=Brassica oleracea OX=3712 GN=MOCS3 PE=3 SV=1MEANGGGSSEIVRELKELKLQKAEIEHRISALEAKLQETAAVERCDAVSNGYSVPAEIEHGLEHGMSPDQIYRYSRQLLLPSFGVEGQSNLLKSSVLVIGAGGLGSPALLYLAACGVGRLGIIDHDVVELNNMHRQVIHTEAFIGHPKVKSAATACRSINSTIKIDEYVEALRTSNALEILSQYDIIVDATDNPPSRYMISDCCVLLGKPLVSGAALGMEGQLTVYNHKGGPCYRCLFPTPPPTTACQRCSDSGVLGVVPGAIGCLQALETIKLASMVGEPLSERMLLFDALSARIRIVKIRGRSAQCTVCGDNSSFNKQQFKDFDYEEFTQFPLSAGPLNLLPAESRISSKEFKEIRQKKERHVLLDVRPSHHYKIVSLPDSLNIPFANLEARLNELTSALKEKEDGHVNSGSCANPSLYVVCRRGNDSQRAVQYLRDSGFSSAKDIIGGLEAWAADVNPNFPSY

>tr|A0A3Q7HIG7|A0A3Q7HIG7_SOLLC/1460 Adenylyltransferase and sulfurtransferase MOCS3 OS=Solanum lycopersicum OX=4081 GN=101267426 PE=3 SV=1MDFNGVEATRIRQEIDHLKSKKRNIEQQISALEAQLNQLEVNSSTVCPQPLSNGDLSNSNGLSPDMIYRYSRHLLLPSFGVQGQANLLKSSVLVIGAGGLGSPALLYLAACGVGRMGIVDHDVVELNNLQRQIIHTEAYIGKSKVESAAATCRSINSSTQIVEHREAFRTSNALEIVSKYDVVVDATDNAPSRYMINDCCVVLGKPLVSGAALGLEGQLTVYNYNGGPCYRCLFPTPPPTNACQRCADSGVLGVVPGVIGCLQALEAIKVASLVGEPLSGRMLLLDALSGRFRNVKLRGRSLQCEACGDDAVLTRQTFSEFDYEKFTQTPLSTVPLKLNFLSADDRISTKEYNEKVRKGDAHILVDVRPSHHYKIVSLPNSMNIPLSTLEGRLPEISAALEKEVNKENGSNTSLFVICRRGNDSQVAVELLHKLGFTSAKDIIGGLESWTHNVDPKFPTY

>tr|A0A445C3C6|A0A445C3C6_ARAHY/1462 Adenylyltransferase and sulfurtransferase MOCS3 OS=Arachis hypogaea OX=3818 GN=MOCS3 PE=3 SV=1MSSETQEAEASRIRRQINELKQERDTIDAKISELQSQLNHLHLKNGVVSNGGSSSHSSNALEPHMIYRYSRHLLLPSFGVQGQENLLNASILVVGAGGLGSPALLYLAACGVGRLGIVDHDVVELNNMHRQIIHTEAYIGQPKVKSAAAACRSINSTIQIVEHQEALRTSNALEILSQYDIIVDATDNAPSRYMINDCCVVLGKPLVSGAALGLEGQLTVYNYNGGPCYRCLFPTPPPTTACQRCADSGVLGVVPGIIGCLQALEAIKIAASVGEILSGRMLLFDALSARIRIVKIRGKSMQCEACAENTTFKQQQFQEFDYENFTQTPLSVGPLRLNLLATESRISSKEYSDIVHNKEPHVLLDVRPAHHFKIVSLTKSLNIPLSTLETRLPEISTALKKEEEQKGVASGSSAQLYVICRRGNDSQRAVQYLQKMGYTSAKDIVGGLESWAHNVDPSFPTY

>tr|A0A445C3F4|A0A445C3F4_ARAHY/1464 Adenylyltransferase and sulfurtransferase MOCS3 OS=Arachis hypogaea OX=3818 GN=MOCS3 PE=3 SV=1MSSETQEAEASRIRRQINELKQERDTIDAKISELQSQLNHLHLKNGVVSNGGSSSHSSNALEPHMIYRYSRHLLLPSFGVQGQENLLNASILVVGAGGLGSPALLYLAACGVGRLGIVDHDVVELNNMHRQIIHTEAYIGQPKVKSAAAACRSINSTIQIVEHQEALRTSNALEILSQYPCYNGINATDNAPSRYMINDCCVVLGKPLVSGAALGLEGQLTVYNYNGGPCYRCLFPTPPPTTACQRCADSGVLGVVPGIIGCLQALEAIKIAASVGEILSGRMLLFDALSARIRIVKIRGKSMQCEACAENTTFKQQQFQEFDYENFTQTPLSVGPLRLNLLATESRISSKEYSDIVHNKEPHVLLDVRPAHHFKIVSLTKSLNIPLSTLETRLPEISTALKKEEEQKGVASGSSAQLYVICRRGNDSQRAVQYLQKMGYTSAKDIVGGLESWAHNVDPSFPTY

>tr|A0A445H097|A0A445H097_GLYSO/1457 Adenylyltransferase and sulfurtransferase MOCS3 OS=Glycine soja OX=3848 GN=MOCS3 PE=3 SV=1MSASEILRELDSLKDEKTKIEHKISALEAQLREINLQNDAAPPNASSSSSYPTNGLTQDMIHRYSRHLVLPSFGVQGQANLLKSSILVVGAGGLGAPALLYFAASGVGRLGVIDHDVVELNNMHRQVIHTEAYVGKPKVKSAAAACCSINSTIQVVEHEEALQTSNALEILSKYDIIVDATDNAPTRYLISDCCVVLGKPLVSGAALGLEGQLTVYNYNGGPCYRCLFPTPPPRTACQSCAEGGVLGVVPGIIGCLQALEAIKIAASVGEPLSGRMLLLDALSGRIRIVKIRGRSMQCEACGENATFTQQQFREFDYEKFTQTPLRVPPLKLNLLPRESRISSKEYSEVIIKKGPHVLVDVRPAHHFKIASLPKSLNIPLSTLEARLPEVSSALKKEEEESGAVSGSSAQLYVVCRRGNDSQRAVQYLHKMGFTSAKDIVGGLESWAHNVDHQFPTY

>tr|A0A445LVR8|A0A445LVR8_GLYSO/1447 Adenylyltransferase and sulfurtransferase MOCS3 OS=Glycine soja OX=3848 GN=MOCS3 PE=3 SV=1MSASEIVRELDSLKDEKTIIEQKISALEAQLREINLQNDAAPPNGLTQDMIHRYSRHLVLPSFGVQGQANLLKSSILVVGAGGLGAPALLYFAASGVGRLGVIDHDVVELNNMHRQVIHTEAYVGKPKVKSAAAACRSINSTIQVVEHEEALRTSNALELLSKYDIIVDATDNAPTRYLISDCCVVLGKPLVSGAALGLEGQLTVYNYNGGPCYRCLFPTPPPRTACQSCAEGGVLGVVPGIIGCLQALEAIKIAASVGEPLSGRMLILDALSGRIRIVKIRGRSMQCEACGENATFTQQQFREFDYEKFTQTPLRVPPLKLNLLPSESRISSKEYSEVILKKEPHVLVDVRPAHHFKIVSLPKSLNIPLSTLEARLPEVSSALKKEEEEGGVVSGSSAQLYVVCRRGNDSQRAVQYLHKMGFISAKDIVGGLESWAHNVDPKFPTY

>tr|A0A4D6MM27|A0A4D6MM27_VIGUN/1460 Adenylyltransferase and sulfurtransferase MOCS3 OS=Vigna unguiculata OX=3917 GN=MOCS3 PE=3 SV=1MSASEILRELDSLKDAKAKIEHKISALEAQLREINLRNDAAPTNGSSHPSSYPTNGLTQDMIHRYSRHLMLPSFGVEGQANLLKSSILVVGAGGLGASALLYFAAAGVGRLGIVDHDVVELNNMHRQVIHTEAYVGKPKVKSAGAACRSVNSSIEVVEHQEALRTSNALEILSKYDIIVDATDNAPTRYLISDCCVVLGKPLVSGAALGMEGQLTVYNYKGGPCYRCLFPLPPPRTACQSCAEGGVLGVVPGIIGCLQALEAIKIAAAVGEPLSGRMLLLDALSGRIRIVKIRGRSTHCEACGENATFTQQKFREFDYEKFTETPLRVPPLKLNLLPSESRISSKEYSEIVLKKEPHVLVDVRPAHHFKIVSLPKSLNIPLSTLEGRLPEISSALKKEEKDSGLVSGSGGGAQLYVVCRRGNDSQRAVQCLQKMGFTSAKDIVGGLESWAYNVDPKFPTY

>tr|A0A4P1R870|A0A4P1R870_LUPAN/1453 Adenylyltransferase and sulfurtransferase MOCS3 OS=Lupinus angustifolius OX=3871 GN=MOCS3 PE=3 SV=1MSEIRESDNVCRIMAELQSLKEAKSTIDNKISLLEAELRDLQNDAANNNELSPQMIYRYSRHLLLPNFGVLGQQNLLNSSILVVGAGGLGSPALLYLAACGVGRLGIVDHDIVELNNMHRQIIHTETYIGKPKVKSAASACRSVNSSIRVVEHQEALRTSNALEIFSKYDIIVDATDNAPTRYMISDCCVVLGKPLVSGAALGLEGQLTVYNYNGGPCYRCLFPTPPPTTACQRCADSGVLGVVPGIIGCLQALEAIKIAASVGEPLSGRMLLFDALSARIRIVKIRGRSLQCEACGENSKFTQQQFREFDYENFTQTPLSVSPLKLNLLPSESRISSKEYNEIILKKEPHVLVDVRPAHHFKIVSLPKSLNIPLSTLEARLPEILSALKKEEEDNRGVVSGSSAQLYVVCRRGNDSQRAVQSLQKLGFTYAKDIVGGLESWAHNVDPNFPTY

>tr|A0A4S4DEV1|A0A4S4DEV1_CAMSI/1448 Adenylyltransferase and sulfurtransferase MOCS3 OS=Camellia sinensis var. sinensis OX=542762 GN=MOCS3 PE=3 SV=1MEANGGESSRILREIENLKSAKSEIERQISDLEAQLRDIDEKDETKTNSTRSCPPLSNGDSGFGHGLSPDMIYRYSRQLLLPSFGVQAQSSLLKASILVVGAGGLGSPALLYLAACGVGRLGIVDHDVVELNNLHRQIIHTEAYIGQSKVESAAAACHSWNMLISMTVMLPRYDIVIDATDNAPSRYLISDCCVVLGKPLVSGAAIGLEGQLTVYNYNGGPCYRCLFPTPPPTTACQRCSDSGVLGIVPGIIGCHQALEAIKIASDVGEPLSRRMLLFDALTARIRIVKIRGRSSQCEVCGENATFTAKQFQEFDYEKFTQSPLSMTPLKLDLLPTEARISSKEYKERVVRGEPHALVDVRPAHHFKISSLPNSMNVPLSSLEARLPEISSALKREEEHASLYVVCRRGNDSQRAVDYLHKMGFTSAKDIIGGLESWAHDVDPNFPTY

>tr|A0A565CR52|A0A565CR52_9BRAS/1469 Adenylyltransferase and sulfurtransferase MOCS3 OS=Arabis nemorensis OX=586526 GN=MOCS3 PE=3 SV=1MEANGGDSSEIVRELEALKLRKSEIEHRISTLEAKLQETTAAVEPCDAVSNGYSSPSSPAIEHGLEHGLSPDQIYRYSRQLLLPSFGVEAQSSLLKSSVLVIGAGGLGSPALLYLAACGVGRLGIIDHDVVELNNMHRQIIHTEAFIGHPKVKSAAAACRSVNSTIKIDEHVEALRTSNALEILSQYDIIVDATDNPPSRYMISDCCVLLGKPLVSGAALGMEGQLTVYNHNGGPCYRCLFPTPPPTTACQRCSDSGVLGVVPGVIGCLQALETIKLASMVGEPLSERMLLFDALSARVRVVKIRGRSAQCVVCGDNSSFKKQQFKNFDYEDFTQFPLTAGPLNLLPTESRISSKEFKEILQKKERHVLLDVRPSHHYKIVSLPDSLNIPLANLEARINELTSAVKEKEDGHVNTGSFTNPSLYVVCRRGNDSQIAVQYLRESGFDSAKDIIGGLEAWATDVNPSFPTY

>tr|A0A5A7UQG2|A0A5A7UQG2_CUCME/1467 Adenylyltransferase and sulfurtransferase MOCS3 OS=Cucumis melo var. makuwa OX=1194695 GN=MOCS3 PE=3 SV=1MASTTGESSRILHEIETLKSAKFDLERRISALESQLHNLNQPHNNGVSNASSTSPSTFPHGLSPDMIYRYSRHLLLPSFGVQGQLRLSKSSVLVVGAGGLGSPALLYLAASGVGRLGIVDHDVVELNNMHRQIIHTEAYIGRSKVESAAATCRSINSTVQIVEHKEALRTSNALEIFSKYDIIVDATDNAPSRYMISDCCVVLGKPLVSGAALGLEGQLTVYNYNGGPCYRCLFPTPPPTTACQRCADSGVLGVVPGIIGCLQALEAIKIASAVGDPLSGRMLLFDALAARIRIVKIRGRSVQCEVCGENSEFKAAQFQEFDYEKFTQSPLSTSPLKLKLLEPNSRISAREYRDRLHSNESHVLVDVRPEHHFKIVSLPNSLNIPLASLDGRLEEVVSALKEEEHKHKQKSKSSSNGDVELYVVCRRGNDSQRAVKYLQENGYPSAKDIIGGLEGWAQEVDPTFPSY

>tr|A0A5B6U6A3|A0A5B6U6A3_9ROSI/1469 Adenylyltransferase and sulfurtransferase MOCS3 OS=Gossypium australe OX=47621 GN=MOCS3 PE=3 SV=1MESNGGGALPIRREIEALKEEKARIEQRISVLQAQLQEEASLAPQQQNDGVCNGFCPSEISTVDANLAHGLSADSIYRYSRHLLLPSFGVKAQSNLLKSSILVVGAGGLGSPALLYLAACGVGRLGIVDHDVVELNNMHRQVIHTEAYIGQPKVKSAAAACRSVNSTIQIVEHKQALRTSNALEILSQYDIVVDATDNAPSRYMISDCCVVLGKPLVSGAALGLEGQLTVYNYKGGPCYRCLFPTPPPTTACQRCSDSGVLGVVPGIIGCLQALEAIKIASDIGEPLSGRMLLFDALSARIRIVKIRGRSLQCEVCGENTTFNQQQFKDLDYEKFTQSPLSTSPPKLKLLSPDSRITSKEYKERIISGEPHVLVDVRPELHYKIVSIPNSLNIPLASLEARLPEISSALKEQEKEGTGLGAHLYVICRRGNDSQRAVDYLRNKGFDLAKDIVGGLESWANDVDSNFPMY

>tr|A0A5B7BD30|A0A5B7BD30_DAVIN/1466 Adenylyltransferase and sulfurtransferase MOCS3 OS=Davidia involucrata OX=16924 GN=MOCS3 PE=3 SV=1MDSNGGDSHRILREIESLKAAKSDIDHRISVLEAQLRDIHENDGTNSTSLCSPISNGDSGFGHGLSPDMIYRYSRHLLLPSFGVQGQSSLLKSSILVVGAGGLGSPALLYLAACGVGRLGIVDHDVVELNNLHRQIIHAEAYIGRSKVESAASACRSINSSIQIVEHKEALRTSNALEIFSKYDIIIDATDNAPSRYMISDCCVVLGKPLVSGAALGSEGQLAVYNYNGGPCYRCLFPTPPPTTACQRCSDSGVLGVVPGIIGCLQALEAIKVASAVGEPLSGRMLLFDALSARIRIVKIRGRSLHCDVCGENAAFNQQLFRDFDYEKFTQSPLSTAPLKLNLLPADARISSKEYKQRVVNGEAHVLVDVRPAHHFNIVSLPASMNFPLSSLEGSLPDISSALKKEEERRGTDTGSGASLYVICRRGNDSQRAVQYLHKMGFSSAKDIIGGLQSWAHDVDPNFPTY

>tr|A0A5D2L6L2|A0A5D2L6L2_GOSTO/1470 Adenylyltransferase and sulfurtransferase MOCS3 OS=Gossypium tomentosum OX=34277 GN=MOCS3 PE=3 SV=1MESNGGGASPIRREIETLKEERARIEQRISFLEAQLQEEASLAPQQQQNDGVCNGFCPSETSTVDANLAHGLSADSIYRYSRHLLLPSFGVKAQSNLLKSSILVVGAGGLGSPALLYLAACGVGRLGIVDHDVVELNNMHRQVIHTEAYIGQPKVKSAAAACRSINSTIQIVEHKQALRTSNALEILSQYDIVVDATDNAPSRYMISDCCVVLGKPLVSGAALGLEGQLTVYNYKGGPCYRCLFPTPPPTTACQRCSDSGVLGVVPGIIGCLQALEAIKIASDIGEPLSGRMLLFDALSARIRIVKIRGRSLQCEVCGENTTFNQQQFKELDYEKFTQSPLSTSPPKLKLLAPDSRITSKEYKERIISGEPHVLVDVRPELHYKIVSMPNSLNIPLASLETRLPEISSALKEQEKEGTRSGANLYVICRRGNDSQRAVDYLRNKGFDLAKDIVGGLESWANDVDPNFPMY

>tr|A0A5D2QWN2|A0A5D2QWN2_GOSTO/1470 Adenylyltransferase and sulfurtransferase MOCS3 OS=Gossypium tomentosum OX=34277 GN=MOCS3 PE=3 SV=1MDSNGGGASPIRREIEALKEEKARIEQRISVLEAQLQEEASLAPQQQQNDGVCNGFCPSEISSVDANLAHGLSADSIYRYSRHLLLPSFGVQAQSNLLKSSILVVGAGGLGSPALLYLAACGVGRLGIVDHDVVELNNMHRQVIHTEAYIGQPKVKSAAAACRSINSTIQIVEHKQALRTSNALEILSQYDIVVDATDNAPSRYMISDCCVVLGKPLVSGAALGLEGQLTVYNYKGGPCYRCLFPTPPPTTACQRCSDSGVLGVVPGIIGCLQALEAIKIASDIGEPLSGRMLLFDALSARIRIVKIRGRSLQCEVCGENTTFNQQQFKDLDYEKFTQSPLSTSPQKLKLLSPDSRITSKEYKERIISGEPHVLVDVRPELHYKIVSIPNSLNIPLASLEARLPEISSALKEQQKESTGSGANLYVICRRGNDSQRAVDYLRNKGFDLAKDIVGGLDSWANDVDPNFPMY

>tr|A0A5D2V7E5|A0A5D2V7E5_GOSMU/1470 Adenylyltransferase and sulfurtransferase MOCS3 OS=Gossypium mustelinum OX=34275 GN=MOCS3 PE=3 SV=1MESNGGGASPIRREIETLKEERALIEQRISFLEAQLQEEASLAPQQQQNDGVCNGFCPSEISTVDANLAHGLSADSIYRYSRHLLLPSFGVKAQSNLLKSSILVVGAGGLGSPALLYLAACGVGRLGIVDHDVVELNNMHRQVIHTEAYIGQPKVKSAAAACCSINSTIQIVEHKQALRTSNALEILSQYDIVVDATDNAPSRYMISDCCVVLGKPLVSGAALGLEGQLTVYNYKGGPCYRCLFPTPPPTTACQRCSDSGVLGVVPGIIGCLQALEAIKIASDIGEPVSGRMLLFDALSARIRIVKIRGRSLQCEVCGENTTFNQQQFKELDYEKFTQSPLSTSPPKLKLLAPDSRITSKEYKERIISGEPHVLVDVRPELHYKIVSMPNSLNIPLASLETRLPEISSALKEQEKEGTRLGANLYVICRRGNDSQRAVDYLRNKGFDLAKDIVGGLESWANDVDPNFPMY

>tr|A0A5D2ZJP0|A0A5D2ZJP0_GOSMU/1470 Adenylyltransferase and sulfurtransferase MOCS3 OS=Gossypium mustelinum OX=34275 GN=MOCS3 PE=3 SV=1MESNGGGASPIRREIEALKEEKARIEQRISVLEAQLQEEASLAPQQQQNDGVCNGFCPSEISSVDANLAHGLSADSIYRYSRHLLLPSFGVQAQSNLLKSSILVVGAGGLGSPALLYLAACGVGRLGIVDHDVVELNNMHRQVIHTEAYIGQPKVKSAAAACRSINSTIQIVEHKQALRTSNALEILSQYDIVVDATDNAPSRYMISDCCVVLGKPLVSGAALGLEGQLTVYNYKGGPCYRCLFPTPPPTTACQRCSDSGVLGVVPGIIGCLQALEAIKIASDIGEPLSGRMLLFDALSARIRIVKIRGRSLQCEVCGENTTFNQQQFKDLDYEKFTQSPLSTSPQKLKLLSPDSRITSKEYKERIISGEPHVLVDVRPELHYKIVSIPNSLNIPLASLEARLPEISSALKEQEKESTGSGANLYVICRRGNDSQRAVDYLRNKGFDLAKDIVGGLDSWANDVDPNFPMY

>tr|A0A5J5A0B5|A0A5J5A0B5_9ASTE/1466 Adenylyltransferase and sulfurtransferase MOCS3 OS=Nyssa sinensis OX=561372 GN=MOCS3 PE=3 SV=1MESNGGDSHRILREIESLKAAKSNIDHRISVLEAQLRDIHIKDGTNSTSSRSLVSNGDSGFGHGLSPDMIYRYSRHLLLPSFGVQGQSGLLKSSILVVGAGGLGSPALLYLAACGVGRLGIVDHDVVELNNLHRQIIHTEAYIGQSKVKSAAAACHSINSSIQIVEHKEALRTSNALEILSKYDIIIDATDNAPSRYMISDCCVVLGKPLVSGAALGSEGQLTVYNYNGGPCYRCLFPTPPPTTACQRCSDSGVLGVVPGIIGCLQALEAIKIASVVGEPLSGRMLLFDAFSARIRIVKIRGRSLHCEVCGESAAFNRQLFQDFDYEKFTQSPLSTAPLKLNLLAADARISSKEYKERVVNGEAHVLVDVRPALHFNIVSLPNSMNIPLSSLEGSLPEISRALKKEEERRGIDAGSGASLYVICRRGNDSQTAVQHLHKMGHTSARDIIGGLQSWAHDVDPNFPAY

>tr|A0A5J5A2J2|A0A5J5A2J2_9ASTE/1466 Adenylyltransferase and sulfurtransferase MOCS3 OS=Nyssa sinensis OX=561372 GN=MOCS3 PE=3 SV=1MESNGGDSHQILREIESLKAAKSDIEHRISVLEAQLRDIHIKDETNSTILRSPLSNGDSGFGHGLSPDMIYRYSRHLLLPSFGVQGQSGLLKSSILVVGAGGLGSPALLYLAACGVGRLGIVDHDVVELNNLHRQIIHAEAYIGRSKVESAAAACRSINSSIQIVEHKEALRTSNALEILSKYDIIIDATDNAPSRYMISDCCVVLGKPLVSGAALGSEGQLTVYNYNGGPCYRCLFPTPPPTTACQRCSDSGVLGVVPGIIGCLQALEAIKIASAVGEPLSGRMLLFDALSARIRIVKIRGRSLHCEVCGESAAFNRQLFQDFDYEKFTQSPLSTAPLKLNLLAADARISSKEYKERVVNGEAHVLVDVRPALHFNIVSLPNSMNIPLSSLEGSLPEISRALKKEEESRGIDAGSGASLYVICRRGNDSQRAVQLLHKMGHTSAKDIIGGLQSWAHDVDPNFPAY

>tr|A0A5J5RRJ7|A0A5J5RRJ7_GOSBA/1470 Adenylyltransferase and sulfurtransferase MOCS3 OS=Gossypium barbadense OX=3634 GN=MOCS3 PE=3 SV=1MESNGGGASPIRREIETLKEERARIEQRISFLEAQLQEEASLAPQQQQNDGVCNGFCPSETSTVDANLAHGLSADSIHRYSRHLLLPSFGVKAQSNLLKSSILVVGAGGLGSPALLYLAACGVGRLGIVDHDVVELNNMHRQVIHTEAYIGQPKVKSAAAACRSINSTIQIVEHKQALRTSNALEILSQYDIVVDATDNAPSRYMISDCCVVLGKPLVSGAALGLEGQLTVYNYKGGPCYRCLFPTPPPTTACQRCSDSGVLGVVPGIIGCLQALEAIKIASDIGEPLSGRMLLFDALSARIRIVKIRGRSLQCEVCGENTTFNQQQFKELDYEKFTQSPLSTSPPKLKLLAPDSRITSKEYKERIISGEPHVLVDVRPELHYKIVSMPNSLNIPLASLETRLPEISSALKEQEKEGTRSGANLYVICRRGNDSQRAVDYLRNKGFDLAKDIVGGLESWANDVDPNFPMY

>tr|A0A5J5W357|A0A5J5W357_GOSBA/1470 Adenylyltransferase and sulfurtransferase MOCS3 OS=Gossypium barbadense OX=3634 GN=MOCS3 PE=3 SV=1MESNGGGASPIRREIEALKEEKARIEQRISVLEAQLQEEASLAPQQQQNDGVCNGFCPSEISSVDANLAHGLSADSIYRYSRHLLLPSFGVQAQSNLLKSSILVVGAGGLGSPALLYLAACGVGRLGIVDHDVVELNNMHRQVIHTEAYIGQPKVKSAAAACRSINSTIQIVEHKQALRTSNALEILSQYDIVVDATDNAPSRYMISDCCVVLGKPLVSGAALGLEGQLTVYNYKGGPCYRCLFPTPPPTTACQRCSDSGVLGVVPGIIGCLQALEAIKIASDIGEPLSGRMLLFDALSARIRIVKIRGRSLQCEVCGENTTFNQQQFKDLDYEKFTQSPLSTSPQKLKLLSPDSRITSKEYKERIISGEPHVLVDVRPELHYKIVSIPNSLNIPLASLEARLPEISSALKEQEKESTGSEANLYVICRRGNDSQRAVDYLRNKGFDLAKDIVGGLDSWANDVDPNFPIY

>tr|A0A5P1FM27|A0A5P1FM27_ASPOF/1456 Adenylyltransferase and sulfurtransferase MOCS3 OS=Asparagus officinalis OX=4686 GN=MOCS3 PE=3 SV=1MDNGNPNEIVNEIKRLKAEKEELEKRISLLEAKLSDEKADSSSTSNGEIAVPDTGNGLTPEMIYRYSRHLILPDFGVEGQRNLSKSSILVVGAGGLGSPIAMYLAACGVGCLGIVDNDKVELNNLHRQIIHTEAYVGQSKVKSAAVSCHAINSSIKLVEHNEALKAANALDIVSKYDIVVDATDNLPSRYMISDCCVLLDKPLISGAALGLEGQLTVYHHNGGPCYRCLFPTPPPTAACQRCSDSGVLGVVPGVIGCLQALEAIKVAGAVGEPLSGRMLLFDALSSRIRIVKIRGRSLQCIACGENATFTQENFRRFDYESFTQSPISDTSRPKLSLLPESARISSHEYKNIVDKGEPHILVDVRPAHHFKISAIPESINIPLSMLEQKMSTLDLVLREARQASSRPASLYVVCRRGNDSQRAVKFLSENGFASAKDIVGGLESWAKDVDPTFPTY

>tr|A0A5S9YE65|A0A5S9YE65_ARATH/1464 Adenylyltransferase and sulfurtransferase MOCS3 OS=Arabidopsis thaliana OX=3702 GN=MOCS3 PE=3 SV=1MMSNGGDSSEIVRELEELKLKKAEIEHRISTLEAKLQDTAAVELYDAVSNGDSYLTAPELEHGLSPDQIYRYSRQLLLPSFAVEGQSNLLKSSVLVIGAGGLGSPALLYLAACGVGQLGIIDHDVVELNNMHRQIIHTEAFIGHPKVKSAAAACRSINSTIKVDEYVEALRTSNALEILSQYDIIVDATDNPPSRYMISDCCVLLGKPLVSGAALGMEGQLTVYNHNGGPCYRCLFPTPPPTSACQRCSDSGVLGVVPGVIGCLQALETIKLASLVGEPLSERMLLFDALSARMRIVKIRGRSSQCTVCGDNSSFNKQTFKDFDYEDFTQFPLFAGPLNLLPAESRISSKEFKEILQKKEQHVLLDVRPSHHYKIVSLPDSLNIPLANLETRLNELTSALKEKGNGHANTESCTNPSVFVVCRRGNDSQRAVQYLRESGFDSAKDIIGGLEAWAANVNPNFPTY

>tr|A0A654GB18|A0A654GB18_ARATH/1464 Adenylyltransferase and sulfurtransferase MOCS3 OS=Arabidopsis thaliana OX=3702 GN=MOCS3 PE=3 SV=1MMSNGGDSSEIVRELEELKLKKAEIEHRISTLEAKLQDTAAVELYDAVSNGDSYLTAPELEHGLSPDQIYRYSRQLLLPSFAVEGQSNLLKSSVLVIGAGGLGSPALLYLAACGVGQLGIIDHDVVELNNMHRQIIHTEAFIGHPKVKSAAAACRSINSTIKVDEYVEALRTSNALEILSQYDIIVDATDNPPSRYMISDCCVLLGKPLVSGAALGMEGQLTVYNHNGGPCYRCLFPTPPPTSACQRCSDSGVLGVVPGVIGCLQALETIKLASLVGEPLSERMLLFDALSARMRIVKIRGRSSQCTVCGDNSSFNKQTFKDFDYEDFTQFPLFAGPLNLLPAESRISSKEFKEILQKKEQHVLLDVRPSHHYKIVSLPDSLNIPLANLETRLNELTSALKEKGNGHANTESCANPSVFVVCRRGNDSQRAVQYLRESGFDSAKDIIGGLEAWAANVNPNFPTY

>tr|A0A6A1VQI2|A0A6A1VQI2_9ROSI/1467 Adenylyltransferase and sulfurtransferase MOCS3 OS=Morella rubra OX=262757 GN=MOCS3 PE=3 SV=1MESNASQILHELESLRAAKADIDRRISALEAQLQQVTLQNDAIPNGSYPPISTVGSSHLNGHDHHLTPAMIHRYSRHLLLPSFGVQAQSNLLKSSVLVVGAGGLGSPALLYLAACGVGRLGIVDHDVVELNNMHRQIIHTETFIGRPKVKSAANACRSVNSTVEIVEHHEALRTSNALEIFNKYDVIVDATDNAPSRYMISDCCVLLGKPLVSGAALGLEGQLTVYNYNGGPCYRCLFPTPPPATACQRCADSGVLGVVPGIIGCLQALEAIKIASAIGEPLSGRMLLFNALEARIRIVKIRGRSLQCEACKENATFTQQQFREFDYEKFTQSPLAPSPLKLKLLQADSRISSKEYHERVANGEAHVLVDVRPAHHFKIVSLPKSLNIPLPSLEARFPEITSALKEEEEQKSVGSGSSAQLYVICRRGNDSQRAVQCLHKMGFTSARDIVGGLESWTHDVDANFPTY

>tr|A0A6A3BDR4|A0A6A3BDR4_HIBSY/1468 Adenylyltransferase and sulfurtransferase MOCS3 OS=Hibiscus syriacus OX=106335 GN=MOCS3 PE=3 SV=1MESSGGNASRIRREIEALKDEKARIEQKILELENQLREEVSLAPQQQENESVCDGCCPSLSAADANLAHGLTAGSIYRYSRHLLLPSFGVQAQSNLLKSSILVVGAGGLGSPALLYLAASGVGRLGIVDHDVVELNNMHRQVIHTETYIGQPKVKSAAAACQSINSTIQIVEHEQALRTSNALEIMSQYDIVVDATDNAPSRYMISDCCVVLGKPLVSGAALGLEGQLTVYNYKGGPCYRCLFPMPPPATACQRCSDSGVLGVVPGIIGCLQALEAIKIASDVGEPLSGQMLLFDALSARIRSVKIRGRSPQCEVCGENTTFNQQRFKDFDYEKFTQSPLSTSPQKLKLLTPESRITSKEYKERVVSGEPHVLVDVRPEHHYKIVSLPKSLNIPLTSLEARVPEISSALKGQEEGGGSGANLYVICRRGNDSQRAVECLQNMGFDLAKDIVGGLESWANDVDPNFPMY

>tr|A0A6A5LY20|A0A6A5LY20_LUPAL/1453 Adenylyltransferase and sulfurtransferase MOCS3 OS=Lupinus albus OX=3870 GN=MOCS3 PE=3 SV=1MCEIRQSENASRIMAELQSLKEAKSTIENKICLLEAELRDLKNDAAPNKGLSPEMIYRYSRHLLLPNFGVSGQENLFNSSILVVGAGGLGSPALLYLAASGVGRLGIVDHDLVELNNMHRQIIHTESYIGQPKVKSAASACRSLNSSIQVVRHQEALRTSNALEILTKYDIIVDATDNAPTRYLISDCCVVLGKPLVSGAALGLEGQLTVYNHNGGPCYRCLFPTPPPTTACQRCADSGVLGVVPGIIGCLQALEAIKIATSVGEPLSGRMLLFDALSARIRIVKIRGRSLQCEACGENSKFTQQQFREFDYENFTQTPLSVSPLKLKLLPTESRISSKEYSEIIIKKEPHVLVDVRPAHHFKIVSLPKSLNIPLSNLEARLPEISSALKKEEEDNKSVVSGSSAQLYVVCRRGNDSQRAVQSLQKMGFTSAKDIIGGLESWAHNVDPNFPTY

>tr|A0A6D2I079|A0A6D2I079_9BRAS/1465 Adenylyltransferase and sulfurtransferase MOCS3 OS=Microthlaspi erraticum OX=1685480 GN=MOCS3 PE=3 SV=1MEANGGDSSEIVRELEELKLRKAEIEHRISTLEAKLHETAAVERLDAAVSNGYSSPAAIEHGLDHGLSPDQIYRYSRQLLLPSFGVEAQSNLLKSSVLVIGAGGLGSPALLYLAACGVGRLGIIDHDVVELNNMHRQIIHTEAFIGHPKVKSAASACRSVNSTIKIDEHVEALRTSNALEILSQYDIIVDATDNPPSRYMISDCCVLLGKPLVSGAALGMEGQLTVYNHNGGPCYRCLFPTPPPTSACQRCSDSGVLGVVPGVIGCLQALETIKLASMVGEPLSERMLLFDALSARIRIVKIRGRSSQCTVCGDNSSFDKHQFKNFDYEDFTQFPLFAGSLKLLPKESRISSEEFKEILQKKERHILLDVRPSHHYKIVSLPDSLNIPLANLESRLNELTSVLKEKEDVNAETGPCTSLYVVCRRGNDSQRAVQYLRESGFDSAKDIIGGLEAWAADVNPNFPTY

>tr|A0A6G1BW01|A0A6G1BW01_9ORYZ/1470 Adenylyltransferase and sulfurtransferase MOCS3 OS=Oryza meyeriana var. granulata OX=110450 GN=MOCS3 PE=3 SV=1MEAIAGELARLRAEREELDTCIRLLESQLEAGRAGEGDVAGTGAGGGSWGGTAACPIRRRGNGFAPADGLPADMIYRYSRHLLLPDFGVEGQRKLSRSSVLVVGAGGLGSPVALYLAACGVGCLGIVDGDDVELNNLHRQIIHKEAYVGKSKVKSAADTCREINSSIKVVEYCHTLKPCNALEIVRNYDIVVDATDNLPTRYMISDCCVLLNKPLISGAALGLEGQLTVYHHNGSPCYRCLFPNPPPVAACQRCSDSGVLGVVPGVIGCLQALEAIKVATDVGEPLSGRMLLFDALSACIRIVKIRGSSTVCTVCGENSTFTQDDFQKFDYENFTQSPMSDKSAPSLDLLPESARVTCKDYKRLVDNGEPHLLLDVRPAHHFQIASVSQSLNIPLSVLEEKLQILETSLKETMDGSASDKPPSLYVVCRRGNDSQIAVQLLREKGFLSAKDIIGGLQSWAQDVDPDFPVY

>tr|A0A6I9TZW6|A0A6I9TZW6_SESIN/1467 Adenylyltransferase and sulfurtransferase MOCS3 OS=Sesamum indicum OX=4182 GN=LOC105170538 PE=3 SV=1MDSNGVAETPADIRREIASLEKVKAEIDHRISALRVRLTSYGSVNGNEVANSSAPRPEQTLLSGQLSPDMIYRYSRHLLLPSFGVEAQANLLKCSVLVIGAGGLGSPALLYLAACGIGCVGIVDHDVVELNNLHRQIIHTEAYIGRSKVESAAAACRAINSSVQIVEHREAFRTSNALEIVRKYDIVVDATDNVPSRYMISDCCVVLGKPLISGAALGLEGQLTVYNYKGGPCYRCLFPTPPPTTACQRCADSGVLGVVPGVIGCLQALEAIKVAGAVGEPLSGRMLLLDALSARIRVVKIRGRSPQCEACGENAKLTEEQFCNFDYEKFTQSPLSTSPLKLHLLPSEARITSKEYNERNLKGEAHVLVDVRPSHHYKIISLPKSMNIPLASLEARLSEVSAALAREEGHKKDDDNQSASLYVICRRGNDSQRAVQYLHKMGFASAKDIIGGLESWAHDVDPSFPTY

>tr|A0A6J0LC24|A0A6J0LC24_RAPSA/1464 Adenylyltransferase and sulfurtransferase MOCS3 OS=Raphanus sativus OX=3726 GN=LOC108828121 PE=3 SV=1MEANGGEIVRELKELKLQKAEIEQRISALEAKLHETTAAVERCDAVSNGCSVPTEIEHGLEHGMSPDQIYRYSRQLLLPSFGVEGQSNLLKSSVLVIGAGGLGSPALLYLAACGVGRLGIIDHDVVELNNMHRQVIHTEAFIGHPKVKSAATACRSINSTIKIDEYVEALRTSNALEILSQYDIIVDATDNPPSRYMISDCCVLLGKPLVSGAALGMEGQLTVYNHKGGPCYRCLFPTPPPTTACQRCSDSGVLGVVPGAIGCLQALETIKLASMVGEPLSERMLLFDALSARIRIVKIRGRSAQCTVCGDNSSFNKQQFKDFDYEEFTQFPLSADPLNLLPAESRISSKEFKEILQKKERHVLLDVRPSHHYKIVSLPDSLNIPLANLEARLNELTSALKEKEYGCVNTGSSTNASLYVVCRRGNDSQRAVQYLRDWGFSSAKDIIGGLEAWTADVNPNFPSY

>tr|A0A6J1AMA1|A0A6J1AMA1_9ROSI/1462 Adenylyltransferase and sulfurtransferase MOCS3 OS=Herrania umbratica OX=108875 GN=LOC110419390 PE=3 SV=1MEPNGDDVARIRREIEALKDTKASIEHKIAVLEAQLQQHQNEDVCNGSCPPISAVDANLANGLSADSVYRYSRHLLLPSFGVEAQSNLLKSSILVVGAGGLGSPALLYLAACGVGRLGIVDHDVVELNNMHRQVIHTEAYIGQPKVKSAAAACRTINSTIQIIEHKEALRTSNALEILSQYDVIIDATDNAPSRYMISDCCVVLGKSLVSGAALGLEGQLTVYNYKGGPCYRCLFPTPPPTTACQRCSDSGVLGVVPGIIGCLQALEAIKIASAVGEPLSGRMLLFDALSARIRIVKIRGRSLQCEVCRENATFDQQYFRDFDYEKFTQSPLSTSPPKLNLLAADSRITSNEYKERIINGEAHVLVDVRPEHHYRIVSMPKSLNIPLASLEARLPEVSSALKEEQECDGTVSGANLYVICRRGNDSQRAVQYLHKMGFDLAKDIVGGLESWAHDVDPNFPMY

>tr|A0A6J1E0P9|A0A6J1E0P9_MOMCH/1465 Adenylyltransferase and sulfurtransferase MOCS3 OS=Momordica charantia OX=3673 GN=LOC111025340 PE=3 SV=1MGLTSTESSRILQDIETLKSAKSDLERRISALESQLRDLNHHQNDGVSNGSCTSLSTVESGFPHGLSPDMIYRYSRHLLLPSFGVQGQSRLLKSSILVVGAGGLGSPALLYLAACGVGRLGIVDHDVVELNNMHRQIIHTEAYIGRSKVESAAATCHSINSTAQIIEHKEALRTSNALEIFSKYDIIVDATDNAPSRYMISDCCVVLGKPLVSGAALGLEGQLTVYNYNGGPCYRCLFPTPPPTTACQRCADSGVLGVVPGIIGCLQALEAIKIASAVGEPLSGRMLLFDALAARIRIVKIRGRSLQCEICGENAKFNAEQFREFDYEKFTQSPLSTSPLRLKLLEPHSRIGAKEYRDRLGNGEAHVLVDVRPEHHFKIVSLPNSLNVPLGSLEGRLDEIASALKEHSDSSSSSDGVQLYVVCRRGNDSQRAVKYLQEQGFDSAKDIIGGLEGWAHEVDPTFPTY

>tr|A0A6N2BV47|A0A6N2BV47_SOLCI/1460 Adenylyltransferase and sulfurtransferase MOCS3 OS=Solanum chilense OX=4083 GN=MOCS3 PE=3 SV=1MDSNGVEAARIRQEIDHLKSKKRNIEQQISALEAQLNQLEVNSSAVCPQPLPNGDLSNSNGLSPDMIYRYSRHLLLPSFGVQGQENLLKSSVLVIGAGGLGSPALLYLAACGVGRIGIVDHDVVELNNLQRQIIHTEAYIGKSKVESAAATCRSINSSTQIVEHREAFRTSNALEIVSKYDVVVDATDNAPSRYMINDCCVVLGKPLVSGAALGLEGQLTVYNYNGGPCYRCLFPTPPPTNACQRCADSGVLGVVPGVIGCLQALEAIKVASLVGEPLSGRMLLLDALSGRFRNVKLRGRSLQCEACGDDAVLTRQTFSEFDYEKFTQTPLSTVPLKLNFLSADDRISTKEYNEKVRKGDAHILVDVRPSHHYKIVSLPNSMNIPLSTLEGRLPEISAALEKEVNKENGSNASLFVICRRGNDSQVAVELLHKLGFTSAKDIIGGLESWTHNVDPKFPTY

>tr|A0A6P4BAK5|A0A6P4BAK5_ZIZJJ/1466 Adenylyltransferase and sulfurtransferase MOCS3 OS=Ziziphus jujuba OX=326968 GN=LOC107432745 PE=3 SV=1MESNGGDASRILREIETLKATKTEIEQRISALEAQLRETNLDDRTGTVLNGSCPPISTVHSDFGHSLSPEMIYRYSRHLLLPSFGVQGQSNLIKSSILVVGAGGLGSPALLYLAACGVGRLGIVDHDVVELNNMHRQIIHREEYIGQPKVKSAAASCLSINSTINIVEHKEALRTSNALDILSQYDIIVDATDNAPSRYLISDCCVVLGKPLVSGAALGMEGQLTVYNYNGGPCYRCLFPTPPPTTACQRCSDSGVLGVVPGIIGCLQALEAIKIASAVGEPLTGRMILFDALSARIRIVKIRGRSLQCEVCGENATFTRQQFQEFDYEKFTQSPLSTLPVTLNLLQADSRISSKEFKEKIAKGEAHVLIDVRPQHHFKIVSLPGSLNIPLPSLEARLSEISATLEDNEKGKYSGSGPQIFVVCRRGNDSQRAVQYLHKVGFTSAKDIIGGLEGWARDVDPDFPAY

>tr|A0A6P4DS10|A0A6P4DS10_ARADU/1462 Adenylyltransferase and sulfurtransferase MOCS3 OS=Arachis duranensis OX=130453 GN=LOC107496349 PE=3 SV=1MSSETQEAEASRIRRQINELKQERDTIDAKISELQSQLNHLHLKNGVVSNGGSSSHSSNALEPHMIYRYSRHLLLPSFGVQGQENLLNASILVVGAGGLGSPALLYLAACGVGRLGIVDHDVVELNNMHRQIIHTEAYIGQPKVKSAAAACRSINSTIQIVEHQEALRTSNALEILSQYDIIVDATDNAPSRYMINDCCVVLGKPLVSGAALGLEGQLTVYNYNGGPCYRCLFPTPPPTTACQRCADSGVLGVVPGIIGCLQALEAIKIAASVGEILSGRMLLFDALSARIRIVKIRGRSMQCEACAENTTFKQQQFQEFDYENFTQTPLSVGPLRLNLLATESRISSKEYSDIVHNKEPHVLLDVRPAHHFKIVSLTKSLNIPLSTLETRLPEISTALKKEEEQKGVASGSSAQLYVICRRGNDSQRAVQYLQKMGYTSAKDIVGGLESWAHNVDPSFPTY

>tr|A0A6P5GSU7|A0A6P5GSU7_ANACO/1453 Adenylyltransferase and sulfurtransferase MOCS3 OS=Ananas comosus OX=4615 GN=LOC109726540 PE=3 SV=1MDAGGFSGASADSAAVEINRLRAEKEALEGRIRALEAQLENAPPAGEETPANGVSGCRLSPEMIFRYSRHLLLPDFGVGGQRMLSQSSILVVGAGGLGSPVVLYLAACGVGCLGIVDSDVVELNNLHRQIIHTEAYVGQSKVKSAAASCRAINSSVKVVEHQEALQAKNALDIVEKYDIVVDATDNLPSRYMISDCCVLLNKPLISGAALGLEGQLTVYNHNGSPCYRCLFPTPPPTAACQRCSDSGVLGVVPGVIGCLQALEAIKVAASVGEPLCGRMLLFDALSSRIRTVKIRGRSLYCSICGDNSVFTKEEFQTFDYESFTQSPMSDKSRPKLSLLPDDARITSKEYKEIVDKGESHMLLDVRPAHHFRITSIPHSVNIPLSVLEGEFSKLDSALTSSGEGSALYVVCRRGNDSQRAVQLLRNKGFVSAKDIIGGLESWAQDVDPNFPAY

>tr|A0A6P6TQQ4|A0A6P6TQQ4_COFAR/1479 Adenylyltransferase and sulfurtransferase MOCS3 OS=Coffea arabica OX=13443 GN=LOC113703591 PE=3 SV=1MESNGRPQASEILRQIESLRSSKDEIERQISDLEAQLQQLNCDENNCRKAEEDEVNSNGSSSCLILPSENGSFDSGHGLASDMIYRYSRQLLLPAFGVQGQANLLKSSVLVIGAGGLGSPALLYLAACGFGRIGIVDHDMVELNNLHRQIIHTEAYIGRPKVESAAAACRAINSTVEIVEHTEAFRATNALGIVRKYDIVVDATDNVPSRYLINDCCVVLGKPLVSGAALGLEGQLTVYNYNGGPCYRCLFPTPPPTTACQRCADSGVLGVVPGIIGCLQALEAIKIGSAVGDPFSGRMLLFDAISGRIRIVKIRGRSLQCEACGENAPLTEKQFQEFDYECFTQTPFSTAPLKLSLLPVDARISSKEYHERIVKGEPHVLVDVRPAHHYKIVSLPNCINIPFPSLEARLHEISSALIKKDESKNSADSNASLYVVCRRGNESQRAVQYLHKMGFSSAKDIIGGIQSWAHDVDPKFPTY

>tr|A0A6P6U3P4|A0A6P6U3P4_COFAR/1479 Adenylyltransferase and sulfurtransferase MOCS3 OS=Coffea arabica OX=13443 GN=LOC113706857 PE=3 SV=1MESNGRPQASEILRQIESLRTSKDEIERQISDLEAQLQQLNCDENNCRKTEEDAVNSNGSSSCLILPSENGSFDSGHGLASDMIYRYSRQLLLPAFGVQAQANLLKSTVLVIGAGGLGSPALLYLAACGFGRIGIVDHDVVELNNLHRQIIHTEAYIGRPKVESAAAACRAINSTVEIVEHKEAFRATNALGIVRKYDIVVDATDNVPSRYLINDCCVVLGKPLVSGAALGLEGQLTVYNYNGGPCYRCLFPTPPPTTACQRCADSGVLGVVPGIIGCLQALEAIKIGSAVGDPFSGRMLLLDAISGQIRIVKIRGRSLQCEACGENAPLTEKQFQEFDYERFTQTPFSTAPLKLSLLPVDARISSKEYHERVVKGEPHVLVDVRPAHHYKIVSLPNSINIPFPSLEARLHEISSALMKKDESKNSADSNASLYVVCRRGNESQRAVEYLHKMGFSSAKDIIGGIQSWAHDVDPKFPTY

>tr|A0A6S7NCR1|A0A6S7NCR1_LACSI/1460 Adenylyltransferase and sulfurtransferase MOCS3 OS=Lactuca saligna OX=75948 GN=MOCS3 PE=3 SV=1MESNGGVDESTRLLKELQSLKDSKRDIEARISALEAQLRQIQSNQQLNKKASSDCSNGGSEFGHDLTPDMIYRYSRQLLLPSFGVQGQSNLLKSSILVIGAGGLGSPALLYLAACGVGRLGMVDHDVVELNNLHRQIIHGEAYIGRSKVESAAAACRSINSTIEIIEHKEALRTSNALEIVSKYDIVIDATDNAPSRYMISDCCVLLGKPLVSGAALGLEGQLAVYNYNGGPCYRCLFPTPPPTTACQRCSDSGVLGVVPGVIGCLQALEAIKVASLVGEPLSGRMLLFDALSARIRIVKIRGRSLQCEACGENATMTLENFQHFDYEKFTQSPLSPAPLKLKLLEEDCRISSKEYEEVVKKGDAHVLIDVRPSQHYKIVSLPNSMNVPLASLEEKLPEIESALKSIDNGNGGVYVVCRRGNDSQRAVQLLHNKGFVSAKDIVGGLESWARDVDHRFPTY

>tr|A0A7J7C2S4|A0A7J7C2S4_TRIWF/1469 Adenylyltransferase and sulfurtransferase MOCS3 OS=Tripterygium wilfordii OX=458696 GN=MOCS3 PE=3 SV=1MDSNGGGEVCRILRELKTLKASKSDIERRISDLEAQLRELNLNNGTVVPNGSSCTLMSTVDSGFGHGLSADMIYRYSRQLLIPSFGVQAQSNLLKSSILVVGAGGLGSPALLYLAACGVGRLGIVDHDVVELNNMHRQIIHTEAYIGQPKVKSAAASCRSINSTIQIVEHKEALCASNALEILSKYDIIVDATDNAPSRYMISDCCVVLGKPLVSGAAVGLEGQLTVYNYNGGPCYRCLFPTPPPTTACQRCSDSGVLGVVPGVIGVLQALETIKIAAAVGEPLSGRMLLFDALSARIRIVKIRGRSLQCEVCGENTAFTQKQFSEFDYENFTQSPLSTSPLKLNLLPADSRISSKEFKERIVNGEANVLVDVRPPHHYKIVSLPGSVNIPLSRLESRLPEISSFLNETKERKALNPDSVPSLYVICRRGNDSQRAVQLLHKMGFTIAKDIIGGLESWAQDVDQNFPTY

>tr|A0A7J7HAQ5|A0A7J7HAQ5_CAMSI/1460 Adenylyltransferase and sulfurtransferase MOCS3 OS=Camellia sinensis OX=4442 GN=MOCS3 PE=3 SV=1MEANGGESSRILREIENLKSAKSEIERRISDLEAQLRDIDEKDETKTNSTRSCPPLSNGDSGFGHGLSPDMIYRYSRQLLLPSFGVQAQSSLLKASILVVGAGGLGSPALLYLAACGVGRLGIVDHDVVELNNLHRQIIHTEAYIGQSKVESAAAACHSINSTIQIVEHKEALRTSNALEILSKYDIVIDATDNAPSRYLISDCCVVLGKPLVSGAAIGLEGQLTVYNYNGGPCYRCLFPTPPPTTACQRCSDSGVLGIVPGIIGCHQALEAIKIASDVGEPLSRRMLLFDALTARIRIVKIRGRSSQCEVCGENATFTAKQFQEFDYEKFTQSPLSMTPLKLDLLPTEARISSKEYKERVVRGEPHALVDVRPAHHFKISSLPNSMNVPLSSLEARLPEISSALKREEEHASLYVVCRRGNDSQRAVDYLHKMGFTSAKDIIGGLESWAHDVDPNFPTY

>tr|A0A7N0UXM5|A0A7N0UXM5_KALFE/1457 Adenylyltransferase and sulfurtransferase MOCS3 OS=Kalanchoe fedtschenkoi OX=63787 GN=MOCS3 PE=3 SV=1MASSDGAVDDASRIRREIEALKNERSDIDRQISDLETQLRGLQSKTEVVHNDGLAAHGLSPDLIYRHSRHLMLPSFGVQGQSKLSKSSVLVVGAGGLGSPALLYLAACGLGKIGIVDHDVVELNNLHRQVIHSEEYIGSPKVESAAAACLKINSTVLITKHQEALRTTNALDIVSKYDIVIDATDNAPSRYLINDCCVVLGKPLVSGAAIGMEGQLTVYNYKGGPCYRCLFPLPPPTTACQRCSDSGVLGVVPGIIGCLQALEAIKIASSVGEPLSGRMLLLDSLSTKIRIVKIRGRSSQCEVCGENSFFTPQHFKGFDYEKFTQTPLSVAPVKLSLLTPDFRITAKEYKQRIVQGEAHVLVDVRPEHHFKIVSLPNALNIPLTMIEQRVGDISSALKEKQPNSSGSSPHLYVVCRRGNDSQRAVQLLHKLGFVAARDIIGGIESWAREVDPKFPTY

>tr|A0A7N2R8P3|A0A7N2R8P3_QUELO/1462 Adenylyltransferase and sulfurtransferase MOCS3 OS=Quercus lobata OX=97700 GN=MOCS3 PE=3 SV=1MEPNGHDEASRILHELQSLKATKSDIDRRISALESQLRHLNLQNGTVVKTSALPNPITAAHHDLTPEMIHRYSRHLLLPSFGVKAQSNLINSSILVVGAGGLGSPALLYLAACGAGRLGIVDHDVVELNNMHRQVIHTEAFIGQPKVKSAATTCRSINSSVQIVEHHEALRTSNALEIFNQYDVIVDATDNAPSRYMISDCCVLLGKPLVSGAALGLEGQLTVYNYNGGPCYRCLFPTPPPTAACQRCADSGVLGVVPGIIGCLQALEAIKIASVVGEPLSGRMLLFDALAARIRIVKIRGRSPQCEACGENATFTQQQFREFDYEKFTQSPLTPSPLKLKLLPSDSRISSKEYHDRVANGEAHVLVDVRPAHHFKIVSLPKSLNIPLPSLEARLPEISSALKEEEERNGSLSAKLYVICRRGNDSQRAVQNLHKLGFTSARDIVGGLESWAHDVDSNFPTY

>tr|A0A830CJX0|A0A830CJX0_9LAMI/1462 Adenylyltransferase and sulfurtransferase MOCS3 OS=Phtheirospermum japonicum OX=374723 GN=MOCS3 PE=3 SV=1MDSNGAEETAADILQEIVSLEKDKARIEARISALRARLISYNSSENGGVEANSSAPRPAESQLSADAIYRYSRHLILPSFGVNAQANLLKSSVLVIGAGGLGAPALLYLAASGVGRVGIVDHDVVELNNLHRQVIHTEAYIGRSKVESAAAACRAINSTVQIVEHKEAFRTSNALEIMKKYDIVVDATDNVPSRYMISDCCVVLGKPLISGAALGVEGQLTVYNYKGGPCYRCLFPTPPPTTACQRCSDSGVLGVVPGIIGCLQALEAIKVAGEVGEPLSGRMLLLDALSARLRVVKIRGRSLQCEACGENAMLTEQQFRDFDYEEFTQSPLSTSPLKLELLEAEARITSKEYNDINSKGEAHVLVDVRPSHHYKIISLPNSINIPLASLEERISEISSALAREEVKNETGRGSSLYVICRRGNDSQRAVELLRKIGFTLAKDIIGGLESWAHNVDPQFPTY

>tr|A0A833V4N6|A0A833V4N6_9POAL/1467 Adenylyltransferase and sulfurtransferase MOCS3 OS=Carex littledalei OX=544730 GN=MOCS3 PE=3 SV=1MAAQVIRTDGATSDWIHGEIQRLKLEKEQLENRISALESQLHTSTSSSPVAKIEENSSCSITDGSTFCANGSSANALSAEMIYRYSRQLLLPDFGVEGQRKLSQSRVLVIGAGGLGSPVAMYLASCGVGCIGIVDGDTVELNNLHRQIIHTEAYVGKPKVQSAAAACHAINSSVKVVEHQGLLHPSNALKIVNQYDVVVDATDNLPSRYMISDCCVVLQKPLVSGAALGLEGQLTVYNHKGSPCYRCLFPTPPPSAACQRCSDSGVLGVVPGVIGCMQALEAIKIAADVGEPLSGRMLLFDALSARIRIVKLRGRSSACTICGENSSFTKEEFERFDYENFTQSPMSDRTRPKLSLLPESARITIKEYKEVLDKSERHTLLDVRPVHQFKITSLPNSVNIPLTDLEKELPKLGSMLNDSSLYVICRRGNTSQTAVQLLHQKGFFSAKDIIGGLESWAQEIDPKFPIY

>tr|A0A834GXZ0|A0A834GXZ0_RHOSS/1470 Adenylyltransferase and sulfurtransferase MOCS3 OS=Rhododendron simsii OX=118357 GN=MOCS3 PE=3 SV=1MESKGAEPSRIRREIDSLKSSKIEIERRISALESQLRDIDGNKDVPSTNLTSPCSPLSTNGDSGFGHGLSPDMIYRYSRHLLLPSFGVKGQSSLLKTSILVVGAGGLGSPALLYLAACGVGRIGIVDHDIVELNNLHRQIIHSEAYIGQSKVESAAAACRSINSTIQIVEYKEALRTTNALEILRKYDIVVDATDNAPSRYLISDCCVVLGKPLVSGAALGLEGQLTVYNYNGGPCYRCLFPTPPPTTACQRCSDSGVLGVVPGIIGCLQALEAIKVASGVGEPLSGRMLLFDALSARIRIVKIRGRLSQCEVCGENAAFTEQQFREFDYEKFTQSPLSTSPLKLNLLPAEARISSKEYNERVFKGEAHVLVDVRPAHHYKIVSLPKSMNIPLSNLEARLSELSSVLKKDDEQSGSDGVSAASLYVVCRRGNDSQRAVQYLHKMGFTSAKDIIGGLESWAHDVDPTFPTY

>tr|A0A834YNZ3|A0A834YNZ3_9MAGN/1466 Adenylyltransferase and sulfurtransferase MOCS3 OS=Tetracentron sinense OX=13715 GN=MOCS3 PE=3 SV=1MESNGGDASRLLREIETLKAAKSDIEHRISILQAQLGEIVAKEETSSISPLSISDACVRHGLSPEMIYRHSRHLLLPSFGVQGQANLSKSSILVVGAGGLGSPALLYLAACGVGRLGIVDHDIVELNNLHRQIIHTEAYIGQPKVTSAAAACRAINSTVQIVEHKEALRTSNALEIVNEYPLYDIVIDATDNLPSRYMISDCCVVSGKPLVSGAALGLEGQLTVYNHNGGPCYRCLFPTPPPTSACQRCSDSGVLGVVPGVIGCLQALEAIKIASVVGEPLSGRMLLFDALSARIRVVKIRARSLQCEICGENTSFTQQLFRDFDYENFTESPLSASPLKLNLLPRSARISSREYKEKVVNGEAHVLVDVRPEHHFQIVSLPKSLNIPLPKLEGKLPEISSALREEQEHTGVISCSDASLYVICRRGNDSQRAVQYLHKMGFTSAQDIIGGLESWAHDVDPNFPTY

>tr|B8AIQ9|B8AIQ9_ORYSI/1470 Adenylyltransferase and sulfurtransferase MOCS3 OS=Oryza sativa subsp. indica OX=39946 GN=MOCS3 PE=3 SV=1MAGELERLRAEREELDSRIRLLESQLGASPTPAGEGDAAGTGAGGGVGGGGATACPIRRRGNGFAAADGLPADMIYRYSRHLLLPDFGVEGQRKLSQSSILVVGAGGLGSPVALYLAACGVGCLGIVDGDDVELNNLHRQIIHKEAFVGKSKVKSAADACREINSSINVMEYHHTLKPSNALEIVRKYDIVVDATDNLPTRYMISDCCVLLNKPLISGAALGLEGQLTVYHHNGSPCYRCLFPNPPPVAACQRCSDSGVLGVVPGVIGCLQALEAIKVATDVGEPLSGRMLLFDALSARIRIVKIRGSSTVCTVCGENSAFTQDDFQKFDYENFTQSPMSDKSAPSLDILPGSARVTCKEYKRLADNGERHLLLDVRPAHHFQIASVSQSLNIPLSELEEKLQMLETSLKDTTDASSSDKPPSLYVVCRRGNDSQIAVQLLREKGFLSAKDIIGGLQSWAQDVDPDFPVY

>tr|B9F0B4|B9F0B4_ORYSJ/1470 Adenylyltransferase and sulfurtransferase MOCS3 OS=Oryza sativa subsp. japonica OX=39947 GN=MOCS3 PE=3 SV=1MAGELERLRAEREELDSRIRLLESQLGASPTPAGEGDAAGTGAGGGGCGCGATACPIRRRGNGFAAADGLPADMIYRYSRHLLLPDFGVEGQRKLSQSSILVVGAGGLGSPVALYLAACGVGCLGIVDGDDVELNNLHRQIIHKEAFVGKSKVKSAADACREINSSINVMEYHHTLKPSNALEIVRKYDIVVDATDNLPTRYMISDCCVLLNKPLISGAALGLEGQLTVYHHNGSPCYRCLFPNPPPVAACQRCSDSGVLGVVPGVIGCLQALEAIKVATDVGEPLSGRMLLFDALSARIRIVKIRGSSTVCTVCGENSAFTQDDFQKFDYENFTQSPMSDKSAPSLDILPGSARVTCKEYKRLVDNGERHLLLDVRPAHHFQIASVSQSLNIPLSELEEKLQMLETSLKDTTDASSSDKPPSLYVVCRRGNDSQIAVQLLREKGFLSAKDIIGGLQSWAQDVDPDFPVY

>tr|B9STZ8|B9STZ8_RICCO/1467 Adenylyltransferase and sulfurtransferase MOCS3 OS=Ricinus communis OX=3988 GN=MOCS3 PE=3 SV=1MESNGSESTGILREIETLKTEKSNIDNRIAALEAQLRQINLQNDTASSNGSCPLISTNDSGLSHALSSDMIYRYSRHLLLPSFGIQGQSNLLKSSILVVGAGGLGSPALLYLAACGVGRLGVVDHDVVELNNMHRQVIHTEAFIGQPKVKSAAAACRSINSTIQIVEHQEALRTSNALEIFSQYDIIVDATDNAPSRYMISDCCVVLGKPLVSGAALGLEGQLTVYNYKGGPCYRCLFPTPPPSTACQRCADSGVLGVVPGIIGCLQALEAIKIASDIGEPLSGRMLLFDALSARIRIVKIRGRSLQCEVCGENSAFTQKQFRDFDYEKFTQTPLSMAPLKLDLLPADSRINSREFNEKVIKGETHVLVDVRPAHHFKIVALPNALNIPLSSLEARLPEISSALKEEGERRGVDSESGVNLYVVCRRGNDSQRAVQLLHKKGFSIAKDIIGGIEAWAHDVDPNFPTY

>tr|D7MV01|D7MV01_ARALL/1464 Adenylyltransferase and sulfurtransferase MOCS3 OS=Arabidopsis lyrata subsp. lyrata OX=81972 GN=MOCS3 PE=3 SV=1MMSNGGDSSEIIRELEELKLKKADIEHRISTLEAKLQDTAAVERYDTVSNGDSYPTAPELKHGLSPDQIYRYSRQLLLPSFAVEGQSNLLKSSVLVIGAGGLGSPALLYLAACGVGRLGIIDHDVVELNNMHRQIIHTEAFIGHPKVKSAATACRSINSTIKVDEYVEALRTSNALEILSQYDIIVDATDNPPSRYMISDCCVLLGKPLVSGAALGMEGQLTVYNHNGGPCYRCLFPTPPPTSACQRCSDSGVLGVVPGVIGCLQALETIKLASMVGEPLSERMLLFDALSARMRIVKIRGRSSQCTVCGDNSSFNKQTFKDFDYEDFTQFPLFAGPLNLLPAESRISSTEFKEILQKKEQHVLLDVRPSHHYKIVSLPDSLNIPLANLEARLNELTSALKEKEDGHVNTGSCTNPSVYVVCRRGNDSQRAVHYLRESGFDSAKDIIGGLEAWAANVNPNFPTY

>tr|E4MVZ3|E4MVZ3_EUTHA/1466 Adenylyltransferase and sulfurtransferase MOCS3 OS=Eutrema halophilum OX=98038 GN=MOCS3 PE=2 SV=1MEASSEIIRELEELKLKKAEIEHRISSLKAKLQETAAAAERCDAVSNGYSYPMAAAAIEHGLGHGLSPDQIYRYSRQLLLPSFGVEGQASLLKSSVLVIGAGGLGSPALLYLAACGVGRLGIIDHDVVELNNMHRQIIHTEAFIGHPKVKSAAAACRSINSTIKIDEYLEALRTSNALEILSQYDIIVDATDNPPSRYMISDCCVLLGKPLVSGAALGMEGQLTVYNHKGGPCYRCLFPTPPPTTACQRCSDSGVLGVVPGVIGCLQALETVKLASMVGEPLTERMLLFDALSARIRIVKIRGKSSLCTVCGDNSSFNKQQFKDFDYEDFTQFPLSAGPLNLLPTESRISSKEFKEILQKKEPHVLLDVRPCHHYKIVSLPDSLNIPLANLEARLNELSSALKEKEDGHVNTGSCTNPSLYVVCRRGNDSQRAVQYLRESGFDSAKDIIGGLEAWAADVNPNFPTY

>tr|I1JJC5|I1JJC5_SOYBN/1447 Adenylyltransferase and sulfurtransferase MOCS3 OS=Glycine max OX=3847 GN=MOCS3 PE=3 SV=1MSASEIVRELDSLKDEKTIIEQKISALEAQLREINLQNDAAPPNGLTQDMIHRYSRHLVLPSFGVQGQANLLKSSILVVGAGGLGAPALLYFAASGVGRLGVIDHDVVELNNMHRQVIHTEAYVGKPKVKSAAAACRSINSTIQVVEHEEALRTSNALELLSKYDIIVDATDNAPTRYLISDCCVVLGKPLVSGAALGLEGQLTVYNYNGGPCYRCLFPTPPPRTACQSCAEGGVLGVVPGIIGCLQALEAIKIAASVGEPLSGRMLILDALSGRIRIVKIRGRSMQCEACGENATFTQQQFREFDYEKFTQTPLRVPPLKLNLLPSESRISSKEYSEVILKKEPHVLVDVRPAHHFKIVSLPKSLNIPLSTLEARLPEVSSALKKEEEEGGVVSGSSAQLYVVCRRGNDSQRAVQYLHKMGFISAKDIVGGLESWAHNVDPKFPTY

>tr|I1P0V2|I1P0V2_ORYGL/1473 Adenylyltransferase and sulfurtransferase MOCS3 OS=Oryza glaberrima OX=4538 GN=MOCS3 PE=3 SV=1MEAMAGELERLRAEREELDSRIRLLESQLGASPTPAGEGDAAGTGAGGGGGGGGATACPIRRRGNGFAAADGLPADMIYRYSRHLLLPDFGVEGQRKLSQSSILVVGAGGLGSPVALYLAACGVGCLGIVDGDDVELNNLHRQIIHKEAYVGKSKVKSAADACREINSSINVMEYNHTLKPSNALEIVRKYDIVVDATDNLPTRYMISDCCVLLNKPLISGAALGLEGQLTVYHHNGSPCYRCLFPNPPPVAACQRCSDSGVLGVVPGVIGCLQALEAIKVATDVGEPLSGRMLLFDALSARIRIVKIRGSSTVCTVCGENSAFTQDDFQKFDYENFTQSPMSDKSAPSLDILPGSARVTCKEYKRLVDNGERHLLLDVRPAHHFQIASVSQSLNIPLSELEEKLQMLETSLKDTTDASSSDKPPSLYVVCRRGNDSQIAVQLLREKGFLSAKDIIGGLQSWAQDVDPDFPVY

>tr|J3LDC8|J3LDC8_ORYBR/1414 Adenylyltransferase and sulfurtransferase MOCS3 OS=Oryza brachyantha OX=4533 GN=MOCS3 PE=3 SV=1MRRRGNGFAAADGLPADMIYRYSRHLLLPDFGVEGQRKLSQSSILVVGAGGLGSPVALYLAACGVGCLGIVDGDDVELNNLHRQIIHKEAYVGKSKVKSAADACCEINSSIKVMEYHHTLKPCNALEIVRKYDIVVDATDNLPTRYMISDCCVLLNKPLISGAALGLEGQLTVYHHNGSPCYRCLFPNPPPVAACQRCSDSGVLGVVPGVIGCLQALEAIKVATDVGEPLSGRMLLFDALSARIRIVKIRGSSNVCTVCGENSAFTQDDFQKFDYENFTQSPMSDKSAPSLDLLPESARVTCTEYKRLVDNGEPHLLLDVRPAHHFQIASVSQSLNIPLAVLEEKLQIVETSLKETMDASTPDKMPSLYVVCRRGNDSQIAVQILREKGFLSAKDIIGGLQSWAQDVDPDFPVY

>tr|M4CFE8|M4CFE8_BRARP/1466 Adenylyltransferase and sulfurtransferase MOCS3 OS=Brassica rapa subsp. pekinensis OX=51351 GN=MOCS3 PE=3 SV=1MAANGGDSSEIVRELKELKLQKAKIEHRISTLEAKLQETATAERCDAVSNGCSVPTEIEHGLEHGMSPDQIYRYSRQLLLPSFGVEGQSNLLKSSVLVIGAGGLGSPALLYLAACGVGRLGIIDHDVVELNNMHRQVIHTEAFIGHPKVKSAATACRSINSTIKIDEYVEALRTSNALEILSQYDIIVDATDNPPSRYMISDCCVLLGKPLVSGAALGMEGQLTVYNHKGGPCYRCLFPTPPPTTACQRCSDSGVLGVVPGAIGCLQALETIKLASMVGEPLSERMLLFDALSARIRIVKIRGRSAQCTVCGDNSSFNKQQFKDFDYEEFTQFPLSAGPLNLLPAESRISSKEFKEILQKKERHVLLDVRPSHHYKIVSLPDSLNIPFANLEARLNELTSALKDKEDDHVNSGSCANPSLYVVCRRGNDSQRAVQYLRDSGFSSAKDIIGGLEAWAADVNPNFPSY

>tr|R0G9R7|R0G9R7_9BRAS/1464 Adenylyltransferase and sulfurtransferase MOCS3 OS=Capsella rubella OX=81985 GN=MOCS3 PE=3 SV=1MESYSGASSEIVRELEELKLKKAELEHRISTLEAKLQDTAAVERCDAVSNGYSYPTAPELEHGLSPDQIYRYSRQLLLPSFAVEGQSNLLKSSVLVIGAGGLGSPALLYLAACGVGRLGIIDHDVVELNNMHRQIIHTEAFIGHPKVKSAAVACRSINSTIKIDEYVEALRTSNALEILSQYDIIVDATDNPPSRYMISDCCVLLGKPLVSGAALGMEGQLTVYNHNGGPCYRCLFPTPPPTTACQRCSDSGVLGVVPGIIGCLQALETIKLASMVGDALTERMLLFDALSARIRIVKIRGRSSQCTVCGDNSSFDKQKFKDFDYEDFTQFPLFAGPLNLLPTESRISSKEFKEILQKKEPHILLDVRPSHHYKIVSLPESLNIPLANLEARLNEITSALKEKEDGHVNTGYCTNPSVYVVCRRGNDSQRAVQYLRDSGFSSAKDIVGGLEAWAADVNPNFPTY

>tr|V4LGS7|V4LGS7_EUTSA/1466 Adenylyltransferase and sulfurtransferase MOCS3 OS=Eutrema salsugineum OX=72664 GN=MOCS3 PE=3 SV=1MEASSEIIRELEELKLKKAEIEHRISSLKAKLQETAAAAERCDAVSNGYSYPMAAAAIEHGLGHGLSPDQIYRYSRQLLLPSFGVEGQASLLKSSVLVIGAGGLGSPALLYLAACGVGRLGIIDHDVVELNNMHRQIIHTEAFIGHPKVKSAAAACRSINSTIKIDEYLEALRTSNALEILSQYDIIVDATDNPPSRYMISDCCVLLGKPLVSGAALGMEGQLTVYNHKGGPCYRCLFPTPPPTTACQRCSDSGVLGVVPGVIGCLQALETVKLASMVGEPLTERMLLFDALSARIRIVKIRGKSSLCTVCGDNSSFNKQQFKDFDYEDFTQFPLSAGPLNLLPTESRISSKEFKEILQKKEPHVLLDVRPCHHYKIVSLPDSLNIPLANLEARLNELSSALKEKEDGHVNTGSCTNPSLYVVCRRGNDSQRAVQYLRESGFDSAKDIIGGLEAWAADVNPNFPTY

>tr|V4UHV7|V4UHV7_CITCL/1467 Adenylyltransferase and sulfurtransferase MOCS3 OS=Citrus clementina OX=85681 GN=MOCS3 PE=3 SV=1METNGGSTDVARVLGEIETLKAAKSDIDYRISALEAQLRDTTVSQPQTDTVSNGSYRPSSAVDYGLSPDMIYRYSRHLLLPSFGVEGQSNLLKSSILVIGAGGLGSPALLYLAACGVGRLGIVDHDVVELNNMHRQVIHTEPYIGQSKVKSAAATCRSINSTVHIIEHREALRTSNALEILSQYEIVVDATDNAPSRYMISDCCVVLGKPLVSGAALGLEGQLTVYNYNGGPCYRCLFPTPPPTTACQRCADSGVLGVVPGIIGCLQALEAIKVASAVGEPLSGRMLLFDALSARIRIVKIRGRSSQCEACGENSTFTQDHFRNFDYEKFTQSPLSTLPLKLNLLSADSRISSKEYKEKVVNGEAHILVDVRPAHHFRIVSLPNSINIPLSDLESRLPEISSAMKEKEEHRGSNASSGSNLYVVCRRGNDSQRAVQALHKLGFTSARDIIGGLESWANDVDPSFPVY

>tr|V4UT14|V4UT14_CITCL/1444 Adenylyltransferase and sulfurtransferase MOCS3 OS=Citrus clementina OX=85681 GN=MOCS3 PE=3 SV=1METNGGSTDVARVLGEIETLKAAKSDIDYRISALEAQLRDTTVSQPQTDTVSNGSYRPSSAVDYGLSPDMIYRYSRHLLLPSFGVEGQSNLLKSSILVIGAGGLGSPALLYLAACGVGRLGIVDHDVVELNNMHRINSTVHIIEHREALRTSNALEILSQYEIVVDATDNAPSRYMISDCCVVLGKPLVSGAALGLEGQLTVYNYNGGPCYRCLFPTPPPTTACQRCADSGVLGVVPGIIGCLQALEAIKVASAVGEPLSGRMLLFDALSARIRIVKIRGRSSQCEACGENSTFTQDHFRNFDYEKFTQSPLSTLPLKLNLLSADSRISSKEYKEKVVNGEAHILVDVRPAHHFRIVSLPNSINIPLSDLESRLPEISSAMKEKEEHRGSNASSGSNLYVVCRRGNDSQRAVQALHKLGFTSARDIIGGLESWANDVDPSFPVY

>tr|V7B929|V7B929_PHAVU/1457 Adenylyltransferase and sulfurtransferase MOCS3 OS=Phaseolus vulgaris OX=3885 GN=MOCS3 PE=3 SV=1MSASEILRELDSLKDAKAKIEHKISALEAQLKEINLRNDAAPPNGSSPSYPTNGLTQDMIHRYSRHLMLPSFGVQGQANLLKSSILVVGAGGLGAPALLYFAASGVGRLGIVDHDVVELNNMHRQVIHTEAYVGKPKVKSAAAACRSINSTIEVVEYEEALRTSNALEILSKYDIIVDATDNAPTRYLISDCCVVLGKPLVSGAALGLEGQLTVYNYNGGPCYRCLFPLPPPRTACQSCAEGGVLGVVPGIIGCLQALEAIKIAAAVGEPLSGRMLLLDALSGRIRIVKIRGRSVHCEACGENATFTQQKFREFDYEKFTETPLRVPPLKLNLLPSESRISTKEYSEIILKKEPHVLVDVRPAHHFKIVSLPKSLNIPLSTLEARLPEISSALKKEEEKDSGLVSGSGAQLYVVCRRGNDSQRAVQCLHKMGFTSAKDIVGGLESWAYNVDPKFPTY

>tr|A0A8B8JJ36|A0A8B8JJ36_ABRPR/1455 Adenylyltransferase and sulfurtransferase MOCS3 OS=Abrus precatorius OX=3816 GN=LOC113846921 PE=3 SV=1MSASEILRELNSLKNEKTKIEHKISVLEAQLRDINLQNDAVSDDGSSLYPTNGLTRDMIHRYSRHLMLPSFGVQGQANLLKSSILVVGAGGLGAPALLYLAASGVGRLGIIDHDVVELNNMHRQIIHTEAFVGKPKVKSAAAACRSINSSIQVVEYEEALQNSNALEIFSKYDLIVDATDNAPTRYLISDCCVVLGKPLVSGAALGLEGQLTVYNYNGGPCYRCLFPIPPPRTACQSCADGGVLGVVPGIIGCLQALEAIKIVASVGEPLSGRMLLFDALAGRIRTVKIRGRSVECKACGENATFAQQQFREFDYEKFTQTPLRVPPVKLNLLPSESRISSKEYSEIILKKEPHVLVDVRPAHHFKIVSLPKSLNIPLSTLEVRLPEISSALKKEEEDSGVVSGSSAQLYVVCRRGNDSQKAVQYLQKLGFTSAKDIVGGLESWAHNVDPKFPTY

>tr|A0A8B8Q770|A0A8B8Q770_9MYRT/1462 Adenylyltransferase and sulfurtransferase MOCS3 OS=Rhodamnia argentea OX=178133 GN=LOC115750003 PE=3 SV=1MDSGGADAARILSEIKGLKATKSDIEAKISALEAQLKDLNCRNDAVSSNGSCPLATSLDSGLGHGLSPEMIHRYSRQLLLPSFGVQAQSNLLKSSILVVGAGGLGSPALLYLAACGVGRLGIVDNDEVELNNMHRQIIHTEAYIGRLKVESAAAACRAINSTVQIVEHKEALRTSNALEILSKYDVVVDATDNAPGRYMISDCCVVLGKPLVSGAALGLEGQLSVYNYNGGPCYRCLFPTPPPTTACQRCSDSGVLGVVPGIIGCLQALEAIKIASAVGEPLVGRMLLFDALSGRIRIVKIRGRSLHCEVCGENATFTRQQFQEFDYEKFTQSPLSASAPPKLNLLSGDSRISSKDYNERLKNGEVHILVDVRPEHHFRIVSLPNSLNIPLSTLEKRLAEISSALREAEGQRDASLYVICRRGNDSQRAVNYLHDIGFTSAKDIIGGLESWARDVDPSFPTY

>tr|A0A6A4LIT2|A0A6A4LIT2_9ERIC/1470 Rhodanese domaincontaining protein (Fragment) OS=Rhododendron williamsianum OX=262921 GN=C3L33_09812 PE=3 SV=1MXSNEAEASRIRREIDSLKSSKIEIERRISALESQLRDIDCNKDEPSTNSTSPCTPLSTNGDSGFGHGLSPDMIYRYSRHLLLPSFGVQGQSSLLKTSILVVGAGGLGSPALLYLAACGVGRIGIVDHDIVELNNLHRQIIHSEAYIGQSKVESAAAACRSINSTIQIVEYKEALRTSNALEILRKYDIVVDATDNAPSRYLISDCCVVLGKPLVSGAALGLEGQLTVYNYNGGPCYRCLFPTPPPTTACQRCSDSGVLGVVPGIIGCLQALEAIKVASGVGEPLSGRMLLFDALSARIRIVKIRGRSSQCEVCGENATFTEQQFREFDYEKFTQSPLSTSPLKLNLLPAEARISSKEYNLRVVKGEAHVLVDVRPALHYKIVSLPKSMNIPLSNLEARLSELSSVLKKDDEQIGSDGVSAASLYVVCRRGNDSQRAVQYLHKMGFTSAKDIVGGLESWAHDVDPTFPTY

>tr|S8C363|S8C363_9LAMI/1448 Rhodanese domaincontaining protein (Fragment) OS=Genlisea aurea OX=192259 GN=M569_13711 PE=3 SV=1TASDIRREIASLVRDKNEIEEKIASLQARLDGFGFENGANSSSAELLQTGELSADMIYRYSRHLLLPSFGVEAQANLLKSSVLVIGAGGLGAPALLYLAACGVGRVGIVDHDVVELNNLHRQVIHTEAYIGRSKVESAAAACRAINSSVKIVEHKEAFTTSNALEIMRRYDVVVDATDNVPSRYLISDCCVVLGKPLVSGAALGLEGQLTVYNFNGGPCYRCLFPTPPPSTACQRCADSGVLGVVPGIIGCLQALEAIKVTGGIGEPLSGRMLLFDAISSRFRVVRIRGKSSQCEACGENAKMSEESFRNFDYEKFTESSLITSPWKLELVPREARITGREYHSRVANGERHVLIDVRPSHHYKIVSLPGSINVPLGRMEGKVGEIRAALSSDDETGVVGGLYVICRRGNDSQRAVEFLHKVGLSCAKDIIGGLESWAKDVDPTFPTY

>tr|A0A0E0K0I4|A0A0E0K0I4_ORYPU/1458 Adenylyltransferase and sulfurtransferase MOCS3 OS=Oryza punctata OX=4537 PE=4 SV=1MEAITGELARLRAEREELDSRIRFLESQLEASPPPSAPPAGEGGAAGTGAGGGGGGAAAAAVCPIRRHGNGFSAADGLPTDMIYRYSRHLLLPDFGVEGQRKLSQSSILVVGAGGLGSPVALYLAACGVGSVAWALLMEMMLSLITFIDRINSSIKVMEYHHTLKPSNALEIVRKYDIVVDATDNLPTRYMISDCCVLLNKPLISGAALGLEGQLTVYHHNGSPCYRCLFPNPPPVAACQRCSDSGVLGVVPGVIGCLQALEAIKVATDVGEPLSGRMLLFDALSSRIRIVKIRGSSTVCTVCGENSAFTQDDFQKFDYENFTQSPMSDKSAPSLDILPESARVTCKEYKRLVDNGERHLLLDVRPAHHFQIASVSQSLNIPLSELEEKLQMLKTSLKGTIDVSSSDKPPSLYVLCRRGNDSQMAVQLLREKGFLSAKDIIGGLQSWAQDVDPDFPVY

>tr|A0A0R0GJH5|A0A0R0GJH5_SOYBN/1335 Rhodanese domaincontaining protein OS=Glycine max OX=3847 GN=100814736 PE=4 SV=1MHRQVIHTEAYVGKPKVKSAAAACCSINSTIQVVEHEEALQTSNALEILSKYDIIVDATDNAPTRYLISDCCVVLGKPLVSGAALGLEGQLTVYNYNGGPCYRCLFPTPPPRTACQSCAEGGVLGVVPGIIGCLQALEAIKIAASVGEPLSGRMLLLDALSGRIRIVKIRGRSMQCEACGENATFTQQQFREFDYEKFTQTPLRVPPLKLNLLPRESRISSKEYSEVIIKKGPHVLVDVRPAHHFKIASLPKSLNIPLSTLEARLPEVSSALKKEEEESGAVSGSSAQLYVVCRRGNDSQRAVQYLHKMGFTSAKDIVGGLESWAHNVDHQFPTY

>tr|A0A1U8J9X0|A0A1U8J9X0_GOSHI/1332 adenylyltransferase and sulfurtransferase MOCS3 OS=Gossypium hirsutum OX=3635 GN=LOC107903468 PE=4 SV=1MHRQVIHTEAYIGQPKVKSAAAACRSINSTIQIVEHKQALRTSNALEILSQYDIVVDATDNAPSRYMISDCCVVLGKPLVSGAALGLEGQLTVYNYKGGPCYRCLFPTPPPTTACQRCSDSGVLGVVPGIIGCLQALEAIKIASDIGEPLSGRMLLFDALSARIRIVKIRGRSLQCEVCGENTTFNQQQFKELDYEKFTQSPLSTSPPKLKLLAPDSRITSKEYKERIISGEPHVLVDVRPELHYKIVSMPNSLNIPLASLETRLPEISSALKEQEKEGTRSGANLYVICRRGNDSQRAVDYLRNKGFDLAKDIVGGLESWANDVDPNFPMY

>tr|A0A8B8JJ89|A0A8B8JJ89_ABRPR/1428 adenylyltransferase and sulfurtransferase MOCS3 isoform X2 OS=Abrus precatorius OX=3816 GN=LOC113846921 PE=4 SV=1MSASEILRELNSLKNEKTKIEHKISVLEAQLRDINLQNDAVSDDGSSLYPTNGLTRDMIHRYSRHLMLPSFGVQASGVGRLGIIDHDVVELNNMHRQIIHTEAFVGKPKVKSAAAACRSINSSIQVVEYEEALQNSNALEIFSKYDLIVDATDNAPTRYLISDCCVVLGKPLVSGAALGLEGQLTVYNYNGGPCYRCLFPIPPPRTACQSCADGGVLGVVPGIIGCLQALEAIKIVASVGEPLSGRMLLFDALAGRIRTVKIRGRSVECKACGENATFAQQQFREFDYEKFTQTPLRVPPVKLNLLPSESRISSKEYSEIILKKEPHVLVDVRPAHHFKIVSLPKSLNIPLSTLEVRLPEISSALKKEEEDSGVVSGSSAQLYVVCRRGNDSQKAVQYLQKLGFTSAKDIVGGLESWAHNVDPKFPTY

>tr|A0A8J4QZ36|A0A8J4QZ36_9ROSI/1462 Uncharacterized protein OS=Castanea mollissima OX=60419 GN=CMV_013769 PE=4 SV=1MEPNGHDEASRILHELQSLKATKSDIDRRISTLESQLRHLNLQNGAVVKTSTLPNPITAVHHDLTPEMIHRYSRHLLLPSFGVKAQSNLINSSILVVGAGGLGSPALLYLAACGAGRLGIVDHDVVELNNMHRQVIHTEAFIGQPKVKSAATTCRSINSSVQIVEHHEALRTSNALEIFNQYDVIVDATDNAPSRYMISDCCVLLGKPLVSGAALGLEGQLTVYNYNGGPCYRCLFPTPPPTAACQRCADSGVLGVVPGIIGCLQALEAIKIASAVGEPLSGRMLLFDALAARIRIVKIRGRSPQCEACGENATFTQQQFQEFDYEKFTQSPLTPSPLKLKLLQSDSRISSKEYHDRVANGEAHVLVDVRPAHHFKIVSLPKSLNIPLPSLEARLPEISSALKEEEERNGSSSAKLYVICRRGNDSQRAVQNLHKLGFTSARDIVGGLESWAHDVDSNFPTY

>tr|A0A8J5Z0C5|A0A8J5Z0C5_9ROSI/1444 Uncharacterized protein OS=Gossypium anomalum OX=47600 GN=CXB51_010341 PE=4 SV=1MESNGGGASPIRREIEALKEEKARIEQRISVLEAQLQEEASLAPQQQNDGVCNGFCPSEISTVDANLAHGLSADSIYRYSRHLLLPSFGVQAQSNLLKSSILVVGAGGLGSPALLYLAACGVGRLGIVDHDVVELNNMHRQVIHTEAYIGQPKVKSAAAACRSYDIVVDATDNAPSRYMISDCCVVLGKPLVSGAALGLEGQLTVYNYNGGPCYRCLFPTPPPTTACQRCSDSGVLGVVPGIIGCLQALEAIKIASSIGEPLSGRMLLFDALSARIRIVKIRGRSLQCEVCGENTTFNQQQFKDLDYEKFTQSPLSTSPPKLKLLTPDSRITSKEYKERIISGEPHVLVDVRPELHYKIVSIPNSLNIPLVSLEARLPEISSALKEQEKEGTGSGANLYVICRRGNDSQRAVDYLRNKGFDLAKDIVGGLESWANDVDPNFPMY

**Sequences retrieve by BlastP with *Saccharomyces cerevisiae* MOCS3 protein sequence (Uba4p /P38820)**

>sp|P38820|UBA4_YEAST/1440 Adenylyltransferase and sulfurtransferase UBA4 OS=Saccharomyces cerevisiae (strain ATCC 204508 / S288c) OX=559292 GN=UBA4 PE=1 SV=1MNDYHLEDTTSELEALRLENAQLREQLAKREDSSRDYPLSLEEYQRYGRQMIVEETGGVAGQVKLKNTKVLVVGAGGLGCPALPYLAGAGVGQIGIVDNDVVETSNLHRQVLHDSSRVGMLKCESARQYITKLNPHINVVTYPVRLNSSNAFDIFKGYNYILDCTDSPLTRYLVSDVAVNLGITVVSASGLGTEGQLTILNFNNIGPCYRCFYPTPPPPNAVTSCQEGGVIGPCIGLVGTMMAVETLKLILGIYTNENFSPFLMLYSGFPQQSLRTFKMRGRQEKCLCCGKNRTITKEAIEKGEINYELFCGARNYNVCEPDERISVDAFQRIYKDDEFLAKHIFLDVRPSHHYEISHFPEAVNIPIKNLRDMNGDLKKLQEKLPSVEKDSNIVILCRYGNDSQLATRLLKDKFGFSNVRDVRGGYFKYIDDIDQTIPKY

>tr|A0A0A8LAB2|A0A0A8LAB2_9SACH/1444 Adenylyltransferase and sulfurtransferase UBA4 OS=Kluyveromyces dobzhanskii CBS 2104 OX=1427455 GN=UBA4 PE=3 SV=1MTESERSNAHLLEEIERLRTENSKLRDELDAERCSSNMDKLPMSLDEFKRYGRQMIVDETEGLKGQLKLKNSSVLVVGAGGLGCPSLPYLAGAGIGKIGIVDNDIVDTSNLHRQVLHDSTKVGMFKCESAKQVLNKLNPHVSVTTYPVRLSNANAFDIFEDYDVILDCTDTPMARYLISDVAVNLGKPVVSASALRTEGQLSIFNFANSGPCYRCFYPTPPAPTSVSSCQEGGVLGPCVGLVGVAMVVETLKLLLGVYTIENFKPFLLQYSGFPDQTLRKFKMRGRREDCPASGSNKTVTRKAIESGEIDYHSFCGSRNYNVLKEDDRITVKKFEEEYRNSSNNKPYFLLDVRPSLHYSISHLPDTHNITVNELRDLQPELEELQTKIPHISKDSEVLVICRYGNDSQLATQLLKDKFKLKDVRDIKGGFFKYIDEINPSLPKY

>tr|A0A0L8RIC4|A0A0L8RIC4_SACEU/1440 Adenylyltransferase and sulfurtransferase UBA4 OS=Saccharomyces eubayanus OX=1080349 GN=UBA4 PE=3 SV=1MAEHQQGSTESELETLRLENAKLREQLARVQDNKKDYPLSLEEYQRYGRQMIVEETGGVVGQVKLKNTKVLVVGAGGLGCPALPYLAGAGVGQIGIVDNDVVETSNLHRQVLHDSSRVGMLKCESARQSITKLNPHVNVVTYPVRLNSTNAFEIFEGYDYVLDCTDSPLTRYLVSDVAVNLGITVVSASGLGTEGQLTILNFNSMGPCYRCFYPTPPPPNAVTSCQEGGVIGPCIGLVGTMMAVETLKLILGVYTNENFKPFLMLYSGFPQQSLRTFKMRGRQESCLCCGKNQTITKETIEKGEINYELFCGSRNYNVCQPDERLSVEAFQDLYKDNDGKTNQILVDVRPSHHYEISHFPEAINLPVKKLRDMNGDIKKLQEELPTVGKDTDIVVLCRYGNDSQVATRLLKDKFGLSNVRDVRGGYFKYIDDIDQNIPKY

>tr|A0A0L8VQ39|A0A0L8VQ39_9SACH/1440 Adenylyltransferase and sulfurtransferase UBA4 OS=Saccharomyces boulardii OX=252598 GN=UBA4 PE=3 SV=1MNDYHLEDTTSELEALRLENAQLREQLAKREDSSRDYPLSLEEYQRYGRQMIVEETGGVAGQVKLKNTKVLVVGAGGLGCPALPYLAGAGVGQIGIVDNDVVETSNLHRQVLHDSSRVGMLKCESARQYITKLNPHINVVTYPVRLNSSNAFDIFKGYNYILDCTDSPLTRYLVSDVAVNLGITVVSASGLGTEGQLTILNFNNIGPCYRCFYPTPPPPNAVTSCQEGGVIGPCIGLVGTMMAVETLKLILGIYTNENFSPFLMLYSGFPQQSLRTFKMRGRQEKCLCCGKNRTITKEAIEKGEINYELFCGARNYNVCEPDERISVDAFQRIYKDDEFLAKHIFLDVRPSHHYEISHFPEAVNIPIKNLRDMNGDLKKLQEKLPSVEKDSNIVILCRYGNDSQLATRLLKDKFGFSNVRDVRGGYFKYIDDIDQTIPKY

>tr|A0A0V1PRU0|A0A0V1PRU0_9ASCO/1447 Adenylyltransferase and sulfurtransferase UBA4 OS=Debaryomyces fabryi OX=58627 GN=UBA4 PE=3 SV=1MNDLSKDDLLERIRQLELENSQLKESISVLSNVSVQSEYDAKYPKIDECFSSDEYKRYGRQMIVPQFGSLKSQIKLKKNKVLVIGAGGLGCPALLYLSASGVGEIGIIDDDLVDISNLHRQVLHTTESVGIHKCESAKRYINKLNPHVKVNTYPFRLSNDNAFNIIEKYDLILDCTDTPATRYLINDVSVICGKTIVSGSGLKTDGQLSILNFHGIGPCYRCFYPKPPSPGSITSCADGGVVGPAIGLIGVTMALEAIKLITDFYTDETFKPFLSMYSGYPQQQLRVFKMRNKQQNCAVCGNKPTILKSTITNNDIDYAEFCGRVNPNVLASEFRISVDEYHDYVHSTNHNSILIDVRPKEQYEITKIPTSINIPWDPTINKADDIDSYFPPNFNKDTETFVICRYGNDSQIAAKKLIEKFGFSKVKDIKGGINKWSEEIDSSIPQY

>tr|A0A0W0D0X4|A0A0W0D0X4_CANGB/1433 Adenylyltransferase and sulfurtransferase UBA4 OS=Candida glabrata OX=5478 GN=UBA4 PE=3 SV=1MTDQDLLAQIELLKKENAQLKEKLKSQDDEQLSLEEYSRYGRQMIVEGTGGVVGQLRLKKAKVLVVGAGGLGSPSLPYLVGAGVGTIGIVDNDIVDTSNLHRQTIHNTAKVGMLKCESAKQVLKDLNPHVNINTYPVRLGPENAFSIFADYDIVMDCTDTPLTRYLISDVAVNLGKTVVSASGLGTEGQLTILNFNNIGPCYRCFYPVSPNPYAVSSCQEGGVIGPCIGLVGTMMAVETLKIIMGVYNNENFEPFLLSYSGFPIQSLRRFKMRGRQSKCQTCGDAPVITKEAIESGIIDYNIFCGSRNYNVCAEGERLTAKEFDENYGPEFSGNNKVLLDVRPSHHFDISHFNNAVNIPLKELRDMDGDISTLQSRIPNINKNSEVVVLCRYGNDSQLATRMLKDEFGIVNVKDVAGGFFKYIDDVDQSIPKY

>tr|A0A1E5RAJ7|A0A1E5RAJ7_9ASCO/1444 Adenylyltransferase and sulfurtransferase UBA4 OS=Hanseniaspora osmophila OX=56408 GN=UBA4 PE=3 SV=1MITTTTEEIFQLKKQIEELKTENAQLKKTNAEVQNEKALPLSISEYSRYGRQMICDVTNGVKGQLKLKNSKVLFIGAGGLACPALAYLAGAGVGTIGIVDDDVVENSNLHRQILHSSAKVGMLKCESAREFLTSLNPHIKIVTHPVRLSNDNAFEIFAKYDIILDCTDTPITRYLVSDVAVNLGKMVVSASGVQTEGQLCILNFKNIGPCYRCFYPTPPPPHAVSSCSAGGVIGPCIGLVGVMMAIETMKIILDVYTIENFKPFLLQYNGFPEQSLRNFKMRGRQAKCQSCGDTKTIDRTAIESGKINYFAFCGSKNYNVVSPEERMTVHEFETKYWNNNKKPRDSYVLLDVRPTHHYEISHLANTHNIALKDLQNLKGDMTKLQSKIKSISETSEVVVMCRHGNESRTATRLLKDEFGVANTKDVIGGFFKYVDEINPQLPKY

>tr|A0A1S7HCQ2|A0A1S7HCQ2_9SACH/1434 Adenylyltransferase and sulfurtransferase ZPAR0B06990_N OS=Zygosaccharomyces parabailii OX=1365886 GN=ZPAR0B06990_N PE=3 SV=1MSAPASDESLTELESLRLENAKLKKKLDEKQLDEYSMTLEEYTRYGRQMIVEGSGGVSGQLKLRSAKILVVGAGGLGCPALPYLAGAGVGHIGIIDNDIVDPSNLHRQVLHSTKKVGMLKCESAKQVLLELNPNVSVETYPIRLEGSNAFDIFEKYDYILDCTDTPMSRYLISDVAVILGKTVVSASGLGTEGQLTILNYNNIGPCYRCFYPKPPTPNSVSSCQEGGVVGPCIGLVGVMMAVEALKLIMGVYDNNFSPFLKLYSGFPEQTLRTFKMRGRQRNCSCCGENPSITKHAIESGVVSYTAFCGSRNYDVCNANERMSVAYFEEQYRKRKDFIFLDVRPSHHYSISHFPDTHNVPLKSLLNMKGSLSKLRHEIPNINPDSEVVVICRHGNESQLATRLLKDDFGIPDVKDVRGGFFKYIDEVDPSIPKY

>tr|A0A1S7HPI8|A0A1S7HPI8_9SACH/1435 Adenylyltransferase and sulfurtransferase ZPAR0H03000_N OS=Zygosaccharomyces parabailii OX=1365886 GN=ZPAR0H03000_N PE=3 SV=1MSVPASGGSLTELESLRLENDKLKKKLNEKQQLNECSMTLEEYTRYGRQMIVEGTGGVSGQLKLRSAKVLVVGAGGLGCPALPYLAGAGVGQIGIIDNDIVDSSNLHRQVLHSTKKVGMLKCESAKQVLLELNPNVSVETYPIRLEGSNAFDIFEKYDYILDCTDTPMSRYLISDVAVISGKTVVSASGLGTEGQLTILNYNNIGPCYRCFYPKPPTPNSVSSCQEGGVIGPCIGLVGVMMAVEALKLVMGVYDNNFSPFLKLYSGFPEQTLRTFKMRGRQRNCSCCGENPSITRHAIESGVVSYTAFCGSRNYDVCDANERMSVAYFEEQYRQRKDFILLDVRPSHHYSISHFPDTHNIPLKSLQNMKGSLSKLRHQIPNINPDSEVVVICRHGNESQLATRLLKDDFGIPDVKDIRGGFFKYIDEVDPSIPKY

>tr|A0A421JGR4|A0A421JGR4_9ASCO/1427 Adenylyltransferase and sulfurtransferase UBA4 OS=Meyerozyma sp. JA9 OX=2028340 GN=UBA4 PE=3 SV=1MSAPHLHELQRLRAENEQLKAELASKQHQPRQNDVKSYKGLSFEEHRRYGRQMIVSEFGSLQSQLKLKQCRTLVVGAGGLGCPSLMYLVGAGVGTIGIVDDDVVDETNLHRQVLHSTTSVGRLKCESAKSYLQTLNPNVNITTYPVRLSNKNAFEIFAHFDLVLDCTDAPASRYLINDVAVYFNIPVVSGSGLRTEGQLSVFNYENGPCYRCFYPDPPPANSVTSCSEGGVLGPVIGLLGTAMAVEAIKVITGFYHDNFKPFLTMYSAYPQQTFRVFKMRGRQKTCLCNHITKQAIESIDYSEFCGTLGPVNVLSDEHRISVHDYNSARNSDHVLVDVRPKEQFEVSTFPGAVNIPWDSVLSKTTTIDNIDNVDNVELEKPIYVVCRYGNDSQLATKKLLEMGYNAKDIRGGVSRWYAEVDQNIPFY

>tr|A0A421JR90|A0A421JR90_9ASCO/1441 Adenylyltransferase and sulfurtransferase UBA4 OS=Spathaspora sp. JA1 OX=2028339 GN=UBA4 PE=3 SV=1MTAPTTKELLERIAALELENSRLRDNQTPQQELPKPSSSSNTTNTLSLEEYKRYGRQMIVPQFGSLPSQLRLKQSKILVVGAGGLGCPALLYLSAAGIGTIGIVDDDVVDISNLHRQVLHTSDNVGMHKCESAKRYITRLNPHVDVETYPMRLNNDNAFSIISKYDLVLDCTDHPAIRYLINDVCVILNKTIVSGSGLKSDGQLSVLNFQTQGPCYRCFYPQPPSPDSVTSCSDGGVIGPAIGLVGTSMAVETIKILTGYYTSDNFKPFLSVYSGYPYQQFRMFKMRGKQKNCVVCGENPSVTREKIESNELNYSVFCGRVTFPTLENQYRISPSEYNQSFQNKEKHVLIDVRPKEQFSITSLPNSVNIEWDPIFRKANSLTEYLPAGTSKDDSIVVVCRYGNDSQLAVRKLQDLHYSNVKDIIGGLSKWSEDVDQSIPQY

>tr|A0A642UTY7|A0A642UTY7_DIURU/1422 Adenylyltransferase and sulfurtransferase UBA4 OS=Diutina rugosa OX=5481 GN=UBA4 PE=3 SV=1MDELTRLREENQRLKQQLAAQQQSQTNGTHTESSSTPQTELCAEEYLRYGRQMITPHFHSLQGQLRLRHTKVLVVGAGGLGCPAIAYLAGAGVGKLGIIDGDTVDESNLHRQILHTTAGQLKSESAQQFVDRLNPHVATEIYPFRLSATNAFNLFSGYDVILDCTDTPATRYLINDVAVLLGKPLVSGSGLKTEGQFTVLNFNNTGPCYRCFHPKPPPPDSVTSCSDGGVIGPVIGLVGTAMATEAIKVITGYYTADNFKPFLAAYSAYPFQQLRHFKMRPRKPDCAVCSAQATITRQSIEAGEIDYVAYCGRVTFDPLPADMRINANQFNKESVIIDVRPKEQFDITKIDGAINVPITDLSQANVDNLQLDHSAPITVVCRFGNDSQIAVQKLRDWGYTDAVDVIGGLKRWRQTYGDVVDY

>tr|A0A642V817|A0A642V817_9ASCO/1416 Adenylyltransferase and sulfurtransferase UBA4 OS=Trichomonascus ciferrii OX=44093 GN=UBA4 PE=3 SV=1MSTELEALRKENERLRRELERREENEKLSLEEYRRYGRQLIMGEIGIRGQLRLKRARVLVVGAGGLGCPVLAYLGGAGVGTIGIVDNDTVDASNIHRQILHDTTKVGLSKAESARQFLHNQNDNVNVQVHNTRLLPDNVFEIFQQYDLVLDCTDTPNTRYLISDAAVILGLPLVSASALKTEGQLSVLNFKNGPCYRCFFPVPPPPDSVLSCGDGGILGPVVGVMGVMQSIEAIKVITGAYEDRQEFKPSLTLFSAYSMPQWRSIRMRGKKPDCATCGNAPTITKQSIESGETDYTAFCGLPSQVTLSPTERVSVKQYAELRDKPHTLIDVREKPQFEICSLEGALNKPYMGLKSGKVNFEEGELQEPVYVCCRYGNDSQATVKILKEQYNLQGVYDIEGGLDKWSQDVDEEFPRY

>tr|A0A6C1DTS1|A0A6C1DTS1_SACPS/1440 Adenylyltransferase and sulfurtransferase UBA4_1 OS=Saccharomyces pastorianus OX=27292 GN=UBA4_1 PE=3 SV=1MNDYHLEDTTSELEALRLENAQLREQLAKREDSSRDYPLSLEEYQRYGRQMIVEETGGVAGQVKLKNTKVLVVGAGGLGCPALPYLAGAGVGQIGIVDNDVVETSNLHRQVLHDSSRVGMLKCESARQYITKLNPHINVVTYPVRLNSSNAFDIFKGYNYILDCTDSPLTRYLVSDVAVNLGITVVSASGLGTEGQLTILNFNNIGPCYRCFYPTPPPPNAVTSCQEGGVIGPCIGLVGTMMAVETLKLILGIYTNENFSPFLMLYSGFPQQSLRTFKMRGRQEKCLCCGKNRTITKEAIEKGEINYELFCGARNYNVCEPDERISVDAFQRIYKDDEFLAKHIFLDVRPSHHYEISHFPEAVNIPIKNLRDMNGDLKKLQEKLPSVEKDSNIVILCRYGNDSQLATRLLKDKFGFSNVRDVRGGYFKYIDDIDQTIPKY

>tr|A0A6C1EGT1|A0A6C1EGT1_SACPS/1440 Adenylyltransferase and sulfurtransferase UBA4_2 OS=Saccharomyces pastorianus OX=27292 GN=UBA4_2 PE=3 SV=1MAEHQQGSTESELETLRLENAKLREQLARVQDNKKDYPLSLEEYQRYGRQMIVEETGGVVGQVKLKNTKVLVVGAGGLGCPALPYLAGAGVGQIGIVDNDVVETSNLHRQVLHDSSRVGMLKCESARQSITKLNPHVNVVTYPVRLNSTNAFEIFEGYDYVLDCTDSPLTRYLVSDVAVNLGITVVSASGLGTEGQLTILNFNRMGPCYRCFYPTPPPPNAVTSCQEGGVIGPCIGLVGTMMAVETLKLILGVYTNENFKPFLMLYSGFPQQSLRTFKMRGRQESCLCCGKNRTITKETIEKGEINYELFCGSRNYNVCQPDERLSVEAFQDLYKDNDRKTNHILVDVRPSHHYEISHFPEAINLPVKKLRDMNGDIKKLQEELPTVGKDTDIVVLCRYGNDSQVATRLLKDKFGLSNVRDVRGGYFKYINDIDQNIPKY

>sp|A3LQF9|UBA4_PICST/1443 Adenylyltransferase and sulfurtransferase UBA4 OS=Scheffersomyces stipitis (strain ATCC 58785 / CBS 6054 / NBRC 10063 / NRRL Y11545) OX=322104 GN=UBA4 PE=3 SV=2MAETNSREEELLRRIQELELENEVLKKKVAEVEYDAKYPKIDENFCLDEYKRYGRQMIVPEFGSLKSQIKLKNSSILVVGAGGLGCPALLYLSAAGIGKIGIVDDDIVDISNLHRQVLHTTDSIGMFKCDSAKKYICKLNPHVIVKTYPVRLHNDNAFDIVNDYDIVLDCTDTPAIRYLINDVSVLCRKTIVSGSGLKSDGQLSILNFNNEGPCYRCFYPKPPSAESITTCSDGGVIGPCIGLLGVSMAVETIKVLTGFYTKDNFKPFLSMYTGYPQQSFRVFKMRGRSDKCSVCGSFPTVSKEAILNNEIDYVAFCGKVDSNVLTPEDRITVQQFSDIVTRQSKAPVLLDVRTKEQFQIAKLPNSINIEWDPTFKKLESLDKYLPEDFDKDTDPVFVVCRYGNDSQLATRKMKQELDFKNAKDIIGGLNKWSDIVNPKFPKY

>sp|A5DMB6|UBA4_PICGU/1424 Adenylyltransferase and sulfurtransferase UBA4 OS=Meyerozyma guilliermondii (strain ATCC 6260 / CBS 566 / DSM 6381 / JCM 1539 / NBRC 10279 / NRRL Y324) OX=294746 GN=UBA4 PE=3 SV=1MDLHELERLRAENEQLKAELASKQHQIKQNDIKSYKGLSFEEHRRYGRQMIVSEFGSLTSQLKLKQCKTLVVGAGGLGCPSLMYLVGAGVGTIGIVDDDLVDETNLHRQVLHSTTSVGRLKCESAKSYLQELNPNVNIITYPVRLSNKNAFEIFADFDLVLDCTDSPASRYLINDVAVYFNIPVVSGSGLRTEGQLSVFNYENGPCYRCFYPDPPAANSVTSCSEGGVLGPVIGLLGTAMAVEAIKVITGFYHNTFKPFLTMYSAYPQQTFRVFKMRGKQKTCLCNHITRQAIESIDYSEFCGTLGPVNLLSHDQRISVHDYNSVRNSDHVLLDVRPKEQFEVSSFPGAVNIPWDSVLSKTTNIDKIDQLQLPPKSPIYVVCRYGNDSQLATKKLLDMGWNNVKDIKGGVSRWYSEVDQNIPFY

>sp|A5DSR2|UBA4_LODEL/1455 Adenylyltransferase and sulfurtransferase UBA4 OS=Lodderomyces elongisporus (strain ATCC 11503 / CBS 2605 / JCM 1781 / NBRC 1676 / NRRL YB4239) OX=379508 GN=UBA4 PE=3 SV=1MTDLSKEELLQRIQQLENENEQLKAVALQSLHTLRYNQSCSHSLQQSNSVNEEPLSLEEFKRYGRQMIVPKFGSLNAQKKLRSSKILVVGAGGLGSPALQYLCAAGIGEIGIIDDDTVDVSNLHRQIIHKSSLVGILKCESAKQSMKDLNPFVKVETYPERLTVFNAFEIIDKYDLVLDCTDHPAVRYLINDVCVLLGKTIVSGSGLRAEGQLTILNYDQVGPCYRCFYPQAPEPSSITSCSDGGVIGPAIGLVGVAMAMETIKLLTGTYTRENFTPFLASYSAYPLQQMKTFKMRPKQSSCKVCGDRPEITKEMVENGSIDYVSFCGHIDEKNPPLQKKYRITVQEYSSLLNSQSREHTLIDVRPKEQFEITNLPGSINLDWPLVFSKCDNDKIDLLLPQDITKADQLYVICRFGNDSQLATAKLIEAGYLNAKDIIGGLNKWSEDIDAAFPKY

>sp|A6ZT19|UBA4_YEAS7/1440 Adenylyltransferase and sulfurtransferase UBA4 OS=Saccharomyces cerevisiae (strain YJM789) OX=307796 GN=UBA4 PE=3 SV=1MNDYHLEDTTSELEALRLENAQLREQLAKREDSSRDYPLSLEEYQRYGRQMIVEETGGVAGQVKLKNTRVLVVGAGGLGCPALPYLAGAGVGQIGIVDNDVVETSNLHRQVLHDSSRVGMLKCESARQYITKLNPHINVVTYPVRLNSSNAFDIFKGYNYILDCTDSPLTRYLVSDVAVNLGITVVSASGLGTEGQLTILNFNNIGPCYRCFYPTPPPPNAVTSCQEGGVIGPCIGLVGTMMAVETLKLILGIYTNENFSPFLMLYSGFPQQSLRTFKMRGRQEKCLCCGKNRTITKEAIEKGEINYELFCGARNYNVCEPDERISVDAFQRIYKDDEFLAKHIFLDVRPSHHYEISHFPEAVNIPIKNLRDMNGDLKKLQEKLPSVEKDSNIVILCRYGNDSQLATRLLKDKFGFSNVRDVRGGYFKYIDDIDQTIPKY

>sp|A7THV5|UBA4_VANPO/1436 Adenylyltransferase and sulfurtransferase UBA4 OS=Vanderwaltozyma polyspora (strain ATCC 22028 / DSM 70294 / BCRC 21397 / CBS 2163 / NBRC 10782 / NRRL Y8283 / UCD 5717) OX=436907 GN=UBA4 PE=3 SV=1MVEVSAEVLAELELLKKENAKLRSQLDSQKSDKLPMSLQEFQRYGRQMIVEETSGIEGQLKLKNSKVLVIGAGGLGCPALLYLAGAGIGTIGIVDNDTVDNSNLHRQVLHDSSKVGMLKCESARQRINLLNPHVNVKTYPVRLDYSNAFTIFENYDYVLDCTDTPITRYLVSDVAVNLGMTVISASGLGSEGQLSILNFKNEGPCYRCFYPTPPPPNSVSSCQEGGVIGPCIGLVGTMMAVETLKVIYDIYTLENFKPFLMMYSGFPNQTLRTFKMRGRQNNCECCGLDPKITRAAIESGLVNYFEFCGSRNYNLCTDEERISSEIYKTEFIDCNEKDHILIDVRPRHHFNISHFNHAINIPVKELKGMKGSLDILKESVPNVSQDSKVIVLCRYGNDSQIATRLLKDEFKINDVKDVKGGFFKYIDEEDHSYPKY

>sp|B3LSM6|UBA4_YEAS1/1440 Adenylyltransferase and sulfurtransferase UBA4 OS=Saccharomyces cerevisiae (strain RM111a) OX=285006 GN=UBA4 PE=3 SV=1MNDYHLEDTTSELEALRLENAQLREQLAKREDSSRDYPLSLEEYQRYGRQMIVEETGGVAGQVKLKNTKVLVVGAGGLGCPALPYLAGAGVGQIGIVDNDVVETSNLHRQVLHDSSRVGMLKCESARQYITKLNPHINVVTYPVRLNSSNAFDIFKGYNYILDCTDSPLTRYLVSDVAVNLGITVVSASGLGTEGQLTILNFNNIGPCYRCFYPTPPPPNAVTSCQEGGVIGPCIGLVGTMMAVETLKLILGIYTNENFSPFLMLYSGFPQQSLRTFKMRGRQEKCLCCGKNRTITKEAIEKGEINYELFCGARNYNVCEPDERISVDAFQRIYKDDEFLAKHIFLDVRPSHHYEISHFPEAVNIPIKNLRDMNGDLKKLQEKLPSVEKDSNIVILCRYGNDSQLATRLLKDKFGFSNVRDVRGGYFKYIDDIDQTIPKY

>sp|B5VK45|UBA4_YEAS6/1440 Adenylyltransferase and sulfurtransferase UBA4 OS=Saccharomyces cerevisiae (strain AWRI1631) OX=545124 GN=UBA4 PE=3 SV=1MNDYHLEDTTSELEALRLENAQLREQLAKREDSSRDYPLSLEEYQRYGRQMIVEETGGVAGQVKLKNTKVLVVGAGGLGCPALPYLAGAGVGQIGIVDNDVVETSNLHRQVLHDSSRVGMLKCESARQYITKLNPHINVVTYPVRLNSSNAFDIFKGYNYILDCTDSPLTRYLVSDVAVNLGITVVSASGLGTEGQLTILNFNNIGPCYRCFYPTPPPPNAVTSCQEGGVIGPCIGLVGTMMAVETLKLILGIYTNENFSPFLMLYSGFPQQSLRTFKMRGRQEKCLCCGKNRTITKEAIEKGEINYELFCGARNYNVCEPDERISVDAFQRIYKDDEFLAKHIFLDVRPSHHYEISHFPEAVNIPIKNLRDMNGDLKKLQEKLPSVEKDSNIVILCRYGNDSQLATRLLKDKFGFSNVRDVRGGYFKYIDDIDQTIPKY

>tr|G0VH39|G0VH39_NAUCC/1440 Adenylyltransferase and sulfurtransferase NCAS0F03270 OS=Naumovozyma castellii (strain ATCC 76901 / BCRC 22586 / CBS 4309 / NBRC 1992 / NRRL Y12630) OX=1064592 GN=NCAS0F03270 PE=3 SV=1MSEVDAEMRAELAALKLENAVLRKQLASNNNKNGTSDSPHPLSLEEYRRYGRQMIVEDIGGLKSQLKLKAAKVLVIGAGGLGCPALPYLTGAGVGTIGIVDNDVVDTSNLHRQVLHDSTKVGMLKCESAKQVLNKLNPHVEIVTYPVRLDYSNAFEIFEKYDCVLDCTDTPLTRYLISDVAVNVGIPVVSASGVGTEGQLTILNFQNEGPCYRCFFPTPPPPTSVSSCSEGGVIGPCIGLVGTMMAIETIKVIIGSYTSENFKPFLMMYSGFPQQTLRTFKMRGRQSKCLTCGDHPTINKEAIQSGSINYELFCGSRNYNVCEPDERMSVQDFEKHREGPNDVILLDVRPSHHYEISHFANTFNIPVKKLRNMDGSMAKLQDEIPRIKQDSEVVVLCRYGNDSQIATRLLKDEFKIPNVRDVKGGFFKYIDEIDPSIPKY

>tr|G0W729|G0W729_NAUDC/1449 Adenylyltransferase and sulfurtransferase NDAI0B05570 OS=Naumovozyma dairenensis (strain ATCC 10597 / BCRC 20456 / CBS 421 / NBRC 0211 / NRRL Y12639) OX=1071378 GN=NDAI0B05570 PE=3 SV=1MSDQELLAQIRALKLENEALKVRLSNTPLPPSPNPLSLEEYNRYGRQMIVDMPSPIESGLPAQLKLKSAKILIIGAGGLGSPALLYLAGAGVGTIGIVDNDIVDTSNLHRQIIHDSHRVNMLKCESAKLSIQNLNPNVNVVTYPTRLTHSNAFEIFEGYDLILDCTDTPLTRYLISDVAVKLGITVVSGSGVGTEAQLTILNFMNMGPCYRCFFPVPPPPTSVASCSEGGVIGPCIGVMGVMMAVEAIKFIVGAYTVENFEPYLVLYSGFPGTSSGQSMRTFKMRRRQKNCACCSNEPSITKESIESGSINYELFCGSRNYNVCQADERISVHEFDSAFSPRDTERAVKENLIMLDVRPNHHYNISHLPDTVNIPLKVLKNMNGSMELLQEVIPTVNTESEVIVLCRYGNDSQLATRLLKDEFKIGNVKDVKGGFFKYIDELDTTIPKY

>tr|G2WFI5|G2WFI5_YEASK/1440 Adenylyltransferase and sulfurtransferase K7_UBA4 OS=Saccharomyces cerevisiae (strain Kyokai no. 7 / NBRC 101557) OX=721032 GN=K7_UBA4 PE=3 SV=1MNDYHLEDTTSELEALRLENAQLREQLAKREDSSRDYPLSLEEYQRYGRQMIVEETGGVAGQVKLKNTKVLVVGAGGLGCPALPYLAGAGVGQIGIVDNDVVETSNLHRQVLHDSSRVGMLKCESARQYITKLNPHINVVTYPVRLNSSNAFDIFKGYNYILDCTDSPLTRYLVSDVAVNLGITVVSASGLGTEGQLTILNFNNIGPCYRCFYPTPPPPNAVTSCQEGGVIGPCIGLVGTMMAVETLKLILGIYTNENFSPFLMLYSGFPQQSLRTFKMRGRQEKCLCCGKNRTITKEAIEKGEINYELFCGARNYNVCEPDERISVDAFQRIYKDDEFLAKHIFIDVRPSHHYEISHFPEAVNIPIKNLRDMNGDLKKLQEKLPSVEKDSNIVILCRYGNDSQLATRLLKDKFGFSNVRDVRGGYFKYIDDIDQTIPKY

>tr|G8BX75|G8BX75_TETPH/1432 Adenylyltransferase and sulfurtransferase TPHA0H01730 OS=Tetrapisispora phaffii (strain ATCC 24235 / CBS 4417 / NBRC 1672 / NRRL Y8282 / UCD 705) OX=1071381 GN=TPHA0H01730 PE=3 SV=1MEQLRKEIENLREENAKLRSQLSESKVNNESLSLTEYQRYGRQMIVEESGGLRGQLELKSTRIVVVGAGGLGCPSLQYLVGAGVGHIGIVDNDTVEASNLHRQTLHDTSRVGTLKCESAKQKLNLLNPFVDIKTYPVRLTSENAFEIFDGYNYVLDCTDSPATRYLVNDVAVSLGITVISASGVGTEGQLCILNFKNQGPCYRCFYPTPPPPNSVSSCSEGGVIGPCIGLVGVMMAIETLKLIYGVYTIENFKPFLMSYSGFPQQSLRVFKMRGKQTTCKCCGSDPKITKETIESGEINYTEFCGQRNYNVCSADERITVKQYHSEYIESLEKDHILIDVRPSHHYDISHFQHSINIPLKELKNMHGSLDKLKERIPQLQENTNLVVLCRFGNDSQLATRIFKDEFKIKSVKDVKGGFFKYIDNINQDIPKY

>tr|G8ZV02|G8ZV02_TORDC/1437 Adenylyltransferase and sulfurtransferase TDEL0E02030 OS=Torulaspora delbrueckii (strain ATCC 10662 / CBS 1146 / NBRC 0425 / NCYC 2629 / NRRL Y866) OX=1076872 GN=TDEL0E02030 PE=3 SV=1MEVSEELRRELTALKLENARLRRQLEGKEASENKYPLSLEEYKRYGRQMIVEETGGTLGQVKLRNSRVLVVGAGGLGSPVLPYLAGAGVGHIGIVDNDVVDTSNLHRQVLHDSESVGTLKCESAKRALNRLNPLVEVITYPVRLSNTNAFEIFRSYDYILDCTDTPLTRYLISDVAVNLGITVVSASGLGTEGQLCILNFKKLGPCYRCFYPKPPTPTSVTSCQEGGVVGPCIGLVGVMMAVETLKLIYGVYAEDNFQPFLTTYSGFPNQSMRTFKMRGRQDNCACCGLSPIVTKDAIESGTINYQNFCGSRSYDCCSLDERISVELFEDQYKNREPSSYILLDVRPHHHYDISHIPQAYNLTVKELRDMDGELTKIQKHIPNINKDSEVVVMCRYGNDSRLATRLLKDQFGISNVKDVTGGLFRYIDRIDPSIPKY

>tr|H2AVZ7|H2AVZ7_KAZAF/1439 Adenylyltransferase and sulfurtransferase KAFR0E03960 OS=Kazachstania africana (strain ATCC 22294 / BCRC 22015 / CBS 2517 / CECT 1963 / NBRC 1671 / NRRL Y8276) OX=1071382 GN=KAFR0E03960 PE=3 SV=1MVSEVNQDLINELAALKLENARLRRQVESQPTTVQEYPLSLEEYRRYGRQMIVEDTGGVQGQVRLKNSKVLVVGAGGLGCPALPYLAGAGIGQIGIVDNDIVDTSNLHRQVLHDSTKVGMLKCESAKAVLNKLNPHVNVVTYPVRLNYSNAFDIFDGYDYVLDCTDTPLTRYLISDVAVNLGITVVSASGLGTEGQLTVLNFNNTGPCYRCFYPIPPNPMSVSSCQEGGVIGPCIGLVGTMMAVEAIKLIIGIYTPENFQPFLTLYSGFPQQTLRTFKMRGRQSKCICCGDNRSITRAAIESGEINYELFCGSRNYNVCSPDERITVNKFKDEYYDRTTQDYVLLDVRPSHHYEISHFPDAFNIPVKKLKDMHGSMAELQKEIPSIKEDSEVLVLCRYGNDSQLATRLLKDEFKLPNVKDIQGGFFRYIDEVDSSIPKY

>tr|I2GVL3|I2GVL3_TETBL/1441 Adenylyltransferase and sulfurtransferase TBLA0A03670 OS=Tetrapisispora blattae (strain ATCC 34711 / CBS 6284 / DSM 70876 / NBRC 10599 / NRRL Y10934 / UCD 777) OX=1071380 GN=TBLA0A03670 PE=3 SV=1MGDMSISVTEELERVKKENELLRKELNELKKESKHDHKELSLEEYRRYGRQMIVEDFGGLEGQKKLKNANVLVVGAGGLGSPSLPYLVGAGVGHIGIVDNDVVDSSNLHRQVLHDTTKVGVFKCESAKEQLSKLNPNIKIDTYSVRLDAFNAFEIFKDYDVILDCTDTPLARYLISDVAVNLGKTVVSASGLGTEGQVTILNFKNIGPCYRCFYPTPPNPYAVNSCSEGGVIGPCIGLVGTMMAVETLKVLLDIYTIENFKPFLMLYSGFPNQTLRNFKMRGRQSSCQCCGKNPTITKNTIESGEINYSLFCGKRNYNVLEAHERMNVSQFHENYIVNSPSNQVILLDVRPPHHFGISHFDNAINISVPQLTKLNGSFSELQKKLPSLNKNSEVVVLCRYGNDSQVATRMLKDNFGLKNVKDVQGGFFKYIDDIDNSIPQY

>tr|J7RX32|J7RX32_KAZNA/1438 Adenylyltransferase and sulfurtransferase KNAG0C05010 OS=Kazachstania naganishii (strain ATCC MYA139 / BCRC 22969 / CBS 8797 / KCTC 17520 / NBRC 10181 / NCYC 3082 / Yp74L3) OX=1071383 GN=KNAG0C05010 PE=3 SV=1MSGNSSDAQAELIRLRRENELLREKLRQSGTQEGVGADRLSLPEYERYGRQMIVESTGGVQGQLLLKSAKVLVVGAGGLGSPCLPYLAGAGVGTIGIVDNDTVEVSNLHRQVIHDSGKVGMLKCESAREVLGKLNPNVKVVTYPVRLDYSNAFRIFEGYDIVLDCTDTPLTRYLVSDVAVNLGMTVVSASGLGTEGQLSVLNFQDCGPCYRCFYPTPPPPTSVASCSEGGVIGPCIGIVGVMMAVETIKIILGVYTKENFKPFLMMYSGFPGQTIRTFKMRGRQPQCIACGTERTITKETIESGIVNYNVFCGSRNYNVCQPEERISVSDLQDELLDMDTTVLLDVRPSHHYKISHLPHTVNVPMKELRNLDRSVDALQSRIPQITESSDVLVICRHGNESQLATRLLKDEFNIKNVRDVTGGLFKYIDEIDPSMPKY

>tr|J8PMX2|J8PMX2_SACAR/1440 Adenylyltransferase and sulfurtransferase UBA4 OS=Saccharomyces arboricola (strain H6 / AS 2.3317 / CBS 10644) OX=1160507 GN=UBA4 PE=3 SV=1MSDHQSRSIESELEALRLENAQLREQLASRKDITQDYPLSLEEYQRYGRQMIVEETGGVAGQVKLKNAKILVVGAGGLGCPALPYLAGAGVGRIGIVDNDVVETSNLHRQVLHNSERVGMLKCESASQSITKLNPHVNVVTYPVRLNSSNAFGIFEGYDYILDCTDSPLTRYLISDVAVNLGITVVSASGLGTEGQLTILNFNNMGPCYRCFYPTPPPPNAVTSCQEGGVIGPCIGLVGTMMAVETLKLILGIYTNENFKPFLMLYSGFPQQSLRTFKMRGRKDNCLCCGENRTITREAIEKGEINYELFCGSRNYNVCQPDERLSVSEFQDLYKGDGCLENSYIIDVRPSHHYEISHFSETINIPVKKLRDMNGDIKKLQEKLPTVGKDSNIVVLCRYGNDSQVATRLLKDKFGFSKVRDVRGGYFKYIDDIDQTIPKY

>sp|Q59WH7|UBA4_CANAL/1438 Adenylyltransferase and sulfurtransferase UBA4 OS=Candida albicans (strain SC5314 / ATCC MYA2876) OX=237561 GN=UBA4 PE=3 SV=1MSEPSKEELLARIAQLELENEQLKQQNGKKSQHEQFNKIDDNFSLDEYKRYGRQMIVPQFGSLESQIKLKNSKVLVVGAGGLGSPALLYLSSAGIGKIGIIDPDTVDTSNLHRQVIHNTEMVGEFKCISAQNYINKLNPHVVVEVYPTALNNDNAFGIVSQYDLVLDCTDHPAVRYLINDVCVLLGKTIVSGSGLKSDGQLTVLNFANSGPCYRCFYPQPPSPDSVTSCSDGGVIGPAIGLVGVAMAVETIKIITGYYTKDNFVPFLASYSAYPQQQLRVFKMRKRQKDCAVCGENPQISQRMIEDGTINYKTFCGRATFDPIDDKFRVSPKDYDSVVQNKKKHILIDVRPREQFQITHLPNAINVQWDPTFRKADAIEQYLPDDSTKDDEIYVVCRFGNDSQLAAKKLIGMGYPNVRDIIGGLDKWSDDVDSKIPKY

>sp|Q6BHZ2|UBA4_DEBHA/1448 Adenylyltransferase and sulfurtransferase UBA4 OS=Debaryomyces hansenii (strain ATCC 36239 / CBS 767 / BCRC 21394 / JCM 1990 / NBRC 0083 / IGC 2968) OX=284592 GN=UBA4 PE=3 SV=1MNDLSREDLLQKIRLLELENAKLKESKSAVSTVSDQYEYDAKYPKIDEYFSSDEYKRYGRQMIVPQFGSLISQVKLKKSKVLFIGAGGLGCPALLYLSASGVGEIGIIDDDLVDISNLHRQVLHTTESVGIHKCESAKRYINKLNPHVKVNTYPFRLSNDNAFDIIEKYDLILDCTDTPATRYLINDVSVICGKTIVSGSGLKTDGQLSILNFHGIGPCYRCFYPKPPSPGSVTSCSDGGVVGPAIGLIGITMALEAIKVITDFYTDETFKPFLSMYSGYPQQQIRVFKMRNKQANCAVCGNNPTVLKSTISDNDIDYAEFCGRVNPNVLAPELRISVQEYHNYINSSQGENSILIDVRPKEQYEITKLPNSINIAWDPTFIKADNIDSYLPSNFDKNTNTFVMCRYGNDSQMATKKLIENFGFNEVKDIKGGINKWSKEIDSKIPQY

>sp|Q6CMC2|UBA4_KLULA/1444 Adenylyltransferase and sulfurtransferase UBA4 OS=Kluyveromyces lactis (strain ATCC 8585 / CBS 2359 / DSM 70799 / NBRC 1267 / NRRL Y1140 / WM37) OX=284590 GN=UBA4 PE=3 SV=1MTETERSNESLLEEIENLRVENAKLKNDLCLERSLNNASDLPMSLDEFKRYGRQMIVDETEGLMGQLKLKNASVLVIGAGGLGCPSLPYLAGAGIGKIGIVDNDTVDTSNLHRQVLHDTIKVGMLKSESAKQVLNKLNPHVSVTSYPVRLSNENAFDIFKDYDVILDCTDTPMARYLISDVAVNLGKPVVSASALRTEGQLSIFNFDNVGPCYRCFYPTPPAPTSVSSCQEGGVLGPCVGLVGVAMVVEALKLLLGVYTRENFKPFLLQYSGFPDQTLRKFKMRGRRVDCPACGTGRTVTRESIESGKINYQSFCGSRNYSVLTEDERIDVHKFERDYWNSSKTKPYVLLDVRPSLHYSISHLPNSHNITVNELRDLPADLNNLQSKIPHLSADSEVLVLCRYGNDSQLATRLLKDKFNLKDVKDVKGGFFKYIDEINPSLPKY

>sp|Q6FR35|UBA4_CANGA/1433 Adenylyltransferase and sulfurtransferase UBA4 OS=Candida glabrata (strain ATCC 2001 / CBS 138 / JCM 3761 / NBRC 0622 / NRRL Y65) OX=284593 GN=UBA4 PE=3 SV=1MTDQDLLAQIELLKKENAQLKEKLKSQDDEQLSLEEYSRYGRQMIVEGTGGVVGQLRLKKAKVLVVGAGGLGSPSLPYLVGAGVGTIGIVDNDIVDTSNLHRQTIHNTAKVGMLKCESAKQVLKDLNPHVNINTYPVRLGPENAFSIFADYDIVMDCTDTPLTRYLISDVAVNLGKTVVSASGLGTEGQLTILNFNNIGPCYRCFYPVSPNPYAVSSCQEGGVIGPCIGLVGTMMAVETLKIIMGVYNNENFEPFLLSYSGFPIQSLRRFKMRGRQSKCQTCGDAPVITKEAIESGIIDYNIFCGSRNYNVCAEGERLTAKEFDENYGPEFSGNNKVLLDVRPSHHFDISHFNNAVNIPLKELRDMDGDISTLQSRIPNINKNSEVVVLCRYGNDSQLATRMLKDEFGITNVKDVAGGFFKYIDDVDQSIPKY

>sp|Q756K6|UBA4_ASHGO/1443 Adenylyltransferase and sulfurtransferase UBA4 OS=Ashbya gossypii (strain ATCC 10895 / CBS 109.51 / FGSC 9923 / NRRL Y1056) OX=284811 GN=UBA4 PE=3 SV=1MTGESLDGSLHALTIELDALRRENANLKQQLKEKDGACGELPMSLEEFQRYGRQMIVGETGGLSGQVKLRSARVLIVGAGGLGCPALQYLAGAGIGHLGIVDNDVVEESNLHRQPLHDTSKVGLLKCDSAKEALSRLNPYCSIKTYPVRLSYANAFEIFPSWDLILDCTDSPMSRYLISDVAVNLGKTVVSGSGLGTEGQLSIYNFENKGPCYRCFYPIPPRPGSVVSCQSGGVLGPCIGVLGIMMAVEALKILFGIYTLENFKPFLMQYSGFPYQTLRMFKMRNRKQGCLCCGDNPTITKSTIESGHIKYEAFCGAINYDVLSKDERLSASEFEANYWSQKERGFVCLDVRPRLHYEISHLPGTYNMTVKELDEMEGSIEELQKHIPVITPDLDIVVLCRYGNDSRLATRILKDKFKLRNVRDVKGGYFAYIDEINPSLPKY

>tr|R9XG71|R9XG71_ASHAC/1443 Adenylyltransferase and sulfurtransferase UBA4 OS=Ashbya aceri OX=566037 GN=UBA4 PE=3 SV=1MTQAGVDGNLDALTNELDALRLENERLKRQLQERETSGGGLPMSLEEFQRYGRQMIVEETGGISGQVKLRNARVLVVGAGGLGCPALQYLAGAGIGHLGIVDDDIVDASNLHRQPLHDTCKVGLLKCDSAKEALLRLNPHCNIKTYAVRLSYANAFGIFSSWDLIMDCTDSPMSRYLISDVAVNLGKTVVSGSGLGTEGQLSIYNFKNAGPCYRCFYPIPPPPGSVVSCQMGGVLGPCIGMLGVMMAVETLKILFDVYTLENFKPFLMQYSGFPYQTLRMFKMRGRKQNCLCCGDKPTITRSMIESGHIEYETFCGAVDYDVLSKDERLSASEFEANYWSQKGKDFVCLDVRPPLHYEISHLPGTHNMTVKELDDMEGNIEELQKRIPAITSDRDIVVLCRYGNDSRLATRILKDKFKLMNVRDIKGGYFAYIDEINPSLPKY

>tr|W0T785|W0T785_KLUMD/1444 Adenylyltransferase and sulfurtransferase UBA4 OS=Kluyveromyces marxianus (strain DMKU31042 / BCC 29191 / NBRC 104275) OX=1003335 GN=UBA4 PE=3 SV=1MTNINGDTSSLLEELERLREENARLKKTLDEQRHHQDDLSLPMSLEEFQRYGRQMIVEETHGLSGQIKLKNAKVLVIGAGGLGCPSLPYLAGAGIGTIGIVDNDLVDTSNLHRQVLHDSTKVGMLKCESAKLVLNKLNPHVSVITYPVRLTNSNAFQIFEGYDLVLDCTDTPVTRYLVSDVAVNLNIPVVSASALRTEGQLSIFNFANIGPCYRCFYPNPPTPTSVSSCQEGGVLGPCVGLVGVAMVVETLKILLGTYTVENFKPFLLQYSGFPDQTLRKFKMRGRKSDCLACGANRIVTRQSIESGEINYETFCGSRHYNVLTEEERVDVFEFEKRYKMKAEDKSFLLLDVRPSLHYSISHLPDTHNITVNELRDMKSDFDALKSRIPSIDADSEVFVICRYGNDSQLATRLLKEKFKIKNVKDVKGGFFKYIDDINPALPKY

>tr|A0A061BHQ2|A0A061BHQ2_CYBFA/1429 Adenylyltransferase and sulfurtransferase UBA4 OS=Cyberlindnera fabianii OX=36022 GN=UBA4 PE=3 SV=1MSQEDLLEELTRLRAENASLRAQVSSQHHTEPTNTSKLVNGLTHDEYKRYGRQMIVPQVGFTGQLSLKHSKVLVVGAGGLGCPALMYLAGCGIGKIGIVDGDTVDVSNLHRQVMHDSTTVGMMKVESARLFINRLNPGVEVRTYAERLSTDNAFDIFEEWDLVLDCTDSPYSRYLISDVAVVLGKTVVSGSGLKTEGQLSILNFENVGPCYRCFYPTPPPPNSVTSCTDGGVIGPVIGLMGVMMALEAIKIITGTYTVENFKPFLSIYSGYDQQSIRTFKMRGRKKDCLSCSGKITKETIQSEINYSEFCGVINYDVLPEDLRITVDEYNRVVMMDQDHILLDVRPREHYAVVNLPHSINIPWDRIQRLKHINELGFDTAKPVYTICRFGNDSQYASKFLNDTFGLNTKDIKGGLSQWSAKIDPDLPTY

>tr|A0A0C7MTJ3|A0A0C7MTJ3_9SACH/1427 Adenylyltransferase and sulfurtransferase UBA4 OS=Lachancea lanzarotensis OX=1245769 GN=UBA4 PE=3 SV=1MDTEIAALKAENESLKQQLREQGQLPMSLEEFKRYGRQMIVQETGGVEGQIKLKNSRVLVIGAGGLGCPCLPYLAGAGVGQIGIVDNDVVETSNLHRQVLHNSSKVGVLKCESAKDVLVKLNPHIKVDTFPVRLDYSNACRIFQGYDVVLDCTDTPLTRYLVSDVAVNLGLTVVSASGLGFEGQLSILNFKNVGPCYRCFFPVPPPPNSVSSCQEGGVIGPCIGLVGVMMAVETLKIILDSYTLDNFKPFLMQYSGFPAQSLRTFKMRGRQATCLACGENPSITRDSIERGHIDYELFCGKRDYNVCLPHERMTVDEFQKSFLKTAELNHILLDVRPRHHYDISHLPNTFNLTVKELRDMDGDMSQLKREIPTVGAQSEVLVLCRHGNDSQLATRILKDQFHLKNVRDVKGGLFKYIDEINPAIPKY

>tr|A0A0P1KZA0|A0A0P1KZA0_9SACH/1436 Adenylyltransferase and sulfurtransferase UBA4 OS=Lachancea quebecensis OX=1654605 GN=UBA4 PE=3 SV=1MGIDSIPEDVLLELEALRRENTALKQKLSSQSELPMSLEEFRRYGRQMIVEETGGVEGQLKLRNAKVLVIGAGGLGCPCLPYLVGAGVGQVGIVDNDVIDTSNLHRQVLHDSTKVGMLKCESAKEVLGKLNPYVQINTYPVRLSYSNAFSIFQNYDIVLDCTDTPLTRYLVSDVAVCLGMTVVSASGLGSEGQLSILNFASAGPCYRCFYPVPPPPSAVSSCQEGGVIGPCIGLVGVMMAVETLKILLNIYTLDNFKPFLIQYSGLPNQTLRTFKMRGRQPSCKACGEERIITRDTIEAGEVNYEAFCGARNYNVCSPEERINVQEFENSVSLDGKPRQILLDVRPHHHYKISHLPNTFNLTVKELRDMEGDMSLLQNKIPDIHNESEVLVMCRYGNDSQLATRLLKDKFNIMKVKDVRGGFFKYIDDINPSLPKY

>tr|A0A1B2JDX5|A0A1B2JDX5_PICPA/1429 Adenylyltransferase and sulfurtransferase UBA4 OS=Komagataella pastoris OX=4922 GN=UBA4 PE=3 SV=1MSVEELTRELERLKEENRTLKNEIADRKVKATEKEPLNGYPLSLDEYSRYGRQLIVPEFQGVQGQVNLKNSKVLVIGAGGLGCPALLYLCAAGVGKIGIVDNDYVDKSNLHRQVLHTTNRVGMLKCESARVYLESLNPNTEVETFPVRLHNDNVFEIMSGYEYVLDCTDTPQTRYLINDGAVLCGLTIVSGSGLKTEGQLSILNFQSQGPCYRCFYPNPPPPNSVTSCKDGGVIGPAIGLVGIMMAYEAIKLITGYYTQENFSPFLTIYSSYPKQSLRTFKMRGRSGKCAACNGTITRKAIESGEIDYQAFCGTVHYKVLDPKNDRISVTKYDELMTTDHSLIDVRPKEQFQICSLPNSINIPIEQLVKKELKDLSQFQSGTPNFFICRYGNDSQSAVEFFRTLGIPSKDIIGGLNCWSNEIDSSVPKY

>tr|A0A1E3P435|A0A1E3P435_WICAA/1428 Adenylyltransferase and sulfurtransferase UBA4 OS=Wickerhamomyces anomalus (strain ATCC 58044 / CBS 1984 / NCYC 433 / NRRL Y3668) OX=683960 GN=UBA4 PE=3 SV=1MTQVDLAEELKRLRLENEQLKQKLCVKEAEEQEKSDSSVTDFSLDEYRRYGRQMIVPQVGFEGQKKLKKAKILVVGAGGLGCPALLYLAGAGVGKIGIVDDDTVDTSNLHRQVLHTTDKVGEYKCESAKSYLNKLNPHVEIQTYPFRLTTCNAFEIFEQYDLVLDCTDSPYSRYLISDVAVITRIPVVSGSGLKTEGQLSILNFENKGPCYRCFYPKPPPPNSVTSCKDGGVIGPVIGLMGVMMALETIKVIVGSYNAENFKPFLSIYSGYDQQSIKTFKMRGRKPDCLACSGHLTREVIARGDINYAEFCGVVNYNVLKPEERITIGEYAKVVDSDHVLIDVRPKEHFEIVTLPNSINIPLDRLRRIQNIEDLGFDSTKTIYTICRYGNDSQYASRFLNDTFDLKTKDVSGGLFQWSNKIDPKFPTY

>tr|A0A1E3QVA1|A0A1E3QVA1_9ASCO/1433 Adenylyltransferase and sulfurtransferase UBA4 OS=Babjeviella inositovora NRRL Y12698 OX=984486 GN=UBA4 PE=3 SV=1MVDTISMVDTVSYTEQIKALKRENEQLKQQLNARVAKDETQVDAFFTLEEYLRYGRQMTVRELGISGQRKLKTARVLVIGAGGLGCPALLYLAGAGVGKLGIVDNDVVDTSNLHRQVLHSTERVGQLKCESAKTYLQKLNPFVEIETYPVRLLNSNSFAIFRNYEVILDCTDTPITRYLISDTATLCGLPVVSGSGLRTEGQLSILNFNNTGPCYRCFYPRPPPPNAVTSCSDGGVLGPAIGLVGVMMALETLKLVSGAYTQDNFKPFLTVYAGYPTQSLRTFNMRGRKADCKACSLQASITEKMITSGEINYTEFCGRVNYNVLQPEERVSVEELAEALKSEVTLLDVRAPEQFEICSLPKATHISLDALEKMSVEELVQLKEPIYVVCRFGNDSQLATRLLKERAGLKRVWDVKGGVSRWADVVDLKFPKY

>tr|A0A1E4SNC0|A0A1E4SNC0_9ASCO/1440 Adenylyltransferase and sulfurtransferase UBA4 OS=Suhomyces tanzawaensis NRRL Y17324 OX=984487 GN=UBA4 PE=3 SV=1MDPSNEQLLARIKELEQENEQLKAQATLQSKSTSSFPKIDDNFSLDEYKRYGRQMIVPDFGSLPSQIKLKNSSILVVGAGGLGCPALLYLSAAGIGKIGIIDDDLVDTSNLHRQVLHTTETVGIHKCESARRYINKLNPHVVVETYQFRLQNDNAFDIVSKYDLVLDCTDTPATRYLINDVSVLCGKTIVSGSGLKTEGQLSILNFNNKGPCYRCFYPKPPSPESVTSCSDGGVIGPVIGLLGITMAVETIKILTGYYTDETFKPFLSMYSAYPQQQLRVFKMRNRKPDCAVCGNLPQILKETILENQIDYAAFCGTVNSNVLNPDERIDVKKYSELLNKNNSQVLLDVRPKEQFGITKLANSINIQWDPIFKKAESIDEYLPQNFDKSKDSVYVVCRYGNDSQLATRKMIDELGFKNVKDLIGGLNKWSEVVDSTVPQY

>tr|A0A1E4TPV2|A0A1E4TPV2_PACTA/1447 Adenylyltransferase and sulfurtransferase UBA4 OS=Pachysolen tannophilus NRRL Y2460 OX=669874 GN=UBA4 PE=3 SV=1MTLDFSKEELLSQIESLKKENEALRHAKIYSETSAAGVAVVTAANRESSFSLEEYRRYGRQMTVPEVGISGQVMLKKSKILVVGAGGLGCPALLYLTGAGVGKIGIVDNDVVDTSNLHRQVLHTSENVGMLKCDSAKKYLNSLNPHVEIVTFPKRLSNDNIFNVFLSDKWDLILDCTDAPLTRYLINDAAVICGLTIVSASGVRADGQLAILNFQNFGPCYRCFYPKPPPPNSVLSCQDGGVIGPAIGVMGVLMASEAIKVICGSYTRENFKPFLNLYSAYPQQSLRTFKMRSKVPNCQVCSIDNDKRIITRSTIESNELNYSEWCGKVSYDVLKMEDRVNVSEFSQKIANDKDKDNTMILDVRPKAHFDICHLPNSINIPYKELSQLKEFSVPSNKTIYCICRFGNDSRLAVKFLKYELGLPNTILDVIGGLNQWSEQVDTQFPKY

>tr|A0A1G4ISY8|A0A1G4ISY8_9SACH/1436 Adenylyltransferase and sulfurtransferase UBA4 OS=Lachancea dasiensis OX=1072105 GN=UBA4 PE=3 SV=1MTDQPVPSDVLNELAALRAENATLKQQLKSETQLPMSLEEFKRYGRQMIVEETRGVEGQLKLRRSKVLVVGAGGLGCPCLPYLAGAGVGLIGIVDNDVVETSNLHRQVLHDSTKVGMLKCESAKEVLSRLNPFVVIKTYPVRLNYSNAFDIFKDYDIVLDCTDTPLTRYLISDVAVNLGLTVVSASGLGSEGQLSILNFKNVGPCYRCFYPVPPPPNAVSSCQEGGVIGPCIGLVGVMMAVETIKLILDIYTLDNFSPFLMQYSGFSLQSLRTFKMRGRQVKCQACGENPTITRDTIEKGDVDYELFCGQRNYNVCLADERISVKEFQNSFLSPGEFNHILLDVRPHHHYEISHLPNTYNLTVKELRDMDGSIAALQSQIPNIHGQSEVLVMCRHGNDSQLATRILKDQFKIANVRDVRGGFFKYIDDINPTLPKY

>tr|A0A1G4J616|A0A1G4J616_9SACH/1431 Adenylyltransferase and sulfurtransferase UBA4 OS=Lachancea sp. CBS 6924 OX=433476 GN=UBA4 PE=3 SV=1MANAQESEIAALKAENASLKQQLKEQSQLPMSLEEFKRYGRQMIVQETGGVEGQVKLKNSRVLVVGAGGLGCPCLPYLAGAGVGQIGIVDNDVVETSNLHRQVLHDSNKVGVLKCESAKDVLLKLNPHIKVDTFPVRLDYSNAFQIFQNYDIVLDCTDTPLTRYLVSDVAVNLGMTVVSASGLGFEGQLSILNFKSVGPCYRCFYPVPPPPNSVSSCQEGGVIGPCIGLVGVMMAVETLKIVLDVYTMDNFKPFLMQYSGFPTQSLRTFKMRGRQASCLACGKNPSITKASIQRGDIDYELFCGKRNYNVCLPHERMTVDEFHTSFSQASELNHILLDVRPRHHYNISHLPNTFNLTVKELRDMEGDISQLKREIPAIDTKSEVLVLCRYGNDSQLATRILKDQFHLSNVRDVKGGFFKYIDEINPNIPKY

>tr|A0A1G4J7B8|A0A1G4J7B8_9SACH/1436 Adenylyltransferase and sulfurtransferase UBA4 OS=Lachancea meyersii CBS 8951 OX=1266667 GN=UBA4 PE=3 SV=1MSSGDVPAEILDELAALKAENFSLKQKLNEQVQLPMSLEEFKRYGRQMIVKETGGVDGQMKLRRSKVLVIGAGGLGSPCLPYLAGAGVGQIGIVDNDVVETSNLHRQVLHDSEKVGMLKCESAKEVLSRLNPHVKIDTYPVRLDYSNAFRIFRSYDIVLDCTDTPLTRYLISDVAVNLGMTVVSASGLATEGQLAVLNFKNVGPCYRCFYPVPPPPNSVSSCQEGGVVGPCIGLVGVMMAVETLKIILDIYTLDNFQPFLMQYSGFPLQSLRTFKMRGRQKTCKACGESRIITKDAIERGHVNYEAFCGKRNYNVCLPHERISVEEFHDSYVASDEQNPILLDVRPHHHYNISHLPNTFNLTVKELRDMEGDISQLKSEIPTISGDSEVLVMCRYGNDSQQATRILKDNFNLSNVRDVQGGFFKYIDEVNPKLPKY

>tr|A0A1G4JQY8|A0A1G4JQY8_9SACH/1435 Adenylyltransferase and sulfurtransferase UBA4 OS=Lachancea mirantina OX=1230905 GN=UBA4 PE=3 SV=1MNLEDRKELEALRKENAELKRKLGVQKFQNDELAMSLEEFKRYGRQMIVETFGGVDGQLKLRNAKVLIIGAGGLGCPCLPYLAGAGIGHIGIVDNDVVDTSNLHRQVLHSSNSVGKLKCDSAKEFLQKLNPHVEVTTYPVRLSHANCFGIFTGYDYVLDCTDTPLTRYLISDVAVCLGMTVVSASGLGYEGQLSILNFKSMGPCYRCFYPAPPSPSNVSSCQEGGVIGPCIGLVGIMMAVETIKLIMGVYTEENFEPFLLQYSGFPHQTLRRFKMRGRKPNCISCGPDSTVNRAAILAGGINYELFCGKRNYDVCKPEERITVEEFEKAYMSQEGLDYTLLDVRPHHHYDISHLPNTFNITVKELRDMDGNMDLLKRHIPKLTDRSEVLVMCRYGNDSQLATKLLKDEFKISTVRDIRGGLFKYIDEINPSLPKY

>tr|A0A1G4KNM0|A0A1G4KNM0_9SACH/1436 Adenylyltransferase and sulfurtransferase UBA4 OS=Lachancea nothofagi CBS 11611 OX=1266666 GN=UBA4 PE=3 SV=1MPSDLGSSKAFDELEALRAENAALKQQLSAQNQLPMSLEEFKRYGRQMIVEDTGGVAGQLKLRGSKVLVIGAGGLGCPCLPYLAGAGVGQIGIVDNDVVETSNLHRQVLHDSSKVGMLKCESAKEVLLKLNPHVTIDTYPVRLNYSNAFDIFQHYDIVLDCTDTPLTRYLISDVAVNLRMTVVSASGLGSEGQLSILNFKDVGPCYRCFYPVPPPPNAVSSCQEGGVIGPCIGLVGVMMAIETIKIILDIYTLDNFQPFLMQYSGFPVQTLRTFKMRGRQTSCKACGDNRTISRKAIEAGDVDYELFCGKRKYDVCSSKERISVKELETSYLSPGELNHILLDVRPHHHYNISHLPNTYNLTVKELRDMEGDITRLQSCIPTVHRQSEVLVICRHGNDSQLATRLLKDQFNITNVRDVRGGFFKYIDDMNPSLPKY

>tr|A0A1G4MEK4|A0A1G4MEK4_LACFM/1447 Adenylyltransferase and sulfurtransferase UBA4 OS=Lachancea fermentati OX=4955 GN=UBA4 PE=3 SV=1MSSKTEHMESEDKLANALKELEALKLENAKLREQLSGEKALDAELPMSLEEFSRYGRQMIVEDTKGIQGQIKLRDAKVLVIGAGGLGCPCLPYLAGAGVGHIGIVDNDTVETSNLHRQILHNSSKVGMLKCESAKKVLQKLNPHVSITTYPVRLGYSNAFGIFEGYDIVLDCTDTPLTRYLISDVAVCLGMTVVSASGLGTEGQLSILNFQGIGPCYRCFYPTPPPPNAVSSCQEGGVIGPCIGLVGVMMAVETLKIIMEIYTSDNFQPFLMQYAGFPQQTLRTFKMRGKKQACLCCGDQPTITRASITSGSINYELFCGKRNYNVCSPEERMTVEEFEADYWKNSGLDYILLDVRPHHHYEISHLPNTFNLTVKELRDMDGDFAKLRSKIPPVSKEKEVLVMCRHGNDSQLATRLLKDQFKIRNVRDIKGGFFKYIDNVNPSLPKY

>tr|A0A1Q3A3D6|A0A1Q3A3D6_ZYGRO/1436 Adenylyltransferase and sulfurtransferase UBA4 OS=Zygosaccharomyces rouxii OX=4956 GN=UBA4 PE=3 SV=1MTVEVPESILRELESLKLENAKLKAELAGEKEFNKYPMSLEEYKRYGRQMIVEDTGGVEGQLKLCNSKVLVVGAGGLGCPALPYLAGAGIGHIGIVDNDVVDTSNLHRQVLHNSNKVGMLKCESAKQVLNELNPHVSVETYPIRLDGANAFPIFAKYDYILDCTDTPLTRYLISDVAVNLQKTVVSASGLGTEGQLTILNFKNKGPCYRCFYPVPPSPTSVSSCQEGGVIGPCIGLVGVMMAIEVMKLIMEVYTEESFKPFLTSYSGFPTQSLRTFKMRGRQKTCACCGENPTITRNAIESGEVDYTLFCGSRKYNVCDSMERMSVTDFENDYHDRQDFVFLDVRPSHHYSISHFPSTHNIPLKKLRDMGGSISELQEHVPNIDKESEVVVVCRYGNDSQLATRLLKDQFGIPKVKDIRGGFFKYIDDVDPSIPKY

>tr|A0A1Q3AH75|A0A1Q3AH75_ZYGRO/1436 Adenylyltransferase and sulfurtransferase UBA4 OS=Zygosaccharomyces rouxii OX=4956 GN=UBA4 PE=3 SV=1MTVGVPENILRELEALKLENARLKAELAEGKELNKYPMSLEEYKRYGRQMIVEDTGGVKGQLKLRNSKVLVVGAGGLGCPALPYLAGAGIGHIGIIDNDIVDTSNLHRQVLHSSDRVGMLKCESAKQVLNKLNPHVNVRTYPIRLDGTNAFPIFAKYDYILDCTDTPLTRYLISDVAVNLKKTVVSASGLGTEGQMTILNFKNKGPCYRCFYPVPPSPNSVSSCQEGGVIGPCIGLVGVMMAIEVMKLIMGVYTEENFKPFLASYSGFPTQSLRTFKMRGRQKTCACCGENPTITREAIVSGAVDYTSFCGSRNYNVCDLTERISVNDFENYYHDRQDFVFLDVRPSHHYSISHFPSSHNIPLKRLKDMGGSISALQKHVPHIDRDSEVVVVCRYGNDSQLATRLLKDQFGIPKVKDIRGGFFKYIDDVDPSIPKY

>tr|A0A1V2L4M1|A0A1V2L4M1_CYBFA/1429 Adenylyltransferase and sulfurtransferase UBA4 OS=Cyberlindnera fabianii OX=36022 GN=UBA4 PE=3 SV=1MSQEDLLEELTRLRAENASLRAQVSSQHHTEPTNTSKLVNGLTHDEYKRYGRQMIVPQVGFTGQLSLKHSKVLVVGAGGLGCPALMYLAGCGIGKIGIVDGDTVDVSNLHRQVMHDSTTVGMMKVESARLFINRLNPGVEVRTYAERLSTDNAFDIFEEWDLVLDCTDSPYSRYLISDVAVVLGKTVVSGSGLKTEGQLSILNFENVGPCYRCFYPTPPPPNSVTSCTDGGVIGPVIGLMGVMMALEAIKIITGTYTVENFKPFLSIYSGYDQQSIRTFKMRGRKKDCLSCSGKITKETIQSEINYSEFCGVINYDVLPEDLRITVDEYNRVVMMDQDHILLDVRPREHYAVVNLPHSINIPWDRIQRLKHIDELGFDTAKPVYTICRFGNDSQYASKFLNDTLGLNTKDIKGGLSQWSAKIDPDLPTY

>tr|A0A1X7R273|A0A1X7R273_9SACH/1450 Adenylyltransferase and sulfurtransferase UBA4 OS=Kazachstania saulgeensis OX=1789683 GN=UBA4 PE=3 SV=1MDGEQELLKQIEQLKLENSQLRNQLENGGSLKKKTFDDDMNTIGDTEYPLSLEEYHRYGRQMIVEETEGVKGQIKLKNAKILVIGAGGLGCPSLPYLAGAGIGTIGIVDNDTIDTSNLHRQILHSTANVGMLKCESAKRYLEQLNPNVNVITYPVRLNNHNAFEIFKDYDLVLDCTDTPITRYLISDVAVNLGMTIVSASGVRTDGQLTILNFKNVGPCYRCFYPVPPSPNSVSSCSEGGVIGPCIGLVGVMMAVETIKLLLDVYTLDNWEPFLMQYSGFPKQSLRTFRMRGRKDSCKCCGESPTITKQSIESNEINYQEFCGVRNYDVCSTNERITVNEFEESYHKIDKKDYILLDVRPPHHYDISHLANTYNIPVKSLRDMDGSLEQLQKIIPDVNEKSEVVVMCRFGNDSRLATRLLKDEFKIKNVKDIAGGFFKYIDDIDQKIPKY

>tr|A0A2P7YUY4|A0A2P7YUY4_9ASCO/1436 Adenylyltransferase and sulfurtransferase UBA4 OS=[Candida] pseudohaemulonii OX=418784 GN=UBA4 PE=3 SV=1MSDLQLRIKQLEEENRRLVGELQREKQRNIEYEPSNDPDFSLEEFRRYGRQMIVPEFGSLPAQRKLKNAKVLVVGAGGLGCPTLLYLTAAGVGKIGIVDNDTVDVSNLHRQVLHTSDKVGMLKCDSAKEYLNRLNPHVDIMTHPIRLDNSNAFNIFGEYDLIVDCTDAPAIRYLINDVAVLSGKTIVSGSGVKTDGQFTILNFNNTGPCYRCFYPQPPKPESVSTCGDAGVFGPAIGLTGISLATETLKVLTGHYDETFKPFLAMYTAYPLLVLRQFKMRGRQAACKVCGTQPTITKQSIESGEINYDQFCGSVNSNVLTPEQRIDAKEAENYLKADDRSVLLDVRPFEQYQIANISRSINIDWALRLSRLESDESIFPETFDKKNDQVYVICRYGNDSQLATKKLTEDFGVAHVRDIKGGINRWSETVDQRLPKY

>tr|A0A376B7B3|A0A376B7B3_9ASCO/1442 Adenylyltransferase and sulfurtransferase UBA4 OS=Saccharomycodes ludwigii OX=36035 GN=UBA4 PE=3 SV=1MAVNSDIDELKKEIEKLKLENANLRKNATLSANTHLPMSIAEYSRYGRQMICEESNGIEGQLKLKNSKVLFIGAGGLACPALAYLAGAGIGHIGIVDDDVVENSNLHRQILHDSTKVGMLKCESAKEYITKLNPHINVTTHPVRLDNYNAFNIFKQYDIILDCTDTPITRYLISDVSVNCGKVVVSASGVGTEGQLCILNFADVGPCYRCFYPTPPPPNTVSSCSDGGVIGPCIGLVGVMMAVETMKLILGVYTVENFKPFLLQYCGFPNQTLRSFKMRYRQATCACCGDAAHRKISQENIESGEINYGIFCGSKNYDVCQPSERLNIQDFYNDYYRKDQKANGHLLLDVRPRHHYQISHLPNSYNVTVKELKQMNGDLTLLKREIPEVNKDSEIIVICRRGNDSRLATRLLKDQFEIPNVKDVIGGFFRYIDEIDPQIPKY

>tr|A0A448YIG7|A0A448YIG7_BRENA/1434 Adenylyltransferase and sulfurtransferase UBA4 OS=Brettanomyces naardenensis OX=13370 GN=UBA4 PE=3 SV=1MSDTEHLQAEIRRLKLENTSLRERCSGCSSTGNQSAAQSSPHHLSLQEYRRYGRQMQVPEFGGLTSQERLKSSSALIIGAGGLGSPALLYLAAAGIGRIGIVDHDVVDTTNLHRQIIHDTDTVNINKAESAKIRINALNPNVEVTTYCEPITNTNSFKIVKDYDIVLDCSDNPATRYLINDTCVKLDKTFVSGSGLRTEGQLTVLNFHRWGPCYRCFFPTPPPPNTVTSCSDGGVIGPCIGITGVMMAVEAIKVLTDYYTDDNFKPFLTVYNGYPQQKLRTFKMRGRVKGCKACDSDEITKEDIESGEVDYEGWCGSMNYNVLDSAERINVRDFSNEWENDKNINPLIDVRAKEQYEIAHLSGSINIPYSELSREVVLPECIDPKKKTYVICRFGRDSQLSTRLLKEKLSDVKDVIGGLDAWKKEVDEDFPAYW

>tr|A0A5P2U7A5|A0A5P2U7A5_KLULC/1444 Adenylyltransferase and sulfurtransferase UBA4 OS=Kluyveromyces lactis OX=28985 GN=UBA4 PE=3 SV=1MTEMEGSNESLLEEIEKLRVENAKLKNDLCLERSLNNASDLPMSLDEFKRYGRQMIVDETEGLVGQLKLKNASVLVIGAGGLGCPSLPYLAGAGIGKIGIVDNDTVDTSNLHRQVLHDTTKVGMLKSESAKQVLNKLNPHVSVTSYPVRLSNENAFDIFKDYDVILDCTDTPMARYLISDVAVNLGKPVVSASALRTEGQLSIFNFDNVGPCYRCFYPTPPAPTSVSSCQEGGVLGPCVGLVGVAMVVETLKLLLGVYTRENFKPFLLQYSGFPDQTLRKFKMRGTRVDCPACGTGRTVTRESIESGKINYQSFCGSRNYSVLSEDERIDVQKFERDYWSSSKTKSYVLLDVRPSLHYSISHLPNSHNITVNELRDLPADLNNLQSRIPHLSEDSEVLVLCRYGNDSQLATRLLKDKFNLKNVKDVKGGFFKYIDEINPSLPKY

>tr|A0A7D9CZA4|A0A7D9CZA4_DEKBR/1434 Adenylyltransferase and sulfurtransferase UBA4 OS=Dekkera bruxellensis OX=5007 GN=UBA4 PE=3 SV=1MTVNKEAELRREIEALKKENKELKAQKLNDNKKSVCALSLEEYRRYGRQMQVPEFGGLSSQLKLKNSKILVIGAGGLGSPALLYLAAAGVGKIGIVDNDVVDRTNLHRQIIHDTSTLNLNKAESARIKMRLLNSNIEVVTYPVALTNLNSFQIVEPYDLVLDCTDTPASRYLINDTCVLLGKTIISGSGLRTEGQLTILNFHNQGPCYRCFYPTPPPPNTVTSCSNGGVIGPCIGVLGTMMAVEALKCVVGFYTQENFHPFLTLYSGFAPGQKLRTFRMRSRMSKCKVCNPDVREITREKIEQGVIDYKGWCGAMNYNVLNPQTQRITAKQLSKVDFSDVEHLNPLLDVRPRIQYDISHIDGSINIPYDDLSRSKSVLEGLDPHKKTYVICRFGNDSQLSTKILLERNFEDVKDVIGGLDSWKKDVNADFPSYW

>tr|A0A7G3ZI40|A0A7G3ZI40_9SACH/1438 Adenylyltransferase and sulfurtransferase UBA4 OS=Torulaspora globosa OX=48254 GN=UBA4 PE=3 SV=1MEASEELRKELTALKLENARLHRELQNREREQEKRYPLSLEEFRRYGRQMIVEDTGGVSGQIKLRNAKVLVVGAGGLGCPVLPYLAGAGVGVIGIVDNDVVDTSNLHRQVLHNSTTVGMLKCESARRVLNKLNPFVEVRTHPVRLSNANAFEIFEFYDIILDATDTPLTRYLISDVAVNLGKTVVSASGLGTEGQLCILNYNNEGPCYRCFYPKPPTPTSVTSCQEGGVIGPCIGLVGTMMAVETLKLILGEYEKIGFQPFLKTYSGFPEQVLRTFRMRGKQSNCACCGQTPTVTRESIESGLIDYDAFCGSRNYDVCTSEERITVEKFEREYRTRSDSNYILLDVRPRHHYKISHLSDSHNVTVKELRDMDGELEKLQAHIPNISKDSEVVVMCRYGNDSRLATRLLIDRFGIPSVKDISGGLFKYIDKVDPSIPKY

>tr|A0A7H9HU17|A0A7H9HU17_9SACH/1436 Adenylyltransferase and sulfurtransferase UBA4 OS=Torulaspora sp. CBS 2947 OX=2792677 GN=UBA4 PE=3 SV=1MEVSEELRKELIALKLENARLHRELEKREQEKRYPLSLEEFRRYGRQMIVEDTGGVSGQIKLRNAKVLVVGAGGLGCPVLSYLAGAGVGVIGIVDNDVVDTSNLHRQVLHNSTTVGMLKCESARQVLNKLNPFVEVKTYPVRLNNANAFEIFEFYDIILDATDTPLTRYLISDVAVNLGKTVVSASGLGTEGQLCILNYNNEGPCYRCFYPKPPTPTSVTSCQEGGVIGPCIGLVGTMMAVETLKLILGEYEKNGFQPFLKTYSGFPEQVIRTFRMRGKQSNCACCGQTPTVTRESIESGLINYDAFCGSRNYDVCTSEERITVELFEKEYRTREDSNYILLDVRPHHHYNISHLSDSHNVTVKELRDMDGELEKLRAHIPNITKDSEVVVMCRYGNDSRLATRLLIDRFGIPSVKDVSGGLFKYIDKVDPSIPKY

>tr|A0A815Z5S7|A0A815Z5S7_YEASX/1440 Adenylyltransferase and sulfurtransferase UBA4 OS=Saccharomyces cerevisiae PE2 OX=1220494 GN=UBA4 PE=3 SV=1MNDYHLEDTTSELEALRLENAQLREQLAKREDSSRDYPLSLEEYQRYGRQMIVEETGGVAGQVKLKNTKVLVVGAGGLGCPALPYLAGAGVGQIGIVDNDVVETSNLHRQVLHDSSRVGMLKCESARQYITKLNPHINVVTYPVRLNSSNAFDIFKGYNYILDCTDSPLTRYLVSDVAVNLGITVVSASGLGTEGQLTILNFNNIGPCYRCFYPTPPPPNAVTSCQEGGVIGPCIGLVGTMMAVETLKLILGIYTNENFSPFLMLYSGFPQQSLRTFKMRGRQEKCLCCGKNRTITKEAIEKGEINYELFCGARNYNVCEPDERISVDAFQRIYKDDEFLAKHIFLDVRPSHHYEISHFPEAVNIPIKNLRDMNGDLKKLQEKLPSVEKDSNIVILCRYGNDSQLATRLLKDKFGFSNVRDVRGGYFKYIDDIDQTIPKY

>tr|A0A871R5J5|A0A871R5J5_DEKBR/1434 Adenylyltransferase and sulfurtransferase OS=Dekkera bruxellensis OX=5007 GN=UBA4 PE=3 SV=1MTVNKEAELRREIEALKKENKELKAQKLNDNKKSVCALSLEEYRRYGRQMQVPEFGGLSSQLKLKNSKILVIGAGGLGSPALLYLAAAGVGKIGIVDNDVVDRTNLHRQIIHDTSTLNLNKAESARIKMRLLNSNIEVVTYPVALTNLNSFQIVEPYDLVLDCTDTPASRYLINDTCVLLGKTIISGSGLRTEGQLTILNFHKQGPCYRCFYPTPPPPNTVTSCSNGGVIGPCIGVLGTMMAVEALKCVVGFYTQENFHPFLTLYSGFAPGQKLRTFRMRSRMPKCKVCNPDVREITREKIEQGVIDYKGWCGAMNYNVLNPQTQRITAKQLSKVDFSDVEHLNPLLDVRPRIQYDISHIDGSINIPYDDLSRSKSVLEGLDPHKKTYVICRFGNDSQLSTKILLERNFEDVKDVIGGLDLWKKDVNADFPSYW

>tr|A0A875S0C6|A0A875S0C6_EENNA/1427 Adenylyltransferase and sulfurtransferase OS=Eeniella nana OX=13502 GN=UBA4 PE=3 SV=1MPSKQELLEEVARLRAENDLLKKKCNKISTLPLNREEYRRYGRQMQVPEFGGLNAQVKLKNSKLLVIGAGGLGCPALLYLAAAGIGQIGIVDHDTVDITNLHRQVIHDTNTVGMFKAESAQIKIQALNPNVKVVTYCEALTNTNCFDIVESYDIVLDCTDNPASRYLINDTCVLLGKTIVSGSGLKTEGQLSILNFHNKGPCYRCFYPKPPPPNSVTSCSDGGVIGPCIGLLGVMMAIEALKCLTEFYTDENFRPFLALYSGYSPQQKLRTFKMRGRSPTCEVCKADCRKVTRKLIESGKIDYENWCGSLNYEVLDSNTERISAKQLAQIDFGVPTINPLIDVRPEEQYDIAHLDHSINIPYAQLSQTDFLPESIDPTKPTYVICRFGRDSQLSTKALQNRLANVKDVIGGLDAWQREVDSDFPVYW

>tr|A0A8B8USV2|A0A8B8USV2_SACPA/1440 Adenylyltransferase and sulfurtransferase OS=Saccharomyces paradoxus OX=27291 GN=UBA4 PE=3 SV=1MSDHELDGTTSELEALRLENARLREQLAKRKDNSQNYPLSLEEYQRYGRQMIVEETGGVAGQVKLKNSKVLVVGAGGLGCPALPYLAGAGVGQIGIVDNDVVETSNLHRQVLHDSSRVGMLKCESARQYITKLNPHINVVTYPVRLNSSNAFEIFRYYDYVLDCTDSPLTRYLVSDVAVNLGITVVSASGLGTEGQLTILNFNNMGPCYRCFYPTPPPPNAVTSCQEGGVIGPCIGLVGTMMAVETLKLILGIYTEENFSPFLMLYSGFPQQSLRTFKMRGRQEKCLCCGKNRTITKEAIEKGEINYELFCGSRNYSVCESDERISVEAFQNIYKDNGFLPRHIFLDVRPSHHYEISHFPEAVNIPIKKLRDMGGDLKKLQEELPTVERDSDIVVLCRYGNDSQLATRLLKDKFGLSNVRDVRGGYFKYIDDIDQTIPKY

>tr|A0A8H4BZU9|A0A8H4BZU9_YEASX/1440 Adenylyltransferase and sulfurtransferase UBA4 OS=Saccharomyces cerevisiae OX=4932 GN=UBA4 PE=3 SV=1MNDYHLEDTTSELEALRLENAQLREQLAKREDSSRDYPLSLEEYQRYGRQMIVEETGGVAGQVKLKNTKVLVVGAGGLGCPALPYLAGAGVGQIGIVDNDVVETSNLHRQVLHDSSRVGMLKCESARQYITKLNPHINVVTYPVRLNSSNAFDIFKGYNYILDCTDSPLTRYLVSDVAVNLGITVVSASGLGTEGQLTILNFNNIGPCYRCFYPTPPPPNAVTSCQEGGVIGPCIGLVGTMMAVETLKLILGIYTNENFSPFLMLYSGFPQQSLRTFKMRGRQEKCLCCGKNRTITKEAIEKGEINYELFCGARNYNVCEPDERISVDAFQRIYKDDEFLAKHIFLDVRPSHHYEISHFPEAVNIPIKNLRDMNGDLKKLQEKLPSVEKDSNIVILCRYGNDSQLATRLLKDKFGFSNVRDVRGGYFKYIDDIDQTIPKY

>tr|A0A8H6EQY1|A0A8H6EQY1_DEKBR/1434 Adenylyltransferase and sulfurtransferase OS=Dekkera bruxellensis OX=5007 GN=UBA4 PE=3 SV=1MTVNKEAELRREIEALKKENKELKAQKLNDNKKSVCALSLEEYRRYGRQMQVPEFGGLSSQLKLKNSKILVIGAGGLGSPALLYLAAAGVGKIGIVDNDVVDRTNLHRQIIHDTSTLNLNKAESARIKMRLLNSNIEVVTYPVALTNLNSFQIVEPYDLVLDCTDTPASRYLINDTCVLLGKTIISGSGLRTEGQLTILNFHNQGPCYRCFYPTPPPPNTVTSCSNGGVIGPCIGVLGTMMAVEALKCVVGFYTQENFHPFLTLYSGFAPGQKLRTFRMRSRMPKCKVCNPDVREITREKIEQGVIDYKGWCGAMNYNVLNPQTQRITAKQLSKVDFSDVEHLNPLLDVRPRIQYDISHIDGSINIPYDDLSRSKSVLEGLDPHKKTYVICRFGNDSQLSTKILLERNFEDVKDVIGGLDSWKKDVNADFPSYW

>tr|B9W999|B9W999_CANDC/1439 Adenylyltransferase and sulfurtransferase UBA4 OS=Candida dubliniensis (strain CD36 / ATCC MYA646 / CBS 7987 / NCPF 3949 / NRRL Y17841) OX=573826 GN=UBA4 PE=3 SV=1MSEPSKEELLAKIAQLEFENNQLKQQQHGLKSKNEQFCKIDENFSLDEYKRYGRQMIVPQFGSLESQIKLKNSKVLVVGAGGLGSPALLYLSSAGIGTIGIIDHDTVDTSNLHRQVIHNTEMVGEFKCISAQNYIKKLNPHVIVDVYPTTLSNDNAFDIISQYDLVLDCTDHPAVRYLINDVCVLLGKTIVSGSGLKSDGQLTVLNFANSGPCYRCFYPQPPSPASVTSCSDGGVIGPAIGLVGVAMAVETIKIITGYYTKDNFVPFLTSYSGYPQQQLRAFKMRKRQKDCAVCGENPQISRRIIEDGTINYKTFCGRVTFDPIDDKFRISPKDYDRVVQNKQRHILLDVRPREQFQITHLPNAINVQWDPVFRKADTIQQYLPEDSTKDNEIYVVCRFGNDSQLAAKKLLDLGYSNVRDIIGGLDKWSDDVDSKIPKY

>tr|C4YCV7|C4YCV7_CANAW/1438 Adenylyltransferase and sulfurtransferase UBA4 OS=Candida albicans (strain WO1) OX=294748 GN=UBA4 PE=3 SV=1MSEPSKEELLARIAQLELENEQLKQQNGKKSQHEQFNKIDDNFSLDEYKRYGRQMIVPQFGSLESQIKLKNSKVLVVGAGGLGSPALLYLSSAGIGKIGIIDPDTVDTSNLHRQVIHNTEMVGEFKCISAQNYINKLNPHVVVEVYPTALNNDNAFGIVSQYDLVLDCTDHPAVRYLINDVCVLLGKTIVSGSGLKSDGQLTVLNFANSGPCYRCFYPQPPSPDSVTSCSDGGVIGPAIGLVGVAMAVETIKIITGYYTKDNFVPFLASYSAYPQQQLRVFKMRKRQKDCAVCGENPQISQRMIEDGTINYKTFCGRATFDPIDDKFRVSPKDYDSVVQNKKKHILIDVRPREQFQITHLPNAINVQWDPTFRKADAIEQYLPDDSTKDDEIYVVCRFGNDSQLAAKKLIGMGYPNVRDIIGGLDKWSDDVDSKIPKY

>tr|C5DJG9|C5DJG9_LACTC/1436 Adenylyltransferase and sulfurtransferase UBA4 OS=Lachancea thermotolerans (strain ATCC 56472 / CBS 6340 / NRRL Y8284) OX=559295 GN=UBA4 PE=3 SV=1MTTDNIPNEVLLELEALRRENEALKQKLSSQTELPMSLEEFRRYGRQMIVEDTRGVEGQIKLRNAKVLVIGAGGLGCPCLPYLVGAGVGQVGVVDNDVIDTSNLHRQVLHDSTKVGMLKCESAKDVLRKLNPHVQIKTYPVRLNYSNAFSIFQGYDIVLDCTDTPLTRYLVSDVAVCLGMTVVSASGLGSEGQLSILNFASVGPCYRCFYPVPPPPNAVSSCQEGGVIGPCIGLVGVMMAVETLKIILDIYTLDNFKPFLIQYSGLPNQTLRSFKMRGRQPSCKACGEEKLITRDTIEAGEVNYEAFCGARNYNVCSPEERINVQEFEDSISSNEKLSQILLDVRPHHHYKISHLPNTFNLTVKELRDMEGDMSLLQNEIPEIHNGSEVLVMCRYGNDSQLATRILKDKFNIMKVKDIRGGFFKYIDDINPSLPKY

>tr|C5DY26|C5DY26_ZYGRC/1436 Adenylyltransferase and sulfurtransferase UBA4 OS=Zygosaccharomyces rouxii (strain ATCC 2623 / CBS 732 / NBRC 1130 / NCYC 568 / NRRL Y229) OX=559307 GN=UBA4 PE=3 SV=1MTVEVPESILRELESLKLENAKLKAELAGEKEFNKYPMSLEEYKRYGRQMIVEDTGGVEGQLKLRNSKVLVVGAGGLGCPALPYLAGAGIGHIGIVDNDVVDTSNLHRQVLHNSNKVGMLKCESAKQVLNELNPHVSVETYPIRLDGANAFPIFAKYDYILDCTDTPLTRYLISDVAVNLQKTVVSASGLGTEGQLTILNFKNKGPCYRCFYPVPPSPTSVSSCQEGGVIGPCIGLVGVMMAIEVMKLIMEVYTEESFKPFLTSYSGFPTQSLRTFKMRGRQKTCACCGENPTITRNAIESGEVDYTLFCGSRKYNVCDSMERMSVTDFENDYHDRQDFVFLDVRPSHHYSISHFPSTHNIPLKKLRDMGGSISELQEHVPNIDKESEVVVVCRYGNDSQLATRLLKDQFGIPKVKDIRGGFFKYIDDVDPSIPKY

>tr|C7GLZ7|C7GLZ7_YEAS2/1440 Adenylyltransferase and sulfurtransferase UBA4 OS=Saccharomyces cerevisiae (strain JAY291) OX=574961 GN=UBA4 PE=3 SV=1MNDYHLEDTTSELEALRLENAQLREQLAKREDSSRDYPLSLEEYQRYGRQMIVEETGGVAGQVKLKNTKVLVVGAGGLGCPALPYLAGAGVGQIGIVDNDVVETSNLHRQVLHDSSRVGMLKCESARQYITKLNPHINVVTYPVRLNSSNAFDIFKGYNYILDCTDSPLTRYLVSDVAVNLGITVVSASGLGTEGQLTILNFNNIGPCYRCFYPTPPPPNAVTSCQEGGVIGPCIGLVGTMMAVETLKLILGIYTNENFSPFLMLYSGFPQQSLRTFKMRGRQEKCLCCGKNRTITKEAIEKGEINYELFCGARNYNVCEPDERISVDAFQRIYKDDEFLAKHIFLDVRPSHHYEISHFPEAVNIPIKNLRDMNGDLKKLQEKLPSVEKDSNIVILCRYGNDSQLATRLLKDKFGFSNVRDVRGGYFKYIDDIDQTIPKY

>tr|C8Z9Q4|C8Z9Q4_YEAS8/1440 Adenylyltransferase and sulfurtransferase UBA4 OS=Saccharomyces cerevisiae (strain Lalvin EC1118 / Prise de mousse) OX=643680 GN=UBA4 PE=3 SV=1MNDYHLEDTTSELEALRLENAQLREQLAKREDSSRDYPLSLEEYQRYGRQMIVEETGGVAGQVKLKNTKVLVVGAGGLGCPALPYLAGAGVGQIGIVDNDVVETSNLHRQVLHDSSRVGMLKCESARQYITKLNPHINVVTYPVRLNSSNAFDIFKGYNYILDCTDSPLTRYLVSDVAVNLGITVVSASGLGTEGQLTILNFNNIGPCYRCFYPTPPPPNAVTSCQEGGVIGPCIGLVGTMMAVETLKLILGIYTNENFSPFLMLYSGFPQQSLRTFKMRGRQEKCLCCGKNRTITKEAIEKGEINYELFCGARNYNVCEPDERISVDAFQRIYKDDEFLAKHIFLDVRPSHHYEISHFPEAVNIPIKNLRDMNGDLKKLQEKLPSVEKDSNIVILCRYGNDSQLATRLLKDKFGFSNVRDVRGGYFKYIDDIDQTIPKY

>tr|F2QSN3|F2QSN3_KOMPC/1429 Adenylyltransferase and sulfurtransferase UBA4 OS=Komagataella phaffii (strain ATCC 76273 / CBS 7435 / CECT 11047 / NRRL Y11430 / Wegner 211) OX=981350 GN=UBA4 PE=3 SV=1MSVEELTRELERVKEENRSLRNKLADKKAEAIEMEPSNGYPLSLDEYSRYGRQLIVPEFQGVQGQVNLKNSKVLVIGAGGLGCPALLYLCAAGVGKIGIVDNDYVDKSNLHRQVLHTTNRVGMLKCESARVYLENLNPNTEIETFPIRLDNDNVFEVMSGYEYVLDCTDTPQTRYLINDGAVLCGLTIVSGSGLKTEGQLSILNFQNQGPCYRCFYPNPPPPNSVTSCKDGGVIGPAIGMVGIMMAYEAIKLITGYYTMENFSPFLTIYSSYPKQSLRTFKMRGRSEKCAICNGTITRKAIESGEIDYQAFCGTIHYRVMDPENDRISVTKYDELVSTDHSLIDVRPKEQFQICSLPNSINIPIDQLMKKELKDLSQFQRGTPNFFICRYGNDSQSAVEFFRTLGIPSKDIIGGLNCWSNEIDSSMPKY

>tr|G3ATB7|G3ATB7_SPAPN/1438 Adenylyltransferase and sulfurtransferase UBA4 OS=Spathaspora passalidarum (strain NRRL Y27907 / 11Y1) OX=619300 GN=UBA4 PE=3 SV=1MTSPTTEDLLARIATLELENSRLRKDKPQPDLPKPDPSSPTTTLSLDEYKRYGRQMIVPQFGSLPSQLRLKQAKILVVGAGGLGCPALLYLSAAGIGTIGIVDDDVVDISNLHRQVLHTSENVGMYKCESAKLYISRLNYHVNVETYPVRLTNDNAFSIVSKYDLVLDCTDHPAIRYLINDVCVILSKTIVSGSGLKSDGQLSVLNFQTYGPCYRCFYPQPPSPDSVTSCSDGGVIGPAIGLVGTSMAVETIKVLTGYYTRENFKPFLSVYSGYPFQQFRMFKMRGKQKSCEVCGEKPSVTRDKIESNELNYGVFCGRVTFPPLDKQYRILPSEYHDFVQNKKHLLIDVRPKEQFAITSLPNSINIEWDPIFRKADSLTEYLPEGTSKDDPIIVVCRFGNDSQLAAQKLINMNYSNVKDIIGGLYKWSDDVDDSIPKY

>tr|G3B353|G3B353_CANTC/1435 Adenylyltransferase and sulfurtransferase UBA4 OS=Candida tenuis (strain ATCC 10573 / BCRC 21748 / CBS 615 / JCM 9827 / NBRC 10315 / NRRL Y1498 / VKM Y70) OX=590646 GN=UBA4 PE=3 SV=1MENLTKQELLERVATLQQENAVLRSLASAPEPSPSLSRQESIANLSLEEYMRYGRQMIVPEFGALESQLVLKRSKILVVGAGGLGCPALLYLTAAGVGEIGIVDNDIVDVSNLHRQVLHSTDTVGMLKCHSAAVMLARLNPHVTVKEYPIRLANDNVFEIFEKYDLILDCTDAPAVRYLVNDAAVILNKTVVSGSGLKTDGQWTILNFAGVGPCYRCFHPKPPAPDSVTSCQDGGVLGPAIGLIGINMALETIKVLTGHYKPEQFQPFMCAFYGYHFQQYRTFKMRGRQKSCQVCGEQPTVTRTLIERGELNYQVFCGRSEPYTLPNELRVSVEEYHQNGGTLIDVRPAEQFSVVALPNSVNIPWGNEFLKLESLESYLPGVPKDKAIFVVCRYGNDSQIATKTIREKFGFTNVRDIKGGLNRWSEEVDKDFPKY

>tr|G8BCB7|G8BCB7_CANPC/1433 Adenylyltransferase and sulfurtransferase UBA4 OS=Candida parapsilosis (strain CDC 317 / ATCC MYA4646) OX=578454 GN=UBA4 PE=3 SV=1MTPTTEDELRDKIRLLELENETLRSSKRTSNVYSKVDEYFSLDEYKRYGRQMIVPQFGSLESQKKLKMSKILVVGAGGLGSPVLEYLSAAGVGAIGILDDDIVDMSNLHRQVIHQTTTVGTLKCESAKAFIRKINPHVNIFTHPMRLSNDNAFEIFNQYDLVLDCTDHPAVRYLINDVCVLLGKTIVSGSGLKADGQFTILNFRNIGPCYRCFYPQAPSPSTVTSCADGGVIGPAIGMVGIAMAMETIKVLTEYYNEENFKPFLSSYSAYPHQQIRNFKMRPKQINCIACGKRREITKEKIENGEIDYVAFCGKVAYEPLDQKHRVSVKDYHGILNSGKVHKLIDVRPKEQYNITKLPNSVNIEWDPVFRKLESLEEYLPIQKSDDIYLICRYGNDSQLATKKLHDLGFQNAKSIDGGIDKWSEVINSSIPKY

>tr|H0GHF8|H0GHF8_SACCK/1440 Adenylyltransferase and sulfurtransferase UBA4 OS=Saccharomyces cerevisiae x Saccharomyces kudriavzevii (strain VIN7) OX=1095631 GN=UBA4 PE=3 SV=1MNDYHLEDTTSELEALRLENAQLREQLAKREDSSRDYPLSLEEYQRYGRQMIVEETGGVAGQVKLKNTKVLVVGAGGLGCPALPYLAGAGVGQIGIVDNDVVETSNLHRQVLHDSSRVGMLKCESARQYITKLNPHINVVTYPVRLNSSNAFDIFKGYNYILDCTDSPLTRYLVSDVAVNLGITVVSASGLGTEGQLTILNFNNIGPCYRCFYPTPPPPNAVTSCQEGGVIGPCIGLVGTMMAVETLKLILGIYTNENFSPFLMLYSGFPQQSLRTFKMRGRQEKCLCCGKNRTITKEAIEKGEINYELFCGARNYNVCEPDERISVDAFQRIYKDDEFLAKHIFLDVRPSHHYEISHFPEAVNIPIKNLRDMNGDLKKLQEKLPSVEKDSNIVILCRYGNDSQLATRLLKDKFGFSNVRDVRGGYFKYIDDIDQTIPKY

>tr|I6NDX0|I6NDX0_ERECY/1439 Adenylyltransferase and sulfurtransferase UBA4 OS=Eremothecium cymbalariae (strain CBS 270.75 / DBVPG 7215 / KCTC 17166 / NRRL Y17582) OX=931890 GN=UBA4 PE=3 SV=1MPAEKELLLQELEALKLENFKLKKQLEQQREQKETLPMSLEEYSRYGRQMIVEQINGVEGQVKLREAKVLVIGAGGLGCPSLPYLVGAGVGHIGIVDDDIVDASNLHRQILHDSVKVGMLKCESAKQVLNKLNPHVQVATYPVRLDNKNAFQIMANYDIIMDCTDTPLARYLISDVAVNLGKTVVSASGLGTEGQLSIYNFKNVGPCYRCFYPNPPPPGSVTSCQQGGVVGPCIGLVGVMMAVETMKVILETYTVENFSPFLIQYSGFPQQSLRTFKMRGKKKDCISCGYNPTVTREAIESGDVNYSNFCGGSRDFNVLADEERITVQQFQENYWLDDKKDYILLDVRPRLHYNVSHLPKTYNVTMKELKDMDGDLKELQKRIPRLTTERDIIVLCRHGNESRLATRVLKDEFRLNNVKDIKGGYFQYIDEISPFLPKY

>tr|K0KR80|K0KR80_WICCF/1429 Adenylyltransferase and sulfurtransferase UBA4 OS=Wickerhamomyces ciferrii (strain ATCC 14091 / BCRC 22168 / CBS 111 / JCM 3599 / NBRC 0793 / NRRL Y1031 F6010) OX=1206466 GN=UBA4 PE=3 SV=1MGQEDIKNELEKLRLENERLRKELASKDSNQINETYTPNSSDFTLDEYKRYGRQMIVPQVTIEGQKKLKNAKVLVIGAGGLGCPALLYLGGAGIGKIGIVDDDTVDISNLHRQVLHTTDKVGEFKCESAKSYINKLNPHVEVQTYPFRLSTCNAFEIFEQYDLVLDCTDSPYSRYLISDVATITKIPVVSGSGLKTEGQLSILNFENKGPCYRCFYPKPPPPNSVTSCKDGGVIGPVIGLMGVMMALETIKVIVGSYSDGNFKPFLSIYSGYEQQSIKTFKMRGRKADCLTCSGQITKEIITKGDINYAEFCGVVNYNVLKPEERISIEEYHKLVESDHVLLDVRPPEHFQIIHLPNSVNIPLDRLKRIESLENLGFDSTKNIYTICRFGNDSQYASRYLNDTFDLKTKDISGGLFQWSNKIDPKFPTY

>tr|N1P4N4|N1P4N4_YEASC/1440 Adenylyltransferase and sulfurtransferase UBA4 OS=Saccharomyces cerevisiae (strain CEN.PK1137D) OX=889517 GN=UBA4 PE=3 SV=1MNDYHLEDTTSELEALRLENAQLREQLAKREDSSRDYPLSLEEYQRYGRQMIVEETGGVAGQVKLKNTKVLVVGAGGLGCPALPYLAGAGVGQIGIVDNDVVETSNLHRQVLHDSSRVGMLKCESARQYITKLNPHINVVTYPVRLNSSNAFDIFKGYNYILDCTDSPLTRYLVSDVAVNLGITVVSASGLGTEGQLTILNFNNIGPCYRCFYPTPPPPNAVTSCQEGGVIGPCIGLVGTMMAVETLKLILGIYTNENFSPFLMLYSGFPQQSLRTFKMRGRQEKCLCCGKNRTITKEAIEKGEINYELFCGARNYNVCEPDERISVDAFQRIYKDDEFLAKHIFLDVRPSHHYEISHFPEAVNIPIKNLRDMNGDLKKLQEKLPSVEKDSNIVILCRYGNDSQLATRLLKDKFGFSNVRDVRGGYFKYIDDIDQTIPKY

>tr|W6MHC0|W6MHC0_9ASCO/1436 Adenylyltransferase and sulfurtransferase UBA4 OS=Kuraishia capsulata CBS 1993 OX=1382522 GN=UBA4 PE=3 SV=1MREKSINDLAKELELLKLENRRLSELIATPTVTSRETSQETTPDLDLPLSLDEYKRYGRQMIVPQLRLNGQLKLKSAKILVVGAGGLGCPALYYLCGAGVGTIGIVDHDTVDISNLHRQILHSNKTVGMLKCDSAQKKLNELNPLVIIITHPVAIANDNVFEIMKDYDLVLDCTDTPATRYLVNDAAVILGKTIVSGSGVKTEGQLSILNFSQTGPCYRCFYPSPPPPNSVTACSDGGVIGPIIGLVGTMMAIEAIKVVTGSYTVESFQPFLSLYSGYPQQSLRTFKMRGKSPKCAVCSTNATLTRDMVESGAIDYSAFCGKVDYNVVDQRTERITVSEYTSLETKGCLLDVRPKEQFEICSLPGSVSIPLAQLQRCHSEDLKGLYEPLYIVCRYGNDSQLAARELRNKFGFEKVKDLIGGLNAWSQEIDDTFPRY

>tr|I2K073|I2K073_DEKBR/1385 Uba4p OS=Brettanomyces bruxellensis AWRI1499 OX=1124627 GN=AWRI1499_1495 PE=4 SV=1MQVPEFGGLSSQLKLKNSKILVIGAGGLGSPALLYLAAAGVGKIGIVDNDVVDRTNLHRQIIHDTSTLNLNKAESARIKMRLLNSNIEVVTYPVALTNLNSFQIVEPYDLVLDCTDTPASRYLINDTCVLLGKTIISGSGLRTEGQLTILNFHNQGPCYRCFYPTPPPPNTVTSCSNGGVIGPCIGVLGTMMAVEALKCVVGFYTQENFHPFLTLYSGFAPGQKLRTFXMRSRMPKCKVCNPDVREITREKIEQGVIDYKGWCGAMNYNVLNPQTQRITAKQLSKVDFSDVEHLNPLLDVRPRIQYDISHIDGSINIPYDDLSRSKSVLEGLDPHKKTYVICRFGNDSQLSTKILLERNFEDVKDVIGGLDSWKKDVNADFPSYW

**Sequence retrieve by BlastP with Human MOCS3 protein sequence (O95396)**

>tr|A0A2Y9FPF4|A0A2Y9FPF4_PHYMC/1455 Adenylyltransferase and sulfurtransferase MOCS3 OS=Physeter macrocephalus OX=9755 GN=MOCS3 PE=3 SV=1MAARQEVLTLQAEVAQREEELSSLKQRLAAALLAEQESERLVPVSPLPPKAALSRDEILRYSRQLVLPELGVQGQLRLATASVLVVGCGGLGCPLAQYLAAAGVGRLGLVDYDVVEGSHLARQVLHGEALAGQAKVFSAAATLRRLNSAVECVPYAQALTPATALDLVGRYDVVADCSDNVPTRYLVNDACVLAGRPLVSASALRFEGQLTVYHYGGGPCYRCVFPQPPPAETVTSCADGGVLGVVTGVLGCLQALEVLKIAAGLGPSYSGSLLLFDALGGHFRCIRLRRRRPDCAASGERPTVTDLQDYESFCGSSATDECRSLQLLSPEERISVVDYKRLLDSGSPHLLLDVRPPVEVDLCRLPHSLHIPLKHLERRNAESLKLLGEAIREGKQGTQEGASLPIYVICKLGNDSQKAVKILQSWTDLDSLTVRDVVGGLMAWAAKIDGTFPQY

>tr|A0A6J2MPJ1|A0A6J2MPJ1_9CHIR/1458 Adenylyltransferase and sulfurtransferase MOCS3 OS=Phyllostomus discolor OX=89673 GN=MOCS3 PE=3 SV=1MAAKAEVRALQAEVARREKELSSLKQRLAAALLAEEEPERPAPVSPLPPKAALTREEILRYSRQLVLPELGVQGQLRLTAASVLVVGCGGLGCPLAQYLAAAGVGRLGLVDYDVVEMSNLPRQVLHGEALAGQAKVFSAAASLRSLNSAVECVPYAQALTPATALDLVRRYDVVADCSDNAPTRYLVNDACVLAGRPLVSASALRFEGQITVYHYDGGPCYRCVFPQPPPADTVTNCADGGVLGVVTGVLGCLQALEVLKIAAGLGPSYSGQLMLFDALRGRFRCIRLRSRRPDCAACGEQPTVTDLQDYEAFCGSSATDKCRSLQLLSPEERVSVTDYKRLLDSGSPHVLLDVRPQVEVDICRLPHALHIPLKQLERRDAESLRLLGEAIQKGKRGTQEGAALPIYVICKLGNESQKAVKILQTLMAVQELGSLTVQDVVGGLMAWAAKIDGTFPQY

>tr|A0A6P3QQB2|A0A6P3QQB2_PTEVA/1458 Adenylyltransferase and sulfurtransferase MOCS3 OS=Pteropus vampyrus OX=132908 GN=MOCS3 PE=3 SV=1MATREEVLALQAEVAQREKELGSLKQRLAAALLAEQEPELLVPVSPLPAKAALSRDEILRYSRQLVLPELGVHGQLRLATASVLIVGCGGLGCPLAQYLAAAGVGRLGLVDYDVVEMSNLPRQVLHGEALAGQAKVFSAAASLRRLNSAVECVPYAQALTPATALDLVRRYDVVADCSDNVPTRYLVNDACVLAGRPLVSASALRFEGQITVYHYEGGPCYRCVFPQPPPAETVTNCADGGVLGVVTGVLGCLQALEVLKIAAGLGPSYSGSLLLFDALRGRFRCIQLRSRRPDCAACGERPTVTDLQDYEAFCGCSATDKCRSLQLLSPEERVSVTDYKRLLDSGSPHLLLDVRPQVEADICRLPHALHIPLKHLERRDTESLKLLGEAIQKGKQGAHEGVALPIYVICKLGNDSQKAVRILQSLTAARELDSSTFQDVVGGLMAWATKIDGTFPQY

>tr|A0A7J7ENS3|A0A7J7ENS3_DICBM/1458 Adenylyltransferase and sulfurtransferase MOCS3 OS=Diceros bicornis minor OX=77932 GN=MOCS3 PE=3 SV=1MAAREEVLALQAEVAQREAELSSLKQRLAAALLADQEPERLVPVPPLPPKAALSRDEILRYSRQLVLPELGVRGQLRLATASVLVVGCGGLGCPLAQYLASAGVGRLGLVDYDVVEMSNLPRQVLHGEALAGQAKVLSAAASLRRLNSAVECVPYAQALTPATALDLVRRYDVVADCSDNVPTRYLVNDACVLAGRPLVSASALRFEGQITVYHYDGGPCYRCVFPQPPPAETVTNCADGGVLGVVTGVLGCLQALEALKIAAGLGPSYSGSLLLFDALRGHFRCIKLRSRRPDCAACGERPTVTDLQDYEAFCGSSATDKCRSLQLLSPEERVSVADYKRLLDSGSPHLLLDVRPQVEVDICRLPHALHIPLKHLQRRDAESLKLLGEAMRKGKQGAQEGAALPIYVICKLGNDSQKAVKILQSLTAARELDSLTVQDVVGGLMAWAAKIDGTFPQY

>sp|A2BDX3|MOCS3_MOUSE/1460 Adenylyltransferase and sulfurtransferase MOCS3 OS=Mus musculus OX=10090 GN=Mocs3 PE=1 SV=1MAAPEDVAALQAEITRREEELASLKRRLAAALTAEPEPERPLRVPPPPLAPRAALSRDEILRYSRQLLLPELGVRGQLRLAAAAVLVVGCGGLGCPLAQYLAAAGVGRLGLVDHDVVETSNLARQVLHGEAQAGESKARSAAAALRRLNSAVECVAYPRALAEDWALDLVRGYDVVADCCDNVPTRYLVNDACVLAGRPLVSASALRFEGQMTVYHHDGGPCYRCVFPRPPPPETVTNCADGGVLGAVPGVLGCAQALEVLKIAAGLGSSYSGSMLLFDGLGGHFRRIRLRRRRPDCVVCGQQPTVTRLQDYEAFCGSSATDKCRALKLLCPEERISVTDYKRLLDSGAPHVLLDVRPQVEVDICRLPHSLHIPLSQLERRDADSLKLLGAALRKGKQESQEGVALPVYVICKLGNDSQKAVKVLQSLTAVPELDSLTVQDIVGGLMAWAAKIDGTFPQY

>sp|A5GFZ6|MOCS3_PIG/1455 Adenylyltransferase and sulfurtransferase MOCS3 OS=Sus scrofa OX=9823 GN=MOCS3 PE=3 SV=1MAAREEVLALQAEVARREEELSSLKHRLAAALLAEQESERLLPVSPLPPKAALSQDEILRYSRQLVLPELGVQGQLRLATASVLIVGCGGLGCPLAQYLAAAGVGRLGLVDYDVVEVSNLARQVLHGEALAGQAKVFSAAASLRRLNSAVECVPYAQALTPATALDLVRRYDVVADCSDNVPTRYLVNDACVLAGRPLVSASALRFEGQITVYHYGGGPCYRCVFPQPPPAETVTNCADGGVLGVVTGVLGCLQALEVLKIAAGLGPSYSGSLLLFDALRGLFRRIQLRRRRPDCAACGERPTVTELQDYEGFCGSSATDKCRSLQLLSPEERVSVIDYKRLLDSGSPHLLLDVRPQVEVDICRLPHALHIPLKHLERRDAESLKLLGEAIREGKQGTQEGASLPIYVICKLGNDSQKAVKILQSLPDLDSLLVQDVVGGLMAWAAKVDGTFPQY

>tr|D4A8L5|D4A8L5_RAT/1414 Adenylyltransferase and sulfurtransferase MOCS3 OS=Rattus norvegicus OX=10116 GN=Mocs3 PE=3 SV=2MPLPSRAALSRDEILRYSRQLLLPELGVRGQLRLAAASVLVVGCGGLGCPLAQYLAAAGVGRLGLVDHDVVETSNLARQVLHGEAQAGHAKAWSAAAALRRLNSAVEYVPYARALSEAWALDLVRGYDVVADCSDNVPTRYLVNDACVLAGRPLVSASALRFEGQMTVYHYDDGPCYRCVFPRPPPAETVTNCADGGVLGVVPGVLGCVQALEVLKIAAGLGTTYSGSMLLFDGLGGHFRRIRLRRRRPDCVVCGQQPTVTCLKNYEAFCGSSATDKCRSLKLLSPEERISVTDYKRLLDSGVPHVLLDVRPQVEVDICRLQHSLHIPLSLLERRDADSLKLLGAALQEEKRNSQEGAALAVYVICKLGNDSQKAVRVLQSLTAVPELDSLKVQDISGGLMAWAAKIDGTFPQY

>tr|F6Q0Z3|F6Q0Z3_HORSE/1458 Adenylyltransferase and sulfurtransferase MOCS3 OS=Equus caballus OX=9796 GN=MOCS3 PE=3 SV=2MAARDKVLALQAEVAQREEELSCLKQRLAAALLAEQKAERRVPVSPLPPKAALSRDEILRYSRQLVLPELGVHGQLRLATASVLVVGCGGLGCPLAQYLAAAGVGRLGLVDYDVVEMSNLARQVLHGESLAGQAKVLSAAAALRRLNSAVECVPYAQALTPATALDLVRRYDVVADCSDNVPTRYLVNDACVLAGRPLVSASALRFEGQITVYHYHGGPCYRCVFPQPPPAETVTNCADGGVLGIVTGVVGCLQALEVLKIAAELGPSYSGSLLLFDALRGHFRCIKLRSRRPDCAACGERPTVTDLQDYEAFCGSSATDKCRSLQLLSPEERVSVTDYKRLLDSGAPHLLLDVRPQVEVDICRLPHALHIPLKHLQRRDAESLKLLGEAIRKGKQGTQEGAALPIYVICKLGNDSQKAVKILQSLTAVQELESLTVQDVVGGLMAWAARIDETFPQY

>tr|G3TPH1|G3TPH1_LOXAF/1460 Adenylyltransferase and sulfurtransferase MOCS3 OS=Loxodonta africana OX=9785 GN=MOCS3 PE=3 SV=1MAARDEVLALRTEVAQREEELSSLKQRLAAALLEKQESESERLVLVSPLPPKAALSRDEILRYSRQLVLPELGVHGQLRLATASVLVVGCGGLGCPLAQYLAAAGVGRIGLVDYDVVEMSNLPRQVLHDEALAGQAKAFSAAAALRRLNSAVECVPYAQALTPATALDLVRRYDVVADCSDNVPTRYLVNDACVLAGRPLVSASALRFEGQITVYHYDGGPCYRCVFPQPPPAETVTNCADGGVLGVVTGVLGCLQALEVLKIAAGLGPSYSGSLLLFDALRGHFRCIRLRKRRPNCAACGERPTVTDLQDYEAFCGSSATDKCRSLQLLSPKERVSVTEYKQLLDSGTSHLLLDVRPQVEVDICRLPHALHIPLKRLERGDAESLKLLREAVRERKQGVQEGAAVPVYVICKLGNDSQKAVKILQSLTAAQELDSLTVQDVVGGLMAWAAKIDDTFPQY

>tr|L5JXV4|L5JXV4_PTEAL/1458 Adenylyltransferase and sulfurtransferase MOCS3 OS=Pteropus alecto OX=9402 GN=MOCS3 PE=3 SV=1MATREEVLALQAEVAQREKELGSLKQRLAAALLAEQEPELLVPVSPLPAKAALSRDEILRYSRQLVLPELGVHGQLRLATASVLIVGCGGLGCPLAQYLAAAGVGRLGLVDYDVVEMSNLPRQVLHGEALAGQAKVFSAAASLRRLNSAVECVPYAQALTPATALDLVRRYDVVADCSDNVPTRYLVNDACVLAGRPLVSASALRFEGQITVYHYEGGPCYRCVFPQPPPAETVTNCADGGVLGVVTGVLGCLQALEVLKIAAGLGPSYSGSLLLFDALRGRFRCIQLRSRRPDCAACGERPTVTDLQDYEAFCGCSATDKCRSLQLLNPEERVSVTDYKRLLDSGSPHLLLDVRPQVEADICRLPHALHIPLKHLERRDTESLKLLGEAIQKGKQGAQEGVALPIYVICKLGNDSQKAVRILQSLTAARELDSSTFQDVVGGLMAWATKIDGTFPQY

>tr|M3X4P1|M3X4P1_FELCA/1469 Adenylyltransferase and sulfurtransferase MOCS3 OS=Felis catus OX=9685 GN=MOCS3 PE=3 SV=2MLFRTTSGRGAMASREEVLALQAQVAQREEELSFLKQKLAAAILAEQEPERLVSVSPLPPKASLSRDEILRYSRQLVLPELGVQGQLRLAAASVLVVGCGGLGCPLAQYLAAAGVGRLGLVDYDVVEMSNLARQVLHGEALAGQAKVFSAAASLRRLNSAVECVPYAEALTPATALDLVRRYDVVADCSDNVPTRYLVNDACVLARRPLVSASALRFEGQITVYHYDGGPCYRCVFPQPPPAETVTNCADGGVLGVVTGVLGCLQALEVLKIAAGLGPSYSGSLLIFDALRGHFRCIQLRGRRPDCAACGERPTVTALQDYEAFCGSSATDKCRSLRLLSPEERVSVTDYKRLLDSGSPHLLLDVRPQVEVDICRLPHALHIPLKRLERRDAESLELLGEAIREGKQGTQEGAAFPVYVICKLGNDSQKAVKILQSLTAVQELESYTVQDVVGGLMAWAAKVDGTFPQY

>sp|O95396|MOCS3_HUMAN/1460 Adenylyltransferase and sulfurtransferase MOCS3 OS=Homo sapiens OX=9606 GN=MOCS3 PE=1 SV=1MASREEVLALQAEVAQREEELNSLKQKLASALLAEQEPQPERLVPVSPLPPKAALSRDEILRYSRQLVLPELGVHGQLRLGTACVLIVGCGGLGCPLAQYLAAAGVGRLGLVDYDVVEMSNLARQVLHGEALAGQAKAFSAAASLRRLNSAVECVPYTQALTPATALDLVRRYDVVADCSDNVPTRYLVNDACVLAGRPLVSASALRFEGQITVYHYDGGPCYRCIFPQPPPAETVTNCADGGVLGVVTGVLGCLQALEVLKIAAGLGPSYSGSLLLFDALRGHFRSIRLRSRRLDCAACGERPTVTDLLDYEAFCGSSATDKCRSLQLLSPEERVSVTDYKRLLDSGAFHLLLDVRPQVEVDICRLPHALHIPLKHLERRDAESLKLLKEAIWEEKQGTQEGAAVPIYVICKLGNDSQKAVKILQSLSAAQELDPLTVRDVVGGLMAWAAKIDGTFPQY

>tr|A0A1U7TG44|A0A1U7TG44_CARSF/1460 Adenylyltransferase and sulfurtransferase MOCS3 OS=Carlito syrichta OX=1868482 GN=MOCS3 PE=3 SV=1MAARQEVLALQAEVARREEELSSLKQRLAAALLEEQEPTPERPVQVSPLPSKAALSRDEILRYSRQLVLPELGVHGQLRLATASVLIVGCGGLGCPLAQYLAAAGVGRLGLVDYDVVEMSNLARQVLHGEALAGQAKAFSAAASLRRLNSAVECVPYAQALTPATALDLVRRYDVVADCSDNVPTRYLVNDACVLAGRPLVSASALRFEGQITVYHYDGGPCYRCVFPQPPPAETVTNCADGGVLGVVTGVLGCLQALEVLKIAAGMGPSYSGSLLLFDALRGHFRCIQLRSRKPDCAACGERPTVTDLQDYEAFCGSSATDKCRSLQLLSPAERVSVTYYKRLLDSGAPHLLLDVRPQVEVDICRLPHALHIPLKQLERRDPESLKLLGEAIREGKRDAQEGAADSIYVICKLGNDSQKAVKILQCLTAAQVLDPVTVRDVVGGLMAWAAKIDGTFPQY

>tr|A0A2K5BVE4|A0A2K5BVE4_AOTNA/1461 Adenylyltransferase and sulfurtransferase MOCS3 OS=Aotus nancymaae OX=37293 GN=MOCS3 PE=3 SV=1MASREEVLALQAEVARREEELYSLKQKLAAALLSEQKPQPERLVPVSPLPPKAVLSRDEILRYSRQLVLPELGVHGQLRLATASVLVVGCGGLGCPLAQYLAAAGVGRLGLVDYDVVEMSNLARQVLHGEALAGQAKAFSAAASLRRLNSEVECVPYTQALTPATALDLVRRYDVVADCSDNVPTRYLVNDACVLAGRPLVSASALRFEGQITVYHYDGGPCYRCIFPQPPPAETVTNCADGGVLGVVTGVLGCLQALEVLKIAAGLGPSYSGSLLLFDALSGHFRCIRLRNRRLDCAACGERPTVTDLLDYEAFCGSSATDKCLSLQLLSPEERVSVTDYKRLLDSGASHLLLDVRPQVEVDICRLPHALHIPLKHLERRDAESLKLLGEAIQEGKQRTQEGAALPIYVICKLGNDSQKAVKILQSLSAAQELDSSLTVRDVVGGLMAWAAKIDGTFPQY

>tr|A0A2U3WT65|A0A2U3WT65_ODORO/1469 Adenylyltransferase and sulfurtransferase MOCS3 OS=Odobenus rosmarus divergens OX=9708 GN=MOCS3 PE=3 SV=1MPFKTTSERGAMASREEVLALQAEVSRREEELSSLKQRLAAALLAEQEPGRLVPVSPLPPKAALSRDEILRYSRQLVLPELGVHGQLRLATASVLVVGCGGLGCPLAQYLAAAGVGRLGLVDYDVVEVSNLARQVLHGEALAGQAKVFSAAAALRRLNSAVECVPYAQALTPATALDLIRRYDVVADCSDNVPTRYLVNDACVLAGRPLVSASALRFEGQITVYHYDGGPCYRCIFPQPPPAETVTNCADGGVLGVVTGVLGCLQALEVLKIAAGLGPSYSRSLLIFDALRGQFRCIQLRSRRPDCAACGERPTVTDLQDYEAFCGSSATEKCRSLQLLSPEERVSVTDYKRLLDSGSPHLLLDVRPQVEVDICRLPHALHIPLQHLERRNAESLELLGEAIREGKQGTQEGVAFPVYVICKLGNDSQKAVKILQSLTAVQELESLTVQDVVGGLMAWAARIDGTFPQY

>tr|A0A2U3YL40|A0A2U3YL40_LEPWE/1469 Adenylyltransferase and sulfurtransferase MOCS3 OS=Leptonychotes weddellii OX=9713 GN=MOCS3 PE=3 SV=1MPFKTTSERGAMASREEVLALQAEVSRREEELSSLKQRLAAALLAEQETERLVPVSPLPPKAALSRDEILRYSRQLVLPELGVHGQLRLATASVLVVGCGGLGCPLAQYLAAAGVGRLGLVDYDVVEVSNLARQVLHGEALAGQAKVFSAAAALRRLNSAVECVPYAQALTPATALDLIRRYDVVADCSDNVPTRYLVNDACVLAGRPLVSASALRFEGQITVYHYDGGPCYRCIFPQPPPAETVTNCADGGVLGVVTGVLGCLQALEVLKIAAGLGPSYSRSLLIFDALRGQFRCIQLRSRRPDCAACGERPTVTDLQDYEAFCGSSATEKCRSLQLLSPEERVSVTDYKRLLDSGSPHLLLDVRPQVEVDICRLPHALHIPLQHLERRNAESLELLGEAIRERKQGTQERVAFPVYVICKLGNDSQKAVKILQSLTAVQELESLTVQDVVGGLTAWAAKIDGTFPQY

>tr|A0A2Y9D997|A0A2Y9D997_TRIMA/1460 Adenylyltransferase and sulfurtransferase MOCS3 OS=Trichechus manatus latirostris OX=127582 GN=LOC101357096 PE=3 SV=1MAANEEVLALRAEVTQREEELSSLKQRLAAALLAEEESEPERLVPVSPLPPKAALSRDEILRYSRQLVLPELGVHGQLRLATASVLVVGCGGLGCPLAQYLAAAGVGRIGLVDYDVIEMSNLARQVLHDEALAGRAKALSAAASLRRLNSAVECVPYAQALTPATALDLVRRYDVVADCSDNVPTRYLVNDACVLAGRPLVSASALRFEGQITVYHYDGGPCYRCVFPQPPPAETVTNCADGGVLGVVTGVLGCLQALEVLKIAAGLGPSYSGSLLLFDALRGHFRCIQLRKRRPDCAVCGERPTVTDLQDYEAFCGSSATDKCRSLHLLSPKERVSVTDYKRLLDSGASHLLLDVRPQVEVDICRLPHALHIPLKHLEQRDAESLKLLGEAVRERKQGTQEGAALPVYVICKLGNDSQKAVKILQSLTAAQELDSVTVQDVMGGLMAWAAKIDETFPLY

>tr|A0A2Y9GQW6|A0A2Y9GQW6_NEOSC/1469 Adenylyltransferase and sulfurtransferase MOCS3 OS=Neomonachus schauinslandi OX=29088 GN=MOCS3 PE=3 SV=1MPFKTTSERGAMASREEVLALQAEVSRREEELSSLKERLAAALLAEQEPERLVPVSPLPPKAALSRDEILRYSRQLVLPELGVHGQLRLATASVLVVGCGGLGCPLAQYLAAAGVGRLGLVDYDVVEVSNLARQVLHGEALAGQAKVFSAAAALRRLNSAVECVPYAQALTPATALDIIRRYDVVADCSDNVPTRYLVNDACVLAGRPLVSASALRFEGQITVYHYDGGPCYRCIFPQPPPAETVTNCADGGVLGVVTGVLGCLQALEVLKIAAGLGPSYSRSLLIFDALRGQFRCIQLRSRRPDCAACGERPTVTDLQDYEAFCGSSATEKCRSLQLLSPEERVSVTDYKRLLDSGSLHLLLDVRPQVEVDICRLPHALHIPLQHLERRNAESLELLREAIRERKQGTQERVAFPVYVICKLGNDSQKAVKILQSLTAVQELESLTVQDVVGGLMAWAAKIDGTFPQY

>tr|A0A2Y9IN50|A0A2Y9IN50_ENHLU/1458 Adenylyltransferase and sulfurtransferase MOCS3 OS=Enhydra lutris kenyoni OX=391180 GN=LOC111141597 PE=3 SV=1MGSRQEVLALRAEVARREEELSSLKQRLAAALLAEQEPERLVPVSPLPPKAALSRDEILRYSRQLVLPELGVHGQLRLATASVLIVGCGGLGCPLAQYLAAAGVGRLGLVDYDVVEVSNLARQVLHGEALAGQAKVFSAAASLRRLNSAVECVPYAQALTAATALDLIRRYDVVADCSDNVPTRYLVNDACVLAGRPLVSASALRFEGQITVYHYDGGPCYRCIFPQPPPAETVTNCADGGVLGVVTGVLGCLQALEVLKIVAGLGPSYCRSLLIFDALRGQFRCIRLRSRRPDCAACGERPTVTDLQDYEAFCGSSATEKCRALHLLSPEERVSVTDYKRLLDSGSPHLLLDVRPQVEVDICRLPHALHIPLKHLERRNAESLELLGEAIREGKQGAQEGAAFPVYVICKLGNDSQKAVKVLQSLTAVQELESSTFQDVVGGLMAWAAKIDGTFPQY

>tr|A0A3L8S609|A0A3L8S609_CHLGU/1477 Adenylyltransferase and sulfurtransferase MOCS3 OS=Chloebia gouldiae OX=44316 GN=MOCS3 PE=3 SV=1MRERPAAEPARGDARDAEDEDGARGPRQPSAAELARGDSGDADEEDEDDDNEEEEGAPGFPAELPPLPARSALSAAEILRYSRQLVLPELGVRGQLRLARSSVLVVGCGGLGCPLAQYLAAAGVGRLGLVDHDVVETSNLQRQVLHGEARRGRPKARSAAAALRRLNSAVQYVPYRGALRPRSALRLVRQYDIVADCSDNVPTRYLVSDACVLAGKPLVSGSALRLEGQLAVYNYRGGPCYRCLFPRPPPPDAVTNCADGGVLGAVTGVIGCMQALEVLKIASGMGSSFGRCMLMFDALEGSFRNIKLRPRNADCAACGDSPSVTCLQDYEAFCGSSATDKCRALRLLPGGDRISVWRYKELLDAREPHVLLDVRPRVEAEICRLPHALHIPLRKLEEKDEEALQSLQRRICEEKQRTDGQTSLPVYVVCKLGNDSQKAVKILQELPAEEFGSVSAKDIKGGLMAWATKIDSTFPQY

>tr|A0A3M0J6D7|A0A3M0J6D7_HIRRU/1469 Adenylyltransferase and sulfurtransferase MOCS3 OS=Hirundo rustica rustica OX=333673 GN=MOCS3 PE=3 SV=1MAGAAAARLRAEIRRRERELRGLRERLAAELARGDTAEQEEDGEEEREREEGARALPPLPARSALSAAEILRYSRQLVLPELGVRGQLRLARSSVLVVGCGGLGCPLAQYLAAAGVGRLGLVDHDVVETSNLQRQVLHGEARRGRPKARSAAAALRRLNSAVQYVPYRGALRPRSALRIVRQYDIVADCSDNVPTRYLVSDACVLAGKPLVSGSALRLEGQLAVYNHGGGPCYRCLFPEPPPPDAVTNCADGGVLGVVTGVIGCMQALEVLKIASGMGSTFNGHMLMFDALEGRFRNIKLRPRRADCAVCGDSPSVTCLQDYEAFCGSSATDKCRALQLLPAGDRISVQRYKELLDERVPHVLLDVRPQVEVDICRLEHAVHIPLRKLEEKDEESLRHLQDRISEEKQRTDGQASVPVYVVCKLGNDSQKAVKILQELPAEEFGSVLAKDIKGGLMAWATKIDSTFPQY

>tr|A0A3Q7MPZ6|A0A3Q7MPZ6_CALUR/1469 Adenylyltransferase and sulfurtransferase MOCS3 OS=Callorhinus ursinus OX=34884 GN=MOCS3 PE=3 SV=1MPFKTTSERGAMASREEVLALQAEVSRREEELSSLKQRLAAALLAEQEPGRLVPVSPLPPKAALSRDEILRYSRQLVLPELGVHGQLRLATASVLVVGCGGLGCPLAQYLAAAGVGRLGLVDYDVVEVSNLARQVLHGEALAGQAKVFSAAAALRRLNSAVECVPYAQALTPATALDLIRRYDVVADCSDNVPTRYLVNDACVLAGRPLVSASALRFEGQITVYHYDGGPCYRCIFPQPPPAETVTNCADGGVLGVVTGVLGCLQALEVLKIAAGLGPSYSRSLLIFDALRGQFRCIQLRSRRPDCAACGERPTVTDLQDYEAFCGSSAIEKCRSLQLLSPEERVSVTDYKRLLDSGSPHLLLDVRPQVEVDICRLPHALHIPLQHLERRNAESLELLGEAIREGKQGTQEGVTFPVYVICKLGNDSQKAVKILQSLTAVQELESLTVQDVVGGLMAWAARIDGTFPQY

>tr|A0A3Q7TLI3|A0A3Q7TLI3_VULVU/1469 Adenylyltransferase and sulfurtransferase MOCS3 OS=Vulpes vulpes OX=9627 GN=MOCS3 PE=3 SV=1MPFRTASGSGAMASREEVLALQAEVARREEELSSLKQRLAAALLAEQAPERLVPVSPLPPKAALSREEILRYSRQLVLPELGVHGQLRLAAASVLVVGCGGLGCPLAQYLAAAGVGRLGLVDYDVVEMSNLARQVLHGEALAGQAKAFSAAASLRRLNSAVECVPYAQALTPATALDLVRRYDVVADCSDNVPTRYLVNDACVLAGRPLVSASALRFEGQITVYHYDGGPCYRCVFPQPPPAETVTNCADGGVLGVVTGVLGCLQALEVLKIAAGLGPSYSRSLLIFDALRGQFRCIQLRSRRLDCAACGERPTVTALQDYEAFCGSSATDKCRSLQLLSPEERVSVADYKRLLDSGSPHLLLDVRPQVEVDICRLPHALHIPLKHLERRNAESLKLLGEAIREGKQGAQAGAAFPVYVICKLGNDSQKAVKILQSLTAVQELESLTVQDVVGGLMAWAAKIDGTFPQY

>tr|A0A452S5A8|A0A452S5A8_URSAM/1458 Adenylyltransferase and sulfurtransferase MOCS3 OS=Ursus americanus OX=9643 GN=MOCS3 PE=3 SV=1MASREEVLALQAEVVRREEELSSLKQRLAAAVLAEQEPERLVPVSPLPPKAALSRDEILRYSRQLVLPELGVHGQLRLATASVLVVGCGGLGCPLAQYLAAAGVGRLGLVDYDVVEMSNLARQVLHGEALAGQAKVFSAAASLRRLNSAVECVPYAQVLTPATALDLVRRYDVVADCSDNVPTRYLVNDACVLAGRPLVSASALRFEGQITVYHYDGGPCYRCIFPQPPPAETVTNCADGGVLGVVTGVLGCLQALEVLKIAAGLGPSYSRSLLIFDALRGQFRCIQLRSRRPDCAACGERPTVTDLQDYEAFCGSSATDKCRSLQLLSPEERVSVTDYKRLLDSGSPHLLLDVRPQVEVDICRLPHALHIPLKHLERRDVQSLELLGEAIREGRQGTQEGAAFPVYVICKLGNDSQKAVKILQSLTAVQDLESLTVQDVVGGLMAWAAKVDGTFPQY

>tr|A0A485NM36|A0A485NM36_LYNPA/1469 Adenylyltransferase and sulfurtransferase MOCS3 OS=Lynx pardinus OX=191816 GN=MOCS3 PE=3 SV=1MLFRTTSGRGAMASREEVLALQAQVAQREEELSFLKQKLAAAILAEQEPERLVSVSPLPPKASLSRDEILRYSRQLVLPELGVQGQLRLAAASVLVVGCGGLGCPLAQYLAAAGVGRLGLVDYDVVEMSNLARQVLHGEALAGQAKVFSAAASLRRLNSAVECVPYAEALTPATALDLVRRYDVVADCSDNVPTRYLVNDACVLAGRPLVSASALRFEGQITVYHYDGGPCYRCVFPQPPPAETVTNCADGGVLGVVTGVLGCLQALEVLKIAAGLGPSYSGSLLIFDALRGHFRCIQLRGRRPDCAACGERPTVTALQDYEAFCGSSATDKCRSLRLLSPEERVSVTDYKRLLDSGSPHLLVDVRPQVEVDICRLPHALHIPLKRLERRDAESLELLGEAIREGKQGTQEGAAFPVYVICKLGNDSQKAVKILQSLTAVQELESYTVQDVVGGLMAWAAKVDGTFPQY

>tr|A0A4X1UHD0|A0A4X1UHD0_PIG/1416 Adenylyltransferase and sulfurtransferase MOCS3 OS=Sus scrofa OX=9823 GN=MOCS3 PE=3 SV=1RLLPVSPLPPKAALSQDEILRYSRQLVLPELGVQGQLRLATASVLIVGCGGLGCPLAQYLAAAGVGRLGLVDYDVVEVSNLARQVLHGEALAGQAKVFSAAASLRRLNSAVECVPYAQALTPATALDLVRRYDVVADCSDNVPTRYLVNDACVLAGRPLVSASALRFEGQITVYHYGGGPCYRCVFPQPPPAETVTNCADGGVLGVVTGVLGCLQALEVLKIAAGLGPSYSGSLLLFDALRGLFRRIQLRRRRPDCAACGERPTVTELQDYEGFCGSSATDKCRSLQLLSPEERVSVIDYKRLLDSGSPHLLLDVRPQVEVDICRLPHALHIPLKHLERRDAESLKLLGEAIREGKQGTQEGASLPIYVICKLGNDSQKAVKILQSLPDLDSLLVQDVVGGLMAWAAKVDGTFPQY

>tr|A0A667G752|A0A667G752_LYNCA/1458 Adenylyltransferase and sulfurtransferase MOCS3 OS=Lynx canadensis OX=61383 GN=MOCS3 PE=3 SV=1MASREEVLALQAQVAQREEELSFLKQKLAAAILAEQEPERLVSVSPLPPKASLSRDEILRYSRQLVLPELGVQGQLRLAAASVLVVGCGGLGCPLAQYLAAAGVGRLGLVDYDVVEMSNLARQVLHGEALAGQAKVFSAAASLRRLNSAVECVPYAEALTPATALDLVRRYDVVADCSDNVPTRYLVNDACVLAGRPLVSASALRFEGQITVYHYDGGPCYRCVFPQPPPAETVTNCADGGVLGVVTGVLGCLQALEVLKIAAGLGPSYSGSLLIFDALRGHFRCIQLRGRRPDCAACGERPTVTALQDYEAFCGSSATDKCRSLRLLSPEERVSVTDYKRLLDSGSPHLLLDVRPRVEVDICRLPHALHIPLKRLERRDAESLELLGEAIREGKQGTQEGAAFPVYVICKLGNDSQKAVKILQSLTAVQELESYTVQDVVGGLMAWAAKVDGTFPQY

>tr|A0A671F1R2|A0A671F1R2_RHIFE/1458 Adenylyltransferase and sulfurtransferase MOCS3 OS=Rhinolophus ferrumequinum OX=59479 GN=MOCS3 PE=3 SV=1MAAREEVLALQAEVAQREEELSALKQRLAAALLAEQEPERLVPLSPLPPKTSLSRDEILRYSRQLVLPELGVHGQLRLATASVLIVGCGGLGCPLAQYLAAAGVGRLGLVDYDVVEMSNLARQVLHGEALAGQAKVYSAAASLRRLNSAVECVPYAQALTPATALDLVRHYDVVADCSDNVPTRYLVNDACVLAGRPLVSASALRLEGQITVYHYDGGPCYRCVFPQPPPAETVTNCADGGVLGVVTGVVGCLQALEVLKIAAGLGPSYSGSLLLFDAFRGRFRCIQLRSRRPDCAACGERPTVTDLQDYEAFCGSSATDKCLSLQLLSPEERISVTDYKRLLDSGSPHLLLDVRPQVEVDICRLPHALHIPLKHLEQRDAGSLKLLGEAIQKGKQSTQDGVVLPIYVICKLGNDSQKAVKILQSVTAVQELDSLTVQDVVGGLMAWAAKIDGTFPQY

>tr|A0A673UJM5|A0A673UJM5_SURSU/1458 Adenylyltransferase and sulfurtransferase MOCS3 OS=Suricata suricatta OX=37032 GN=MOCS3 PE=3 SV=1MASSEEVLALRAQVAQREEELSFLKQKLAAAVLAEQEPQRLVSVSPLPPKASLSRDEILRYSRQLVLPELGVQGQLRLATASVLVVGCGGLGCPLAQYLAAAGVGRLGLVDYDVVEMSNLARQVLHGEALAGQAKVFSAAASLRRLNSAVECVPYAEALTPATALDLVRRYDVVADCSDNVPTRYLVNDACVLAGRPLVSASALRFEGQITVYHYDGGPCYRCVFPQPPPAETVTNCADGGVLGVVTGVLGCLQALEVLKIAAGLGPSYSGSLLIFDALRGHFRRIQLRGRRPDCAACGERPTVTALQDYEAFCGSSATDKCRTLQLLSPEERVSVTDYKRLLDSGSPHLLLDVRPQVEVDICRLPHALHIPLKHLERRDAQSLELLGGAIREGKEGAQEGAAFPVYVICKLGNDSQKAVKILQSLTAAQELESVTVQDVAGGLMAWAAKIDGTFPQY

>tr|A0A6J0A1D5|A0A6J0A1D5_ACIJB/1458 Adenylyltransferase and sulfurtransferase MOCS3 OS=Acinonyx jubatus OX=32536 GN=MOCS3 PE=3 SV=1MASREEVLALQAQVAQREEELSFLKQKLAAAILAEQEPERLVSVSPLPPKASLSRDEILRYSRQLVLPELGVQGQLRLAAASVLVVGCGGLGCPLAQYLAAAGVGRLGLVDYDVVEMSNLARQVLHGEALAGQAKVFSAAASLRRLNSAVECVPYAEALTPATALDLVRRYDVVADCSDNVPTRYLVNDACVLAGRPLVSASALRFEGQITVYHYDGGPCYRCVFPQPPPAETVTNCADGGVLGVVTGVLGCLQALEVLKIAAGLGPSYSGSLLIFDALRGHFRCIQLRGRRPDCAACGERPTVTALQDYEAFCGSSATDKCRSLRLLSPEERVSVTDYKRLLDSGSPHLLLDVRPQVEVDICRLPHALHIPLKRLERRDAESLELLGEAIREGKQGTQEGAAFPVYVICKLGNDSQKAVKILQSLTAVRELESYTVQDVVGGLMAWAAKIDGAFPQY

>tr|A0A6J0HAJ7|A0A6J0HAJ7_9PASS/1520 Adenylyltransferase and sulfurtransferase MOCS3 OS=Lepidothrix coronata OX=321398 GN=MOCS3 PE=3 SV=1MAGAGAARLRAEIRRRERELRGLRDRLQAELARGDTAEQQRDEEEEDGARGPRERLAAEVSLGNAEQEEDGDRGLRDLLARGDAGSAEDDDGDDDDAEGAGAFPAELPPLPPRAALSAAEIARYSRQLLLPELGVRGQLRLARSAVLVVGCGGLGCPLAQYLAAAGVGRLGLLDHDVVETSNLHRQVLHGEARRGRPKAASAAAALRRLNSAVQYVPYCGALSPRSALRLVRQYDIVADCSDNAPTRYLVNDACVLAGKPLVSGSALRLEGQLVVYNYRGGPCYRCLFPEPPPPHTVTSCADGGVLGVVPGIIGCIQALEVLKIASGMGSSFNQFMLVFDALEGRFRNIKLRPKKPDCAVCGDSPTVTCLQDYEAFCGSSATDKCRTLHLLPSKDRISVEQYKKVLDDKVPHVLLDVRPRVEVDICRLEHAVHIPLSKLEEKDEEQLEYLERRIREEKQRTNDQASLPVYVVCKLGNDSQKAVKILQELPAKESGSLLAKDIKGGLMAWATKIDPTFPQY

>tr|A0A6J2FFF5|A0A6J2FFF5_ZALCA/1469 Adenylyltransferase and sulfurtransferase MOCS3 OS=Zalophus californianus OX=9704 GN=MOCS3 PE=3 SV=1MPFKTTSERGAMASREEVLALQAEVSRREEELSSLKQRLAAALLAEQEPGRLVPVSPLPPKAALSRDEILRYSRQLVLPELGVHGQLRLATASVLVVGCGGLGCPLAQYLAAAGVGRLGLVDYDVVEVSNLARQVLHGEALAGQAKVFSAAAALRRLNSAVECVPYAQALTPATALDLIRRYDVVADCSDNVPTRYLVNDACVLAGRPLVSASALRFEGQITVYHYDGGPCYRCIFPQPPPAETVTNCADGGVLGVVTGVLGCLQALEVLKIAAGLGPSYSRSLLIFDALRGQFRCIQLRSRRPDCAACGERPTVTDLQDYEAFCGSSAIEKCRSLQLLSPEERVSVTDYKRLLDSGSPHLLLDVRPQVEVDICRLPHALHIPLQHLERRNAESLELLGEAIREGKQGTQEGVTFPVYVICKLGNDSQKAVKILQSLAAVQELESLTVQDVVGGLMAWAARIDGTFPQY

>tr|A0A6J2J610|A0A6J2J610_9PASS/1522 Adenylyltransferase and sulfurtransferase MOCS3 OS=Pipra filicauda OX=649802 GN=MOCS3 PE=3 SV=2MAGAGAARLRAEIRRRERELRGLRDRLQAELARGDTAEQQQDEEEEDGARGPRERLAAEVSLGDAEQEEDGDRGLRDLLARGDAGSAEDDDGDDDAEGVMMSGAFPAELPPLPPRAALSAAEIARYSRQLLLPELGVRGQLRLARSAVLVVGCGGLGCPLAQYLAAAGVGRLGLLDHDVVETSNLHRQVLHGEARRGRPKAASAAAALRRLNSAVQYVPYCGALSPRSALRLVRQYDIVADCSDNAPTRYLVNDACVLAGKPLVSGSALRLEGQLVVYNYRGGPCYRCLFPEPPPPHTVTSCADGGVLGVVPGIIGCIQALEVLKIASGMGSSFNQFMLVFDALEGRFRNIRLRPKKPDCAVCGANPTVTCLQDYEAFCGSSATDKCRTLHLLPSKDRISVEQYKKVLDDKVPHVLLDVRPRVEVDICRLEHAVHIPLSKLEEKDEEQLEYLEKRIREEKQRTNDQASLPVYVVCKLGNDSQKAVKILQELPAKESGSLLAKDIKGGLMAWATKIDPTFPQY

>tr|A0A6P4WR34|A0A6P4WR34_PANPR/1533 Adenylyltransferase and sulfurtransferase MOCS3 OS=Panthera pardus OX=9691 GN=MOCS3 PE=3 SV=1MWPGSSSPKALLMVPLEISRNYKSLNLNLRVSSGGACLVFAFFPSSVKLFSLFQSLGDGKPEAEMLFRTTSGRGAMASREEVLALQAQVAQREEELSFLKQRLAAAVLAEQEPERLVSVSPLPPKASLSRDEILRYSRQLVLPELGVQGQLRLAAASVLVVGCGGLGCPLAQYLAAAGVGRLGLVDYDVVEMSNLARQVLHGEALAGQAKVFSAAASLRRLNSAVECVPYAEALTPATALDLVRRYDVVADCSDNVPTRYLVNDACVLAGRPLVSASALRFEGQITVYHYDGGPCYRCVFPQPPPAETVTNCADGGVLGVVTGVLGCLQALEVLKIAAGLGPSYSGSLLIFDALRGHFRCIQLRGRRPDCAACGERPTVTALEDYEAFCGSSATDKCRSLRLLSPEERVSVTDYKRLLDSGSPHLLLDVRPQVEVDICRLPHALHIPLKRLERRDAESLELLGEAIREGKQGTQEGAAFPVYVICKLGNDSQKAVKILQSLTAVQELESFTVQDVVGGLMAWAAKIDGTFPQY

>tr|A0A6P6IJ77|A0A6P6IJ77_PUMCO/1469 Adenylyltransferase and sulfurtransferase MOCS3 OS=Puma concolor OX=9696 GN=MOCS3 PE=3 SV=1MLFRTTSGRGAMASRAEVLALQAQVAQREEELSFLKQKLAAAILAEQEPERLVSVSPLPPKASLSRDEILRYSRQLVLPELGVQGQLRLAAASVLVVGCGGLGCPLAQYLAAAGVGRLGLVDYDVVEMSNLARQVLHGEALAGQAKVFSAAASLRRLNSAVECVPYAEALTPATALDLVRRYDVVADCSDNVPTRYLVSDACVLAGRPLVSASALRFEGQITVYHYDGGPCYRCVFPQPPPAETVTNCADGGVLGVVTGVLGCLQALEVLKIAAGLGPSYSGSLLIFDALRGHFRCIQLRGRRPDCAACGERPTVTALQDYEAFCGSSATDKCRSLQLLSPEERVSVTDYKRLLDSGSPHLLLDVRPQVEVDICRLPHALHIPLKRLERRDAESLELLGEAIREGKQGTQEGAAFPVYVICKLGNDSQKAVKILQSLTAVRELESYTVQDVVGGLMAWAAKIDGAFPQY

>tr|A0A6P7XL61|A0A6P7XL61_9AMPH/1546 Adenylyltransferase and sulfurtransferase MOCS3 OS=Microcaecilia unicolor OX=1415580 GN=MOCS3 PE=3 SV=1MFAAVDTKHQDLLIQNLENILNKIKNKTPLINESFQQFLNTKNGYLVSSLVSEFLEFFSLGFTLAVFQPGTGTQVHGPDRGNLAQDLGIVENENTRDGPLLLEVMKRCQNSNSREDGSSDSSLPVLHNLVTELPLLEAKTSLSNEAILRYSRQLVLPELGVKGQLNLSKSSVLIVGCGGLGCPLAQYLAAAGIGRLGLLDYDVVEINNLHRQVLHGENRQGIPKSLSAATALKQLNSGVEYICYNLILTPKIALELIQQYDIIADCSDNVPTRYLVNDACVLTGKPLVSASALRMEGQLTVYNYHGSPCYRCLFPVPPPAETVTNCSDGGVLGVVPGIMGCLQALEVLKIASGLGTSFGQFMLMFDALEGRFRNIRLRPKNQNCAVCGANPTIRVLQDYEAFCGSSASDKCRTLHLLSKEERISAQEYKKILDEQVPHLLVDVRPPVEVDICHLPHSIYITLDKLERKNLDCLNHLNRKLLEGKQITDEEMSFPVYVICKLGNDSQKAVKILQELSGKELGVLLAKDITGGLMAWSNKVDPTFPQY

>tr|A0A6P7XTB5|A0A6P7XTB5_9AMPH/1546 Adenylyltransferase and sulfurtransferase MOCS3 OS=Microcaecilia unicolor OX=1415580 GN=MOCS3 PE=3 SV=1MFAAVDTKHQDLLIQNLENILNKIKQNKTPLINESFQQFLNTKNGYLVSSLVSEFLEFFSLGFTLAVFQPGTGTVHGPDRGNLAQDLGIVENENTRDGPLLLEVMKRCQNSNSREDGSSDSSLPVLHNLVTELPLLEAKTSLSNEAILRYSRQLVLPELGVKGQLNLSKSSVLIVGCGGLGCPLAQYLAAAGIGRLGLLDYDVVEINNLHRQVLHGENRQGIPKSLSAATALKQLNSGVEYICYNLILTPKIALELIQQYDIIADCSDNVPTRYLVNDACVLTGKPLVSASALRMEGQLTVYNYHGSPCYRCLFPVPPPAETVTNCSDGGVLGVVPGIMGCLQALEVLKIASGLGTSFGQFMLMFDALEGRFRNIRLRPKNQNCAVCGANPTIRVLQDYEAFCGSSASDKCRTLHLLSKEERISAQEYKKILDEQVPHLLVDVRPPVEVDICHLPHSIYITLDKLERKNLDCLNHLNRKLLEGKQITDEEMSFPVYVICKLGNDSQKAVKILQELSGKELGVLLAKDITGGLMAWSNKVDPTFPQY

>tr|A0A6P7XTZ4|A0A6P7XTZ4_9AMPH/1547 Adenylyltransferase and sulfurtransferase MOCS3 OS=Microcaecilia unicolor OX=1415580 GN=MOCS3 PE=3 SV=1MFAAVDTKHQDLLIQNLENILNKIKQNKTPLINESFQQFLNTKNGYLVSSLVSEFLEFFSLGFTLAVFQPGTGTQVHGPDRGNLAQDLGIVENENTRDGPLLLEVMKRCQNSNSREDGSSDSSLPVLHNLVTELPLLEAKTSLSNEAILRYSRQLVLPELGVKGQLNLSKSSVLIVGCGGLGCPLAQYLAAAGIGRLGLLDYDVVEINNLHRQVLHGENRQGIPKSLSAATALKQLNSGVEYICYNLILTPKIALELIQQYDIIADCSDNVPTRYLVNDACVLTGKPLVSASALRMEGQLTVYNYHGSPCYRCLFPVPPPAETVTNCSDGGVLGVVPGIMGCLQALEVLKIASGLGTSFGQFMLMFDALEGRFRNIRLRPKNQNCAVCGANPTIRVLQDYEAFCGSSASDKCRTLHLLSKEERISAQEYKKILDEQVPHLLVDVRPPVEVDICHLPHSIYITLDKLERKNLDCLNHLNRKLLEGKQITDEEMSFPVYVICKLGNDSQKAVKILQELSGKELGVLLAKDITGGLMAWSNKVDPTFPQY

>tr|A0A6P8QB68|A0A6P8QB68_GEOSA/1546 Adenylyltransferase and sulfurtransferase MOCS3 OS=Geotrypetes seraphini OX=260995 GN=LOC117357330 PE=3 SV=1MFAAEDTKSQDLLIQNLENILNKIKQNKTPFVNENFQHFLNTKNGYLVSSLVTEFLQFFDLGCTMAVFQPETGTLHGSDRGNLAHVLGIVENENTRVGPLLLEVMKRCQNFSSRGDGSSDSSLPVLHNLVTELPLLEAKISLSNKAILRYSRQLVLPELGVKGQLNLSKSSVLIVGCGGLGCPLAQYLAAAGIGRLGLLDYDVVEMNNLHRQVLHGENRQGIPKSLSAATTLKQLNSEVEYICYNMTLTSKIALELIQQYDIIADCSDNVPTRYLVNDACVLTGKPLVSASALRMEGQLTIYNYQGNPCYRCLFPVPPPAETVTNCADGGVLGVVPGIMGCLQALEVQKIASGIGTSFGQFILMFDALEGRFRNIRLRAKNQNCAVCGANPTIGVLQDYEAFCGSSASDKCRTVHLLSKEERISAQEYKKILDEKVPHLLVDVRPPVEVDICHLPDSIYIPLDKLEKKNLDCLNHLKRKLLEGRLKTGEEMSFPVYVICKLGNDSQKAVKILQDLSGKELGALLAKDIAGGLMAWSNKVDPTFPQY

>tr|A0A6P8QCL0|A0A6P8QCL0_GEOSA/1545 Adenylyltransferase and sulfurtransferase MOCS3 OS=Geotrypetes seraphini OX=260995 GN=LOC117357330 PE=3 SV=1MFAAEDTKSQDLLIQNLENILNKIKNKTPFVNENFQHFLNTKNGYLVSSLVTEFLQFFDLGCTMAVFQPETGTLHGSDRGNLAHVLGIVENENTRVGPLLLEVMKRCQNFSSRGDGSSDSSLPVLHNLVTELPLLEAKISLSNKAILRYSRQLVLPELGVKGQLNLSKSSVLIVGCGGLGCPLAQYLAAAGIGRLGLLDYDVVEMNNLHRQVLHGENRQGIPKSLSAATTLKQLNSEVEYICYNMTLTSKIALELIQQYDIIADCSDNVPTRYLVNDACVLTGKPLVSASALRMEGQLTIYNYQGNPCYRCLFPVPPPAETVTNCADGGVLGVVPGIMGCLQALEVQKIASGIGTSFGQFILMFDALEGRFRNIRLRAKNQNCAVCGANPTIGVLQDYEAFCGSSASDKCRTVHLLSKEERISAQEYKKILDEKVPHLLVDVRPPVEVDICHLPDSIYIPLDKLEKKNLDCLNHLKRKLLEGRLKTGEEMSFPVYVICKLGNDSQKAVKILQDLSGKELGALLAKDIAGGLMAWSNKVDPTFPQY

>tr|A0A6P8R4N3|A0A6P8R4N3_GEOSA/1482 Adenylyltransferase and sulfurtransferase MOCS3 OS=Geotrypetes seraphini OX=260995 GN=LOC117357330 PE=3 SV=1MAVFQPETGTLHGSDRGNLAHVLGIVENENTRVGPLLLEVMKRCQNFSSRGDGSSDSSLPVLHNLVTELPLLEAKISLSNKAILRYSRQLVLPELGVKGQLNLSKSSVLIVGCGGLGCPLAQYLAAAGIGRLGLLDYDVVEMNNLHRQVLHGENRQGIPKSLSAATTLKQLNSEVEYICYNMTLTSKIALELIQQYDIIADCSDNVPTRYLVNDACVLTGKPLVSASALRMEGQLTIYNYQGNPCYRCLFPVPPPAETVTNCADGGVLGVVPGIMGCLQALEVQKIASGIGTSFGQFILMFDALEGRFRNIRLRAKNQNCAVCGANPTIGVLQDYEAFCGSSASDKCRTVHLLSKEERISAQEYKKILDEKVPHLLVDVRPPVEVDICHLPDSIYIPLDKLEKKNLDCLNHLKRKLLEGRLKTGEEMSFPVYVICKLGNDSQKAVKILQDLSGKELGALLAKDIAGGLMAWSNKVDPTFPQY

>tr|A0A7J7S7X2|A0A7J7S7X2_RHIFE/1458 Adenylyltransferase and sulfurtransferase MOCS3 OS=Rhinolophus ferrumequinum OX=59479 GN=MOCS3 PE=3 SV=1MAAREEVLALQAEVAQREEELSALKQRLAAALLAEQEPERLVPLSPLPPKTSLSRDEILRYSRQLVLPELGVHGQLRLATASVLIVGCGGLGCPLAQYLAAAGVGRLGLVDYDVVEMSNLARQVLHGEALAGQAKVYSAAASLRRLNSAVECVPYAQALTPATALDLVRHYDVVADCSDNVPTRYLVNDACVLAGRPLVSASALRLEGQITVYHYDGGPCYRCVFPQPPPAETVTNCADGGVLGVVTGVVGCLQALEVLKIAAGLGPSYSGSLLLFDAFRGRFRCIQLRSRRPDCAACGERPTVTDLQDYEAFCGSSATDKCLSLQLLSPEERISVTDYKRLLDSGSPHLLLDVRPQVEVDICRLPHALHIPLKHLEQRDAGSLKLLGEAIQKGKQSTQDGVVLPIYVICKLGNDSQKAVKILQSVTAVQELDSRTVQDVVGGLMAWAAKIDGTFPQY

>tr|A0A7J7VZR9|A0A7J7VZR9_MYOMY/1458 Adenylyltransferase and sulfurtransferase MOCS3 OS=Myotis myotis OX=51298 GN=MOCS3 PE=3 SV=1MAAREEVLALQAEVTRREEELSSLKQRLAAALLAEDEPAPRVPVSPLPPKAALSRDEILRYSRQLVLPELGVHGQLRLATASVLVVGCGGLGCPLAQYLAAAGVGRLGLVDYDVVEMSNLARQVLHGEALAGQAKVFSAAASLRRLNSAVECVPYAQALTPATALDLVRRYDVVADCSDNVPTRYLVSDACVLAGRPLVSASALRFEGQITVYHYDGGPCYRCLFPQPPPAETVTSCADGGVLGVVTGILGCLQALEVLKIAAGMGPSYSGSLLLFDALGGHFRCIRLRSRRPDCAACGERPTVTDLQDYEAFCGSSATDKCRSLRLLSPEERVSVSDYKRLLDAGSPHLLLDVRPQVEVDICRLPHALHIPLKHLERRDGKSLKLLGEAIQEGKRGAQEGAALPIYVICKLGNDSQKAVRILQSLTAIPELCSLAVQDVVGGLMAWAAKIDETFPQY

>tr|A0A7J8DJ84|A0A7J8DJ84_ROUAE/1458 Adenylyltransferase and sulfurtransferase MOCS3 OS=Rousettus aegyptiacus OX=9407 GN=MOCS3 PE=3 SV=1MATREEVLALQAEVARREEELGSLKQRLAAALLAEQEPERLVPVSPLPAKAALSRDEILRYSRQLVLPELGVHGQLRLATASVLIVGCGGLGCPLAQYLAAAGVGRLGLVDYDVVEMSNLARQVLHGEALAGQAKVFSAAASLRRLNSAVECVPYAQALTPATALDLVRRYDVVADCSDNVPTRYLVNDACVLAGRPLVSASALRFEGQITVYHYEGGPCYRCIFPQPPPAETVTNCADGGVLGVVTGVLGCLQALEVLKIAAGLGPSYSGSLLLFDALRGRFRCIQLRSRRPDCAACGERPSVTDLQDYEALCGCSATDKCRSLQLLSPEERVSVTDYKRLLDSGTPHLLLDVRPQVEVDICRLPHALHIPLKHLERRDTESLKLLGEAIQKEKQGAHEGVALPIYVICKLGNDSQKAVRILQSLTAAQELDSSTFQDVVGGLMAWATKIDGTFPQY

>tr|A0A7J8HHS7|A0A7J8HHS7_MOLMO/1458 Adenylyltransferase and sulfurtransferase MOCS3 OS=Molossus molossus OX=27622 GN=MOCS3 PE=3 SV=1MAAREEVLALQAEVAQREEELRSLKQRLAAALSAQEEPERLVPVPPLPPKAALSREEILRYSRQLVLPELGVHGQLRLATASVLVVGCGGLGCPLAQYLAAAGVGRLGLVDYDVVEMSNLARQVLHGEALAGQAKVFSAAAALRRLNSAVECVPYAQALTPATALDLVRRYDVVADCSDNAPTRYLVNDACVLAGRPLVSASALRLEGQITVYHYEGGPCYRCVFPQPPPADTVTSCADGGVLGVVTGVLGCLQALEVLKIAAGLGPSYSGSLLLFDALRGRFRCIRLRSRRPDCAACGERPTVTDLQDYEAFCGSSATDKCRSLQLLSPGERVSVTDYKRLLDSGSPHLLLDVRPQVEVDICRLPHALHIPLKQLERRNAESLKLLGEAIQKGKQDTQEGAALPIYVICKLGNDSQKAVKILQSLAEIQELDFLTVKDVVGGLIAWAAKIDGTFPQY

>tr|A0A811XXL2|A0A811XXL2_NYCPR/1469 Adenylyltransferase and sulfurtransferase MOCS3 OS=Nyctereutes procyonoides OX=34880 GN=MOCS3 PE=3 SV=1MPFRTASRSGAMASREEVLALQAEVARREEELSSLKQRLAAALLAEQAPERLVPASPLPPKAALSREEILRYSRQLVLPELGVRGQLRLAAASVLVVGCGGLGCPLAQYLAAAGVGRLGLVDYDVVEMSNLARQVLHGEALAGQAKAFSAAASLRRLNSAVECVPYAQALTPATALGLVRRYDVVADCSDNAPTRYLVSDACVLAGRPLVSASALRWEGQVTVYHYDGGPCYRCVFPQPPPAETVTSCADGGVLGVVTGVLGCLQALEVLKIAAGLGPSYSRSLLIFDALRGQFRCIQLRSRRLDCAACGERPTVTALQDYEAFCGSSATDKCRSLQLLSPEERVSVADYKRLLDSGSPHLLLDVRPQVEVDICRLPHALHVPLKHLERRDAESLKLLGDAIREGKQGAQAGAAFPVYVICKLGNDSQRAVKILQSLTAVQELESLAVQDVVGGLMAWAAKIDGTFPQY

>tr|A0A8C3MIT7|A0A8C3MIT7_GEOPR/1523 Adenylyltransferase and sulfurtransferase MOCS3 OS=Geospiza parvula OX=87175 GN=MOCS3 PE=3 SV=1RLRAEIRRRERELRGLRERLAAELARGDTAEQEEDGERERDEAACGARQPSAAEPARGDAGDAEDEDGARGLRERLAAELARGDSGDADEEDEDDDEEEEEGSLGFPAELPPLPARSALSAAEILRYSRQLVLPELGVRGQLRLARSSVLVVGCGGLGCPLAQYLAAAGVGRLGLVDHDVVETSNLQRQVLHGEARRGRPKARSAAAALRRLNSAVQYVPYRGALRPRSALRLVRQYDIVADCSDNVPTRYLVSDACVLAGKPLVSGSALRLEGQLAVYNYRGGPCYRCLFPRPPPPDTVTNCADGGVLGAVTGIIGCLQALEVLKIASGMGSSLSGYMLMFDALEGRFRNIKLRPRKADCAVCGDSPSVTCLQDYEAFCGSCATDKCRALQLLPGADRVSVQQYKALLDARVPHVLLDVRPPVEAEICRLPHALHIPLRSLQDRDQESLQRLQNRIGEEKQRTDDQTPLPVYVVCKLGNDSQKAVKILQELPAEEFGSVVAKDIKGGLMAWATKIDSTFPQY

>tr|H0XRE9|H0XRE9_OTOGA/1460 Adenylyltransferase and sulfurtransferase MOCS3 OS=Otolemur garnettii OX=30611 GN=MOCS3 PE=3 SV=1MAAREKVLALQAEVAQREEELSFLKQKLAAALLAEQEPPLEHLDPLSPLPPKAALSRDEILRYSRQLVLPELGVHGQLRLATASVLIVGCGGLGCPLAQYLAAAGVGRLGLVDYDVVEMSNLARQVLHGEALAGQAKAFSAAASLRRLNSAVECVPYAQALTPATALDLVRRYDVVADCSDNVPTRYLVNDACVLAGRPLVSASALRFEGQITVYHYDGGPCYRCVFPQPPPAETVTNCADGGVLGVVTGVLGCLQALEVLKIAAGLGPSYSGRMLLFDALRGHFRCIQLRIRRPDCAACGERPTVTDLQDYEAFCGSSATDKCRSLQLLSSEERVSVTDYKRLLDSGAPHLLLDVRPQVEVDICCLPHALHIPLKHLERRDAASLKLLGEAIGEGKRDTQPGAAFSIYVICKLGNDSQKAVKILQSLTEAHELDSLTVRDVVGGLMAWAAKIDRTFPQY

>tr|M3Z5N2|M3Z5N2_MUSPF/1458 Adenylyltransferase and sulfurtransferase MOCS3 OS=Mustela putorius furo OX=9669 GN=MOCS3 PE=3 SV=1MGSSEEVLALQAEVARREEELSSLKQRLAAALLAEQEPERLVPVSPLPPKAALSRDEILRYSRQLVLPELGVHGQLRLATASVLIVGCGGLGCPLAQYLAAAGVGRLGLVDYDVVEASNLARQVLHGEALAGQAKVFSAAASLRRLNSAVECVPYAQALTAATALDLIRRYDVVADCSDNVPTRYLVNDACVLAGRPLVSASALRFEGQITVYHYDGGPCYRCIFPQPPPAETVTSCADGGVLGVVTGVLGCLQALEVLKIVAGLGPSYSRSLLIFDALRGQFRCIRLRSRRPDCAACGERPTVTDLEDYEAFCGSSATEKCRSLHLLSPEERVSVTDYKRLLDSGSPHLLLDVRPQVEVDICRLPHALHIPLKHLERRDAESLELLGEAIREGKQGTQEGAAFPVYVICKLGNDSQKAVKVLQSLTAVQELESSTIQDVVGGLMAWAAKIDGTFPQY

>tr|A0A3Q7W3H0|A0A3Q7W3H0_URSAR/1458 Adenylyltransferase and sulfurtransferase MOCS3 OS=Ursus arctos horribilis OX=116960 GN=MOCS3 PE=3 SV=2MASREEVLALQAEVVRREEELSSLKQRLAAAVLAEQEPERLVPVSPLPPKAALSRDEILRYSRQLVLPELGVHGQLRLATASVLVVGCGGLGCPLAQYLAAAGVGRLGLVDYDVVEMSNLARQVLHGEALAGQAKVFSAAASLRRLNSAVECVPYAQVLTPATALDLVRRYDVVADCSDNVPTRYLVNDACVLAGRPLVSASALRFEGQITVYHYDGGPCYRCIFPQPPPAETVTNCADGGVLGVVTGVLGCLQALEVLKIAAGLGPSYSRSLLIFDALRGQFRCIQLRSRRPDCAACGERPTVTDLQDYEAFCGSSATDKCRSLQLLSPEERVSVTDYKRLLDSGSPHLLLDVRPQVEVDICRLPHALHIPLKHLERRDVQSLELLGEAIREGRQGTQEGAAFPVYVICKLGNDSQKAVKILQSLTAVQDLESLTVQDVVGGLMAWAAKVDGTFPQY

>tr|A0A8B7R642|A0A8B7R642_HIPAR/1458 Adenylyltransferase and sulfurtransferase MOCS3 OS=Hipposideros armiger OX=186990 GN=MOCS3 PE=3 SV=1MAAREEVLALQAEVARREEELSSLKQRLAAALLAEQEPERLLPVPPLPPKASLSRDEILRYSRQLVLPELGVRGQLRLATASVLIVGCGGLGCPLAQYLAAAGVGRLGLVDYDVVEVSNLARQVLHGEALAGQAKVYSAAASLRRLNSAVECVPYAQALTPATALELVRHYDVVADCSDNVPTRYLVNDACVLAGRPLVSASALRLEGQMTVYHYDGGPCYRCVFPRPPPAETVTNCADGGVLGVVTGVLGCLQALEVLKIAAGLGPSYSGSLLLFDALRGRFRSIQLRSRRTDCAACGERPTVTDLQDYEAFCGSSATDKCRSLQLLSPEERISVTDYKRLLDSGSPHLLLDVRPQVEVDICRLPHALHIPLKRLEQRDAGSLKLLGEAIQKGKRGTQEGTALPIYVICKLGNDSQKAAKILQSLTAVQELDSSAVQDVVGGLMAWAAKIDGTFPQY

>tr|A0A8C0L179|A0A8C0L179_CANLU/1458 Adenylyltransferase and sulfurtransferase MOCS3 OS=Canis lupus dingo OX=286419 GN=MOCS3 PE=3 SV=1MASREEVLALQAEVARREEELSSLKQRLAAALLAEQAPERRVPVSPLPPKAALSREEILRYSRQLVLPELGVHGQLRLAAASVLVVGCGGLGCPLAQYLAAAGVGRLGLVDYDVVEMSNLARQVLHGEALAGQAKAFSAAASLRRLNSAVECVPYAQALTPATALDLVRRYDVVADCSDNVPTRYLVNDACVLAGRPLVSASALRFEGQITVYHYDGGPCYRCVFPQPPPAETVTNCADGGVLGVVTGVLGCLQALEVLKVAAGLGPSYSRSLLIFDALRGQFRCIQLRSRRLDCAACGERPTVTALQDYEAFCGSSATDKCRSLQLLSPEERVSVADYKRLLDSGSPHLLLDVRPQVEVDICRLPHALHIPLKHLERRNAESLKLLGEAIREGKQGAQAGAAFPVYVICKLGNDSQKAVKILQSLTAVQELESLTVQDVVGGLMAWAAKIDGTFPQY

>tr|A0A8C0PEZ7|A0A8C0PEZ7_CANLF/1498 Adenylyltransferase and sulfurtransferase MOCS3 OS=Canis lupus familiaris OX=9615 GN=MOCS3 PE=3 SV=1MSTYHFPPQTPWPRFSLFQSLGDGKPEPEVPFRTTSRSGAMASREEVLALQAEVARREEELSSLKQRLAAALLAEQAPERRVPVSPLPPKAALSREEILRYSRQLVLPELGVHGQLRLAAASVLVVGCGGLGCPLAQYLAAAGVGRLGLVDYDVVEMSNLARQVLHGEALAGQAKAFSAAASLRRLNSAVECVPYAQALTPATALDLVRRYDVVADCSDNVPTRYLVNDACVLAGRPLVSASALRFEGQITVYHYDGGPCYRCVFPQPPPAETVTNCADGGVLGVVTGVLGCLQALEVLKIAAGLGPSYSRSLLIFDALRGQFRCIQLRSRRLDCAACGERPTVTALQDYEAFCGSSATDKCRSLQLLSPEERVSVADYKRLLDSGSPHLLLDVRPQVEVDICRLPHALHIPLKHLERRNAESLKLLGEAIREGKQGAQAGAAFPVYVICKLGNDSQKAVKILQSLTAVQELESLTVQDVVGGLMAWAAKIDGTFPQY

>tr|A0A8C0SDA6|A0A8C0SDA6_CANLF/1498 Adenylyltransferase and sulfurtransferase MOCS3 OS=Canis lupus familiaris OX=9615 GN=MOCS3 PE=3 SV=1MSTYHFPPQTPWPRFSLFQSLGDGKPEPEVPFRTTSGSGAMASREEVLALQAEVARREEELSSLKQRLAAALLAEQAPERRVPVSPLPPKAALSREEILRYSRQLVLPELGVHGQLRLAAASVLVVGCGGLGCPLAQYLAAAGVGRLGLVDYDVVEMSNLARQVLHGEALAGQAKAFSAAASLRRLNSAVECVPYAQALTPATALDLVRRYDVVADCSDNVPTRYLVNDACVLAGRPLVSASALRFEGQITVYHYDGGPCYRCVFPQPPPAETVTNCADGGVLGVVTGVLGCLQALEVLKIAAGLGPSYSRSLLIFDALRGQFRCIQLRSRRLDCAACGERPTVTALQDYEAFCGSSATDKCRSLQLLSPEERVSVADYKRLLDSGSPHLLLDVRPQVEVDICRLPHALHIPLKHLERRNAESLKLLGEAIREGKPGAQAGAAFPVYVICKLGNDSQKAVKILQSLTAVQELESLTVQDVVGGLMAWAAKIDGTFPQY

>tr|A0A8C3D929|A0A8C3D929_9CORV/1532 Adenylyltransferase and sulfurtransferase MOCS3 OS=Corvus moneduloides OX=1196302 GN=MOCS3 PE=3 SV=1MAGAAARLRAEIRRRERELRGLRERLAAELARGDTAEQQEDREREQQEEGACGLRQPSPAELARGDAGDAEDEDGARGMRERLAAELARGDSGDADEEDEDDDEEEEETEGALGFPAELPPLPARSALSAAEILRYSRQLVLPELGVRGQLRLARSSVLVVGCGGLGCPLAQYLAAAGVGRLGLVDHDVVETSNLQRQVLHGEARRGRPKARSAAAALRRLNSAVQYVPYRGALRPRSALRLVRQYDIVADCSDNVPTRYLVSDACVLAGRPLVSGSALRLEGQLVVYNYRGGPCYRCLFPRPPPPDTVTNCADGGVLGVVTGIIGCIQALEVLKIASGMGSSFSQSMLMFDALEGRFRNIKLRPKKADCAVCGDNPSITCLQDYEAFCGSSATDKCRTLQLLPSQDRISVHQYKELLDEQVPHVLLDVRPQVEVDICRLEHAVHIPLSKLEEKNEESMQYLQKRICEEKQRTDDQTSLPVYVVCKLGNDSQKAVKILQELPAKEFGSVLAKDIKGGLMAWATKIDSTFPQY

>tr|A0A8C3VKJ6|A0A8C3VKJ6_CATUS/1438 Adenylyltransferase and sulfurtransferase MOCS3 OS=Catharus ustulatus OX=91951 GN=MOCS3 PE=3 SV=1GRSRSVPVPVAEGAHQQPHDERQVLPPLPARAALSAAEILRYSRQLVLPELGVRGQLRLARSSVLVVGCGGLGCPLAQYLAAAGVGRLGLVDHDVVETSNLQRQVLHGEARCGRPKARSAAAALRRLNSAVQYVPYRGALRPRSALRLVRQYDIVADCSDNVPTRYLLSDACVLAGKPLVSGSALRLEGQLVVYNHGGGPCYRCLFPQPPPPDTVTNCADGGVLGVVTGIIGCLQALEVLKIASGMGCSLHRYMLMFDALEGRFRHIKLRPRKAECAVCGDSPSITCLQDYEAFCGSCATDKCRAVQLLPAAARISVQQYKQLLDAAVPHVLLDVRPQVEADICRLQHALHIPLRQLQDRDQQALQLLRERIAEEKRRTDGQTPVPVYVVCKLGNDSQKAVKVLQELPAEELGSVLAKDIKGGLMAWATKIDSTFPQY

>tr|A0A8C3W1P1|A0A8C3W1P1_9CETA/1455 Adenylyltransferase and sulfurtransferase MOCS3 OS=Catagonus wagneri OX=51154 GN=MOCS3 PE=3 SV=1MAAREEVLALQAEVARREDELSSLKQRLAAAILAEQESERLLPVSPLPPKAALSPDEILRYSRQLVLPELGVQGQLRLATASVLVVGCGGLGCPLAQYLAAAGVGRLGLVDYDVVEVSNLARQVLHGEALAGQAKVFSAAASLRRLNSAVECVPYAQALTPATALDLVRRYDVVADCSDNVPTRYLVNDACVLAGRPLVSASALRFEGQITVYHYGGGPCYRCVFPQPPPVETVTNCADGGVLGVVTGVLGCLQALEVLKIAAGLGPSYSGSLLLFDALRGLFRCIQLRRRRPDCAACGERPTVTELQDYEGFCGSSATDKCRSLQLLSREERVSVIDYKQILDSGSPHLLLDVRPQVEVDICHLPHALHIPLKHLERRDAESLKLLGEAIREGKQGAPEGASLPIYVICKLGNDSQKAVKMLQSLTDLESLTVKDVVGGLMAWAAKVDGTFPQY

>tr|A0A8C4PVK2|A0A8C4PVK2_EQUAS/1458 Adenylyltransferase and sulfurtransferase MOCS3 OS=Equus asinus asinus OX=83772 GN=MOCS3 PE=3 SV=1MATREEVLALQAEVAQREEELSCLKQRLAAALLAEQKPERRVPVSPLPPKAALSRDEILRYSRQLVLPELGVHGQLRLATASVLVVGCGGLGCPLAQYLAAAGVGRLGLVDYDVVEMSNLARQVLHGESLAGQAKVLSAAAALRRLNSAVECVPYAQALTPATALDLVRRYDVVADCSDNVPTRYLVNDACVLAGRPLVSASALRFEGQITVYHYHGGPCYRCVFPQPPPAETVTNCADGGVLGIVTGVVGCLQALEVLKIAAELGPSYSGSLLLFDALRGHFRCIKLRSRRPDCAACGERPTVTDLQDYEAFCGSSATDKCRSLQLLSPEERVSVTDYKRLLDSGAPHLLLDVRPQVEVDICRLPHALHIPLKHLQRRDAESLKLLGEAIRKGKQGTQEGAALPIYVICKLGNDSQKAVKILQSLTAVQELESLTVQDVVGGLMAWAARIDETFPQY

>tr|A0A8C7EMK1|A0A8C7EMK1_NEOVI/1458 Adenylyltransferase and sulfurtransferase MOCS3 OS=Neovison vison OX=452646 GN=MOCS3 PE=3 SV=1MGSREEVLALEAEVARREEELSSLKQRLASALLAEQEPERLPPVSPLPPKAALSRDEILRYSRQLVLPELGVHGQLRLATASVLIVGCGGLGCPLAQYLAAAGVGRLGLVDYDVVEVSNLARQVLHGEALAGQAKVFSAAASLRRLNSAVECVPYAQALTAATALDLIRRYDVVADCSDNVPTRYLVNDACVLAGRPLVSASALRFEGQITVYHYDGGPCYRCVFPQPPPAETVTSCADGGVLGVVTGVLGCLQALEVLKIVAGLGPSYSRSLLIFDALRGQFRCIRLRSRRPDCAACGERPTVADLRDYEAFCGSSATEKCRSLHLLSPEERVSVTDYKRLLDSGSPHLLLDVRPQVEVDICRLPHALHIPLKHLERRDAESLELLGEAIREGKQGTQEGAAFPVYVICKLGNDSQKAVKVLQSLTAVQELESSTIQDVVGGLMAWAAKIDGTFPQY

>tr|A0A8C8WI93|A0A8C8WI93_PANLE/1458 Adenylyltransferase and sulfurtransferase MOCS3 OS=Panthera leo OX=9689 GN=MOCS3 PE=3 SV=1MASREEVLALQAQVAQREEELSFLKQKLAAAVLAEQEPERLVSVSPLPPKASLSRDEILRYSRQLVLPELGVQGQLRLAAASVLVVGCGGLGCPLAQYLAAAGVGRLGLVDYDVVEMSNLARQVLHGEALAGQAKVFSAAASLRRLNSAVECVPYAEALTPATALDLVRRYDVVADCSDNVPTRYLVNDACVLAGRPLVSASALRFEGQITVYHYDGGPCYRCVFPQPPPAETVTNCADGGVLGVVTGVLGCLQALEVLKIAAGLGPSYSGSLLIFDALRGHFRCIQLRGRRPDCAACGERPTVTALEDYEAFCGSSATDKCRSLRLLSPEERVSVTDYKRLLDSGSPHLLLDVRPQVEVDICRLPHALHIPLKRLERRDAESLELLGEAIREGKQGTQEGAAFPVYVICKLGNDSQKAVKILQSLTAVQELESFTVQDVVGGLMAWAAKIDGTFPQY

>tr|A0A8C9NQA2|A0A8C9NQA2_SERCA/1418 Adenylyltransferase and sulfurtransferase MOCS3 OS=Serinus canaria OX=9135 GN=MOCS3 PE=3 SV=1FPAELPPLPARSALSAAEILRYSRQLVLPELGVRGQLRLARSSVLVVGCGGLGCPLAQYLAAAGVGRLGLVDHDVVETSNLQRQVLHGEARRGRPKARSAAAALRRLNSAVQYVPYRGALRPRSALRLVRQYDIVADCSDNVPTRYLVSDACVLAGKPLVSGSALRLEGQLAVYNYRGGPCYRCLFPEPPPPDTVTNCADGGVLGVVTGIIGCMQALEVLKIASGMGSSFGGYMLMFDALEGRFRNIKLRPRKADCAVCGDNPSVTCLQDYEAFCGSSATDKCRALRLLPSGDRVSVQQYKELLDARVPHVLLDVRPQVEAEICRLQHALHIPLRSLEEKDEQALQRLQKRICEEKQRTDDQTPLPVYVVCKLGNDSQKAVKILQELPAEEFGSVLAKDIKGGLMAWATKIDSTFPQY

>tr|A0A8D2ADA0|A0A8D2ADA0_SCIVU/1460 Adenylyltransferase and sulfurtransferase MOCS3 OS=Sciurus vulgaris OX=55149 GN=MOCS3 PE=3 SV=1MAAREQVLALQAEVAQREEELDSLKQRLATALLAEQESEPERLVPASPLPPKAALSRDEILRYSRQLVLPELGVQGQLRLATASVLIVGCGGLGCPLAQYLAAAGVGRLGLVDYDVVEMSNLARQVLHGEALAGQAKAFSAAASLRRLNSAVECVPYAQALTPATALDLVRRYDVVADCSDNVPTRYLVNDACVLAGRPLVSASALRLEGQITVYHYDGGPCYRCVFPQPPPAETVTNCADGGVLGVVTGVLGCLQALEVLKIAAGLGPSYSGSLLLFDALRGHFRCIRLRRRRPDCAACGECPTVTDLQDYEAFCGSSATDKCRSLQLLGPEERVSVTDYKRLLDSGAPHLLLDVRPQVEVDICRLPHALHIPLRHLEQRDAESLKLLGEAIQEGKRAIQEGAVLPIYVICKLGNDSQKAVRILQSLTAARESDSFMVQDVVGGLMAWAAKVDGTFPQY

>tr|A0A6G1AFB9|A0A6G1AFB9_CROCR/1469 MOCS3 sulfurtransferase (Fragment) OS=Crocuta crocuta OX=9678 GN=Mocs3 PE=3 SV=1CLSRQLPGGSAMASREEVLALRAQVAEREEELSFLKQKLAAATMAEQEPQRLVSVSPLPPKASLSRDEILRYSRQLVLPELGVQGQLRLAAASVLVVGCGGLGCPLAQYLAAAGVGRLGLVDYDVVEMSNLARQVLHGEALTGQAKVFSAAASLRRLNSAVECVPYAEALTPATALDLVRRYDVVADCSDNVPTRYLVNDACVLAGRPLVSASALRFEGQITVYHYGGGPCYRCVFPQPPPAETVTNCVDGGVLGVVTGVLGCLQALEVLKIAAGLGPSYSGSLLIFDALKGRFRCIQLRGRRPDCAACGERPTVTALQDYEAFCGSSATDKCRGLQLLSPEERVSVTDYKRLLDSGSPHLLLDVRPQVEVDICRLSHALHIPLKHLERRDAESLKLLREAIREGKQGPQEGAAFPVYAICKLGNDSQRAVKILQSLMAVQELELVTVQDVVGGLMAWAAKIDGTFPQY

>tr|A0A7K4VN16|A0A7K4VN16_9EMBE/1475 MOCS3 sulfurtransferase (Fragment) OS=Emberiza fucata OX=337179 GN=Mocs3 PE=3 SV=1QPSAAEPARGDAGHAEDEDGARGLRERLAAELARGDSGDADEEDEDDDEDEEEGSLGFPAELPPLPARSALSAAEILRYSRQLVLPELGVRGQLRLARSSVLVVGCGGLGCPLAQYLAAAGVGRLGLVDHDVVETSNLQRQVLHGEARRGRPKARSAAAALRRLNSAVQYVPYRGALRPRSALRLVRQYDIVADCSDNAPTRYLVSDACVLAGKPLVSGSALRLEGQLAVYNYGGGPCYRCLFPRPPPPDTVTNCADGGVLGAVTGIIGCLQALEVLKIASGMGSSFSGYMLMFDALEGRFRNIKLRPRRADCAVCGDSPSITCLQDYEAFCGSCATDKCRALQLLPGADRVSAQQYKALLDARVPHVLLDVRPPVEAEICRLPHALHVPLRCLQDGDRESLRRLQKRICEEKQRTDGQTPLPVYVVCKLGNDSQKAVKILQELPAEEFGSVVAKDIKGGLMAWATKIDSTFPQY

>tr|A0A7K4W1M8|A0A7K4W1M8_9TYRA/1465 MOCS3 sulfurtransferase (Fragment) OS=Tachuris rubrigastra OX=495162 GN=Mocs3 PE=3 SV=1AAEVALGDAEEEDGDGGLRDLLARGDAGSAEEDNDDDDDDDAEGAGAFPAELPPLPPRAALSAAEIARYSRQLVLPELGVRGQLRLARSAVLVVGCGGLGCPLAQYLAAAGVGRLGLLDHDVVETSNLQRQVLHGEARRGRPKAASAAAALRRLNSAVQYVPYCGALSPRSALRIVRQYDIVADCSDNAPTRYLVNDACVLAGKPLVSGSALRLEGQLVVYNYRGGPCYRCLFPEPPPPDAVTSCADGGVLGVVPGIVGCIQALEVLKIASGMGSSFNQFMLMFDALEGRFRNIKLRPKKPDCAVCGDNPTVTCLQDYEAFCGSSATDKCRTLHLLPSKDRISVEQYKTVLDAKVPHVLLDVRPQVEVDICRLEHAVHIPLSKLEEKDEEQLEYLEKRICEEKQRTKDQASLPVYVVCKLGNDSQKAVKILQELPAKESGSILAKDIKGGLMAWATKIDPTFPQY

>tr|A0A7K5ZVZ6|A0A7K5ZVZ6_ONYCO/1465 MOCS3 sulfurtransferase (Fragment) OS=Onychorhynchus coronatus OX=360224 GN=Mocs3 PE=3 SV=1AAEVALGDAEEEEEEDGDGGLRDLLARGDAGSAEDDDDDDDAEGSDAFPAELPPLPPRAALSAAEIARYSRQLVLPELGVRGQLRLARSAVLVVGCGGLGCPLAQYLAAAGVGRLGLLDHDVVETSNLQRQVLHGEARRGRPKAASAAAALRRLNSAVQYVPYCGALSPRSALRLVRQYDIVADCSDNAPTRYLVNDACVLAGKPLVSGSALRLEGQLVVYNYRGGPCYRCLFPEPPPPDTVTSCADGGVLGVVPGIIGCIQALEVLKIASGMGSSFNQFMLMFDALEGRFRNIKLRPKKPDCAVCGDNPTITCLQDYEAFCGSSATDKCRTLHLLPSKDRISVEQYKKVLDDKVPHVLLDVRPQVEVDICCLEHAVHIPLSKLEEKDEEQLEYLEKRICEEKQRTNDQASLPVYVVCKLGNDSQKAVKILQELPGKESGSVLAKDIKGGLMAWATKIDPTFPQY

>tr|A0A7K6JTR5|A0A7K6JTR5_9PASE/1454 MOCS3 sulfurtransferase (Fragment) OS=Oreocharis arfaki OX=979223 GN=Mocs3 PE=3 SV=1RGMRERLAAELARGDSGDADEEDEDDEDEEDEGALGFPAELPPLPARSALSAAEILRYSRQLVLPELGVRGQLRLARSSVLVVGCGGLGCPLAQYRAAAGVGRLGLVDHDVVETSNLQRQVLHGEARRGRPKARSAAAALRRLNSAVQYVPYRGALGPRSALRLVRQYDIVADCSDNVPTRYLVSDACVLAGKPLVSGSALRLEGQLVVYNYRGGPCYRCLFPRPPPPDTVTNCADGGVLGVVTGIIGCIQALEVLKIASGMGSSFSQSMLMFDALEGRFRNIKLRPKKPDCAVCGDSPSVTCLQDYEAFCGSSATDKCRTLQLLPGKDRISVQQYKELLDERVPHVLLDVRPQVEVDICRLEHAVHIPLSKLQEKDGESLQYLQRRICEERQRTDDQTSLPVYVVCKLGNDSQKAVKILQELPAKEFGSVLAKDIKGGLMAWATKIDSTFPQY

>tr|A0A7K6L061|A0A7K6L061_9CORV/1472 MOCS3 sulfurtransferase (Fragment) OS=Falcunculus frontatus OX=254539 GN=Mocs3 PE=3 SV=1AAELARGDARDAEDEDGARGMRERPAAELAREDSGDADEEDEDDDEEEEEGALGFPAELPPLPVRSALSAAEILRYSRQLLLPELGVRGQLRLARSSVLVVGCGGLGCPLAQYLAAAGVGRLGLVDHDVVETSNLQRQVLHGEARRGRPKARSAAAALRRLNSAVQYVPYRGALGPRSALRLVRQYDIVADCSDNVPTRYLVSDACVLAGKPLVSGSALRLEGQLVVYNYRGGPCYRCLFPQPPPPDTVTNCADGGVLGVVTGIIGCIQALEVLKIASGMGCSFNQYMLMFDALEGRFRNIKLRPRKPDCAVCGDKPSITCLQDYEAFCGSSATDKCRTLQLLPGEDRISVQQYKELLDEQVPHVLLDVRPQVEVDICRLEHALHIPLSKLEEKDEESLQYLQKRIREEKQRTDDQTSLPVYVVCKLGNDSQKAVKILQELPAEEFGSVLAKDIKGGLMAWATKIDSTFPQY

>tr|A0A7K7C357|A0A7K7C357_APHCE/1481 MOCS3 sulfurtransferase (Fragment) OS=Aphelocoma coerulescens OX=39617 GN=Mocs3 PE=3 SV=1QPSAAELARRDAGDAEDEDGARGMRERLAAELARGDSGDADEEGEDDDDDDEEKEMEMEGALGFPAELPPLPARSALSAAEILRYSRQLVLPELGVRGQLRLARSSVLVVGCGGLGCPLAQYLAAAGVGRLGLVDHDVVETSNLQRQVLHGEARCGRPKARSAAAALRRLNSAVQCVPYRGALRPRSALRLVRQYDIVADCSDNVPTRYLVSDACVLAGRPLVSGSALRLEGQLAVYNYRGGPCYRCLFPRPPPPDTVTNCADGGVLGAVTGIIGCIQALEVLKIASGMGSSFNQYMLMFDALEGRFRNIKLRPKKADCAVCGDNPSITCLQDYEAFCGSSATDKCRTLQLLPSQDRISVRQYKELLDEQVPHVLLDVRPQVEVDICRLEHAVHIPLSKLEEKNEESLQYLQKRICEEKRRTDDQTSLPVYVVCKLGNDSQKAVKILQELPAKEFGSVLAKDIKGGLMAWATKIDSTFPQY

>tr|A0A7K7DL19|A0A7K7DL19_PHEME/1475 MOCS3 sulfurtransferase (Fragment) OS=Pheucticus melanocephalus OX=371919 GN=Mocs3 PE=3 SV=1QPSAAEPARGDAGDAEDEDGARGLRERLAAELARGDSGDADEEDEDDDDEEEEGSLGFPAELPPLPARSALSAAEILRYSRQLVLPELGVRGQLRLARSSVLVVGCGGLGCPLAQYLAAAGVGRLGLVDHDVVETSNLQRQVLHGEARRGRPKARSAAAALRRLNSAVQYVPYRGALRPRSALRLVRQYDIVADCSDNAPTRYLVSDACVLAGKPLVSGSALRLEGQLAVYNYRGGPCYRCLFPRPPPPDTVTNCADGGVLGAVTGIIGCLQALEVLKIASGMGSSFSGYMLMFDALEGRFRNIKLRPRRADCAVCGDSPSVTCLQDYEAFCGSCATDKCRALRLLPGADRVSVQQYKALLDARVPHVLLDVRPQVEAEICRLPHALHIPLRSLQDGHRESLQRLQKRICEEKQRTDDQTPLPVYVVCKLGNDSQKAVKILQELPGEEFGSVVAKDIKGGLMAWATKIDSTFPQY

>tr|A0A7K7GQY8|A0A7K7GQY8_ERIRU/1475 MOCS3 sulfurtransferase (Fragment) OS=Erithacus rubecula OX=37610 GN=Mocs3 PE=3 SV=1QPPAAELARGDAAHAGDEDGARGLRERLAAELARGDSGDADEEDEDDDEEEQEGAPGFPAELPPLPARAALSAAEILRYSRQLVLPELGVRGQLRLARSSVLVVGCGGLGCPLAQYLAAAGVGRLGLVDHDVVETSNLQRQVLHGEARCGRPKARSAAAALRRLNSAVQYVPYRGALRPRSALRLVRQYDIVADCSDNVPTRYLVSDACVLAGKPLVSGSALRLEGQLVVYNHGGGPCYRCLFPEPPPPDTVTNCADGGVLGVVTGIIGCLQALEVLKIASGMGSSFHRYMLMFDALEGRFRNIKLRPRKADCAVCGERPSITCLQDYEAFCGSSATDKCRAVRLLPGAHRVSVQQYKQLLDGRVPHLLLDVRPQVEADICRLQHALHIPLRRLQERDPPALQLLRRRIGEERRRADGQAALPVYVVCKLGNDSQKAVKILQELPDEEFGALVAKDIKGGLMAWATKIDSTFPQY

>tr|A0A7K7IR02|A0A7K7IR02_LOXCU/1475 MOCS3 sulfurtransferase (Fragment) OS=Loxia curvirostra OX=64802 GN=Mocs3 PE=3 SV=1QPSAAEPARGDAGDAEDEDGARGLRERLAAELARGDSGDADEEDEDDEEEEEEGALGFPAELPPLPARSALSAAEILRYSRQLVLPELGVRGQLRLARSSVLVVGCGGLGCPLAQYLAAAGVGRLGLVDHDVVETSNLQRQVLHGEARRGRPKARSAAAALRRLNSAVQYVPYRGALRPRSALRLVRQYDIVADCSDNVPTRYLVSDACVLAGKPLVSGSALRLEGQLAVYNYRGGPCYRCLFPEPPPPDTVTNCADGGVLGVVTGIIGCMQALEVLKIASGMGSSFGGYMLMFDALEGRFRNIKLRPRKADCAVCGDNPSVTCLQDYEAFCGSSATDKCRALRLLPSGDRVSVQQYKELLDARVPHVLLDVRPQVEAEICRLQHALHIPLRSLEEKDEQALQRLQKRICEEKQRTDDQTPLPVYVVCKLGNDSQKAVKILQELPAEEFGSVLAKDIKGGLMAWATKIDSTFPQY

>tr|A0A7K7KA84|A0A7K7KA84_AGEPH/1475 MOCS3 sulfurtransferase (Fragment) OS=Agelaius phoeniceus OX=39638 GN=Mocs3 PE=3 SV=1QPPAAEPARGDAGDAEDEDGARGLRERLAAELARGDSGDADEEDEDDEDEDEDGALGFPAELPPLPARSALSAAEILRYSRQLVLPELGVRGQLLLARSSVLVVGCGGLGCPLAQYLAAAGVGRLGLVDHDVVETSNLQRQVLHGEARRGRPKARSAAAALRRLNSAVQYVPYRGALRPRSALRLVRQYDIVADCSDNVPTRYLVSDACVLAGKPLVSGSALRLEGQLAVYNHRGGPCYRCLFPRPPPPDTVTNCADGGVLGAVTGIIGCLQALEVLKIASGMGSSFSGYMLMFDALEGRFRNIKLRPRRADCAVCGDSPSITCLQDYEAFCGSCATDKCRALQLLPGADRVSVQQYKALLDARAPHVLLDVRPPVEAEICRLPHALHIPLRRLQDRDPASLQRLQQRICEEKQRSDDQTPLPVYVVCKLGNDSQKAVKILQELPAEEFGSVLAKDIKGGLMAWATKIDSTFPQY

>tr|A0A7K7M2M0|A0A7K7M2M0_9PASS/1474 MOCS3 sulfurtransferase (Fragment) OS=Brachypodius atriceps OX=182895 GN=Mocs3 PE=3 SV=1QPSAAELARGDAGDAEDEDGARGLRERLAAELAREDSGDADEEDDDEEEEEEGTLGFPAELPPLPARSALSAAEILRYSRQLVLPELGVRGQLRLARSSVLVVGCGGLGCPLAQYLAAAGVGRLGLVDHDVVETSNLQRQVLHGEARRGRPKARSAAAALRRLNSAVQYVPYRGQLRPRSALRLVRQYDIVADCSDNVPTRYLVSDACVLAGKPLVSGSALRLEGQLAVYNYGGGPCYRCLFPEPPPPDTVTNCADGGVLGVVTGIIGCMQALEVLKIASGMGSSFNRYMLMFDALEGRFRNIKLRPRKADCAVCGDNPSVTCLQDYEAFCGSSATDKCRSLQLLPSGDRISVQRYKELLDERVPHVLLDVRPQVEVEICRLEHALHIPLRKLEEKDEESLQHLQKRICEEKQRTDDQTSVPVYVVCKLGNDSQKAVKILQELSAKEIGSVLAKDIRGGLMAWAAKIDSTFPQY

>tr|A0A7K7PPW8|A0A7K7PPW8_ACRAR/1469 MOCS3 sulfurtransferase (Fragment) OS=Acrocephalus arundinaceus OX=39621 GN=Mocs3 PE=3 SV=1AAEPARGDAGDAEDEDGARGLRERLAAELARGDSGDADEEDDDEEEEGTLGFPAELPPLPARSALSAAEILRYSRQLVLPELGVRGQLRLARSSVLVVGCGGLGCPLAQYLAAAGVGRLGLVDHDVVETSNLQRQVLHGEARRGRPKARSAAAALRRLNSAVQYVPYRGALRARSALRLVRQYDIVADCSDNVPTRYLVSDACVLAGKPLVSGSALRLEGQLVVYNYRGGPCYRCLFPEPPPPDTVTNCADGGVLGVVTGIIGCMQALEVLKIASGMGSSFNQYMLMFDALEGRFRNIKLRPRKADCAVCGDNPSVTCLQDYEAFCGSSATDKCRSVQLLPSGDRISVQQYKELLDRRVPHVLLDVRPQVEVDICRLEHAVHIPLRKLEEKDEESLQHLQKRICEEKQRTDDQTSVPVYVVCKLGNDSQKAVKILQELPASQFGSVLAKDIKGGLMAWATKIDSTFPQY

>tr|A0A7K7QV05|A0A7K7QV05_POEAT/1427 MOCS3 sulfurtransferase (Fragment) OS=Poecile atricapillus OX=48891 GN=Mocs3 PE=3 SV=1AEEGEGALGFPAELPPLPARSALSAAEILRYSRQLVLPELGVRGQLRLARSSVLVVGCGGLGCPLAQYLAAAGVGRLGLVDHDVVETSNLQRQVLHGEARRGRPKARSAAAALRRLNSAVQYVPYRGTLRPRSALSLVRQYDIVADCSDNVPTRYLVSDACVLAGKPLVSGSALRLEGQLAVYNHGGGPCYRCLFPEPPPPDAVTNCADGGVLGVVTGIIGCMQALEVLKIASGMGSSFSRCMLMFDALEGRFRNIKLRPRRADCAACGDSPSITCLQDYEAFCGSSATDKCRSLRLLPSGDRISVQRYKELLDARLPHLLLDVRPQVEVEICRLEHALHIPLRKLQEKDEESLRRLQKRISEEKQRTDDQTSFPVYVVCKLGNDSQKAVKILQELPAKEFGSVLVKDIKGGLMAWATKIDSTFPQY

>tr|A0A7K8DDC7|A0A7K8DDC7_9CORV/1455 MOCS3 sulfurtransferase (Fragment) OS=Eulacestoma nigropectus OX=461239 GN=Mocs3 PE=3 SV=1RGMRERLAAELARGDSGDADEEDEDDDEEEEEEGALGFPAELPPLPARSALSAAEILRYSRQLVLPELGVRGQLRLARSSVLVVGCGGLGCPLAQYLAAAGVGRLGLVDHDVVETSNLQRQVLHGEARRGRPKARSAAAALRRLNSAVQYVPYRGALGPRSALRLVRQYDIVADCSDNVPTRYLVSDACVLAGKPLVSGSALRLEGQLVVYNYRGGPCYRCLFPRPPPPDAVTNCADGGVLGVVTGIVGCIQALEVLKIASGMGSSFSRSMLMFDALEGRFRNIKLRPKKPDCAVCGDNPSVTCLQDYEAFCGSSATDKCRTLQLLPGEDRISVQQYKELLDERVPHVLLDVRPQVEVDICRLEHAVHIPLSKLEEKNEESLQYLQKRICEEKQRTDDQTSLPVYVVCKLGNDSQKAVKILQQLPAKEFGSVLAKDIKGGLMAWATKIDSTFPQY

>tr|A0A7K8J757|A0A7K8J757_9PASS/1457 MOCS3 sulfurtransferase (Fragment) OS=Chaetorhynchus papuensis OX=254446 GN=Mocs3 PE=3 SV=1RGMRERLAAELARGDSGGDADEEDEDDDDEEKEEEGALGFPAELPPLPARSALSAAEILRYSRQLVLPELGVRGQLRLARSSVLVVGCGGLGCPLAQYLAAAGVGRLGLVDHDVVETSNLQRQVLHGEARRGRPKARSAAAALRRLNSAVQYVPYRGALGPRSALRLVRQYDIVADCSDNVPTRYLVSDACVLAGKPLVSGSALRLEGQLAVYNYRGGPCYRCLFPRPPPPDTVTNCADGGVLGAVTGIIGCMQALEVLKIASGMGSSFHQYMLMFDALEGRFRNIKLRPRKPDCAVCGDNPSVTCLQDYEAFCGSCATDKCRALRLLPSRDRISVQQYKELLDERVPHVLLDVRPQVEVDICRLEHAVHIPLRKLEEKNEESLRYLQKRICEEKQRTDGQTSVPVYVVCKLGNDSQKAVKILQELPAEEFGSVLAKDIKGGLMAWATKIDSTFPQY

>tr|A0A7L0L7B0|A0A7L0L7B0_9SYLV/1471 MOCS3 sulfurtransferase (Fragment) OS=Sylvietta virens OX=208069 GN=Mocs3 PE=3 SV=1QRSAAEQARGDAGDAEDEDGARGQRERSAAELARGDSGDADEEDDDEEEGALGFPAELPPLPARSALSAAEILRYSRQLVLPELGVRGQLRLARSSVLVVGCGGLGCPLAQYLAAAGVGRLGLVDHDVVETSNLQRQVLHGEARRGRPKARSAAAALRRLNSAVQYVPYRGALRPRSALRLVRQYDIVADCSDNVPTRYLVSDACVLAGKPLVSGSALRLEGQLVVYNYGGGPCYRCLFPEPPPPDTVTNCADGGVLGVVTGIIGCMQALEVLKIASGMGSSFNRYMLMFDALEGRFRNIKLRPKKADCAVCGDNPSVTCLQDYEAFCGSSATDKCRALRLLPSADRISVQQYKELLDERVPHVLLDVRPQVEVDICRLEHAIHIPLRKLEEKDEESLQHLQKRICEEKQRTDDQTSVPVYVVCKLGNDSQKAVKILQELPAEEFGSVLAKDIKGGLMAWATKIDSTFPQY

>tr|A0A7L1ZPR2|A0A7L1ZPR2_LEILU/1459 MOCS3 sulfurtransferase (Fragment) OS=Leiothrix lutea OX=36275 GN=Mocs3 PE=3 SV=1EEEDGARGLRERLAAELARGDSGDADEEDEDDDEEEEGTLGFPAELPPLPARSALSAAEILRYSRQLVLPELGVRGQLLLARSSVLVVGCGGLGCPLAQYLAAAGVGRLGLVDHDVVETSNLQRQVLHGEARRGRPKARSAAAALRRLNSAVQYVPYRALLRPRSALRLVRQYDIVADCSDNVPTRYLVSDACVLAGKPLVSGSALRLEGQLVVYNYRGGPCYRCLFPEPPPPDTVTNCADGGVLGVVTGIIGCMQALEVLKIASGMGSSFNQHMLMFDALEGRFRNIKLRPRKADCAVCGDSPSITCLQDYEAFCGSSATDKCRSLQLLPGGDRISVQQYKELLDARAPHVLLDVRPRVEVDICRLQHALHIPLRKLEEKDEESLQHLQKRISEEKQRTDDQTSVPVYVVCKLGNDSQKAVKILQELPAKEFGSVLAKDIKGGLMAWATKIDPTFPQY

>tr|A0A7L2BKM0|A0A7L2BKM0_9PASS/1472 MOCS3 sulfurtransferase (Fragment) OS=Alaudala cheleensis OX=670337 GN=Mocs3_0 PE=3 SV=1QPSAAEPARGDAGDAEGETRGLRERLAAELARGDSGDADEEDEDDENEEDESLGFPAELPPLPARSALSAAEILRYSRQLVLPELGVRGQLLLARSSVLVVGCGGLGCPLAQYLAAAGVGRLGLVDHDVVETSNLQRQVLHGEARRGRPKARSAAAALRRLNSAVHYVPYRGALRPRSALRIVRQYDIVADCSDNAPTRYLVNDACVLAGKPLVSGSALRLEGQLAVYNHRGGPCYRCLFPEPPPPDTVTNCADGGVLGVVTGVIGCMQALEVLKIASGMGSSFSQYMLMFDALEGRFRNIKLRPRRADCAVCGDSPSVTSLQDYEAFCGSSATDKCRALRLLPSGDRISVQQYKELLDGRVPHVLLDVRPRVEVDICRLEHALHIPLRKLEEKDEESLRQLQKRISEEKQRTDDQTSLPVYVVCKLGNDSQKAVKILQELPAQEFGSVVAKDIKGGLMAWATKIDSTFPQY

>tr|A0A7L3R1X3|A0A7L3R1X3_9SYLV/1472 MOCS3 sulfurtransferase (Fragment) OS=Cettia cetti OX=68486 GN=Mocs3 PE=3 SV=1QPPAAEPARGQAGDAEDEDGARGPRERPAAELARGDSGDADEEDDEEEEDGTLGFPAELPPLPARTALSAAEILRYSRQLVLPELGVRGQLRLARSSVLVVGCGGLGCPLAQYLAAAGVGRLGLVDHDVVETSNLQRQVLHGEARRGRPKARSAAAALRRLNSAVQYVPYRGALRPRSALRLVRQYDIVADCSDNVPTRYLVSDACVLAGKPLVSGSALRLEGQLVVYNHGGGPCYRCLFPEPPPPDTVTNCADGGVLGVVTGIIGCMQALEVLKIASGMGSSFNRYMLMFDALEGRFRNIKLRPRKADCAVCGDNPSVTCLQDYEAFCGSSATDKCRTLRLLPSGDRISVQQYKELLEERVPHVLLDVRPQVEVDICRLEHAVHIPLRKLEEKDEESLQRLQKRICEEKQRTDDQTSVPVYVVCKLGNDSQKAVKILQELPAKEFGSVLAKDIKGGLMAWATKIDSTFPQY

>tr|A0A7L3VLR7|A0A7L3VLR7_MOLAT/1475 MOCS3 sulfurtransferase (Fragment) OS=Molothrus ater OX=84834 GN=Mocs3 PE=3 SV=1QPSAAEPARGDAGDAEDEDGTRGLRERLAAELARGDSGDADEEDEDDEDEDEDGALGFPAELPPLPARSALSAAEILRYSRQLVLPELGVRGQLRLARSSVLVVGCGGLGCPLAQYLAAAGVGRLGLVDHDVVETSNLQRQVLHGEARRGRPKARSAAAALRRLNSAVQYVPYRGALRPRSALRLVRQYDIVADCSDNVPTRYLVSDACVLAGKPLVSGSALRLEGQLAVYNHRGGPCYRCLFPRPPPPDTVTNCADGGVLGAVTGIIGCLQALEVLKIASGMGSSFSGYMLMFDALEGRFRNIKLRPRRADCAVCGDSPSITCLQDYEAFCGSCATDKCRALQLLPGADRVSVQQYKALLDARAPHVLLDVRPPVEAEICRLPHALHIPLRRLQDRDPASLQRLQKRICEEKQRSDDQTPLPVYVVCKLGNDSQKAVKILQELPAEEFGSVLAKDIKGGLMAWATKIDSTFPQY

>tr|A0A7L4EDX1|A0A7L4EDX1_HIRRU/1477 MOCS3 sulfurtransferase (Fragment) OS=Hirundo rustica OX=43150 GN=Mocs3 PE=3 SV=1MAGAAAARLRAEIRRRERELRGLRERLAAELARGDTAEQEEDGEEEREREEGARAGTLGFPAELPPLPARSALSAAEILRYSRQLVLPELGVRGQLRLARSSVLVVGCGGLGCPLAQYLAAAGVGRLGLVDHDVVETSNLQRQVLHGEARRGRPKARSAAAALRRLNSAVQYVPYRGALRPRSALRIVRQYDIVADCSDNVPTRYLVSDACVLAGKPLVSGSALRLEGQLAVYNHGGGPCYRCLFPEPPPPDAVTNCADGGVLGVVTGVIGCMQALEVLKIASGMGSTFNGHMLMFDALEGRFRNIKLRPRRADCAVCGDSPSVTCLQDYEAFCGSSATDKCRALQLLPAGDRISVQRYKELLDERVPHVLLDVRPQVEVDICRLEHAVHIPLRKLEEKDEESLRHLQDRISEEKQRTDGQASVPVYVVCKLGNDSQKAVKILQELPAEEFGSVLAKDIKGGLMAWATKIDSTFPQY

>tr|D2GVM6|D2GVM6_AILME/1458 Molybdenum cofactor synthesis 3 (Fragment) OS=Ailuropoda melanoleuca OX=9646 GN=MOCS3 PE=3 SV=1MASREEVLALQAEVARREEELSSLKQRLAAAVLAEQEPERLVPVSPLPPRAALSRDEILRYSRQLVLPELGVHGQLRLATASVLVVGCGGLGCPLAQYLAAAGVGRLGLVDYDVVEMSNLARQVLHGEALAGQAKVFSAAASLRRLNSAVECVPYAQALTPATALDLVRRYDVVADCSDNVPTRYLVNDACVLAGRPLVSASALRFEGQITVYHYDGGPCYRCIFPQPPPAETVTNCADGGVLGVVTGVLGCLQALEVLKIAAGLGPSYSRSLLIFDALRGQFRCIQLRSRRPDCAACGERPTVTDLQDYEAFCGSSATDKCRSLQLLSPEERVSVTDYKRLLDSGSPHLLLDVRPQVEVDICRLPHALHIPLKHLERRDAQSLELLGEAIREGRQGTQEGAAFPVYVICKLGNDSQKAVKTLQSLTAVQELESLTVQDVVGGLMAWAAKVDGTFPQY

>tr|A0A7K8MCA9|A0A7K8MCA9_9CORV/1475 MOCS3 sulfurtransferase (Fragment) OS=Ptilorrhoa leucosticta OX=449384 GN=Mocs3 PE=3 SV=1MAGAAAAARLRAEIRRRERELRGLRERLAAELARGDAAEQEEDGEREREQEEGGALGFPAELPPLPARSALSAAEILRYSRQLVLPELGVRGQLRLARSSVLVVGCGGLGCPLAQYLAAAGVGRLGLVDHDVVETSNLQRQVLHGEARRGRPKARSAAAALRRLNSAVQYVPYRGALGPRSALRLVRQYDIVADCSDNVPTRYLVSDACVLAGKPLVSGSALRLEGQLVVYNYRGGPCYRCLFPRPPPPDTVTNCADGGVLGVVTGIIGCMQALEVLKIASGMGSSFNQCMLMFDALEGRFRNIKLRPKKADCAVCGDNPSVTRLQDYEAFCGSSATDKCRTLQLLPSKDRISVQQYKELMDEQVPHVLLDVRPQVEVDICRLEHAVHIPLSKLEEKNEESLQYLQERICKEKQRTDDQTSLPVYVVCKLGNDSQKAVKILQELPAEEFGSVLAKDIKGGLMAWATKIDSTFPQY

>tr|A0A7K9GX59|A0A7K9GX59_LOXLE/1317 MOCS3 sulfurtransferase (Fragment) OS=Loxia leucoptera OX=96539 GN=Mocs3 PE=4 SV=1AALRRLNSAVQYVPYRGALRPRSALRLVRQYDIVADCSDNVPTRYLVSDACVLAGKPLVSGSALRLEGQLAVYNYRGGPCYRCLFPEPPPPDTVTNCADGGVLGVVTGIIGCMQALEVLKIASGMGSSFGGYMLMFDALEGRFRNIKLRPRKADCAVCGDNPSVTCLQDYEAFCGSSATDKCRALRLLPSGDRVSVQQYKELLDARVPHVLLDVRPQVEAEICRLQHALHIPLRSLEEKDEQALQRLQKRICEEKQRTDDQTPLPVYVVCKLGNDSQKAVKILQELPAEEFGSVLAKDIKGGLMAWATKIDSTFPQY

>tr|A0A850V4R8|A0A850V4R8_9CORV/1475 MOCS3 sulfurtransferase (Fragment) OS=Chloropsis hardwickii OX=667144 GN=Mocs3 PE=3 SV=1QPSAAEAARGDAGDAEDEDGARGLRERLAAELARGDSGDADEEDEDDDEEQEEGALGFPAELPPLPARSALSAAEILRYSRQLVLPELGVRGQLRLARSSVLVVGCGGLGCPLAQYLAAAGVGRLGLVDHDVVETSNLQRQVLHGEARRGRPKARSAAAALRRLNSAVQYVPYRGALRPRSAMRLVRQYDIVADCSDNVPTRYLVSDACVLAGKPLVSGSALRLEGQLAVYNHGGGPCYRCLFPEPPPPDTVTNCADGGVLGVVTGIIGCLQALEVLKIASGMGSSFSGHMLMFDALEGRFRNIKLRPRKADCAVCGDNPSVTCLQDYEAFCGSSATDKCRTLQLLPGADRISVQRYKELLDARVPHLLLDVRPQVEVDICRLPHAVHIPLRKLEEKDEESLQLLQKMIGEEKQRTDGQTSLPVYVVCKLGNDSQKAVKILQELPAEEFGSVLAKDIKGGLMAWAAKIDSTFPQY

>tr|A0A851R2L3|A0A851R2L3_TYCCO/1475 MOCS3 sulfurtransferase (Fragment) OS=Tychaedon coryphoeus OX=614051 GN=Mocs3 PE=3 SV=1QPSAAELARGDAGHAEDEDGARGLRERLAAELARGDSGDADEEDEDDDEEEQDGALGFPAELPPLPARAALSAAEILRYSRQLVLPELGVRGQLRLARSSVLVVGCGGLGCPLAQYLAAAGVGRLGLVDHDVVETSNLQRQVLHGEARCGRPKARSAAAALRRLNSAVQYVPYRGALRPRSALRLVRQYDIVADCSDNVPTRYLVSDACVLAGKPLVSGSALRLEGQLAVYNHGGGPCYRCLFPEPPPPDTVTNCADGGVLGVVTGIIGCLQALEVLKIASGMGSSFHQYMLVFDALEGRFRNIKLRPRKADCAVCGERPSITCLQDYEAFCGSSATDKCRAVRLLPSADRISVQQYKELLDARAPHVLLDVRPRVEADICRLQHALHIPLRTLQEGDEQALQLLRKRICEEKRRTDGQSSLPVYVVCKLGNDSQKAVKILQELPAKEFGSVLAKDIKGGLMAWATKIDSTFPQY

>tr|A0A851VH17|A0A851VH17_9PASS/1475 MOCS3 sulfurtransferase (Fragment) OS=Copsychus sechellarum OX=797021 GN=Mocs3 PE=3 SV=1QPSAAEPARGDAGHAEDEDGARGLRERLAAELARGDSGDADEEDEDDDEEEQDGALGFPAELPPLPARAALSAAEILRYSRQLVLPELGVRGQLRLARSSVLVVGCGGLGCPLAQYLAAAGVGRLGLVDHDVVETSNLQRQVLHGEARCGRPKARSAAAALRRLNSAVQYVPYRGALRPRSALRLVRQYDIVADCSDNVPTRYLVSDACVLAGKPLVSGSALRLEGQLVVYNHGGGPCYRCLFPEPPPPDTVTNCADGGVLGVVTGIIGCLQALEVLKIASGMGSSFHQYMLVFDALEGRFRNIKLRPRKADCAVCGERPSITCLQDYEAFCGSSATDKCRAVRLLPGADRISVQQYRALLDARAPHVLLDVRPQVEADICRLQHALHIPLRKLQDRDEQALQLLQERIGEEKRRTDGQSSLPVYVVCKLGNDSQKAVKILQELPAEEFGSVLAKDIKGGLMAWATEIDSTFPQY

>tr|A0A851WIX3|A0A851WIX3_9CORV/1477 MOCS3 sulfurtransferase (Fragment) OS=Corvus moneduloides OX=1196302 GN=Mocs3 PE=3 SV=1QPSPAELARGDAGDAEDEDGARGMRERLAAELARGDSGDADEEDEDDDEEEEETEGALGFPAELPPLPARSALSAAEILRYSRQLVLPELGVRGQLRLARSSVLVVGCGGLGCPLAQYLAAAGVGRLGLVDHDVVETSNLQRQVLHGEARRGRPKARSAAAALRRLNSAVQYVPYRGALRPRSALRLVRQYDIVADCSDNVPTRYLVSDACVLAGRPLVSGSALRLEGQLVVYNYRGGPCYRCLFPRPPPPDTVTNCADGGVLGVVTGIIGCIQALEVLKIASGMGSSFSQSMLMFDALEGRFRNIKLRPKKADCAVCGDNPSITCLQDYEAFCGSSATDKCRTLQLLPSQDRISVHQYKELLDEQVPHVLLDVRPQVEVDICRLEHAVHIPLSKLEEKNEESMQYLQKRICEEKQRTDDQTSLPVYVVCKLGNDSQKAVKILQELPAKEFGSVLAKDIKGGLMAWATKIDSTFPQY

>tr|A0A852AK12|A0A852AK12_CALOR/1476 MOCS3 sulfurtransferase (Fragment) OS=Calcarius ornatus OX=198940 GN=Mocs3 PE=3 SV=1QPSAAEPARGDAGDAEDEDGARGLRERLAAELARGDSGDADEEGEDDDDDEEEEGSLGFPAELPPLPARSALSAAEILRYSRQLVLPELGVRGQLRLARSSVLVVGCGGLGCPLAQYLAAAGVGRLGLVDHDVVETSNLQRQVLHGEARRGRPKARSAAAALRRLNSAVQYVPYRGALRPRSALRLVRQYDIVADCSDNVPTRYLVSDACVLAGRPLVSGSALRLEGQLAVYNHRGGPCYRCLFPRPPPPDTVTNCADGGVLGAVTGIIGCLQALEVLKIASGMGSSFSGYMLMFDALEGRFRNIKLRPRRADCAVCGDSPSVTCLQDYEAFCGSSATDKCRALQLLPGADRLSVQQYKALLDARVPHVLLDVRPPVEAEICRLPHALHVPLRCLQERDPDSLRRLQKRICEEKQRTDDQTPLPVYVVCKLGNDSQKAVKILQELPAEEFGSLLAKDIKGGLMAWATKIDSTFPQY

>tr|A0A852IDS0|A0A852IDS0_9PASS/1470 MOCS3 sulfurtransferase (Fragment) OS=Nicator chloris OX=237433 GN=Mocs3 PE=3 SV=1AELARGDARDAEDEDGARGLRERLAAELARGDSGDADEEDEDDDEEEEGALGFPAELPPLPARSALSAAEILRYSRQLVLPELGVRGQLRLARSSVLVVGCGGLGCPLAQYLAAAGVGRLGLVDHDVVETSNLQRQVLHGEARRGRPKARSAAAALRRLNSAVQYVPYRGALRPRSALRLVRQYDIVADCSDNVPTRYLVSDACVLAGKPLVSGSALRLEGQLVVYNYRGGPCYRCLFPQPPPPDTVTNCADGGVLGVVTGIIGCMQALEVLKIASGMGSSFNQYMLMFDALEGRFRNIKLRPKKADCAVCGDNPSVTCLQDYEAFCGSSATDKCRTLQLLPSGDRISVQQYKELLDQRVPHVLLDVRPQVEVDICRLEHAVHIPLRKLEEKDEESLQHLLKRISEEKQRTDDQTSLPVYVVCKLGNDSQKAVKILQELPAKEFGSVLAKDIRGGLMAWATKIDSTFPQY

>tr|A0A852MXX4|A0A852MXX4_9PASS/1473 MOCS3 sulfurtransferase (Fragment) OS=Pteruthius melanotis OX=357074 GN=Mocs3 PE=3 SV=1QPSAAELARGDAGDAEDEDGARGMRERLAAELARGDSGDADEEDEDDDEEEGALGFPAELPPLPARSALSAAEILRYSRQLVLPELGVRGQLRLARSSVLVVGCGGLGCPLAQYLAAAGVGRLGLVDHDVVETSNLQRQVLHGEARRGRPKARSAAAALRRLNSAVQYVPYRGALGPRSALRLVRQYDIVADCSDNVPTRYLVSDACVLAGKPLVSGSALRLEGQLVVYNYRGGPCYRCLFPRPPPPDTVTNCADGGVLGVVTGIIGCIQALEVLKIASGMGSSFNQYMLMFDALEGRFRNIKLRPKKPDCAVCGDNPSVTCLQDYEAFCGSSATDKCRTLQLLPSEDRISVQQYKELLDEQVPHVLLDVRPQVEVEICRLEHAVHIPLSKLEGKDEESLQYLQKRICEEKQRTDDQTSLPVYVVCKLGNDSQKAVKILQELPAKEFGSVLAKDIRGGLMAWATKIDSAFPQY

>tr|A0A852NMY5|A0A852NMY5_9PASS/1466 MOCS3 sulfurtransferase (Fragment) OS=Atrichornis clamosus OX=449594 GN=Mocs3 PE=3 SV=1AMAGAGVARLRAEIRRRERELRGLRERLAAELEDDDDDNDDDDEGTFGFPAELPPLPARSALSAAEILRYSRQLVLPELGVRGQLRLARSAVLVVGCGGLGCPLAQYLAAAGVGRLGLVDHDVVETSNLQRQVLHGEARRGRPKARSAAAALRRLNSAVHYVPYPGALRPRSALRLVRQYDVVADCSDNVPTRYLVNDACVLAGKPLVSGSALRLEGQLAVYNHRGGPCYRCLFPQPPPPDTVTNCADGGVLGVVTGIIGCIQALEVLKIASGMGSSFNRFMLMFDALEGRFRNIKLRPKKPDCAVCGDNPTVTSLQDYEAFCGSSATDKCRTLRLLPSKDRISVEQYKKLLDERVPHVLLDVRPQVELDICRLEHAVHIPLSRLEDKDEEHLQHLQKRICEEKQRINDQASLPVYVVCKLGNDSQKAVKILQELPAKEFGSVLAKDIKGGLMAWATKIDSTFPQY

>tr|A0A8I3PZC3|A0A8I3PZC3_CANLF/1498 Molybdenum cofactor synthesis 3 OS=Canis lupus familiaris OX=9615 GN=MOCS3 PE=4 SV=1MSTYHFPPQTPWPRFSLFQSLGDGKPEPEVPFRTTSRSGAMASREEVLALQAEVARREEELSSLKQRLAAALLAEQAPERRVPVSPLPPKAALSREEILRYSRQLVLPELGVHGQLRLAAASVLVVGCGGLGCPLAQYLAAAGVGRLGLVDYDVVEMSNLARQVLHGEALAGQAKAFSAAASLRRLNSAVECVPYAQALTPATALDLVRRYDVVADCSDNVPTRYLVNDACVLAGRPLVSASALRFEGQITVYHYDGGPCYRCVFPQPPPAETVTNCADGGVLGVVTGVLGCLQALEVLKIAAGLGPSYSRSLLIFDALRGQFRCIQLRSRRLDCAACGERPTVTALQDYEAFCGSSATDKCRSLQLLSPEERVSVADYKRLLDSGSPHLLLDVRPQVEVDICRLPHALHIPLKHLERRNAESLKLLGEAIREGKQGAQAGAAFPVYVICKLGNDSQKAVKILQSLTAVQELESLTVQDVVGGLMAWAAKIDGTFPQY
